# Creation of an Anti-Inflammatory, Leptin-Dependent Anti-Obesity Celastrol Mimic with Better Druggability

**DOI:** 10.3389/fphar.2021.705252

**Published:** 2021-08-30

**Authors:** Bo Zhou, Yaxia Yuan, Le Shi, Sheng Hu, Dong Wang, Yang Yang, Yuanhu Pan, Dexin Kong, Alexander N. Shikov, Pierre Duez, Moonsoo Jin, Xiaohua Li, Xuebo Hu

**Affiliations:** ^1^ Laboratory of Natural Medicine and Molecular Engineering, College of Plant Science and Technology, Huazhong Agricultural University, Wuhan, China; ^2^ Department of Pharmacodynamics, College of Pharmacy, University of Florida, Gainesville, FL, United States; ^3^ Hubei Cancer Hospital, Wuhan, China; ^4^ Agricultural Bioinformatics Key Laboratory of Hubei Province, College of Informatics, Huazhong Agricultural University, Wuhan, China; ^5^ National Reference Laboratory of Veterinary Drug Residues and MAO Key Laboratory for Detection of Veterinary Drug Residues, Huazhong Agricultural University, Wuhan, China; ^6^ Department of Pharmaceutical Formulations, St. Petersburg State Chemical Pharmaceutical University, St. Petersburg, Russia; ^7^ Unit of Therapeutic Chemistry and Pharmacognosy, University of Mons, Mons, Belgium; ^8^ Department of Radiology, Weill Cornell Medical College, New York, NY, United States

**Keywords:** natural products, obesity, celastrol, *Glycyrrhiza uralensis*, glycyrrhetinic acid, inflammation, *Tripterygium wilfordii*

## Abstract

Obesity is characterized by an excessive body mass, but is also closely associated with metabolic syndrome. And, so far, only limited pharmacological treatments are available for obesity management. Celastrol, a pentacyclic triterpenoid from a traditional Chinese medicine (*Tripterygium wilfordii* Hook.f.), has shown remarkable potency against obesity, inflammation and cancer, but its high toxicity, low natural abundance and tedious chemical synthesis hindered its translation into clinics. In the present work, a triterpenoid library was screened for compounds with both high natural abundance and structural similarity to celastrol; from this library, glycyrrhetinic acid (GA), a compound present in extremely high yields in *Glycyrrhiza uralensis* Fisch. ex DC., was selected as a possible scaffold for a celastrol mimic active against obesity. A simple chemical modification of GA resulted in GA-02, a derivative that suppressed 68% of food intake in diet-induced obesity mice and led to 26.4% weight loss in 2 weeks. GA-02 plays a role in obesity treatment by re-activating leptin signaling and reducing systemic and, more importantly, hypothalamic inflammation. GA-02 was readily bioavailable with unnoticeable *in vitro* and *in vivo* toxicities. The strategy of scaffold search and modification on the basis of bio-content and structural similarity has proved to be a green, economic, efficient and practical way of widening the medicinal applications of “imperfect” bioactive natural compounds.

## Introduction

Widespread obesity has attracted vast attention all over the world. Based on statistics from the World Health Organization, there were over 1.9 billion people over-weighted and 650 million were obese in 2016 (World Health Organization, 2021). These figures might continuously grow even higher as the global obesity population has nearly tripled since 1975 (World Health Organization, 2021). Obesity is considered a major driver for many metabolism-related diseases, including steatohepatitis, diabetes, cardiovascular and neurodegenerative diseases, as well as cancers (Finucane et al., 2011; Swinburn et al., 2011). Although there is an urgent need for drugs that control obesity, only limited pharmacological targets have so far proved druggable (Bocarsly, 2018).

Leptin, a metabolism regulation hormone, is encoded by *ob* gene and secreted by adipocytes in white adipose tissue for maintaining energy homeostasis and body weight, though binding to its receptors in hypothalamus (Ahima et al., 1996; Dietrich and Horvath, 2013; Pan and Myers, 2018; Friedman, 2019). High concentrations of circulating leptin with obvious fat accumulation was observed in obese human subjects or rodents with consumption of high-fat diet (HFD) (Frederich et al., 1995). This has led to a notion of “leptin resistance” or “leptin insensitivity,” as endogenous or exogenous leptin seems unable to control appetite and body weight (Considine et al., 1996; El-Haschimi et al., 2000). Interestingly, excess energy intake has been shown to enhance the expression of inflammatory cytokines in the hypothalamus (Valdearcos et al., 2015). Inflammation in metabolic organs is a well-known consequence of obesity (Wellen and Hotamisligil, 2005; Olefsky and Glass, 2010; Lumeng and Saltiel, 2011), but inflammation in hypothalamus precedes the onset of obvious obesity, occuring much earlier than the inflammatory response in peripheral tissues (Thaler et al., 2012). Hypothalamic inflammation effectively contributes to leptin resistance and subsequent obesity development (De Souza et al., 2005; Zhang et al., 2008; Posey et al., 2009). Also, intracerebroventricular tumor necrosis factor (TNF) was found to promote food intake and increased body weight in a rodent model (Romanatto et al., 2007); whereas treatment with anti-TNF agents reduced body weight gain under HFD, TNF receptor-deficient mice were shown resistant to HFD-induced body weight gain (Arruda et al., 2011). All these results suggest that suppressing hypothalamic inflammation might be a key towards new approaches for treating obesity.

Celastrol, a friedelane-type pentacyclic triterpenoid of natural origin, has distinct activities in inflammatory diseases (Kannaiyan et al., 2011; Guo et al., 2017). Recently, celastrol was found to be a highly effective anti-obesity agent (Liu et al., 2015). In HFD-induced obese mice, celastrol (100–150 μg/kg; i.p.) reduced body weight by 45–50% and alleviated insulin insensitivity and liver damage (Liu et al., 2015). Despite this therapeutic potential, further clinical applications of celastrol may be impeded by (i) adverse pharmacological properties, notably linked to the sympathetic nervous system; celastrol showed systemic toxicity (mice; oral; LD_50_, 20.5 mg/kg; 40% mortality at 4 mg/kg) with serious brain, heart and liver damage (mice; oral; 1 mg/kg) (Cascão et al., 2017); and (ii) limited availability; celastrol can be isolated from the root of thunder god vine (*Tripterygium wilfordii* Hook. f.), but it is found at relatively low concentrations (0.0003–0.05%). Recently, the total synthesis of celastrol was reported; however, as many as 28 steps of chemical reactions were required, under harshly catalytic conditions, with an overall yield of only 0.48% (Camelio et al., 2015).

To further study celastrol-type compounds, the present work proposes to screen a library of triterpenoids for a new scaffold amenable to hemisynthesis to investigate potential anti-obesity properties. The resulting best template was glycyrrhetinic acid (GA), which had an extremely high yield in *Glycyrrhiza uralensis*, the monarch drug in traditional Chinese herb medicine over centuries. GA-02, a derivative of GA, was obtained by a few mild chemical reactions. While showing outstanding anti-obesity efficacy, GA-02 exhibited significantly lower toxicity and superior availability than celastrol, which may have paved an easy way towards drugging.

## Material and Methods

### Material

Antibodies used in the experiment included p-STAT3 (Tyr705) (Cat. 9145, CTS; Clone D3A7; 1:3,000 dilution for IHF staining), anti-TNF-α (Cat. GB11188, Service; 1:100 dilution for IHF staining), anti-IL-1β (Cat. GB11113, Service; 1:100 dilution for IHF staining) and p-HSL (Cat. 4126S, CST;1:100 dilution for IHF staining). Celastrol (98.0%) was purchased from Sigma. Glycyrrhetinic acid was from Huaian Brothers Biological Science. Foetal bovine serum (FBS) was from NTC (NCT-HK014, Natocor, Cordoba, Argentina). DMEM (SH30022.01) was purchased from Hyclone. Penicillin/streptomycin was obtained from Biosharp (Shanghai, China). Hydrocortisone and recombinant hEGF were purchased from Sangon Biotech (Shanghai, China). FITC, MTT and DMSO were all purchased from Sigma-Aldrich (China).

### Structural Similarity Screening of Natural Triterpenoids

The chemical and pharmacological information on 1,480 natural triterpenoids were collected from published literature. For each compound, a “bio-content” value was assigned, according to the published % w/w in their respective herbal drug of origin; the structural information was obtained though PubChem. Using default feature of extensive-connectivity fingerprint four module of Pipeline Pilot 8.5 software (Papadatos et al., 2014) for molecular similarity assessment, structural similarity scores were calculated for each chemical, using celastrol as a probe (Supplementary Tables S1, S2). By two-dimensional scattered distribution, the 1480 triterpenoids and celastrol were arrayed with logarithmic of bio-content values as the *x* axis and structural similarity scores as *y* axis.

### Molecular Docking

The molecular docking of celastrol and GA-02 to Nur77 (nerve growth factor IB, NGFIB; PDB code: 4JGV) was studied with AutoDock version 4.2 (Morris et al., 2009), using the Lamarckian genetic algorithm. The running number was set to 200, and each individual run number was set to 300. The maximum energy evaluation was 25,000,000, and 300,000 for a maximum of generations. The grid center was chosen according to reported THPN coordinates (−12.08, 18.29, −4.233) of Nur77 structure. The grid box size was 100 × 80 × 80 points in each axis and the grid space was set at 0.2 Å. The top 20 docking conformations were further relaxed by MD simulations using Amber package (Case et al., 2012). After 10,000 steps of energy minimization, short MD simulations were conducted on each of the bound structures for 50 ps. The backbone atoms of Nur77 were fixed with a force constant (30 kcal/mol) during simulations. Then Poisson-Boltzmann Surface Area approach and Molecular Mechanics (Rastelli et al., 2010) were used to evaluate the binding free energy of all binding conformation candidates, and the best binding modes of celastrol and GA-02 were selected according to the binding energy. PyMOL version 2.40 and PoseView were used for molecular visualization.

### Protein Expression and Surface Plasmon Resonance

Plasmid encoding Nur77 ligand binding domain (Nur77-LBD) fusion with glutathione S-transferase (GST) (Zhan et al., 2012) was a generous gift from Professor Qiao Wu, Xiamen University. The plasmid was transformed into *Escherichia coli* BL21 and the protein Nur77-LBD was purified with Glutathione Agarose (Thermo Fisher Scientific) with a standard GST fusion protein purification procedure. The protein was further purified by size-exclusion chromatography (Bio-Rad) and then stored at −70°C before use. The binding kinetics of GA-02 to purified Nur77-LBD was measured by surface plasmon resonance (SPR; OpenSPR, Nicoyalife). Simply, Nur77-LBD was immobilized to a GST sensor chip (Nicoyalife). Analyte at indicted concentrations in phosphate buffer saline (PBS) running buffer, pH 8, was flowed over the sensor chip at 20 μl/min for 5 min to allow interaction. The data from the control was collected exactly as in the experiment. Binding kinetic parameters were obtained by fitting the curve to a one-to-one binding model using TraceDrawer (Nicoyalife) software.

### Cell Culture

HMEC-1 cells were maintained in cell culture medium (MCDB 131, Sigma) supplemented with 10% FBS, l-glutamine (10 mM), 1% penicillin/streptomycin, hydrocortisone (1 mg/ml) and recombinant hEGF (10 ng/ml). To induce the ICAM-1 expression, LPS (1 μg/ml, 055:B5, Sigma) was added to the culture medium. HEK293T, L02 and H9C2 cells were cultured in DMEM supplemented with 10% FBS and 1% penicillin/streptomycin. The cells were maintained at 37°C with 5% CO_2_. Cells were normally passaged two times per week.

### Flow Cytometry

The expression of ICAM-1 on the surface of LPS-induced HMEC-1 was detected and quantified by flow cytometry according to a previous study. (Hu et al., 2012). In brief, HMEC-1 cells were inoculated into 48-well plates (2  ×  10^5^ cells/well) in 0.2 ml complete MCDB131 culture medium. When they reached about 80% confluency, cells were treated with tested chemicals at different concentrations (all in 0.1% DMSO) for 3 h. Celastrol was applied as a positive control. Then the cells were treated with LPS (1 μg/ml) for 12 h. Cells were harvested and then washed with PBS, followed by 5% BSA in PBS for 30 min for blocking. Then cells were stained with fluorescence-labeled anti-ICAM-1 antibody (LB-2, Santa Cruz). The fluorescence intensity was then determined using flow cytometry (Guava easyCyte H8, EMD Millipore).

### Dual-Luciferase Reporter Assay

The dual-luciferase reporter assay was conducted as previously described (Huang et al., 2017). HEK293 cells cultured in DMEM were inoculated into 24-well plates (5 × 10^4^ cells/well) and were transiently transfected with a plasmid encoding NF-κB-controlled luciferase plasmid using lipofectamine 3000 (Invitrogen, L3000-001). Cells were treated with GA-02 or celastrol in triplicate for 5 h, then activated by TNF-α (10 ng/ml) for 24 h. Cells were harvested and the luciferase activity was determined according to the manufacturer instructions. In addition, HEK293 cells were pre-treated with LPS (1 μg/ml) for 6 h and then stimulated with GA-02 or celastrol for 3 h. Total RNA was extracted and reverse transcribed into cDNA using reverse transcription kit (TRUEscript). The target gene mRNA transcript level, relatively to the GAPDH control, was measured by QT-PCR using specific primers (Supplementary Table S3). The qPCR data were analyzed by 2^−△△Ct^ method.

### MTT Assay

To determine cell viability, cells were seeded into 96-well plate (1 × 10^4^ cells/well) for 24 h before stimulation. Cells were treated with GA-02 and celastrol at different concentrations (DMSO less than 0.1%) for 24 h. The cell viability were determined by MTT assay (Kumar et al., 2018). All experiments were performed in triplicate. The IC_50_ value was calculated with GraphPad prim 8.

### Microarray Gene Expression Profiles in the Hypothalamus of Diet-Induced Obese Mice

After the acclimation period, diet-induced obese (DIO) mice were administered with vehicle, celastrol (100 μg/kg) or GA-02 (20 mg/kg) intraperitoneally for continuous 4 days. Mice were sacrificed 14 h after the last injection and the hypothalamus was extracted and kept frozen in liquid nitrogen until total RNA extraction. The total RNA was extracted and the transcriptome sequencing was conducted by UniqueGene (Wuhan, China).

### Animal Experiments

The animal experiments were conducted according to the guidelines for animals care of Huazhong Agricultural University (Wuhan, China), and were approved by the Institutional Animal Care and Ethic Committee (HZAUMO-2018-043). Male C57BL/6J mice were obtained from Disease Control and Prevention Center of Hubei province. Male leptin-deficient (*ob/ob*) and leptin receptor-deficient (*db/db*) mice, 6 weeks old, were obtained from the National Resource Center of Model Mice (Nanjing, China). High fat diet was purchased from Medicine Bio-mart Corp (Medicine Bio-mart, Jiangsu, China). Mice were housed in a pathogen-free facility (12 h dark/light cycle) with free access to food and water.

### High Fat Diet and GA-02 Treatment

Six-weeks-old male C57BL/6J mice with similar body weight and blood glucose level were randomized into different groups. After feeding with HFD for 14 weeks, the mice were treated with GA-02 at the doses of 4, 12, 20 mg/kg by intraperitoneal injection once a day for 2 weeks. The mice were maintained with HFD throughout the experiment. The body weight and daily food intake were measured and recorded every day.

For intraperitoneal treatment, mice were administered with 25 μl of vehicle (DMSO) for 3 days as acclimation before GA-02 treatment. GA-02 was dissolved and administered to mice in 25 μl DMSO. Vehicle groups received 25 μl of DMSO as control. For oral administration, mice were fed with vehicle (100 μl PBS) for 4 days before GA-02 or vehicle treatment. GA-02 was administered to mice in 100 μl PBS. The treatments were performed 1 h before dark cycle.

### Body Composition Measurement

After 14-days treatment with GA-02 or vehicle (DMSO), the fat and lean masses were assessed on a MiniQMR (QMR21-070H, NIUMAG, China) NMR analyzer. Briefly, the MiniQMR analyzer was calibrated according to the manufacturer’s instruction. After anesthesia (10% chloral hydrate, 0.1 ml/10 g body weight), the body weights were recorded and then the mice were subjected to body fat and lean mass measurement.

### Hematoxylin and Eosin Staining

All tissues (except the adipose tissue) were harvested and fixed in 4% paraformaldehyde; the adipose tissue was fixed in adipose tissue fixative (G1119, Servicebio (Wuhan, China) for 48 h. After dehydration, the tissues were embedded in paraffin, and dissected into 5 μm thick sections, then stained with hematoxylin/eosin (H&E) and imaged under light microscope (model 3D HISTECH Pannoramic 250).

### Glucose Tolerance and Insulin Tolerance Test

At the end of the experiment, the glucose tolerance (GTT) and insulin tolerance (ITT) were quantified. Briefly, for GTT, mice were fasted for 12 h, and glucose (1 g/kg) was administered intraperitoneally. The blood glucose was measured at 0, 30, 60, 90, and 120 min after glucose injection by a Glucometer from the tail vein blood sample. For ITT assay, 6 h fasted mice were treated with recombinant human insulin (1 IU/kg, Lilly) intraperitoneally. Blood glucose was measured according to the same method as for GTT.

### Blood Biochemical Assay

Blood sample was collected from vena ophthalmica, transferred to heparinized tubes, and centrifuged (3,000 rpm at 4°C) for 15 min alanine aminotransferase (ALT), aspartate transaminase (AST), glucose, total cholesterol (TC), triglyceride (TG), urea, creatinine and total protein (TP) levels were measured on plasma using an automated blood biochemistry analyzer BS-240 (Bio-Medical Electronics). Hematological parameters were measured on a BC-5000VET system (Bio-Medical Electronics).

### Norepinephrine (NE) Quantification

Epididymal and subcutaneous tissues were harvested and washed in PBS for 10 min and then 100 mg of each sample were cut and shred by scissors. The sample was then added with 1 ml RIPA lysis buffer and homogenized by a handhold homogenizer for 10 min at 4°C. Twenty minutes later, the sample was spun down at 8,000 rpm for 10 min at 4°C. The total protein concentration in the supernatant was then quantified by bicinchoninic acid assay as before (Hu et al., 2012). The NE was then quantified by ELISA according to a manufacture kit (4A Biotech, China).

### Statistical Analysis

The data were expressed as mean ± SEM for the body weight and mean ± SD for other experiments. The comparisons between two groups were analyzed for significance by two-way analysis of variance (ANOVA) and Dunnet’s or Bonferoni using GraphPad Prism 8. The Statistical significance was accepted at *p < 0.05, **p < 0.01, ***p < 0.001.

## Results

### Structural Similarity Screening of Natural Triterpenoids for a Celastrol Analogue

**FIGURE 1:**
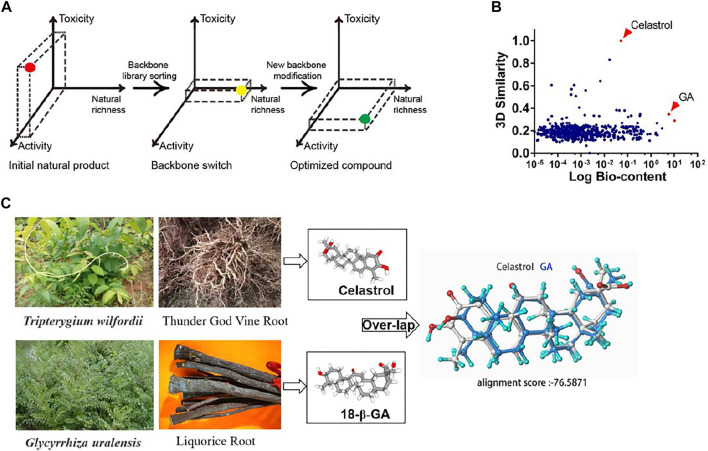
Scaffold similarity search: from celastrol to GA. **(A)** Schematic diagram of the similarity search strategy. **(B)** Scatter diagram analysis of a pool of 1,380 triterpenoids. The *x* axis represents the logarithmic value of the triterpenoids bio-content (published % w/w in their respective herbal drugs of origin). The *y* axis corresponds to the computed structural similarity score relatively to celastrol. **(C)** The plant source and stero-structure of celastrol and GA.

With over 20,000 members repertoried, triterpenoids represent one of most versatile chemical groups produced by various species throughout the plant and microbial kingdoms (Dzubak et al., 2006); many of them have medicinal potential but rarely with a balance of activity, availability and toxicity. To overcome the problems raised by celastrol, we proposed a strategic 2-step rationale: (i) an appropriate structural similarity screening against a triterpenoid library (determination of structural similarity scores from 3D structures), taking into account their natural abundances and pharmacological activities, with a focus on moderately active compounds; and (ii) the optimization of an ideal candidate by a few steps of hemisynthesis (Figure 1A). From a global analysis of literature, a total of 1,380 natural triterpenoids were collected with their published contents (% w/w) in their respective herbal drugs of origin (Supplementary Table S1). These structures were tested for similarity to celastrol by determining similarity scores through Pipeline Pilot 8.5 (Supplementary Table S2). A two-dimensional comparison yielded glycyrrhizic acid and 18-β-glycyrrhetinic acid (GA) as the celestrol analogs with the highest proportions in their source drugs (Figure 1B). GA, displaying a higher celastrol structural similarity, was selected as template for further structure modification (Figure 1C). Although some of tested triterpenoids, such as pristimerin, exhibited even higher structural similarity scores, they were not chosen as scaffolds because of their low natural abundance, indicating unlikely commercial availability and further drug development possibilities (Supplementary Figure S1). Additionally, GA, a major bioactive pentacyclic triterpene extracted from the underground rhizome of licorice (*Glycyrrhiza* spp.), has a long tradition of medicinal use (Tahara et al., 1971; Asl and Hosseinzadeh, 2008).

### Generation of GA-02

Obesity is strongly associated with chronic metabolic inflammation (Hotamisligil, 2006; Lumeng et al., 2007) and celastrol distinctively inhibits inflammatory diseases (Kannaiyan et al., 2011; Guo et al., 2017); and so we investigated the anti-inflammatory activities of a panel of commercially available pentacyclic triterpenoids, that present structural similarity with celastrol, through their impact on lipopolysaccharides induced expression of intercellular adhesion molecule-1 (ICAM-1) in human microvascular endothelial cells (HMEC-1) (Zhang et al., 2018).

Although the activity of GA was marginally higher than other tested compounds, its anti-inflammatory activity was much lower compared to celastrol (Supplementary Figure S1). Literature data indicate that several triterpenoids, bearing a conjugated carbonyl and a nearby hydroxyl group on ring A, exhibit decent anti-inflammatory (Kaileh et al., 2007; Abdelwahab et al., 2011) and anti-obesity activities (Lee et al., 2016; Murtaza et al., 2017) (Supplementary Figure S2). Based on this observation, we hypothesized that introducing these moieties on the ring A of GA may increase its activity. We carried out structural modifications from GA and the resulting compound GA-02 was verified by nuclear magnetic resonance and mass spectroscopy (Supplementary Figures S3, S4A–C, S5A–G).

### GA-02 Resembles Celastrol in a Receptor-Binding Model

**FIGURE 2:**
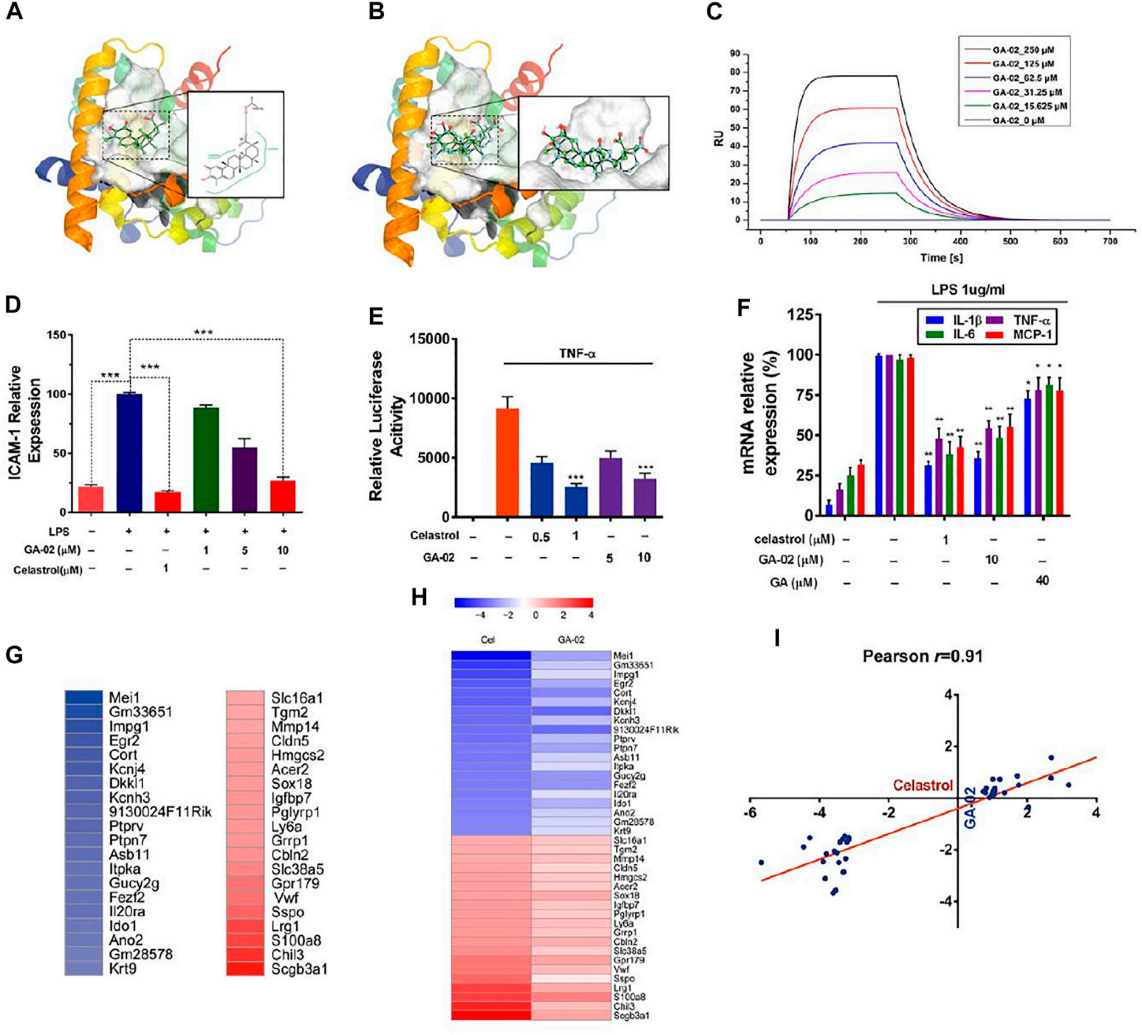
GA-02 mimics celastrol in receptor binding and key gene expression. **(A)** The binding mode of GA-02 (cyan ball-and-stick model) with Nur77 generated by PoseView. Nur77 (PDB code 4JGV) is shown as ribbon diagram, and GA-02 as green ball-and-stick model. **(B)** The van der Waals surface of superimposed celastrol (green) and GA-02 (cyan) in the Nur77 binding site, and the superimposed celastrol (green) and GA-02 (cyan) in ball-and-stick style. **(C)** GA-02 binding to Nur77 was illustrated by SPR assay of purified Nur77-LBD with GA-02. **(D)** Anti-inflammatory activity of GA-02 quantified by ICAM-1 level in LPS-treated endothelial cells. **(E)** Effects of GA-02 on NF-κB signaling. **(F)** The relative levels of *IL-1β*, *IL-6*, *TNF-α* and *MCP-1* mRNAs detected by RT-PCR. **(G)** A heat map of the most up-regulated (red) and down-regulated (blue) genes in the hypothalamus of celastrol-treated DIO mice. **(H)** The relative gene expression of the corresponding genes in the hypothalamus of GA02-treated DIO mice. The color scales show the celastrol- and GA-02-induced logFC in gene expression. **(I)** Logarithmic fold changes of the celastrol-versus GA-02-induced gene expression changes in the hypothalamus. The line indicates the best fit with its Pearson product–moment correlation coefficient (*r*).

Previous research has shown that celastrol exhibit potent anti-inflammatory activities via binding with a high affinity (Kd = 0.29 μM) to the receptor Nur77 and inducing autophagy of damaged mitochondria (Hu et al., 2017). Molecular docking indicate that GA-02 and celastrol present similar binding with the target protein Nur77-LBD (Figures 2A,B). Both molecules fit into a hydrophobic cavity of Nur77; their hydrophobic moieties interacting with hydrophobic amino acids while their hydrophilic moieties form hydrogen bonds with polar residues or are exposed to solvent. GA-02, compared to celastrol, presents (i) a ketone group on ring C that does not interfere with receptor binding, as it is on the solvent-exposed side; (ii) an additional methyl group in ring A of GA-02, favorable for interaction with the hydrophobic binding site. Meanwhile, the modified polar groups on ring A of GA-02 maintain similar solvent exposure as the corresponding groups in celastrol. Although the modifications introduced in GA-02 slightly perturb the conformation of the 5-rings system compared to celastrol, GA-02, as expected, maintains a similar binding configuration. The calculated free energies of these two compounds binding to the receptor were very close, i.e. −33.3458 and −33.6608 kcal/mol for GA-02 and celastrol, respectively. While in the SPR analysis, the affinity of GA-02 to Nur77 was relatively low with a Kd of 101 μM (Figure 2C), on the other hand, celastrol was measured at 0.53 μM (Supplementary Figure S6).

Based on ICAM-1 level in LPS-treated HMEC-1, the anti-inflammatory activities of GA-02 at 10 μM and celastrol at 1 μM were comparable (Figure 2D). Previously, a study showed that celastrol inhibit inflammation though the NF-κB pathway by inhibing IκB kinase, suppressing IκB phosphorylation and ubiquitin-mediated degradation (Hu et al., 2017). To explore the possible role of GA-02 in NF-κB signaling, we transfected HEK293 cells with a NF-κB luciferase reporter gene. In this model, both GA-02 and celastrol treatments significantly suppressed TNF-α activated luciferase activity (Figure 2E). Furthermore, the inductions of *TNF-α*, *MCP-1*, *IL-1β*, and *IL-6*, known to be mediated by NF-κB signaling upon LPS treatment, were decreased by both GA-02 and celastrol (Figure 2F).

In gene expression profiles obtained from the hypothalamus of diet-induced obese (DIO) mice treated with celastrol and GA-02, the expressions of the 20 genes mostly up- or down-regulated by celastrol treatment were compared using Pearson product-moment correlation analysis (Figures 2G,H). For these genes, the hypothalamic gene expression signature profiles were highly correlated between celastrol and GA-02 with an *r* value of 0.91 (Figures 2G–I), indicating that, *in vivo*, GA-02 and celastrol may play a similar anti-obesity role. No other genes were significantly up- or down-regulated by GA-02, confirming this probably similar activity profile.

### GA-02 Reduces the Body Weight and Daily Food Intake in DIO Mice

**FIGURE 3:**
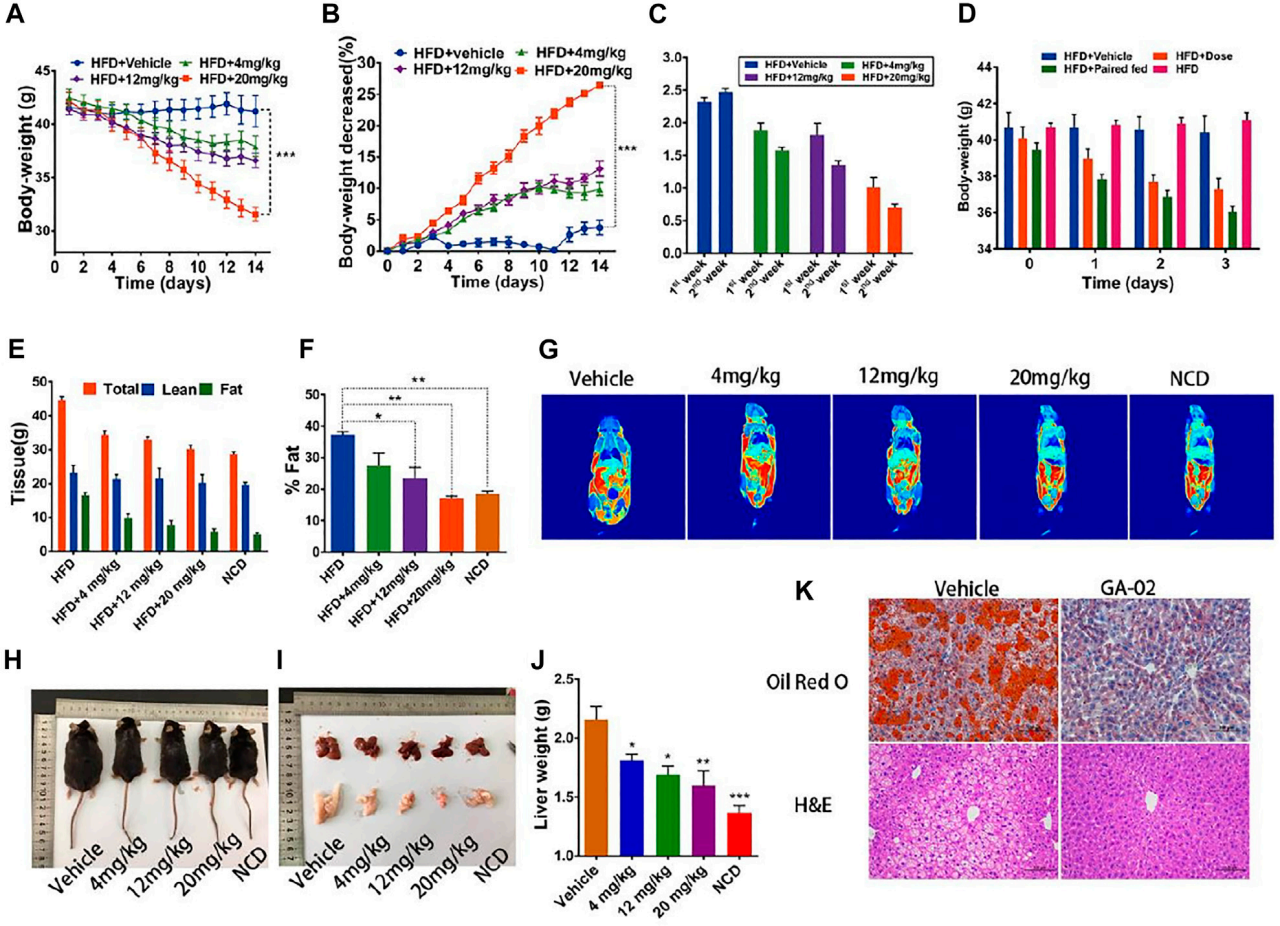
GA-02 reduces the body weight of DIO mice. DIO mice received vehicle or GA-02 for 2 weeks. **(A)** The body weight, **(B)** the change percentage of body weight (%), the experiments **(A,B)** were repeated with two cohorts (*n* = 6 for each group). **(C)** The daily food intake in the first and second week for each group. **(D)** The body weight changes in the first 3 days. **(E)** The total tissue weight and deduced lean mass and **(F)** fat mass by MRI after a 2-weeks treatment (*n* = 3 for each group), which was repeated in two cohorts. **(G)** Representative MRI imaging of *in vivo* mice fat. **(H)** The representative images of each dose group after a 2-weeks treatment. **(I)** The representative images Liver and epididymal fat pad. **(J)** Liver and weight for each treated group (*n* = 4 for vehicle group; *n* = 3 for each GA-02 group); **(K)** The H&E and Oil Red O staining of liver sections after high dose of GA-02 treatment.Scale bar, 100 µm (*p < 0.05, **p < 0.01, ***p < 0.001).

To evaluate the anti-obesity activity of GA-02, DIO-mice were intraperitoneally treated with GA-02. Groups of obese mice were treated with GA-02 at 4, 12, and 20 mg/kg, respectively. Tested doses are higher compared to celastrol, based on the potency differences observed *in vitro.* As expected, the body weights of all treated mice were significantly reduced during the 14 days experiment (Figure 3A, *p* = 0.0058, *p* = 0.0005, *p* < 0.0001, respectively for GA-02 treatment from low to high doses; *n* = 6).

On the first 3 days in the high-dose group, the daily food intakes were significantly reduced, (Supplementary Figures S7A,B). The body weight changes were similar to the food intake, indicating that reduced body weight was contributed by the reduced food intake (Supplementary Figure S7C).

Over the first week, the average daily food intake was reduced from the control group of 2.31 ± 0.18 g to 1.83 ± 0.28, 1.68 ± 0.35, and 0.85 ± 0.23 g for the low, middle and high dose-treatment groups, corresponding to a 20.8, 27.3, and 63.2% reduction, respectively (*n* = 6). Over the second week, the average daily food intake and was reduced from 2.47 ± 0.13 g (control group) to 1.55 ± 0.12, 1.35 ± 0.18, and 0.70 ± 0.16 g in low, middle and high dose-treatment groups, corresponding to a 37.2, 45.3, and 68.8% reduction (*n* = 6) (Figure 3C).

As shown by quantitative magnetic resonance, GA-02 significantly lowered the mice fat mass without changing the total lean mass (Figures 3E–J). Whereas vehicle-treated DIO mice had obvious fat accumulation in heart, liver, kidney and abdomen, GA-02 treated DIO mice, were thinner and had much less abdominal and epididymal fat (Supplementary Figure S8). Visible lipid droplets accumulated in the liver of vehicle group, but the liver turned into brighter red after GA-02 treatment; the liver weight was also significantly reduced upon high dose treatment (Figures 3I,J). Tissue staining further indicated that GA-02 of high dose significantly reduced the fat accumulation in the liver, compared to vehicle treatment (Figure 3K). The fasting blood glucose was significantly reduced and even achieved normal levels for the 20 mg/kg dose group (Supplementary Figures S9B,C; *p* < 0.001). The liver and epididymal white adipose tissue (eWAT) weights were significantly reduced upon drug treatment (Supplementary Figure S9A). The plasma levels of alanine and aspartate transaminases were also reduced in the GA-02 treatment of high dose compared to vehicle treatment, indicating improved liver function (Supplementary Figures S9C,D). Glucose tolerance tests (GTT) and insulin tolerance test (ITT) indicated that the clearance of blood glucose and the insulin resistance were both significantly improved by GA-02 treatment (Supplementary Figures S10A–D, *p* < 0.001).

### The Anti-Obesity Effect of GA-02 Is Leptin-Dependent

To explore whether the reduction of food intake and the subsequent weight loss could be due to toxicity, we conducted parallel assays in lean and old lean mice (the body weight of lean mice and old lean mice was 25 and 30 g in average, respectively). On the lean mice, high dosage of GA-02 (20 mg/kg; i.p.) did not alter the daily food intake and body weight, while mice received vehicle treatment maintained a steady increase (Supplementary Figures S11A–C). However, when GA-02 was administered to old lean mice maintained on normal chow diet (NCD), their body weights significantly decreased by 8.25 ± 1.57% in 2 weeks treatment (*n* = 6) (Supplementary Figures S11D–E). GA-02 also lowered the daily food intake of old lean mice (Supplementary Figure S11F), indicating that the body weight loss brought by GA-02 was not specifically due to high-fat-diet inappetance, but was closely related to the level of obesity. GA-02 was also administrated to lean mice that were fed with NCD or HFD for 60 days. The long-term treatment did not reduce the body weight on both GA-02 and vehicle groups, indicating that GA-02 has no long-term toxicity (Supplementary Figures S12A–E). The GA-02 treated mice fed on HFD also did not gain weight, showing that GA-02 suppressed HFD-induced obesity development (Supplementary Figure S12F).

**FIGURE 4:**
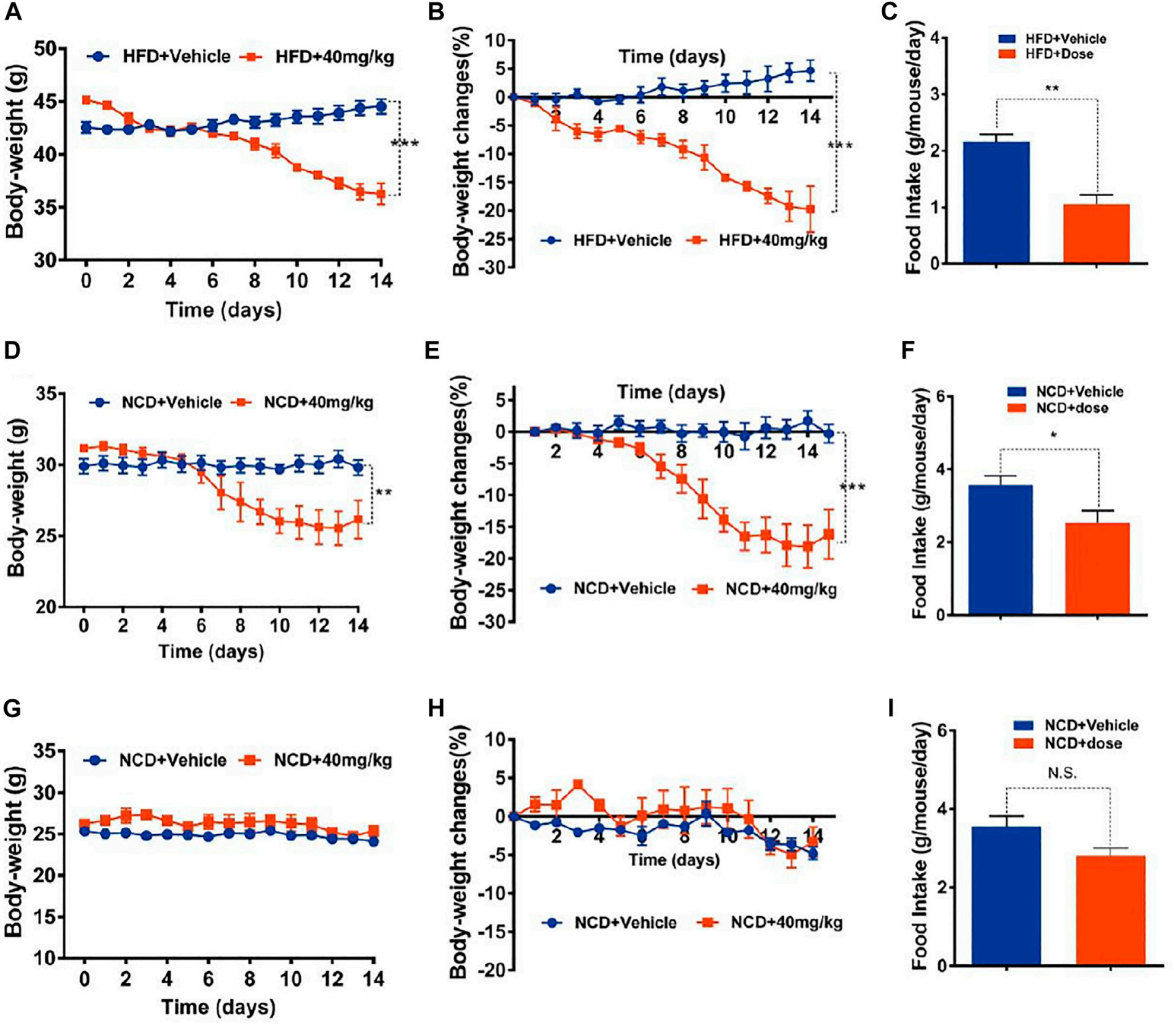
Oral treatment of mice with GA-02. **(A–C)** DIO mice, **(D–F)** old lean mice, **(G–I)** lean mice were orally administered vehicle (PBS, pH = 9) or GA-02 (40 mg/kg; o.a.) for 2 weeks. **(A)** Body weight and **(B)** percent decrease (%) in body weight of DIO mice during the treatment. **(C)** The average food intake **(G)** of DIO mice during the treatment (*n* = 5 for vehicle group and *n* = 5 for GA-02 group in **A–C**). **(D)** Body weight and **(E)** percent decrease (%) in body weight of old lean mice during GA-02 treatment; **(F)** The averages food intake of the old lean mice during the treatment. (*n* = 6 for vehicle group and *n* = 6 for GA-02 group in **D–F**). **(G)** Body weight and **(H)** percent decrease (%) in body weight of lean mice during GA-02 treatment; **(I)** The average food intakes of lean mice during the treatment. *n* = 6 for each group in **(G–I)**. Error bars are represented as mean ± SEM. (*p < 0.05, **p < 0.01, ***p < 0.001).

Oral administration (o.a.) of drugs is mostly preferred over other administration routes with advantages of safety, compliance, pain and versatility (Sastry et al., 2000). In order to eliminate a possible influence of intraperitoneal (i.p.) injection on mice food intake and body weight, the oral administration of GA-02 was also explored. The body weight of DIO-mice was reduced by 19.6 ± 1.46% in 2 weeks with GA-02 at 40 mg/kg with o.a. (Figures 4A,B). The food intake was also reduced by 50% (Figure 8C). In line with i.p. data, oral administration of GA-02 significantly lowered the body weight and average daily food intake of obese and old lean mice but had minimal effect to lean mice (Figures 4D–I), suggesting it specifically targets mice with abnormal weight.

**FIGURE 5:**
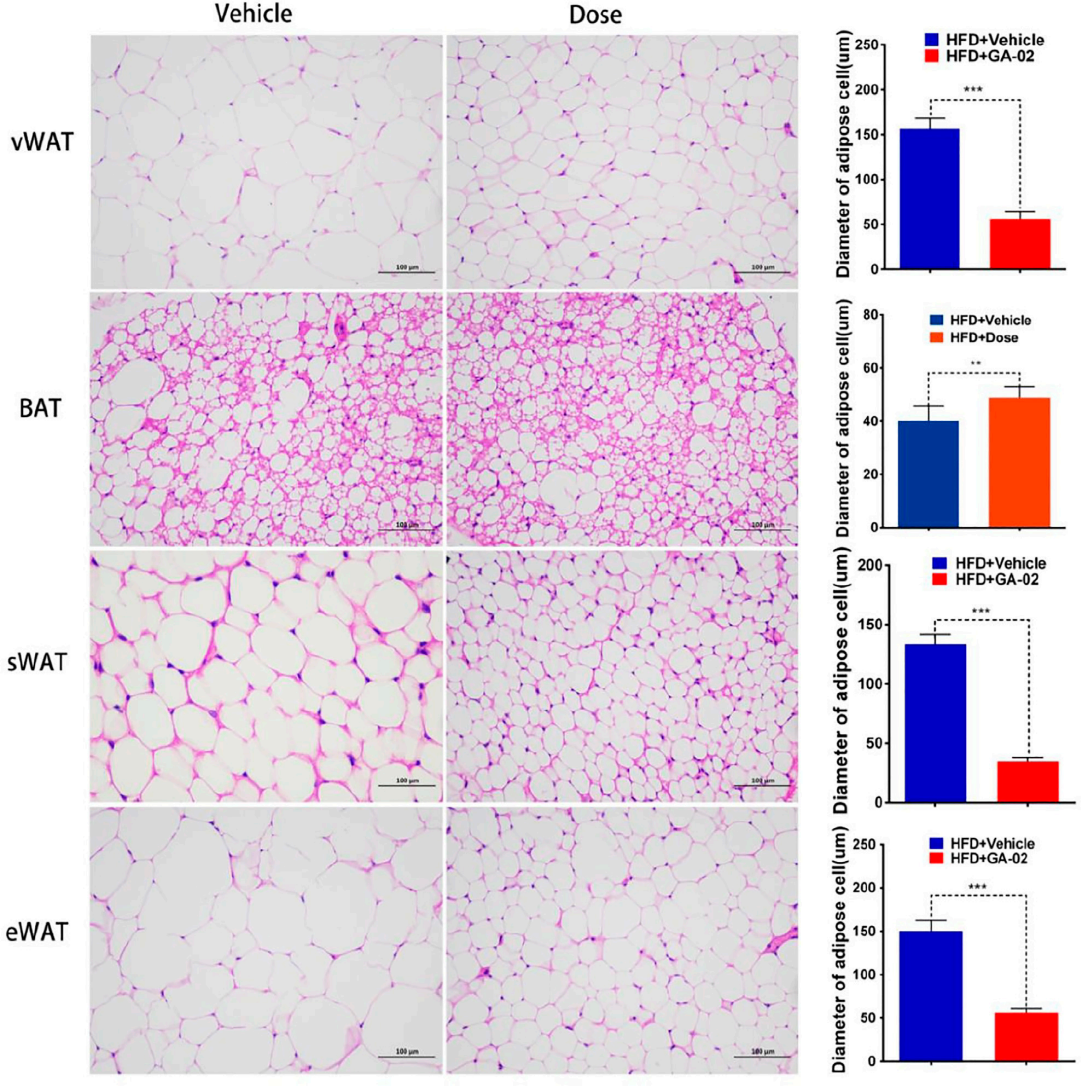
GA-02 minimally affects mice deficient in leptin signaling. Eight-weeks old male *db/db* and *ob/ob* mice were exposed to a 2-weeks treatment with vehicle or GA-02 (20 mg/kg20 mg/kg; i.p.). **(A)** The body weight changes, **(B)** the percent of body weight changes (%), **(C)** the average daily food intakes over the first week of treatment for *db/db* mice group (*n* = 5 for each group). **(D)** The body weight changes, **(E)** the percent of body weight changes (%), **(F)** the average daily food intake over the first week for *ob/ob* mice group (*n* = 5 for each group). Body weight values are expressed as mean ± SEM (*p < 0.05, **p < 0.01, ***p < 0.001).

To investigate whether the GA-02 induced weight loss is related with the leptin signaling pathway, GA-02 was administered to leptin signaling-deficient mice. In vehicle-treated leptin receptor-deficient (*db/db*) mice on NCD, the body weight increased from 40.12 ± 1.33 g to 46.81 ± 0.76 g, corresponding to a 16.73 ± 2.57% weight gain in 2 weeks (Figures 5A,B); GA-02-treated *db/db* mice gained similar weight, from 39.86 ± 1.01 g to 45.61 ± 1.27 g, corresponding to a 14.42 ± 0.80% weight gain (Figures 5A,B). In the latter group, the daily food intake was slightly, but not significantly, reduced (Figure 5C). In leptin-deficient *ob/ob* mice, over 2 weeks, there was no alteration of body weight in the vehicle group (Figure 5D); the GA-02 treatment led to a slight, but not significant, decrease in body weight and daily food intake (Figures 5D–F).

### GA-02 Activates the Leptin Signaling in DIO Mice

**FIGURE 6:**
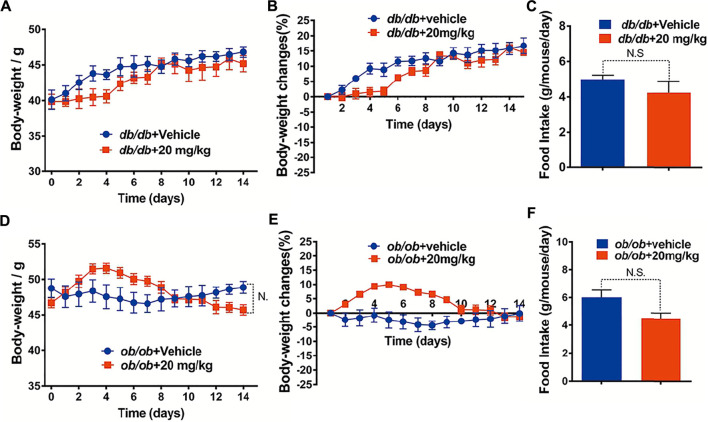
GA-02 re-activates leptin signaling. Lean mice were treated with vehicle or GA-02 (20 mg/kg; i.p.) (*n* = 8 for each group) for 2 days, and each group was divided into two subgroups, and administered either saline or leptin (1 mg/kg; *n* = 4 for each group; i.p.). **(A)** The average daily food intake and **(B)** body weight changes after 16 h of saline/leptin administration. DIO mice were treated with vehicle or GA-02 (*n* = 8 for each group; 20 mg/kg; i.p.) for 2 days, and each group was divided into two subgroups, and administered either saline or leptin (1 mg/kg; *n* = 4 for each group). **(C)** The average daily food intake and **(D)** body weight changes after 16 h of saline/leptin administration. Values are represented as mean ± SD. (*p < 0.05, **p < 0.01, ***p < 0.001). GA-02 minimally affects mice deficient in leptin signaling. Eight-weeks old male *db/db* and *ob/ob* mice were exposed to a 2-weeks treatment with vehicle or GA-02 (20 mg/kg20 mg/kg; i.p.). **(A)** The body weight changes, **(B)** the percent of body weight changes (%), **(C)** the average daily food intakes over the first week of treatment for *db/db* mice group (*n* = 5 for each group). **(D)** The body weight changes, **(E)** the percent of body weight changes (%), **(F)** the average daily food intake over the first week for *ob/ob* mice group (*n* = 5 for each group). Body weight values are expressed as mean ± SEM (*p < 0.05, **p < 0.01, ***p < 0.001).

**FIGURE 7:**
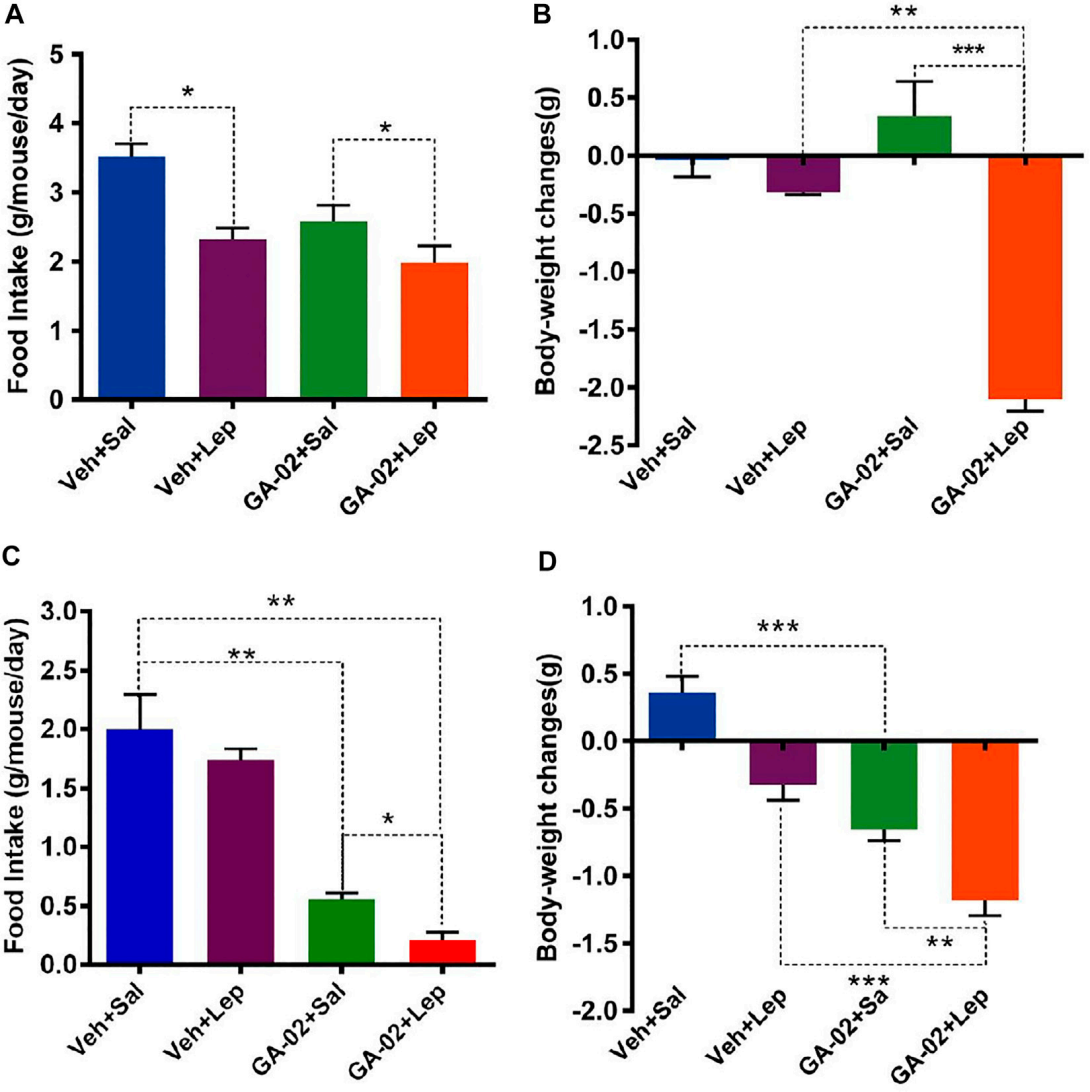
GA-02 activates leptin signaling in the hypothalamus of DIO mice. Representative images **(A)** and quantitative cell number **(B)** of pSTAT3 (Tyr705)-positive cells from the ventromedial hypothalamus (VMH); Mice with DIO received vehicle or GA-02 (20 mg/kg, 15 h; i.p.) and then were injected with saline or leptin (1 mg/kg, 30 min; i.p.). **(C)** NE concentration in epididymal and subcutaneous fat of DIO mice that were injected with vehicle or GA-02 (20 mg/kg) for 3 days and subsequently given saline or leptin (1 mg/kg). Representative images of immunostaining **(D)** and relative fluorescence intensity **(E)** of p-HSL (red) in paraffin sections of epididymal fat of DIO mice that were administered vehicle or GA-02 (20 mg/kg) for 3 days and subsequently given saline or leptin (1 mg/kg). Scale bar, 100 µm. Values are represented as mean ± SD. (*p < 0.05, **p < 0.01, ***p < 0.001).

To examine the sensitivity to exogenous leptin under GA-02 treatment, lean mice were administered leptin and/or GA-02 and maintained on NCD over 14 days. Exogenous leptin led to a 24% decrease of food intake whereas GA-02 plus leptin resulted in a 26% decrease (Figure 6A). Leptin alone resulted in a slight and significant reduction of body weight (*p* < 0.05), but not GA-02 (Figure 6B). Consistent with previous data (Liu et al., 2015), the daily food intake of leptin-treated DIO mice did not show any significant changes (*p* > 0.05); by contrast, the GA-02 alone treatment reduced their daily food intake from 2.01 ± 0.50 g to 0.56 ± 0.06 g (a 72.1% reduction, Figure 6C). Leptin plus GA-02 decreased the food intake even more, to a 89.1% reduction (Figure 6C), the body weight lost amounting to 1.18 ± 0.04 g per day (Figure 7D). This suggests that the anorectic and weight-reducing effects of GA-02 are mediated by leptin-signaling.

Leptin, through binding to its receptor in hypothalamus, promotes STAT3 phosphorylation (pSTAT3). (Allison and Myers, 2014). The STAT3 (Tyr705) phosphorylation levels in hypothalamus were analyzed by immunofluorescence microscopy. Obese mice treated with GA-02 or leptin alone showed notably increased p-STAT3 (Tyr705) levels, whereas leptin administed after GA-02 induced p-STAT3 (Tyr705) to an even higher degree (Figure 7A). Norepinephrine (NE) promotes lipolysis and fat mass reduction through sympathetic innervation of adipose tissue (Pirzgalska et al., 2017). The NE levels in epididymal fat dissected from leptin treated DIO mice were significantly higher than the control (*p* < 0.05). However, GA-02 led to considerably higher levels of NE than the control or leptin treatment (*p* < 0.001). Further administration of leptin after GA-02 led to a boosting of NE (Figure 7B). Similar with stimulation of sympathetic innervation in epididymal fat, GA-02 alone and GA-02 plus leptin treatment led to an increase in NE level in the subcutaneous fat tissue (Figure 7C). Hormone-sensitive lipase (HSL), responsible for catalyzing the triacylglycerol hydrolysis, is known to be highly phosphorylated in response to leptin activation in the adipose tissue. (Zeng et al., 2015). In our model, GA-02 or leptin treatment led to significant higher levels of basal p-HSL compared to vehicle groups, but leptin plus GA-02 led to a significant enhanced p-HSL level in epididymal white adipose tissue (e-WAT) (Figure 7C).

**FIGURE 8:**
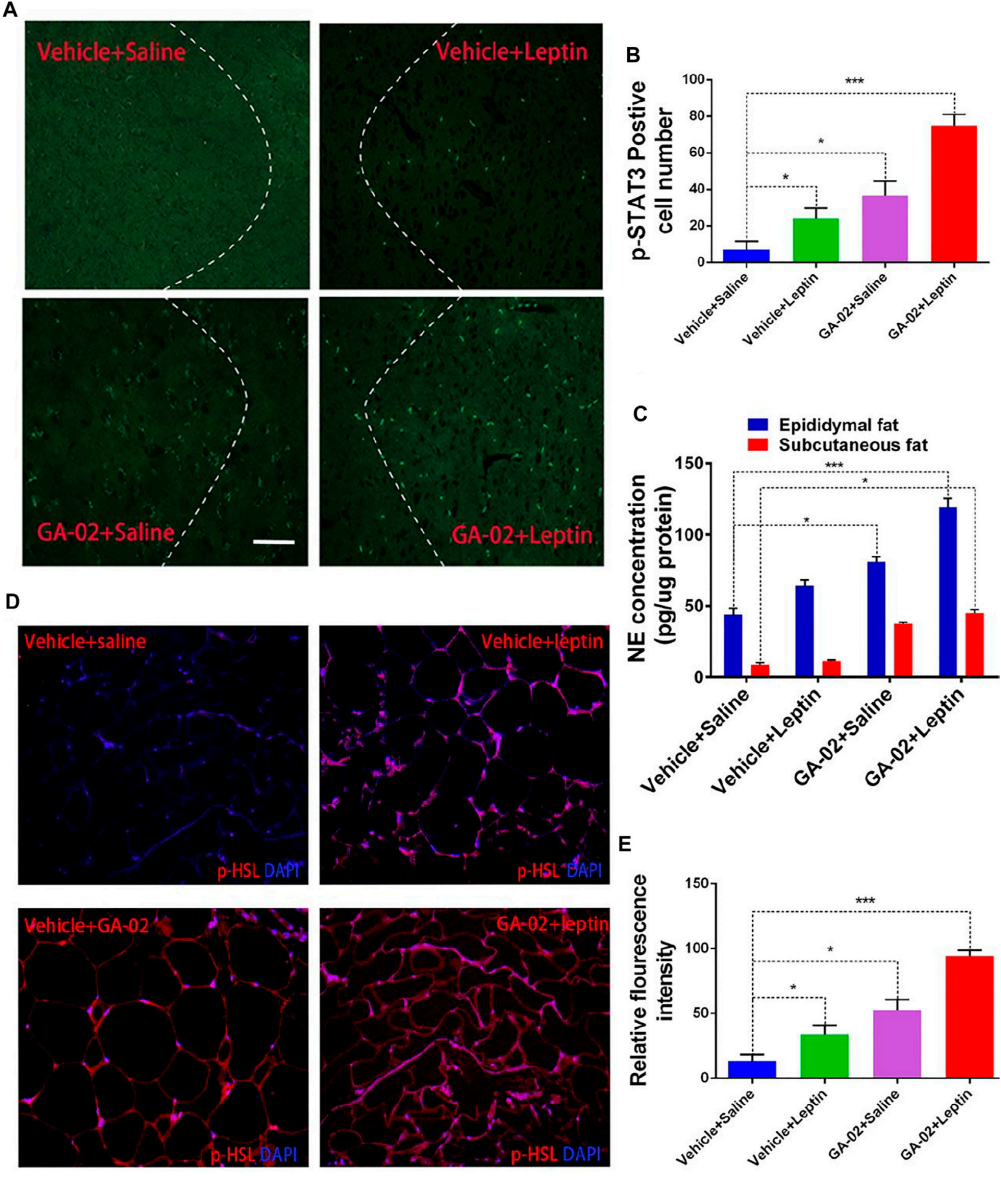
Representative images of vWAT, BAT, sWAT and eWAT adipocytes. The adipocytes volume and diameter changes in DIO mice treated with vehicle and GA-02 (20 mg/kg; i.p.) for 14 days (mean ± SEM). Scale bar, 100 µm. (*p < 0.05, **p < 0.01, ***p < 0.001).

HSL-induced WAT cell lipolysis, investigated by H&E staining, showed that the administration of GA-02 to DIO mice led to a significant reduction of the volume and diameter of visceral-WAT (v-WAT), subcutaneous-WAT (s-WAT) and e-WAT adipocytes, a major form of energy storage; however, the diameter of BAT adipocyte in GA-02 group is significantly bigger than that of vehicle treatment, indicating more thermogenesis from GA-02 treatment (Figure 8).

**FIGURE 9:**
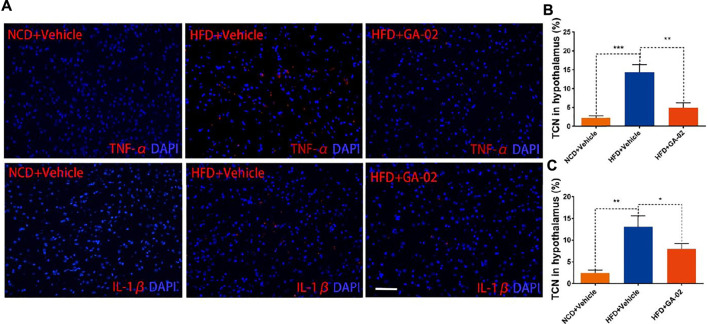
Representative images of immunostaining for TNF-α and IL-1β in the hypothalamus. The immunostaining of for DAPI and TNF-α [**(A)**, upper panel] and IL-1β [**(B)**, lower panel] was conducted from hypothalamus of NCD fed mice or DIO mice that were treated with vehicle and GA-02 (20 mg/kg; i.p.) for 7 days. The percentage of positive TNF-α **(B)** and IL-1β **(C)** from total cell number (TCN) was quantified. Scale bar, 100 µm. (*p < 0.05, **p < 0.01, ***p < 0.001).

HFD-associated hypothalamic inflammation might play a pivotal role in energy imbalance and increasing inflammatory signaling in the hypothalamus might contribute to leptin resistance (Posey et al., 2009). We analyzed TNF-α and IL-1β levels in hypothalamus though immunostaining. In the, DIO mice treated with vehicle alone showed higher level of TNF-α and IL-1β positive cells compared to the NCD mice treated with vehicle; in the hypothalamus of DIO mice, however, GA-02 treatment showed significantly lower level of TNF-α and IL-1β positive cells (Figure 9). In hematological parameters, feeding mice with HFD significantly increased the quantity of monocytes and lymphocytes compared to NCD in plasma; the GA-02 treatment significantly reduced the numbers of these blood cells subgroup (Supplementary Figure S13). These results indicate that the GA-02 treatment significantly ameliorates the HFD-induced inflammation, both in hypothalamus and in periphery. Taken together, these results indicate that, in DIO mice, there could be a crucial link between hypothalamic inflammatory suppression and leptin signaling activation.

### The Safety Assessment of GA-02

To evaluate the cytotoxicity of GA-02 *in vitro*, cultures of rat cardiomyoblasts H9C2, human foetal hepatocytes L02 and human embryonic kidney cells HEK293T were treated with GA-02; the IC_50s_, measured by a MTT assay (Supplementary Table S5), indicate a low cytotoxicity of GA-02 in all tested cell lines. An *in vivo* acute toxicity test, conducted in normal C57BL/6J mice orally administered with GA-02, resulted in a LD_50_ of 939.8 mg/kg. H&E staining showed that DIO mice treated with GA-02 for 14 days at the dose of 20 mg/kg/day, exhibited no visible lesion in heart, liver, spleen, lung, kidney and brain (Supplementary Figure S14A). These mice blood biochemical parameters, examined after 14 days treatment at the dose of 20 mg/kg/day, indicated that glucose, cholesterol, triglyceride and total proteins were all significantly improved to normal range (Supplementary Figures S14B–G).

To assess the therapeutic potential of GA-02, its pharmacokinetic profile was examined *in vivo* in SD male rats via both i.p. and o.a., at a single dose (20 mg/kg; Supplementary Figure S15). The area under the curve for i.p. was 42.05 ng h.ml^−1^, with a Cmax of 12.08 ng ml^−1^ and a half-life of 2.70 h. The o.a. bioavailability of GA-02 was measured at 41%.

## Discussion

Obesity has emerged as a worldwide major health burden for many decades but effective treatment is still in high demand. Recently, celastrol, a triterpenoid natural product from a well-known traditional Chinese medicine (TCM), has been proposed to be an excellent agent against obesity (Liu et al., 2015). Although great advancements have been made, its strong toxicity may hamper a further translation into clinics (Cascão et al., 2017). Meanwhile, it caught our attention that celastrol has limited natural abundance in the original plant (Supplementary Table S1). From a tritepenoids library we generated, it can be seen that their contents in their respective herbal drugs ranged from as low as 0.02 ppm (dysolenticin A in the leaves and twigs of *Dysoxylum lenticellatum*) to as high as 10.3% (glycyrrhizin, in the root of *G. uralensis*; Supplementary Table S1). Although it is not fully understood why plants store secondary metabolites in so huge variations, a plant resource rich in a given compound will certainly make its exploitation more economic, convenient and environmentally friendly.

Structural similarity is one of key strategies for drug discovery (Dar et al., 2018; Trosset and Cavé, 2019). Diverse structures and biological activities beign known for naturally occurring triterpenoids, we hypothesized that some compounds with structures similar to celastrol could present similar anti-inflammatory and anti-obesity activities, while providing key drugging profiles in safety and availability. By modeling structural similarities, all these triterpenoids could be compared relatively to celastrol in the form of similarity scores. From a correlation analysis between the similarity scores and natural bio-contents of these triterpenoids, glycyrrhizin and glycyrrhetinic acid (GA) emerge as the two compounds with the highest yield and moderate structural similarity to celastrol (top 5%). GA is the aglycone of glycyrrhizin, which can be easily converted into GA, meaning the GA yield from the plant will be higher than its natural 15%, through an easy conversion. The extremely high natural abundance gives GA a major advantage of cost effectiveness when compared to celastrol. Glycyrrhizin and GA are major active ingredients of licorice with diverse pharmacological activities, such as anti-inflammation, anti-tumor and anti-hepatotoxicity (Pastorino et al., 2018). As a most important TCM drug, licorice takes a part in 80% of TCM formulas, meaning a favorable safety profile, recorded from thousands of years of practice. Licorice is widely cultivated in China and has a rich distribution in most of West Asian and European countries, which enables sufficient supply and low cost of GA (Hayashi et al., 2003). Based on this scaffold for further development of drug mimics then seems highly favorable.

Basing on such a scaffold benefits from an abundant resource and low toxicity, but, indeed, chemical modifications may result in alterations of its efficacy and safety profiles. Typically, there are many ways to optimize the activity of a chemical lead, including functional groups modification, structure-activity relationship-directed structure optimization and active fragment-oriented molecular re-designing (Xiao et al., 2016). In this study, we noticed key groups in the ring A of structurally close molecules, all possessing anti-inflammatory efficacy; meanwhile, obesity was also reported to be tightly connected to inflammation (Hotamisligil, 2006; Lumeng et al., 2007). Then we adopted a quantitative anti-inflammatory evaluation method, that we previously developed (Hu et al., 2012), to test a series of modified triterpenoids so to identify a best drug candidate, GA-02 (Figures 1, 2).

GA-02 treatment of HFD-induced obese mice led to a drastic decrease of daily food intake and body weight loss (Figures 3, 4). The GA-02 did not exhibited apparent dose-effect, because the weight losing between dosage of 4 and 8 mg/kg was very close (Figure 3). Also, all dosage between 0.01, 0.1, 1 and 2 mg/kg did not significantly reduce the body weight of the mice (data not shown). Our hypothesis is that it might need a threshold of high concentration, for example, more than one target, for the best efficacy of weight loss. NMR and morphology analysis indicate that the body weight reduction was mainly due to GA-02-induced anorexia and subsequent catabolism of adipose tissue (Figure 3). On the other hand, GA-02 administered to mice with low plasma levels of leptin (i.e., lean mice) or to leptin signaling-deficient mice minimally altered their body weight and daily food intake (Figure 5; Supplementary Figure S12). Further evidence for a leptin re-sensitizing effect of GA-02 were the physiological and biochemical responses of mice treatment with exogenous leptin and GA-02, i.e., a significant increase of leptin-dependent molecular markers, hypothalamus STAT3 (Tyr705) phosphorylation and NE-mediated HSL phosphorylation in adipocytes, leading to white adipose tissue lypolysis (Figures 6, 7). It is important to note that the determination of the number of pSTAT3 positive cells was performed in the ventromedial hypothalamus, a region important for the anorexigenic action of leptin (Ahima and Flier, 2000).

Hypothalamic inflammatory activation probably plays a pivotal role, contributing to leptin resistance and weight gain. We observed obvious hypothalamic inflammation in HFD-induced obese mice, that was significantly inhibited by GA-02 treatment (Figure 9). Thus, the appetite suppression and weight loss induced by celastrol or GA-02 indicate a crucial link between leptin signaling re-sensibilization and hypothalamic inflammation suppression, pointing to a likely possibility for obesity control. So far, however, the molecular mechanisms behind these effects still need further investigation.

As discussed earlier, total synthesis of structurally sophisticated natural products often require many steps of cascade reactions. To pinpoint differences between naturally evolved and human-engineered compounds, a comparison model of celastrol chemical synthesis and GA biosynthesis is herein proposed, presuming a constant catalytic efficiency in each reaction step (Supplementary Figure S17A). The final yield was proposed to be an exponentiation with catalytic efficiency as the base and catalytic steps as the index. It can be seen that, whether from actual calculation (average 84% efficiency per step) or from an ideally presumed 90% percent yield, the 28-steps synthesis of celastrol result in an extremely low yield. Also, 27 of these steps require harsh synthesis conditions, environment unfriendly; the synthesis yield remains as low as the extraction yield from roots of *T. wilfordii*. By contrast, *in planta*, GA requires only 14 steps to be generated from primary metabolites, which results in a higher yield (16.2% of GA and its downstream product glycyrrhizin). We also calculated that, if the *in planta* synthesis of GA was continuously extended to obtain celastrol, the final yield would be pretty close (Supplementary Figure S16A). Thus our mathematical model indicates that the advantages of biosynthesis reside in a concisely short and efficient sequence of enzymatic reactions, that have been optimized through millions of years of biogenic enzymes evolution. Its efficient obtaining through hemisynthesis makes the possible costs of GA-02 much lower compared to celastrol; the cost of celastrol synthesis would be 20 times higher than its extraction from plant and 200 times higher than GA-02 (Supplementary Figures 16A–C).

Although GA-02 showed similar trend to celastrol in computational binding to receptor Nur77, the binding affinity of GA-02 measured *in vitro* by SPR was much lower than that of celastrol (Figure 2C). Except difference of experimental time span (2 vs 3 weeks), this was consistent with the actual i.p.administration that the dose of GA-02 in our study was much higher than celastrol (100 μg/kg) in a previous study (Figure 3) (Liu et al., 2015). But for o.a. administration, the dosage become close (40 mg/kg in our study for GA-02 or 10 mg/kg for celastrol in the previous study (Liu et al., 2015). It clearly indicated that other factors might have played a role for the weight loss. For example, the strong toxicity of celastrol might have also contributed since the dosage of o.a. administration was 100 times higher than that of i.p. (Liu et al., 2015). Nevertheless, the dosage of GA-02 either with i.p. or o.a. is within an acceptable range for further clinical translation.

All in all, hemisynthesis procedures provide a sustainable and practical strategy of bulky production of drug mimics. Although total synthesis of natural products is continuously making exciting progress (Baran, 2018), competition with naturally evolved process remains a challenge in overall efficiency, yield, sustainability and economy for structure-sophisticated compounds. Indeed, plants remain an excellent chemical factory for compounds of interest.

## Conclusion

In the present work, combining high natural abundance and structural similarity screening criteria allowed to identify mimics of celastrol and to pinpoint the possible relevance of GA. Although the interest of an extremely high content of GA in *Glycyrrhiza* spp. has been extensively investigated (You et al., 2013), an effective bridge with efficacy-directed structure modification has not been established before. As a proof of this concept, we showed that limited modifications of GA result in GA-02, a potent anti-obesity agent on DIO mice, presenting a similar activity profile as the well-known lead compound, celastrol. This strategy of modifying a readily-obtainable natural scaffold contributes a unique and efficient path to improve the profile of a natural product, a critical parameter for clinical translation. In addition, the scheme we established offers a green, economic and environment-friendly approach to compounds with complex structures.

## Data Availability

The original contributions presented in the study are included in the article/Supplementary Material, further inquiries can be directed to the corresponding author.

## Abbreviations

ALT, alanine aminotransferase; AST, aspartate transaminase; DIO, diet-induced obese; eWAT, epididymal white adipose tissue; GA, glycyrrhetinic acid; GTT, glucose tolerance; H&E, hematoxylin/eosin; HFD, high-fat diet; HMEC-1, human microvascular endothelial cells; HSL, Hormone-sensitive lipase; i.p., intraperitoneal; ICAM-1, intercellular adhesion molecule-1; ITT, insulin tolerance; LBD, ligand binding Domain; NCD, normal chow diet; NE, Norepinephrine; o.a, oral administration; PBS, phosphate buffer saline; pSTAT3, STAT3 phosphorylation; SPR, surface plasmon resonance; s-WAT, subcutaneous-WAT; TC, total cholesterol; TCM, traditional Chinese medicine; TG, triglyceride; TP, total protein; TNF, tumor necrosis factor; v-WAT, visceral-WAT.
