# Supplementary material for: Creation of an Anti-Inflammatory, Leptin-Dependent Anti-Obesity Celastrol Mimic with Better Druggability
Source: Front Pharmacol. 2021 Aug 30;12:705252. doi: 10.3389/fphar.2021.705252 (PMC8435713; doi:10.3389/fphar.2021.705252)
Supplement: Supplementary file 1 [file DataSheet1.docx]

Content

[Supplementary Text 3](#_Toc16441)

[Leptin treatment and immunofluorescence staining 5](#_Toc19344)

[Supplementary Tables 7](#_Toc9192)

[Table S1 The library of the collected natural triterpenoids (Page 25-196) 7](#_Toc25851)

[Table S2 Structural similarity scores relative to celastrol (Page 197-213) 7](#_Toc22498)

[Table S3 Primers used for RT-PCR 7](#_Toc24442)

[Table S4 GA-02 structural assignments 9](#_Toc24505)

[Table S4 GA-02 structural assignments 9](#_Toc12821)

[Table S5 Antiproliferative Activity of GA-02 and Celastrol 10](#_Toc14635)

[Supplementary Figures 11](#_Toc794)

[Supplementary Fig 1 Commercial available triterpenoids and anti-inflammatory activity assay. (a) 11](#_Toc15021)

[Supplementary Fig 2 Top 8 triterpenoids of highest structural similarity scores 12](#_Toc31491)

[to celastrol 12](#_Toc20290)

[Supplementary Fig 3 Hemisynthesis of GA-02 (a) The structure of a few potent anti-inflammatory natural products bearing a conjugated carbonyl and a nearby hydroxyl group on ring A; (b) Structure modification from GA to GA-02，and related conditions for each reaction . 12](#_Toc29554)

[Supplementary Fig 4a](#_Toc16902) ^[1](#_Toc16902)^[H NMR (600MHz) spectrum of GA-01 in DMSO-d6 13](#_Toc16902)

[Supplementary Fig 4b](#_Toc2831) ^[13](#_Toc2831)^[C NMR (150MHz) spectrum of GA-01 in DMSO-d6 14](#_Toc2831)

[Supplementary Fig 4c HRESIMS spectrum of of GA-01(Positive and negative ion modes). 15](#_Toc7958)

[Supplementary Fig 5a](#_Toc11595) ^[1](#_Toc11595)^[H NMR (600MHz) spectrum of GA-02 in DMSO-d6 16](#_Toc11595)

[Supplementary Fig 5b DEP135 NMR (150MHz) spectrum of GA-02 in DMSO-d6 16](#_Toc7358)

[Supplementary Fig 5c 13C NMR (150MHz) spectrum of GA-02 in DMSO-d6 17](#_Toc21850)

[Supplementary Fig 5d HRESIMS spectrums of GA-02(Positive and negative ion modes). 18](#_Toc22295)

[Supplementary Fig 5e COSY (600MHz) spectrum of GA-02 in DMSO-d6 19](#_Toc19938)

[Supplementary Fig 5f HMBC (600MHz) spectrum of GA-02 in DMSO-d6 20](#_Toc14702)

[Supplementary Fig 5g HSQC (600MHz) spectrum of GA-02 in DMSO-d6 21](#_Toc31532)

[Supplementary Fig 6. Celastrol binding to receptor Nur77. (a) The binding mode of celastrol with Nur77 generated by PoseView. Nur77 (PDB code 4JGV) is shown as ribbon diagram, and celastrol as green ball-and-stick model. (b) Celastrol binding to Nur77 was illustrated by SPR assay of purified Nur77-LBD with celastrol. 22](#_Toc3132)

[Supplementary Fig 7 Food intake and body weight changes during the first three days of high dose GA-02 treatment. 23](#_Toc27615)

[Supplementary Fig 8 Representative image of visceral tissue from DIO mice 24](#_Toc4975)

[Supplementary Fig 9 Tissue and blood parameters of mice. 25](#_Toc18507)

[Supplementary Fig 10 GA-02 Improves Glucose Homeostasis in DIO Mice 26](#_Toc31878)

[Supplementary Fig 11 The effects of GA-02 in young-lean and old-lean mice 27](#_Toc21311)

[Supplementary Fig 12 Food Intake Calculation as Calories and Long-Term GA-02 Treatment. 28](#_Toc14085)

[Supplemental Fig 13 Plasma hematology assay 29](#_Toc19797)

[Supplementary Fig 14 No detectable toxicity in mice upon GA-02 treatment. 30](#_Toc6449)

[Supplementary Fig 15 Plasma concentration-time profiles of GA-02. 31](#_Toc20006)

[Supplementary Fig 16. A comparison of different production strategies for celastrol and GA-02. (a) A computational model of the catalytic efficiency of chemical synthesis and biosynthesis. 32](#_Toc22233)

[Table S1. The libirary of plant triterpenoids 33](#_Toc12927)

[Table S2. 3D structural similarity score relative to celastrol 205](#_Toc14590)

Supplementary Text

**Experimental producers for GA-02 synthesis and structural verification**

All ^1^H, ^13^C, ^1^H-^1^H COSY, HSQC, and HMBC NMR spectra were collected on a Bruker Avance 600 at 600 MHz for ^1^H and 150 MHz for ^13^C nuclei using TMS as an internal standard. The highly resolution LC-MS analysis was performed on an Agilent 6530 spectrometer with a Poroshell 120 (EC-C18 column, 4.5×50 mm, 2.7 μm, Agilent Techonologies). MS spectra were recorded on a Mariner mass spectrometer (ESI) and highly resolution mass spectrometry (HRMS) spectra on a Shimadzu Technologies LC/MSD TOF instrument. Analytical and preparative TLC was performed on silica gel (200−300 mesh) GF/UV 254 plates, and the chromatograms were visualized under UV light at 254 and 365 nm. All solvents were reagent grade and. The purity of all tested compounds was characterized by HPLC analysis. Individual compounds with a purity of >95% were used for subsequent experiments.

**GA-02 synthesis**

**Preparation of Methyl 3,12-dioxoolean-9(11)-en-28-oate (GA-01).**

Preparation of Jones’ Reagent: 26.7 g of CrO_3_ was dissolved in 50 ml cold water and 23 mL of conc H_2_SO_4_. The mixture was cooled to 0 °C, and 27 mL of cold water was added dropwise to obtain a maroon colored solution.

GA (23.5 g, 50.0 mmol) was dissolved in acetone and dichloromethane (v/v=1:1) (500 mL). The reaction mixture was cooled to 0 °C, and the Jones’ reagent was added dropwise to the reaction until a color change from green to brown color. The mixture was allowed to stir for 30 min at rt. The solvent was removed in vacuo and extracted with dichloromethane (3 ×500 mL) and water (2×300 mL). The combined organic layers were washed with water, brine, dried over anhydrous sodium sulfate, and filtered. The solvent was removed in vacuo to give GA-01 (21.1 g, 90%) which was crystalized in methanol/ dichloromethane as a white solid product; mp = 103−105 °C; (^1^H and ^13^C NMR data, see Supplemental Fig 3); HRMS affording the [M+H]^+^ ion at 469.3m/z, [M-H]^-^ ion at 467.3m/z (calculated [M+H]^+^ for C_30_H_44_O_4_ at 469.3).

**2-Hydroxy-3-oxooleana-18β, 20β-olean-12-en-29-oic acid (GA-02)**

To a solution of GA-01 (23.4 g, 50 mmol) in tert-butanol (300 mL) was added potassium tert-butoxide (28.02 g, 250 mmol) at 40−45 °C, and the mixture was stirred for 3 h. After the mixture was cooled, 5% aqueous HCl was added to adjust to pH = 4. Evaporation of the solvent under vacuum gave a white residue which was filtered and washed with water. The crude material was subjected to crystalized in methanol/ dichloromethane =9:1 to afford GA-02, which was recrystallized once again as a white crystal (13.2g, 55%), mp 297−300 °C; ^1^H and ^13^C NMR data, see Supplemental Fig 4a-f; HRESIMS affording the [M+H] ^+^ ion at 483.3 m/z (calcd [M+H]^-^ for C_30_H_42_O_5_ at 483.3), [M-H]^-^ ion at 481.3m/z.

## Leptin treatment and immunofluorescence staining

The imunofluorescence staining of tissues was performed. DIO mice were acclimated with 25 μL of vehicle intraperitoneally for 3 d. Then mice were injected with 25 μL of either vehicle or GA-02 for 3 d. 14 hr later, each mouse was injected once more with vehicle or GA-02. Each group was divided into two subgroups, and after 6 h of the 4th injection, each subgroup was administered with 100μL saline or leptin (500μg/kg in 100 μL saline). After 1 h of leptin or saline administration, brains were fixed via cardiac perfusion with cold 4% PFA through the heart after the blood was flushed out with saline. The brain, visceral and epididymal fat were collected and fixed respectively in 4% PFA and the adipose tissue fixative.

Following additional 24h fixation, collected tissue were incubated in 20% sucrose for 2 days, sections were collected and washed in PBS, incubated with 0.3% H_2_O_2_–0.1% NaOH in PBS, 0.3% glycine, and then 0.03% SDS. Following incubation with blocking buffer (3% goat serum), the sections were incubated with p-STAT3Tyr705 antibody (1:3,000 dilution in blocking buffer; Cat. 9145, CTS) for 48 h at 4 °C. After washing three times with PBST, the sections were incubated with FITC-conjugated goat anti-rabbit IgG. After washes, the sections were subjected to image processing. The imunofluorescence staining of p-HSL in epididymal fat and TNF-α/IL-1β in the hypothalamus were according the same method as p-STAT3 in hypothalamus. When necessary, the fluorescence was quantified by software ImageJ (Rasband, W.S., ImageJ, U. S. National Institutes of Health, Bethesda, Maryland, USA, https://imagej.nih.gov/ij/, 1997-2018).

**Acute toxicity test**

To evaluate the toxicity of GA-02 in vivo, an up-and-down producer (UDP) study was performance in accordance with the method provided by Organization for Economic Cooperation and Development (OECD) Guideline No.425 (OECD, 2008). C57BL/6J mice (8 weeks old) were used in this study, and food was withdrawn overnight before treatment. Mice were intragastric administrated with GA-02 at concentration of 175, 550, 1000, 2000 mg/kg. Mice activity and lethality were observed in 24h and 14 days after dosing. LD_50_ value was calculated using AOT425.

**Pharmacokinetic study**

Three SD male rats were intravenously treated with GA-02 i.v. (20mg/kg) or oral (20 mg/kg). Blood samples were taken from suborbital vein at 0, 15, 30, 45, 60 min, and 2 h, 3 h, 5 h, 7 h, 9 h, 12 h, 24 h. 100μL of plasma was quickly transferred to the Eppendorf tubes containing acetonitrile (100 μL) and then immediately centrifuged at 12000 rpm for 10 min. The obtained supernatant was moved to another blank Eppendorf tube and was evaporated at 40 °C under N_2_. The dried residue was resolved with 190 μL of mobile phase and 10 μL of 4 μg mL^−1^ 18β-glycyrrhetinic acid internal standard, the mixture mixed well vortexed for 1 min and centrifuged at 12,000 g for 10 min. Finally, the supernatant was filtered through a 0.22-μm hydrophobic membrane for injection into the LC-MS/MS. The mobile phase consisted of acetonitrile and 0.5% formic acid (80:20, v/v). The total running time was 4.5 min at a flow rate of 0.4 mL/min at 30 °C. Analytes were detected using an electrospray ionization source (ESI) interface in positive ion mode. Multiple reaction monitoring was applied to monitor the transitions at m/z 483.3→129.4 for GA-02 (collision energy: 59 eV). Dwell time was 550 ms. Other instrumental parameters were as follows: gas temperature, 350 °C; gas flow, 8 mL per min; nebulizer pressure, 30 psi; and capillary energy, 4000 V.

Supplementary Tables

Table S1 The library of the collected natural triterpenoids (Page 25-196).

Table S2 Structural similarity scores relative to celastrol (Page 197-213).

Table S3 Primers used for RT-PCR

| Gene | Sequences |
| --- | --- |
| IL-1β | F; 5’-ATGATGGCTTATTACAGTGGCAA-3’  R; 5’-GTCGGAGATTCGTAGCTGGA-3’ |
| IL-6 | F：5’-ATCCAGTTGCCTTCTTGGGACTGA-3’  R：5’-TAAGCCTCCGACTTGTGAAGTGGT-3’ |
| TNF-α | F：5’-GAGGCCAAGCCCTGGTATG-3’  R：5’-CGGGCCGATTGATCTCAGC-3’ |
| MCP-1 | F：5’-CAGCCAGATGCAATCAATGCC-3’  R：5’-TGGAATCCTGAACCCACTTCT-3’ |
| GAPDH | F：5’-ACCACAGTCCATGCCATCAC-3’  R：5’-TCCACCACCCTGTTGCTGTA -3’ |

Table S4 GA-02 structural assignments


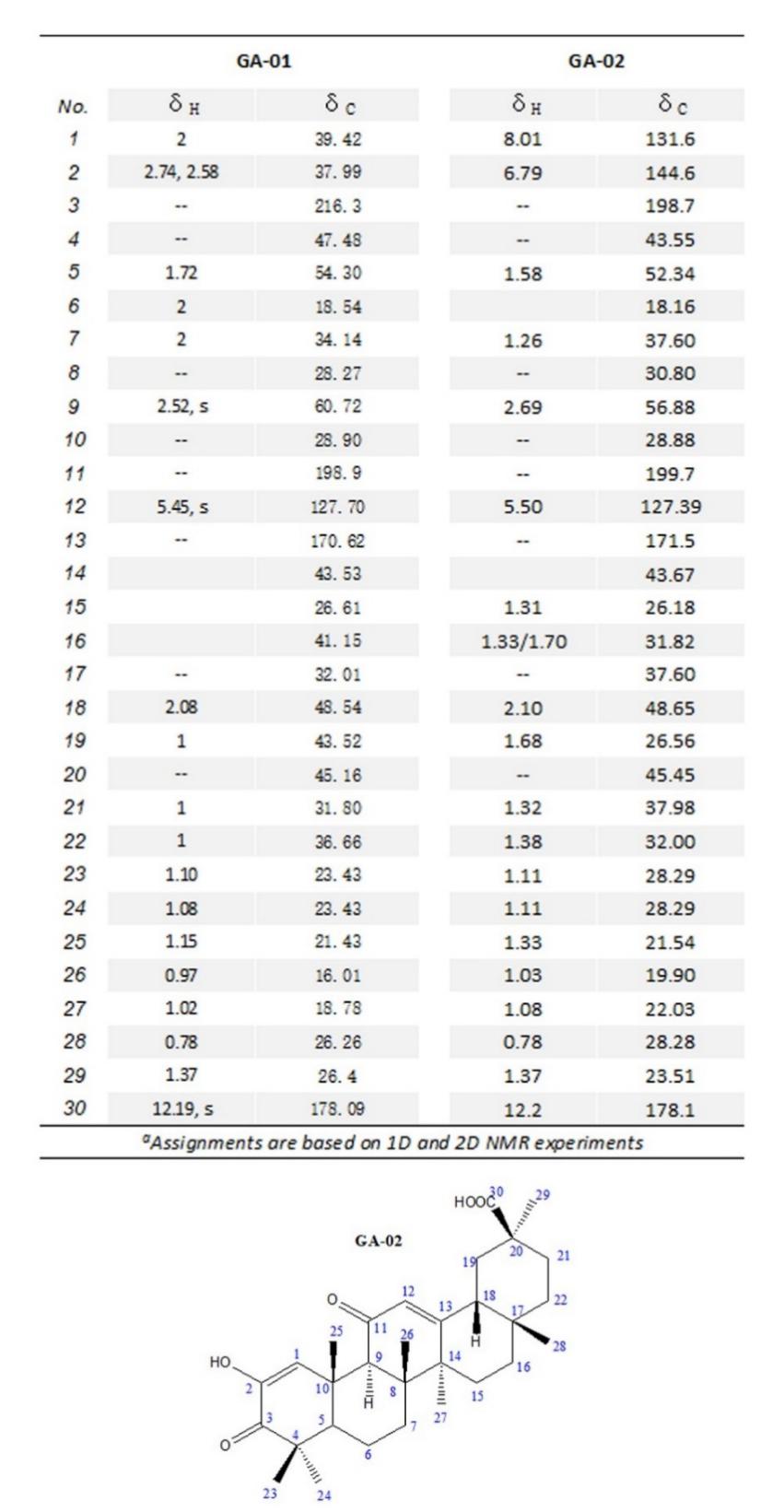

Table S4 GA-02 structural assignments

Table S5 Antiproliferative Activity of GA-02 and Celastrol

| Compound | IC_50_ (uM) | | |
| --- | --- | --- | --- |
|  | 293T | H9C2 | L02 |
| Celastrol | 14.1 | 12.2 | 32.4 |
| GA-02 | 62.5 | 122.5 | 275.9 |

Supplementary Figures


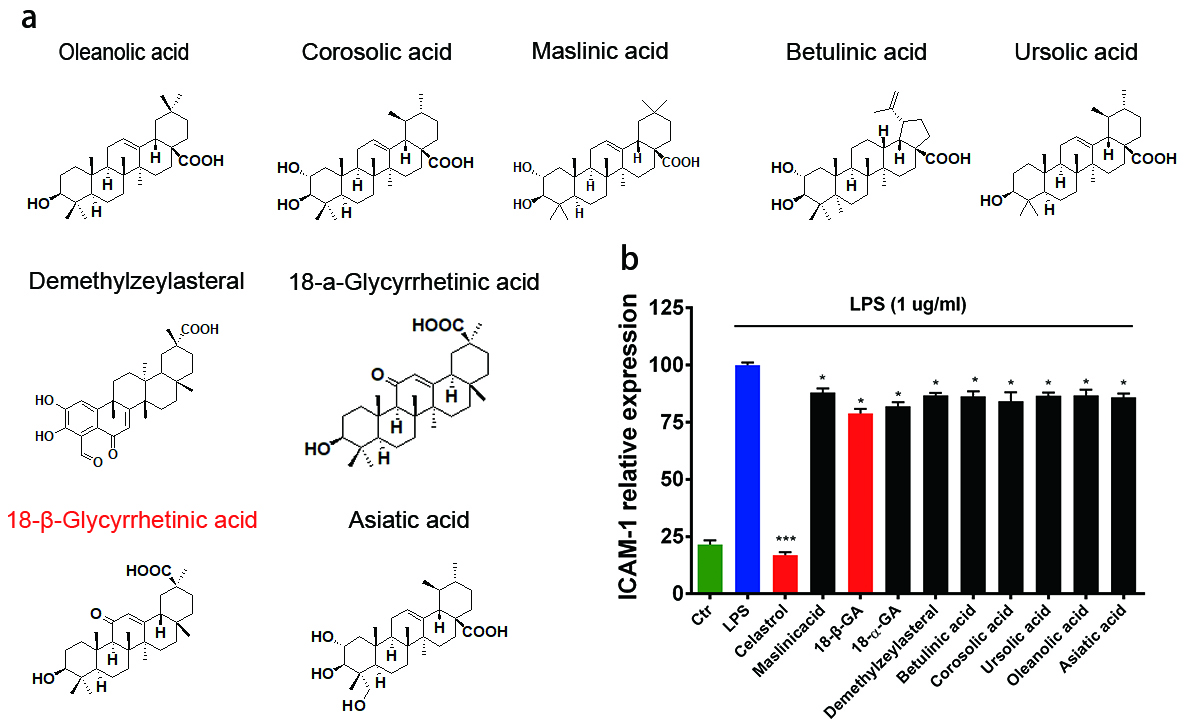


Supplementary Fig 1 Commercial available triterpenoids and anti-inflammatory activity assay. (a) Structures of commercial available triterpenoids; (b) Anti-inflammatory activity assay. All chemical treatments were compared to LPS treatment alone for significance assay. p values were determined by two-way ANOVA with Student’s t test (*p < 0.05, ***p < 0.001).


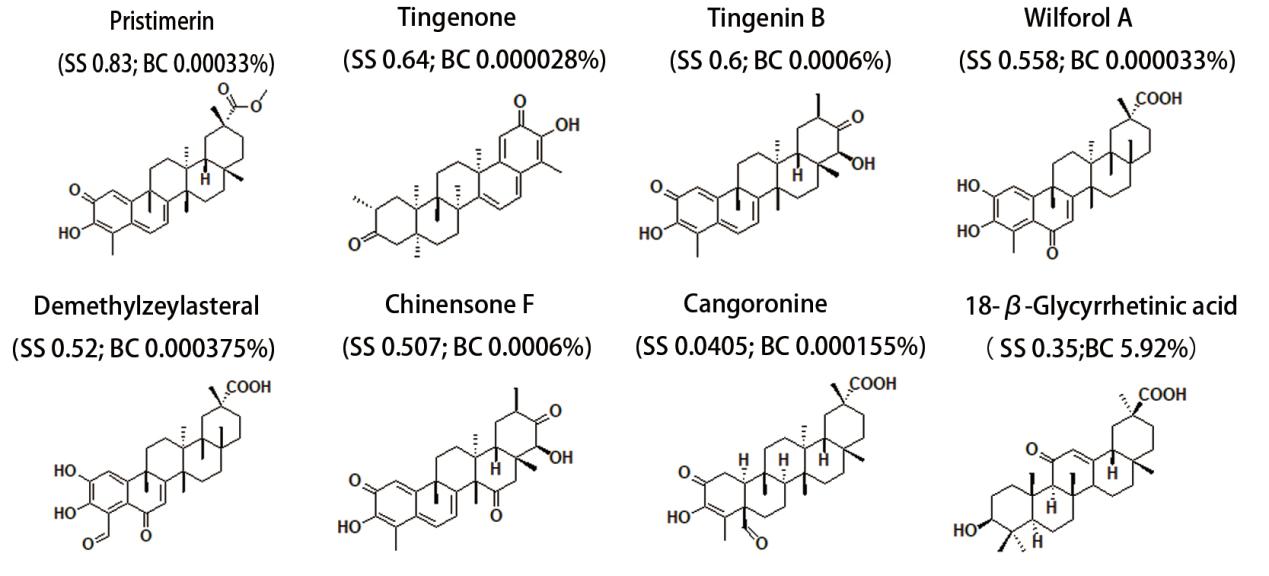


Supplementary Fig 2 Top 8 triterpenoids of highest structural similarity scores

to celastrol


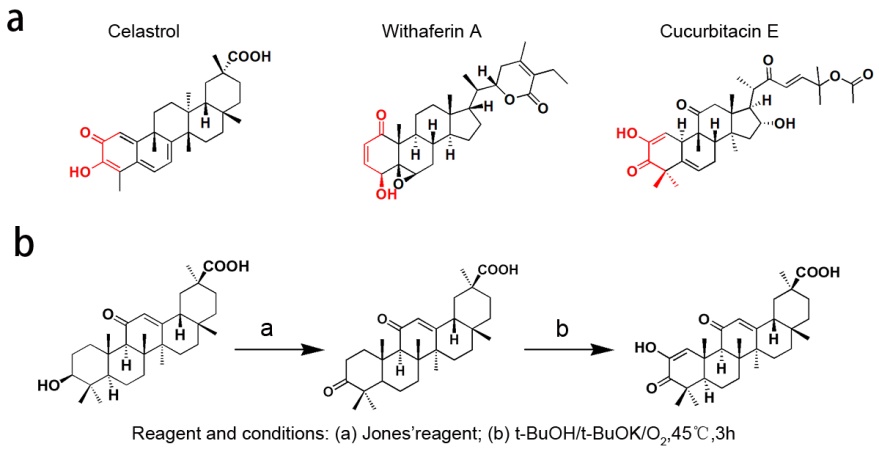


Supplementary Fig 3 **Hemisynthesis of GA-02** (a) The structure of a few potent anti-inflammatory natural products bearing a conjugated carbonyl and a nearby hydroxyl group on ring A; (b) Structure modification from GA to GA-02，and related conditions for each reaction .


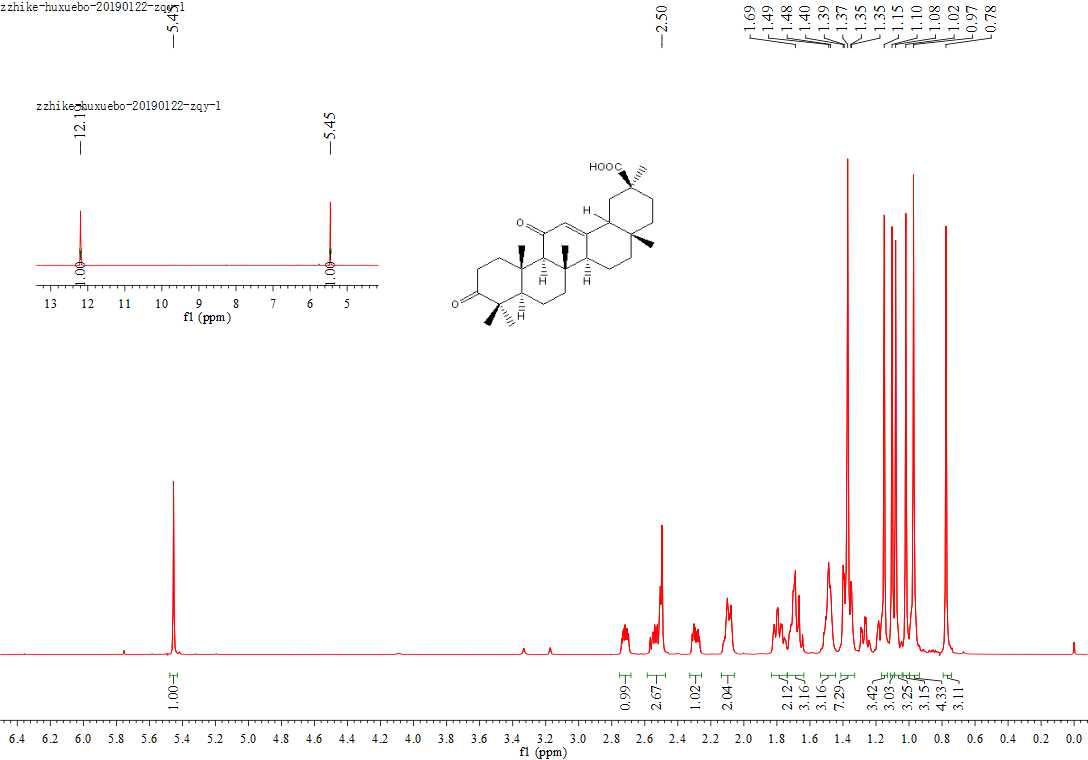


Supplementary Fig 4a ^1^H NMR (600MHz) spectrum of GA-01 in DMSO-d6


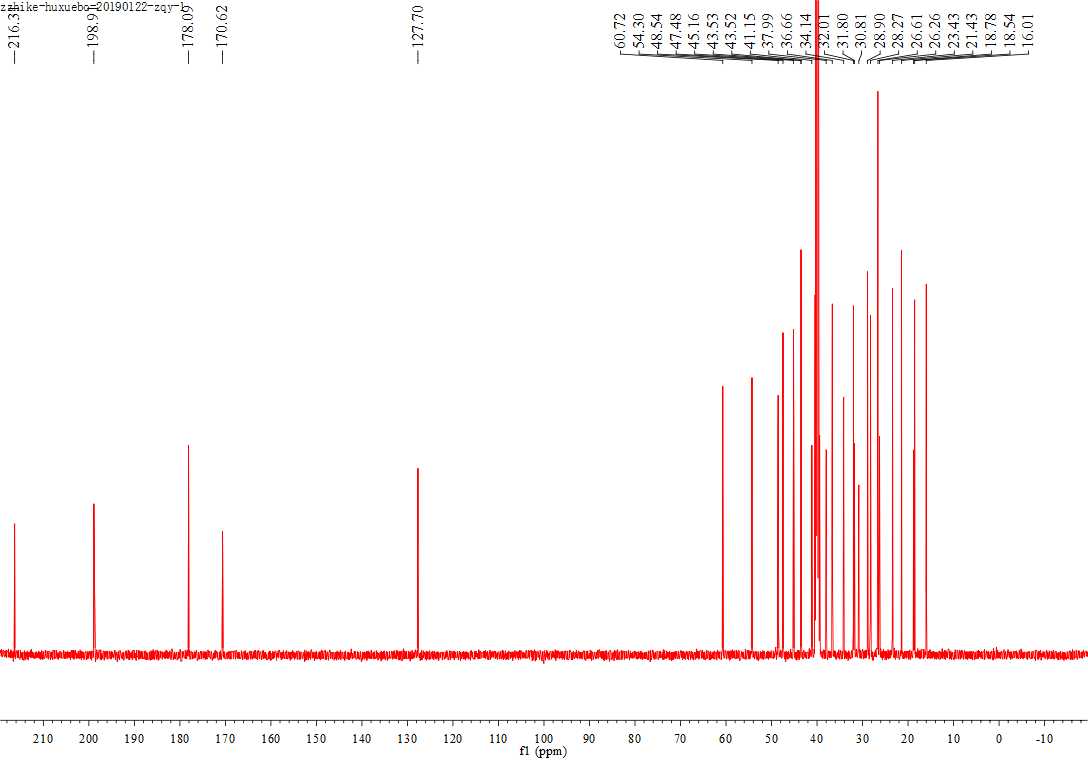


Supplementary Fig 4b ^13^C NMR (150MHz) spectrum of GA-01 in DMSO-d6

Supplementary Fig 4c HRESIMS spectrum of of GA-01(Positive and negative ion modes).


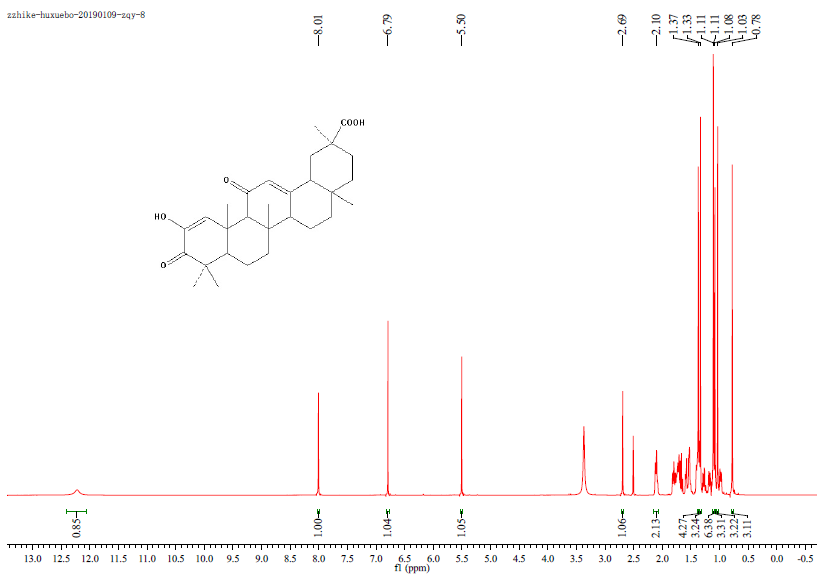


Supplementary Fig 5a ^1^H NMR (600MHz) spectrum of GA-02 in DMSO-d6


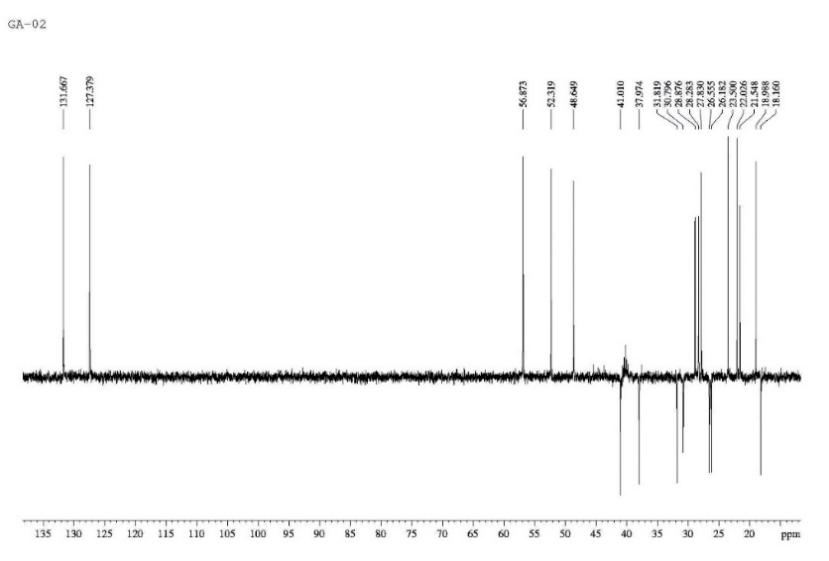


Supplementary Fig 5b DEP135 NMR (150MHz) spectrum of GA-02 in DMSO-d6


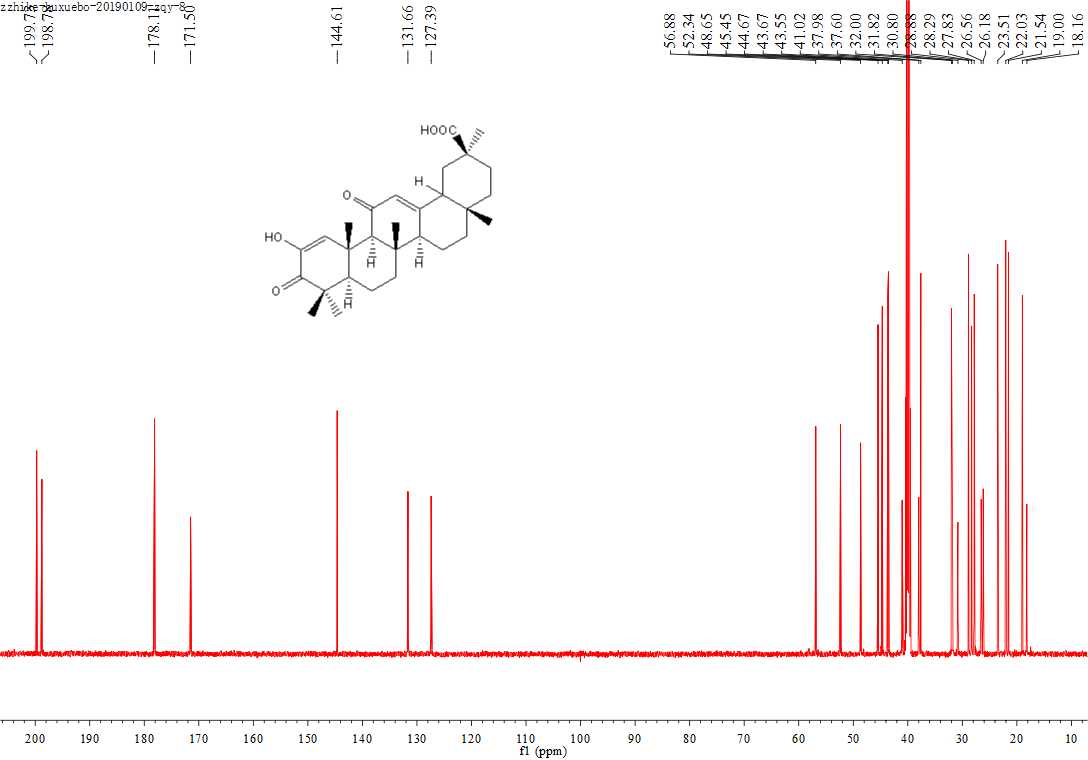


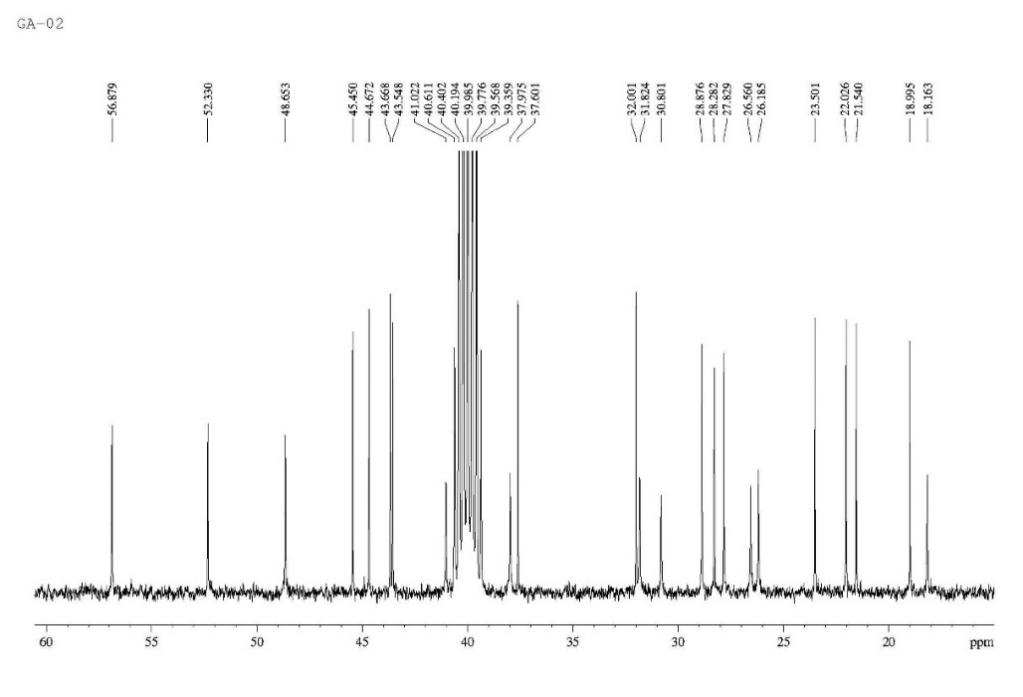


Supplementary Fig 5c 13C NMR (150MHz) spectrum of GA-02 in DMSO-d6

Supplementary Fig 5d HRESIMS spectrums of GA-02(Positive and negative ion modes).


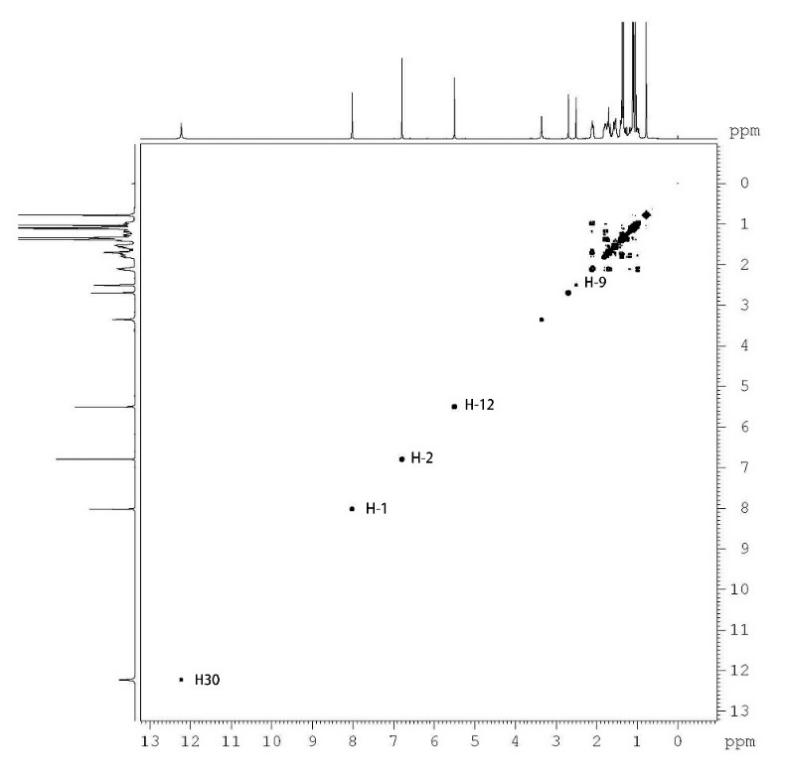


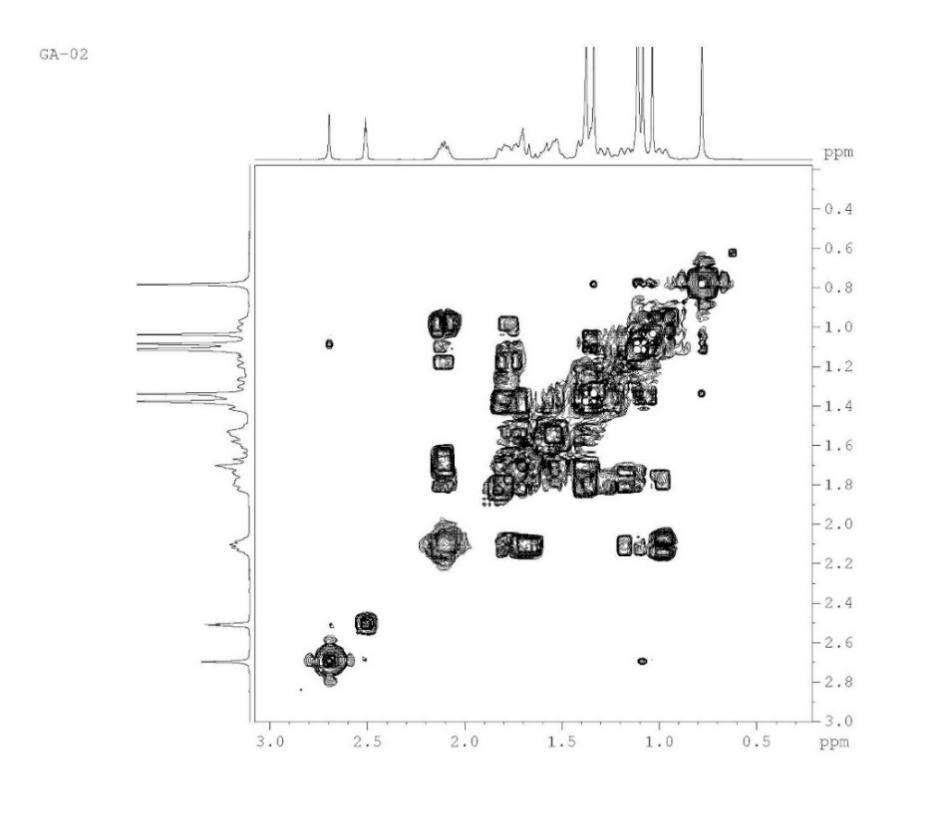


Supplementary Fig 5e COSY (600MHz) spectrum of GA-02 in DMSO-d6


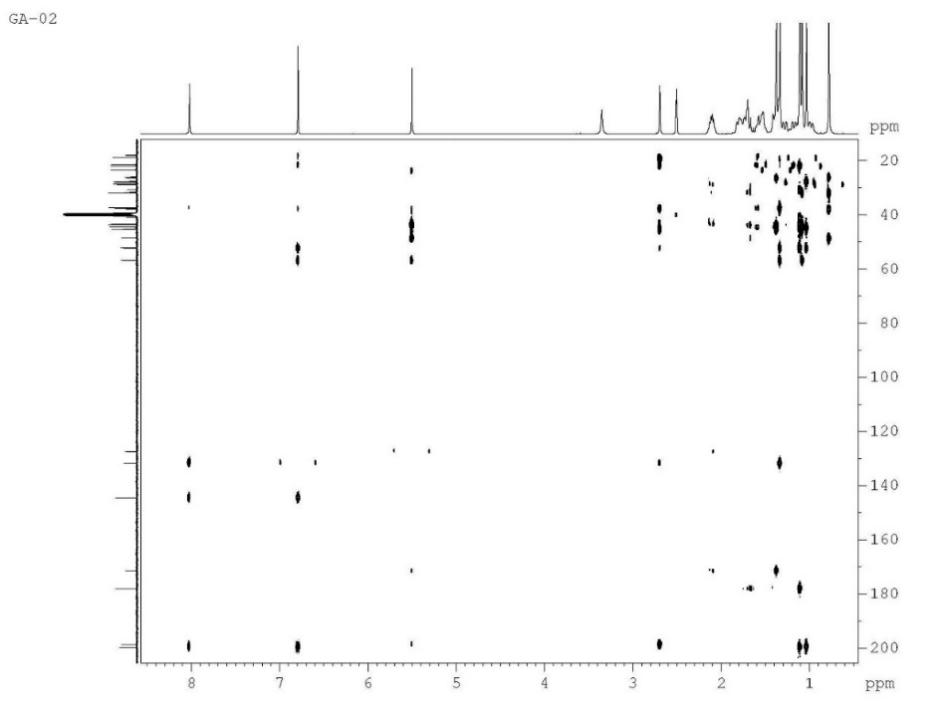


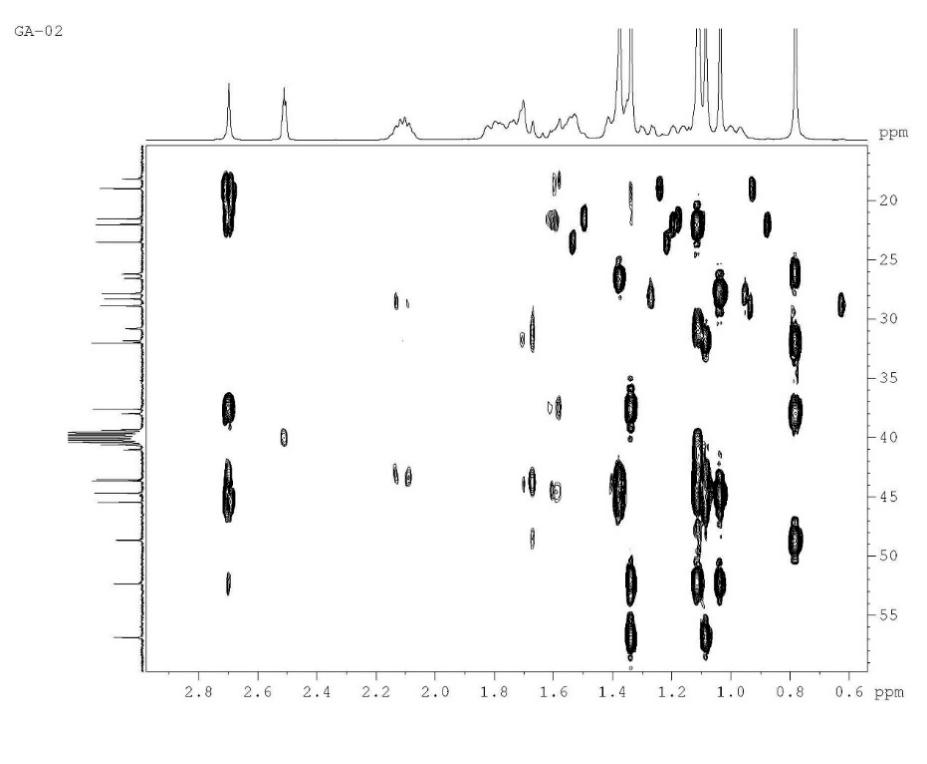


Supplementary Fig 5f HMBC (600MHz) spectrum of GA-02 in DMSO-d6


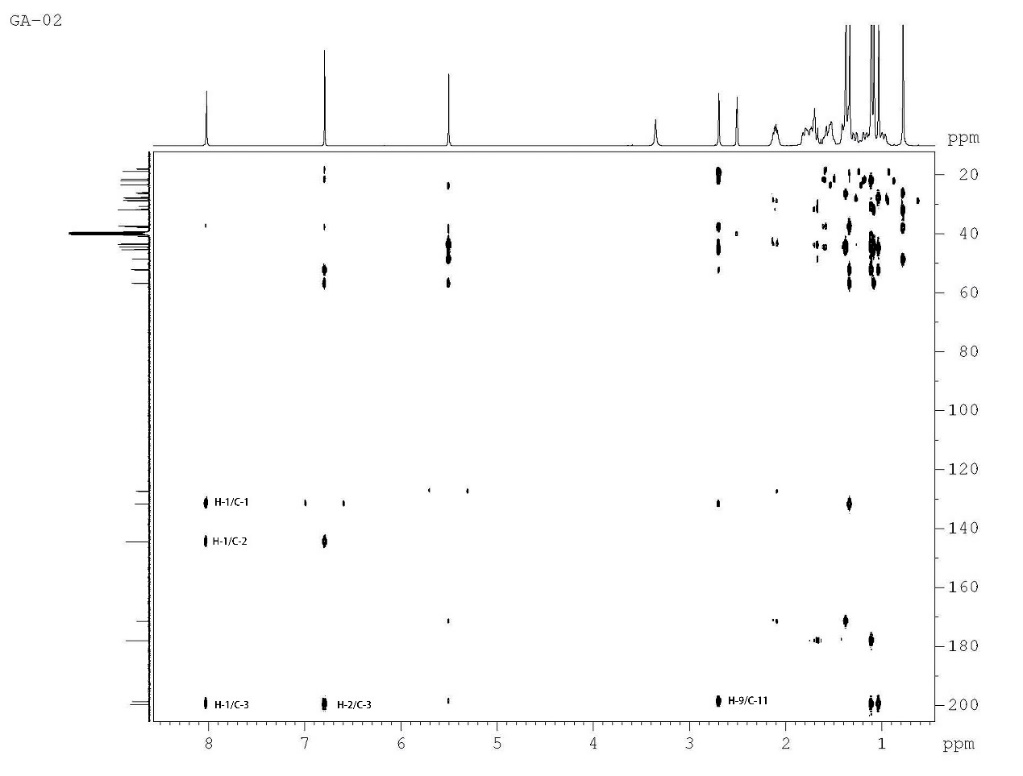


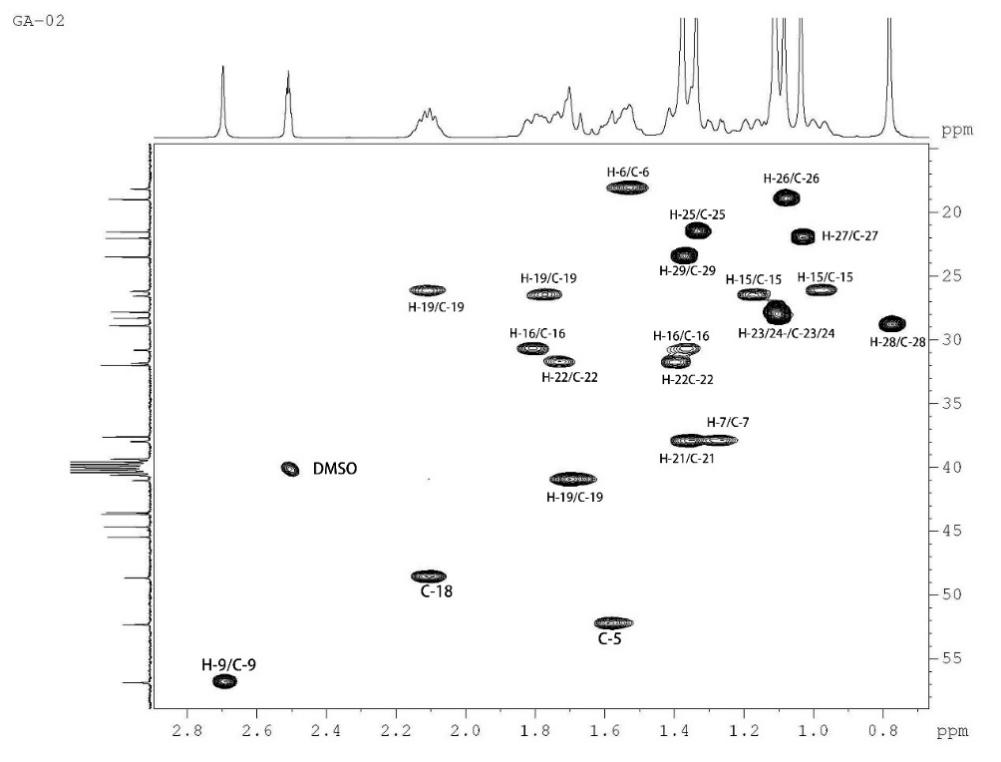


Supplementary Fig 5g HSQC (600MHz) spectrum of GA-02 in DMSO-d6


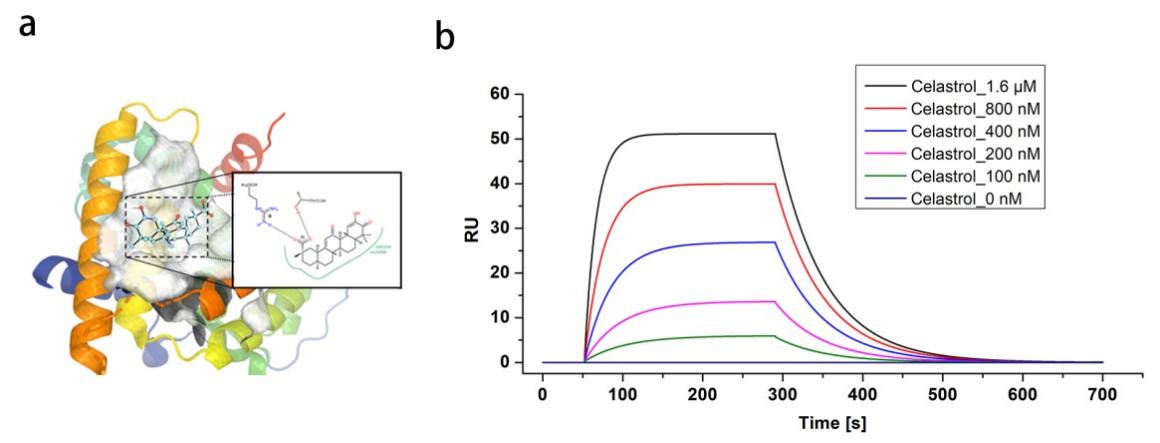


Supplementary Fig 6. Celastrol binding to receptor Nur77. (a) The binding mode of celastrol with Nur77 generated by PoseView. Nur77 (PDB code 4JGV) is shown as ribbon diagram, and celastrol as green ball-and-stick model. (b) Celastrol binding to Nur77 was illustrated by SPR assay of purified Nur77-LBD with celastrol.


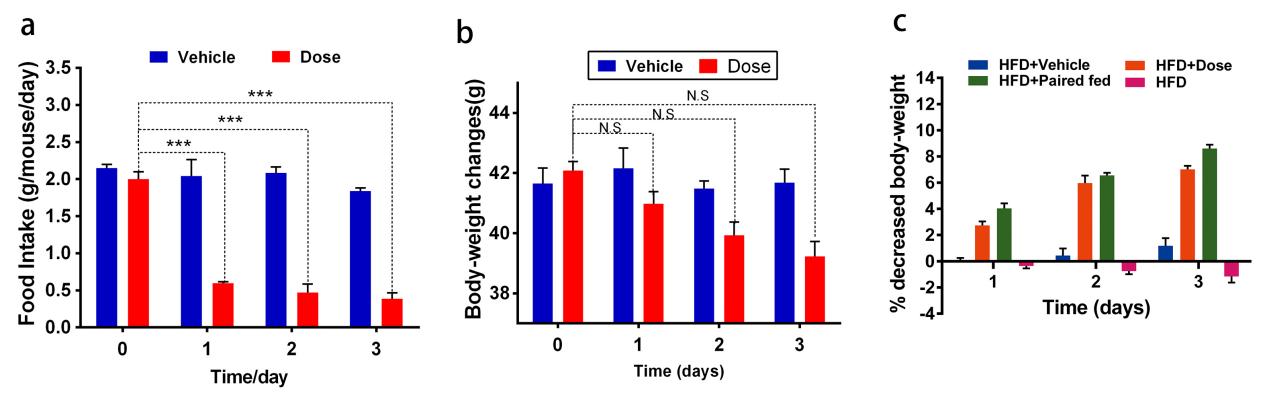


Supplementary Fig 7 Food intake and body weight changes during the first three days of high dose GA-02 treatment. (a) Daily food intake；(b) Body weight changes and (c) Body weight changes% during the first three days of GA-02 (20mg/kg) treatment. (n = 5, vehicle; n = 5, GA-02; n = 5, paired-fed; n = 5, HFD). p values were determined by two-way ANOVA with Student’s t test (*p < 0.05, **p < 0.01, ***p < 0.001).


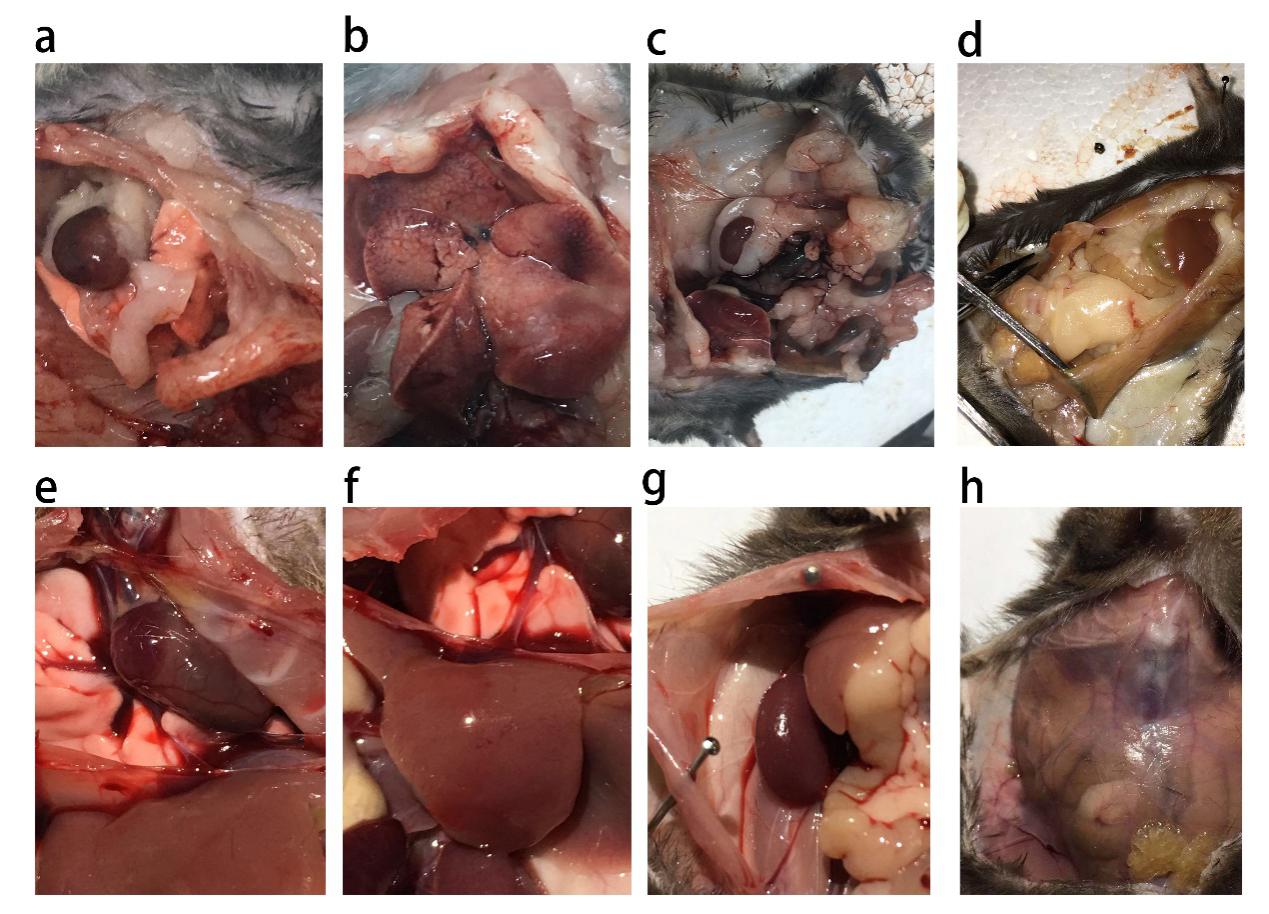


Supplementary Fig 8 Representative image of visceral tissue from DIO mice.(a) heart, (b)Liver, (c)Kidney, (d) Epididymal fat; visceral tissue image from DIO mice after two weeks of GA-02 treatment (e) Heart of DIO mice after 2-week treatment of GA-02, (f) Liver, (g) Kidney and (h) Subcutaneous fat image of GA-02 treatment.


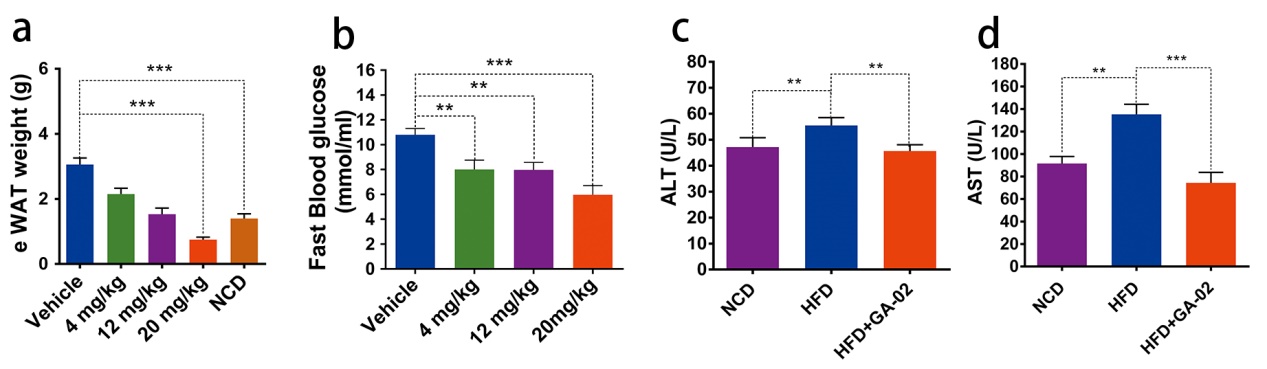


Supplementary Fig 9 Tissue and blood parameters of mice. (a) epididymal fat weight for each treated group (n = 4 for vehicle group; n = 3 for each GA-02 group). (b) Fasted blood glucose for each treated group (n = 4 for vehicle group; n = 3 for each GA-02 group). (c) ALT and (d) AST (U/L, n = 4 for each group). Values are represented as mean ± SD. (**p* < 0.05, ***p* < 0.01, ****p* < 0.001).

**
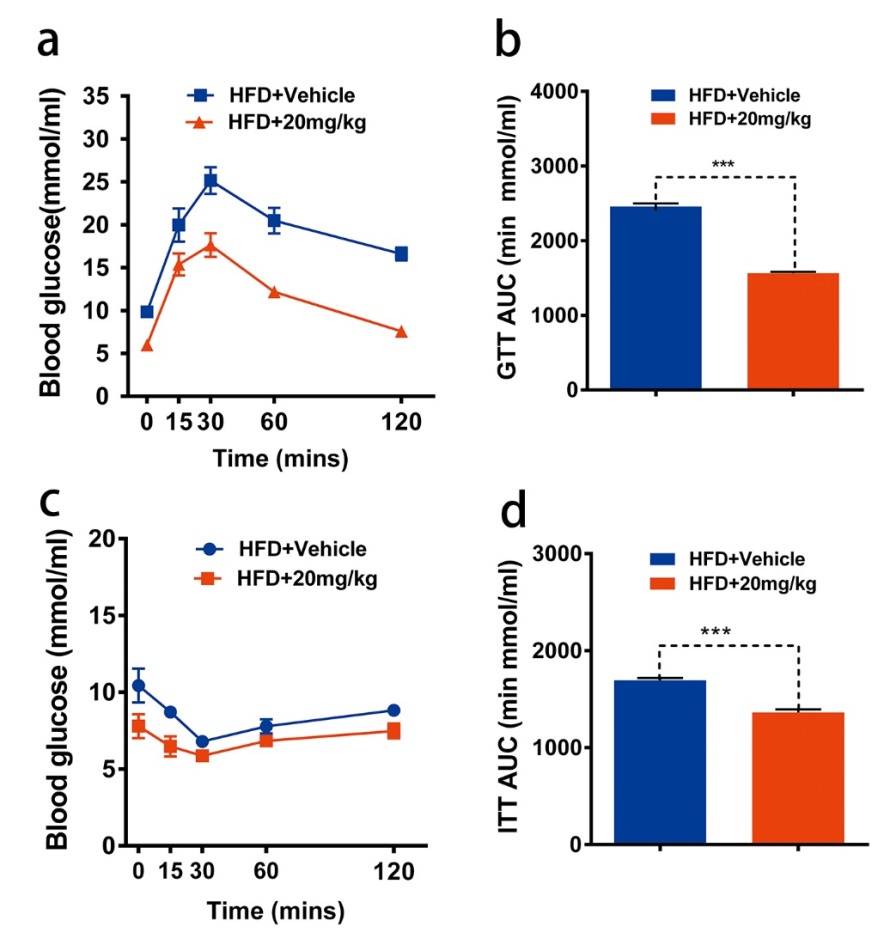
**

Supplementary Fig 10 GA-02 Improves Glucose Homeostasis in DIO Mice**.** Mice were acclimated for four days with daily i.p. injections of vehicle. Subsequently, mice were treated with vehicle (Vehicle) or GA-02 (20 mg/kg) (i.p. once a day) for two weeks. (a) Glucose tolerance test (GTT) of DIO mice after two-week treatments (n = 5 for Vehicle; n = 5 for GA-02). (b) Area under the curve (AUC) of GTT. (c) Insulin tolerance test (ITT) after two-week treatments. (d) AUC of ITT. Error bars are represented as mean ± SEM. p values were determined by two-way ANOVA with Student’s t test (*p < 0.05, **p < 0.01, ***p < 0.001).


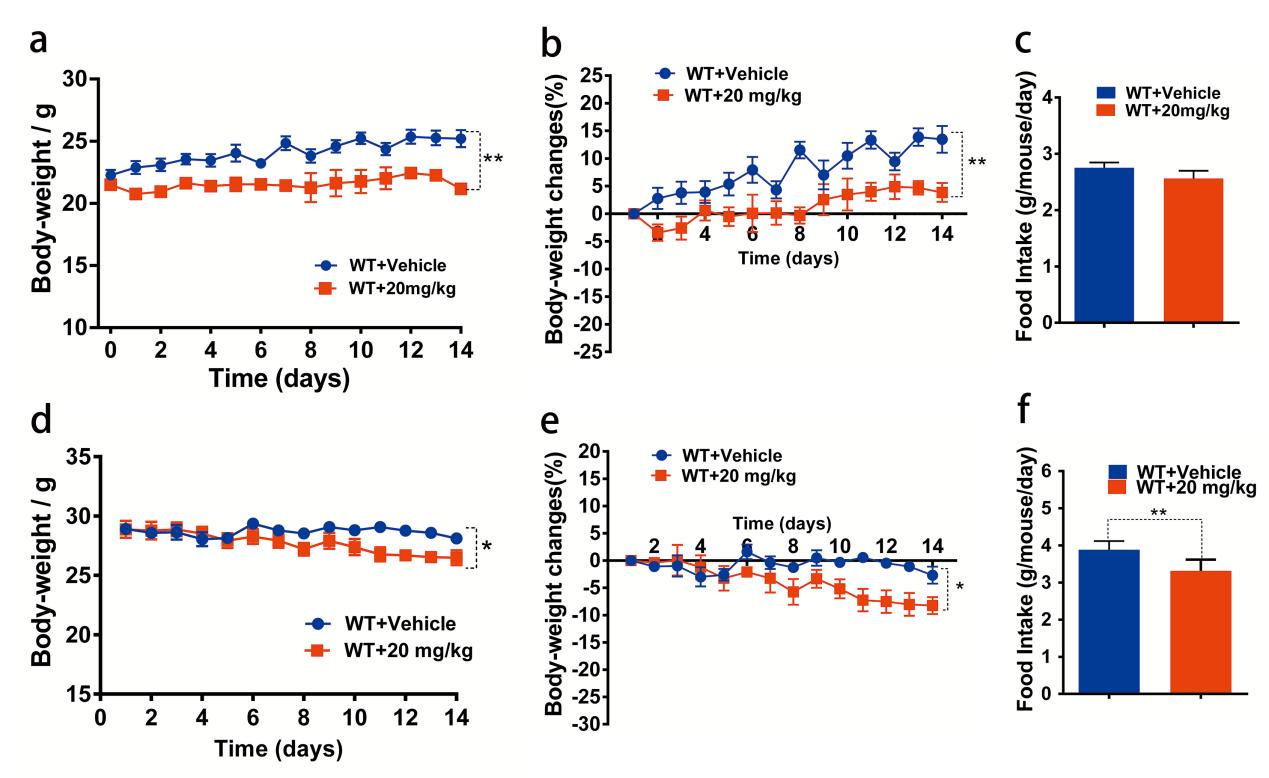


Supplementary Fig 11 **The effects of GA-02 in lean and old-lean mice.** Lean and old-lean mice received vehicle or GA-02 (20 mg/kg; i.p.) for 2 weeks. (a) The body weight changes of lean, (b) the percentage of body weight changes (%) of lean mice, (c) the average daily food intake over the first week in lean mice group (n = 6 for vehicle; n = 8 for GA-02 group). (d) The body weight changes of old lean mice, (e) the percentage of body weight changes (%) of old lean mice, (f) the average daily food of intake over the first week of old-lean mice group (n = 6 for vehicle group; n = 8 for GA-02 group). Body weight values are represented as mean ± SEM (**p* < 0.05, ***p* < 0.01, ****p* < 0.001).


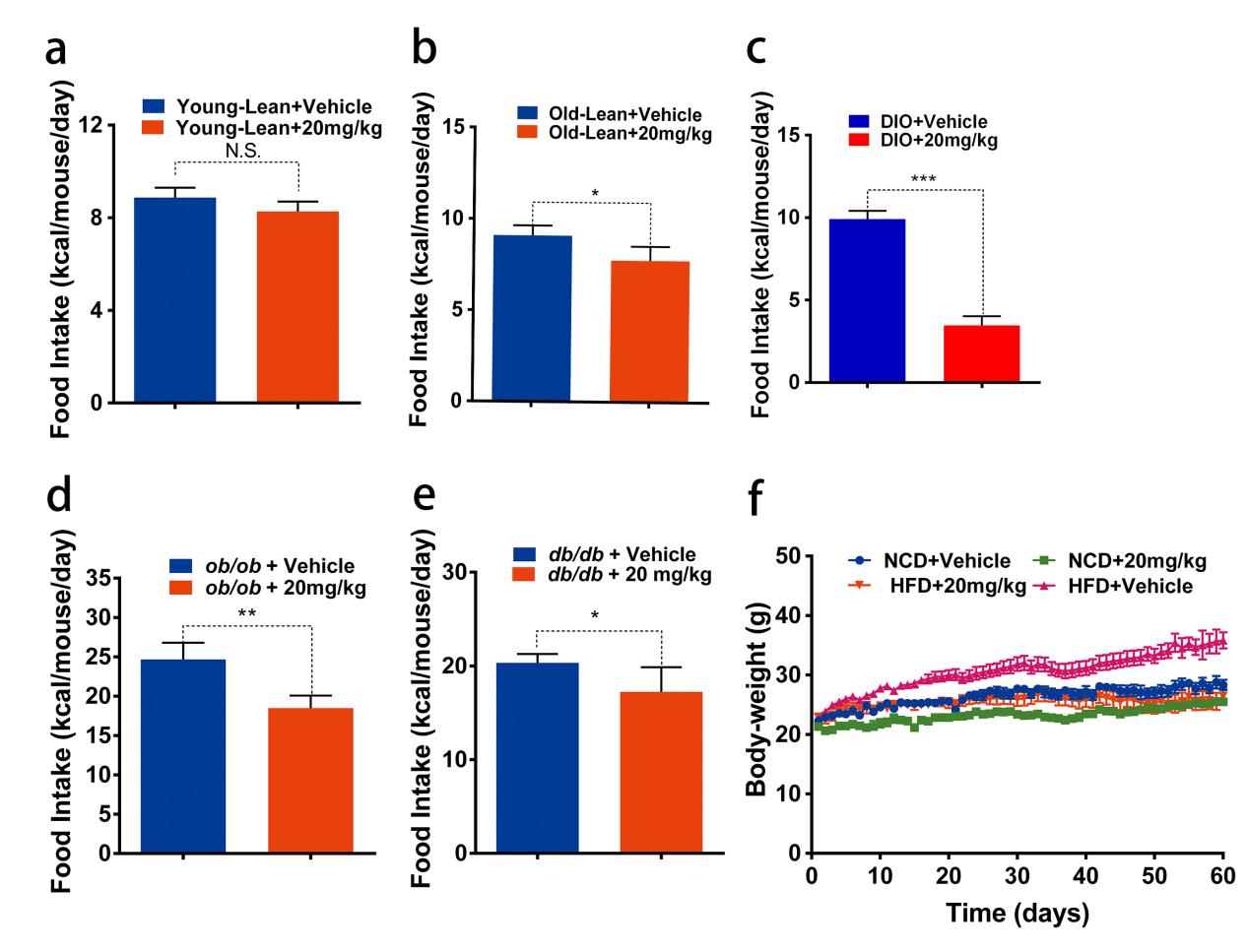


Supplementary Fig 12 Food Intake Calculation as Calories and Long-Term GA-02 Treatment. (a–e) Daily food consumption (kcal/mouse/day) of mice. (f) Body weight changes during 60 days of treatment (n = 6 vehicle/normal chow diet, n = 6 GA-02/normal chow diet, n = 6 vehicle/HFD, n = 6 GA-02/HFD). WT male mice fed with chow or high fat diet were injected either with vehicle or GA-02 (20 mg/kg, once a day, i.p.) for 60 days. Error bars are represented as mean ± SEM. p values were determined by two-way ANOVA with Student’s t test (*p < 0.05, **p < 0.01, ***p < 0.001).


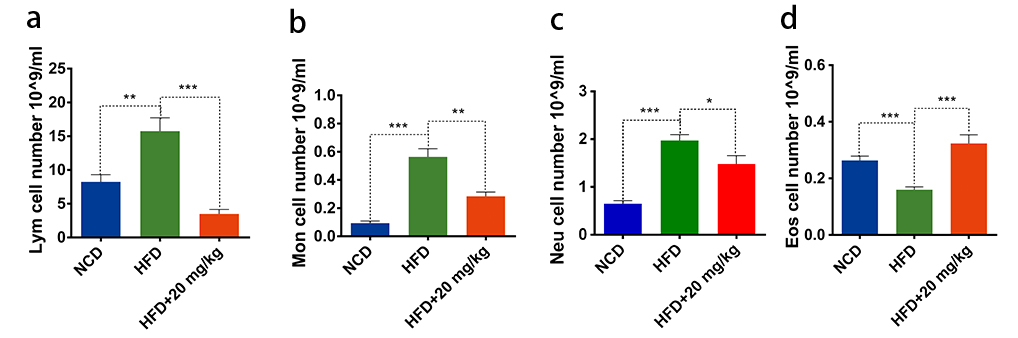


Supplemental Fig 13 Plasma hematology assay (a) Lym cell, (b)Mon cell, (c)Neu cell, (d)Eos cell number from the plasma of DIO mice that were treated with HFD or HFD+GA-02. Error bars are represented as mean ± SEM. p values were determined by by Student’s t test (**p* < 0.05, ***p* < 0.01, ****p* < 0.001).


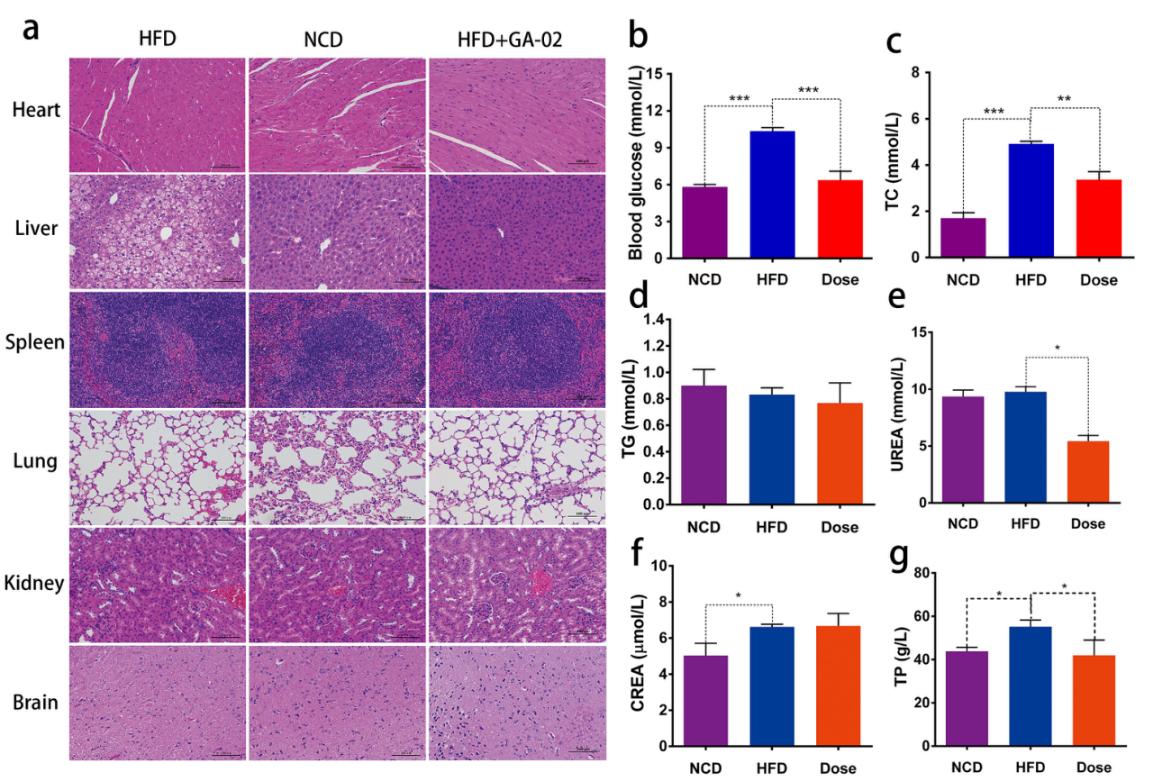


Supplementary Fig 14 No detectable toxicity in mice upon GA-02 treatment. (a) Tissue H&E staining assay after 14 days of GA-02 (20 mg/kg; i.p.) treatment. Scale bars, 100 μm. The blood biochemical parameters include (b) glucose (GLU), (c) total cholesterol (TC), (d) triglycerides (TG), (e) urea (UREA), (f) creatinine (CREA) and (g) total proteins (TP) levels after 14 days treatment. Error bars are represented as mean ± SEM. p values were determined by by Student’s t test (**p* < 0.05, ***p* < 0.01, ****p* < 0.001).


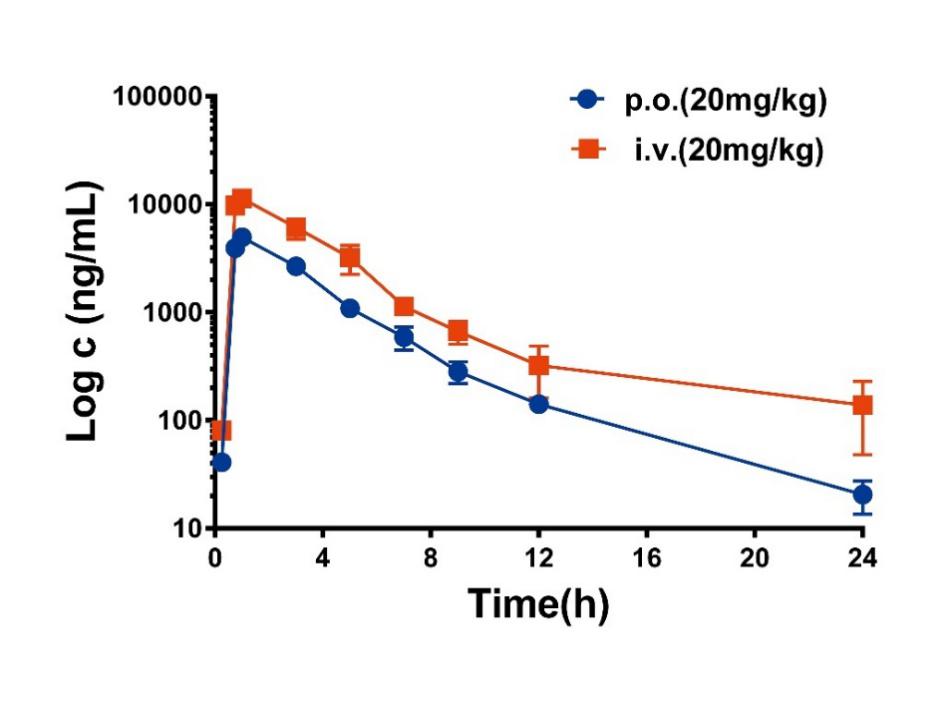


Supplementary Fig 15 Plasma concentration-time profiles of GA-02. After intravenous administration of GA-02 (20mg/kg) to rats, blood samples were taken from the suborbital vein at 0,15, 30, 45, 60 min, and 2 h, 3 h, 5 h, 7 h, 9 h, 12 h, 24 h and analyzed by LC-MS/MS.


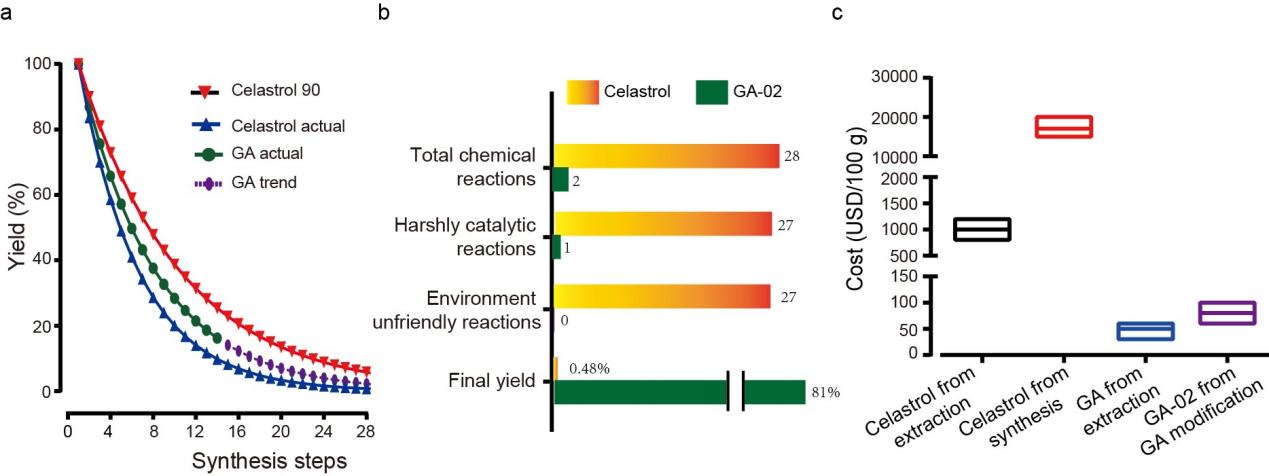


Supplementary Fig 16. A comparison of different production strategies for celastrol and GA-02. (a) A computational model of the catalytic efficiency of chemical synthesis and biosynthesis. An exponential curve modeled the dynamic of chemically synthesized celastrol with a constant catalytic rate for each step (Celastrol actual) or a hypothetical 90% catalytic rate for each step (Celastrol 90). GA was also modeled in the same way for 14 steps of production (GA actual) and extended for further 14 steps at the same catalytic rate (GA trend). (b) A characteristic comparison of total synthesis of celastrol and GA-02 production. (c) Prices of different chemical on market (celastrol and GA from plant extraction) and predicted cost based on small scale production (celastrol synthesis and GA-02 obtained by our strategy).

| Table S1. The libirary of plant triterpenoids | | | | | | | | |
| --- | --- | --- | --- | --- | --- | --- | --- | --- |
| **ID** | ENG Name | CAS Number | Structure | Type | Content | Original Content | Source | Reference |
| **1** | Niloticin | 115404-57-4 | 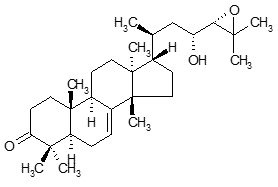 | Triterpenoids | 0.000300% | 80mg/30kg | *phellodendron chinense* | Chem.Pharm.Bull.38(6) 1616-1619 (1990) |
| **2** | Simiarenol | 1615-94-7 | 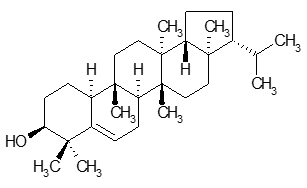 | Triterpenoids | 0.000045% | 1.5mg/3.3kg | dry peels sweet potatoes | Chemistry & Biodiversity (2019), 16(3) |
| **3** | Ursolic acid | 77-52-1 | 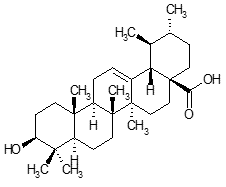 | Triterpenoids | 3.00000% | 3.00000% | *Eriobotrya japonica(Thunb.)Lind*l | Planta Med,2003,69:327 |
| **4** | 3β-Acetoxyurs-11-en-28,13β-olide | 35959-08-1 | 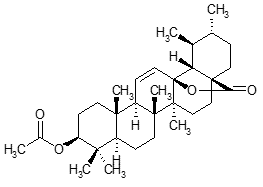 | Triterpenoids | 0.00050% | 0.00050% | *E. camaldulensis var. obtusa* | JNP, 2000, 63, No. 9 |
| **5** | 3β-Hydroxyurs-11-en-28,13β-olide | 35959-05-8 | 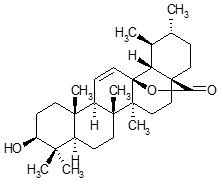 | Triterpenoids | 0.00035% | 0.00035% | *E. camaldulensis var. obtusa* | JNP, 2000, 63, No. 9 |
| **6** | Betulin | 473-98-3 | 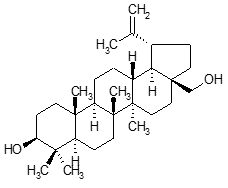 | Triterpenoids | 0.0090% | 0.0090% | *Betula platyphylla Suk* | *Fitoterapia (1989), 60(5), 468-9* |
| **7** | Betulinic acid | 472-15-1 | 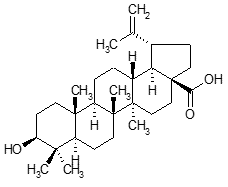 | Triterpenoids | 0.1166% | 0.1166% | *Betula platyphylla Suk* | *Fitoterapia (1989), 60(5), 468-9* |
| **8** | Acetylursolic acid | 7372-30-7 | 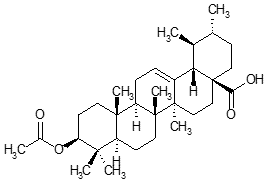 | Triterpenoids | 0.000500% | 20mg/4kg | M. nigra stem barks | Natural Product Research (2014), 28(13), 952-959. |
| **9** | uvaol | 545-46-0 | 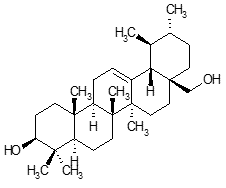 | Triterpenoids | 0.001000% | 40mg/4kg | M. nigra stem barks | Natural Product Research (2014), 28(13), 952-959. |
| **10** | 20S,24R-Epoxydammara-12β,25-diol-3-one | 25279-15-6 | 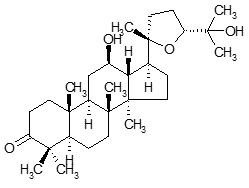 | Triterpenoids | 0.000360% | 90mg/25kg | stem bark of T. sinensis | Fitoterapia (2016), 112, 97-103. |
| **11** | ocotillone | 35761-54-7 |  | Triterpenoids | 0.002620% | 655mg/25kg | stem bark of T. sinensis | Fitoterapia (2016), 112, 97-103. |
| **12** | (20S,24R)-epoxydammarane-3β,25-diol -marane-3β,25-diol | 5986-39-0 |  | Triterpenoids | 0.000024% | 6mg/25kg | stem bark of T. sinensis | Fitoterapia (2016), 112, 97-103. |
| **13** | methyl shoreate | 21671-01-2 |  | Triterpenoids | 0.000048% | 12mg/25kg | stem bark of T. sinensis | Fitoterapia (2016), 112, 97-103. |
| **14** | shoreic acid | 21671-00-1 |  | Triterpenoids | 0.000493% | 123.3mg/25kg | stem bark of T. sinensis | Fitoterapia (2016), 112, 97-103. |
| **15** | richenone | 134528-34-0 |  | Triterpenoids | 0.000052% | 13mg/25kg | stem bark of T. sinensis | Fitoterapia (2016), 112, 97-103. |
| **16** | cylindrictone D | 1075182-95-4 |  | Triterpenoids | 0.000057% | 14.3mg/25kg | stem bark of T. sinensis | Fitoterapia (2016), 112, 97-103. |
| **17** | piscidinol A | 100198-09-2 |  | Triterpenoids | 0.000273% | 68.3mg/25kg | stem bark of T. sinensis | Fitoterapia (2016), 112, 97-103. |
| **18** | hispidol B | 78739-39-6 |  | Triterpenoids | 0.000880% | 220mg/25kg | stem bark of T. sinensis | Fitoterapia (2016), 112, 97-103. |
| **19** | hollongdione | 32206-97-6 |  | Triterpenoids | 0.000054% | 13.5mg/25kg | stem bark of T. sinensis | Fitoterapia (2016), 112, 97-104. |
| **20** | bourjotinolone B | 6985-31-5 |  | Triterpenoids | 0.000093% | 23.3mg/25kg | stem bark of T. sinensis | Fitoterapia (2016), 112, 97-104. |
| **21** | Ocotillone | 22549-21-9 | 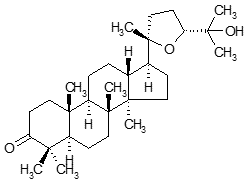 | Triterpenoids | 0.001486% | 5.5mg/370g | s. Tetrigona melanoleuca propolis | PLoS One (2015), 10(5), e0126886/1-e0126886/11 |
| **22** | ocotillone II | 19865-86-2 | 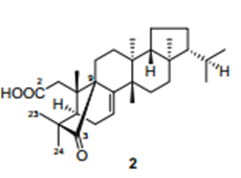 | Triterpenoids | 0.001297% | 4.8mg/370g | s. Tetrigona melanoleuca propolis | PLoS One (2015), 10(5), e0126886/1-e0126886/11 |
| **23** | Saikosaponin b2 | 58316-41-9 | 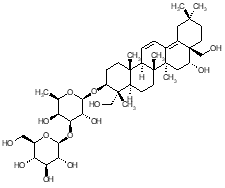 | Triterpenoids | 0.000440% | 22mg/5kg | aerial parts of B. chinense | Natural Product Communications (2008), 3(2), 155-159 |
| **24** | Shoreic acid | 21671-00-1 | 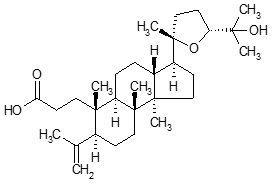 | Triterpenoids | 0.000180% | 9mg/5kg | roots of A. odorata | Natural Product Research (2016), 30, (4), 433-437 |
| **25** | 3-Acetoxy-27-hydroxy-20(29)-lupen -28-oic acid methyl ester | 263844-80-0 | 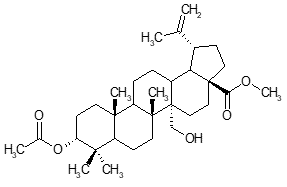 | Triterpenoids | 0.005208% | 250mg/4.8kg | roots of P. nigellastrum | J. Nat. Prod.2000,63,3,390-392 |
| **26** | 3,4-Secocucurbita-4,24-diene-3,26,29-trioic acid | 329975-47-5 |  | Triterpenoids | 0.001608% | 19.3mg/1.2kg | Fruiting bodies of R. lepida | Phytochemistry. 2016 Jul;127:63-8 |
| **27** | Alstonic acid A | 1159579-44-8 |  | Triterpenoids | 0.001929% | 164mg/8.5kg | leaves of A. scholaris | Phytochemistry (Elsevier) (2009), 70, (5), 650-654 |
| **28** | Alstonic acid B | 1159579-45-9 | 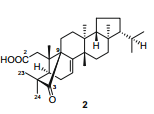 | Triterpenoids | 0.000153% | 13mg/8.5kg | leaves of A. scholaris | Phytochemistry (Elsevier) (2009), 70, (5), 650-654 |
| **29** | Walsunoid A | 1885961-62-5 |  | Triterpenoids | 0.000042% | 5mg/12kg | leaves of W. robusta | J Nat Prod. 2016 Apr 22;79(4):899-906 |
| **30** | Walsunoid B | 885969-11-8 |  | Triterpenoids | 0.000208% | 25mg/12kg | leaves of W. robusta | J Nat Prod. 2016 Apr 22;79(4):899-906 |
| **31** | Walsunoid C | 1885961-65-8 |  | Triterpenoids | 0.000108% | 13mg/12kg | leaves of W. robusta | J Nat Prod. 2016 Apr 22;79(4):899-906 |
| **32** | Walsunoid D | 1885961-67-0 |  | Triterpenoids | 0.000033% | 4mg/12kg | leaves of W. robusta | J Nat Prod. 2016 Apr 22;79(4):899-906 |
| **33** | Walsunoid E | 1885961-69-2 |  | Triterpenoids | 0.000025% | 3mg/12kg | leaves of W. robusta | J Nat Prod. 2016 Apr 22;79(4):899-906 |
| **34** | Walsunoid F | 1885961-71-6 |  | Triterpenoids | 0.000300% | 36mg/12kg | leaves of W. robusta | J Nat Prod. 2016 Apr 22;79(4):899-906 |
| **35** | Walsunoid G | 1885961-73-8 |  | Triterpenoids | 0.000125% | 15mg/12kg | leaves of W. robusta | J Nat Prod. 2016 Apr 22;79(4):899-906 |
| **36** | Walsunoid H | 1885961-75-0 |  | Triterpenoids | 0.000133% | 16mg/12kg | leaves of W. robusta | J Nat Prod. 2016 Apr 22;79(4):899-906 |
| **37** | Walsunoid I | 1885961-77-2 |  | Triterpenoids | 0.000042% | 5mg/12kg | leaves of W. robusta | J Nat Prod. 2016 Apr 22;79(4):899-906 |
| **38** | Serratenediol | 2239-24-9 | 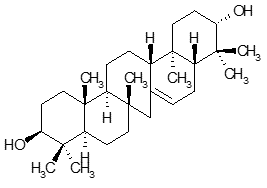 | Triterpenoids | 0.001140% | 17.1mg/1.5kg | H. phlegmaria | Natural Product Research (2019), 33, (14), 2051-2059 |
| **39** | 21β-Hydroxyserrat-14-en-3β-yl acetate | 1260-05-5 |  | Triterpenoids | 0.002020% | 30.3mg/1.5kg | H. phlegmaria | Natural Product Research (2019), 33, (14), 2051-2060 |
| **40** | 21α-Hydroxyserrat-14-en-3β-yl acetate | 35302-52-4 |  | Triterpenoids | 0.003333% | 50mg/1.5kg | H. phlegmaria | Natural Product Research (2019), 33, (14), 2051-2061 |
| **41** | 21α-Hydroxyserrat-14-en-3β-ol | 2239-24-9 |  | Triterpenoids | 0.001140% | 17.1mg/1.5kg | H. phlegmaria | Natural Product Research (2019), 33, (14), 2051-2062 |
| **42** | Corosolic acid | 4547-24-4 | 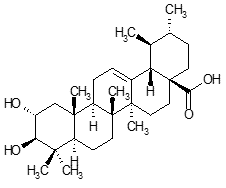 | Triterpenoids | 0.01000% | 0.01000% | *Lagerstroemia speciosa L* | Phytochemistry. 2014 Oct;106:94-103. |
| **43** | 2α,3α,23-Trihydroxyolean-12-en-28-oic acid | 102519-34-6 | 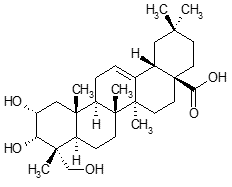 | Triterpenoids | 0.000110% | 11mg/10kg | leaves of E. japonica | Chemistry of Natural Compounds (2019), 55, (1), 169-171 |
| **44** | Jacoumaric Acid | 63303-42-4 |  | Triterpenoids | 0.000160% | 16mg/10kg | leaves of E. japonica | Chemistry of Natural Compounds (2019), 55, (1), 169-171 |
| **45** | Euscaphic Acid | 53155-25-2 |  | Triterpenoids | 0.002000% | 200mg/10kg | leaves of E. japonica | Chemistry of Natural Compounds (2019), 55, (1), 169-171 |
| **46** | Arjunic Acid | 31298-06-3 |  | Triterpenoids | 0.000200% | 20mg/10kg | leaves of E. japonica | Chemistry of Natural Compounds (2019), 55, (1), 169-171 |
| **47** | 5-Glutinen-3β-ol | 545-24-4 | 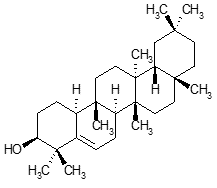 | Triterpenoids | 0.003125% | 20mg/640g | Poulsenia armata Miq. Standl | Natural Product Research (2005), 19, (2), 197-202 |
| **48** | Salvibuchanic acid | 1802384-44-6 |  | Triterpenoids | 0.001400% | 13.3mg/950g | roots of S. buchanani | Natural Product Research (2018), 32, (17), 2025-2030 |
| **49** | Hyptadienic acid | 128397-09-1 | 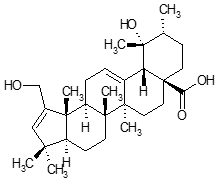 | Triterpenoids | 0.001400% | 13.3mg/950g | roots of S. buchanani | Natural Product Research (2018), 32, (17), 2025-2030 |
| **50** | 3α,27-Dihydroxylupen-20(29)-en-28-oic acid methyl ester | 263844-79-7 | 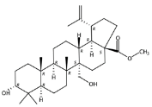 | Triterpenoids | 0.000333% | 16mg/4.8kg | leaves of A. scholaris w | J. Nat. Prod.2000,63,3,390-392 |
| **51** | 3α-acetoxy-27-hydroxylupen-20(29)-en-28-oic acid methyl ester | 263844-80-0 | 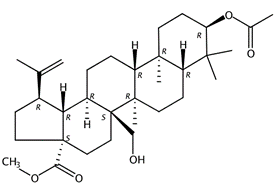 | Triterpenoids | 0.005208% | 250mg/4.8kg | leaves of A. scholaris w | J. Nat. Prod.2000,63,3,390-393 |
| **52** | 3α-Acetoxyolean-12-ene-27,28-dioic acid 28-methyl ester | 107180-43-8 | 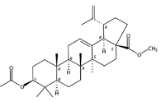 | Triterpenoids | 0.000313% | 15mg/4.8kg | leaves of A. scholaris w | J. Nat. Prod.2000,63,3,390-394 |
| **53** | 3,27-Dihydroxy-20(29)-lupen -28-oic acid methyl ester | 263844-79-7 | 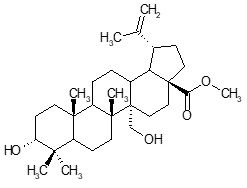 | Triterpenoids | 0.005208% | 250mg/4.8kg | leaves of A. scholaris w | J. Nat. Prod.2000,63,3,390-395 |
| **54** | Alstonic acid A | 1159579-44-8 |  | Triterpenoids | 0.001929% | 164mg/8.5kg | leaves of A. scholaris | Phytochemistry (Elsevier) (2009), 70, (5), 650-654 |
| **55** | Alstonic acid B | 1159579-45-9 | 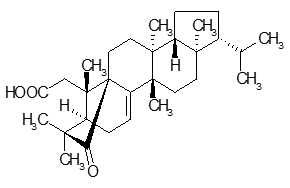 | Triterpenoids | 0.000153% | 13mg/8.5kg | leaves of A. scholaris | Phytochemistry (Elsevier) (2009), 70, (5), 650-654 |
| **56** | Sculponeatic acid | 1169806-02-3 | 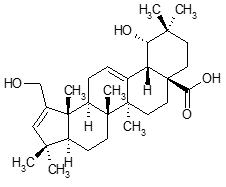 | Triterpenoids | 0.002000% | 50mg/2.5kg | aerial parts of I. sculponeata | Chemical & Pharmaceutical Bulletin (2009), 57, (5), 525-527 |
| **57** | Sculponeatin N | 1169805-98-4 |  | Triterpenoids | 0.008000% | 200mg/2.5kg | aerial parts of I. sculponeata | Chemical & Pharmaceutical Bulletin (2009), 57, (5), 525-528 |
| **58** | Sculponeatin O | 1169806-00-1 |  | Triterpenoids | 0.001200% | 30mg/2.5kg | aerial parts of I. sculponeata | Chemical & Pharmaceutical Bulletin (2009), 57, (5), 525-529 |
| **59** | Sculponeatic Acid | 1169806-02-3 |  | Triterpenoids | 0.002000% | 50mg/2.5kg | aerial parts of I. sculponeata | Chemical & Pharmaceutical Bulletin (2009), 57, (5), 525-530 |
| **60** | sculponeatin A | 85287-58-7 |  | Triterpenoids | 0.011480% | 287mg/2.5kg | aerial parts of I. sculponeata | Chemical & Pharmaceutical Bulletin (2009), 57, (5), 525-531 |
| **61** | sculponeatin K | 477529-70-7 |  | Triterpenoids | 0.000560% | 14mg/2.5kg | aerial parts of I. sculponeata | Chemical & Pharmaceutical Bulletin (2009), 57, (5), 525-532 |
| **62** | hyptadienic acid | 128397-09-1 |  | Triterpenoids | 0.009920% | 248mg/2.5kg | aerial parts of I. sculponeata | Chemical & Pharmaceutical Bulletin (2009), 57, (5), 525-533 |
| **63** | 3β-Hydroxylanosta-9(11),24Z-dien-26-oic acid | 129724-83-0 | 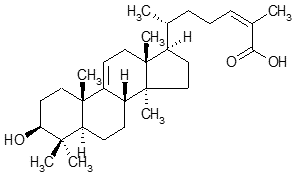 | Triterpenoids | 0.000155% | 10.1mg/6.5kg | stem of S. glaucescens | Planta Medica (2012), 78, (5), 472-479 |
| **64** | Schiglausin A | 1426820-46-3 |  | Triterpenoids | 0.000371% | 24.1mg/6.5kg | stem of S. glaucescens | Planta Medica (2012), 78, (5), 472-480 |
| **65** | Schiglausin B | 1426240-50-7 |  | Triterpenoids | 0.000126% | 8.2mg/6.5kg | stem of S. glaucescens | Planta Medica (2012), 78, (5), 472-481 |
| **66** | Schiglausin C | 1426820-47-4 |  | Triterpenoids | 0.000585% | 38mg/6.5kg | stem of S. glaucescens | Planta Medica (2012), 78, (5), 472-482 |
| **67** | Schiglausin D | 1426240-33-6 |  | Triterpenoids | 0.000968% | 62.9mg/6.5kg | stem of S. glaucescens | Planta Medica (2012), 78, (5), 472-483 |
| **68** | Schiglausin E | 1426241-52-2 |  | Triterpenoids | 0.000074% | 4.8mg/6.5kg | stem of S. glaucescens | Planta Medica (2012), 78, (5), 472-484 |
| **69** | Schiglausin F | 1426242-25-2 |  | Triterpenoids | 0.000131% | 8.5mg/6.5kg | stem of S. glaucescens | Planta Medica (2012), 78, (5), 472-485 |
| **70** | Schiglausin G | 1426241-63-5 |  | Triterpenoids | 0.000186% | 12.1mg/6.5kg | stem of S. glaucescens | Planta Medica (2012), 78, (5), 472-486 |
| **71** | Schiglausin H | 1426820-48-5 |  | Triterpenoids | 0.000162% | 10.5mg/6.5kg | stem of S. glaucescens | Planta Medica (2012), 78, (5), 472-487 |
| **72** | Schiglausin I | 1426570-34-4 |  | Triterpenoids | 0.000923% | 6.0mg/6.5kg | stem of S. glaucescens | Planta Medica (2012), 78, (5), 472-488 |
| **73** | Schiglausin J | 1426570-35-5 |  | Triterpenoids | 0.000100% | 6.5mg/6.5kg | stem of S. glaucescens | Planta Medica (2012), 78, (5), 472-489 |
| **74** | Coccinic acid | 107783-45-9 | 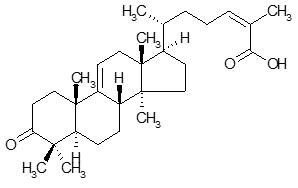 | Triterpenoids | 0.000026% | 3.8mg/14.5kg | stems of S. glaucescens | Natural Product Research (2019), 33, (3), 328-334 |
| **75** | lancifoic acid A | 879327-55-6 |  | Triterpenoids | 0.000068% | 9.8mg/14.5kg | stems of S. glaucescens | Natural Product Research (2019), 33, (3), 328-334 |
| **76** | schisandronic acid | 55511-14-3 |  | Triterpenoids | 0.000041% | 6.0mg/14.5kg | stems of S. glaucescens | Natural Product Research (2019), 33, (3), 328-335 |
| **77** | anwuweizonic acid | 117020-59-4 |  | Triterpenoids | 0.000059% | 8.6mg/14.5kg | stems of S. glaucescens | Natural Product Research (2019), 33, (3), 328-336 |
| **78** | manwuweizic acid | 116963-87-2 |  | Triterpenoids | 0.000279% | 40.5g/14.5kg | stems of S. glaucescens | Natural Product Research (2019), 33, (3), 328-338 |
| **79** | Kadsuric acid | 62393-88-8 | 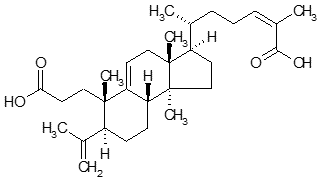 | Triterpenoids | 0.000368% | 25mg/6.8kg | leaves and stems of S. micrantha | Chemical & Pharmaceutical Bulletin (2003), 51, (10), 1174-1176 |
| **80** | Friedelanol | 5085-72-3 | 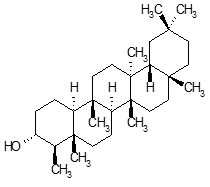 | Triterpenoids | 0.002000% | 60mg/3kg | *K. indica* | Chemistry of Natural Compounds (2018), 54, (3), 567-569 |
| **81** | friedelin | 559-74-0 |  | Triterpenoids | 0.001000% | 30mg/3kg | *K. indica* | Chemistry of Natural Compounds (2018), 54, (3), 567-569 |
| **82** | Cabraleadiol 3-acetate | 35833-62-6 | 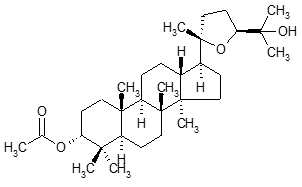 | Triterpenoids | 0.001680% | 8.4mg/500g | Roots of M. flexuosa | Natural Product Research (2013), 27, (22), 2118-2125 |
| **83** | 3β,20S-Dihydroxydammar-24-ene | 2220227-44-9 |  | Triterpenoids | 0.004640% | 23.2mg/500g | Roots of M. flexuosa | Natural Product Research (2013), 27, (22), 2118-2125 |
| **84** | Cabraleadiol | 67253-01-4 |  | Triterpenoids | 0.001680% | 8.4mg/500g | Roots of M. flexuosa | Natural Product Research (2013), 27, (22), 2118-2125 |
| **85** | Pre-schisanartanin B | 1033288-92-4 | 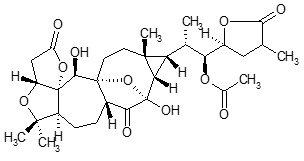 | Triterpenoids | 0.000746% | 10.3mg/1.38kg | dried fruits of S. arisanensis | J. Nat. Prod.2010,73,7,1228-1233 |
| **86** | Arisanlactone A | 1234185-96-6 |  | Triterpenoids | 0.001543% | 21.3mg/1.38kg | dried fruits of S. arisanensis | J. Nat. Prod.2010,73,7,1228-1234 |
| **87** | Arisanlactone B | 1234185-98-8 |  | Triterpenoids | 0.000717% | 9.9mg/1.38kg | dried fruits of S. arisanensis | J. Nat. Prod.2010,73,7,1228-1235 |
| **88** | Arisanlactone C | 1234185-99-9 |  | Triterpenoids | 0.001188% | 16.4mg/1.38kg | dried fruits of S. arisanensis | J. Nat. Prod.2010,73,7,1228-1236 |
| **89** | 2β-Hydroxyarisanlactone C | 1234186-00-5 |  | Triterpenoids | 0.000572% | 7.9mg/1.38kg | dried fruits of S. arisanensis | J. Nat. Prod.2010,73,7,1228-1237 |
| **90** | Schindilactone D | 1033288-84-4 |  | Triterpenoids | 0.001130% | 15.6mg/1.38kg | dried fruits of S. arisanensis | J. Nat. Prod.2010,73,7,1228-1238 |
| **91** | Tormentic acid | 13850-16-3 | 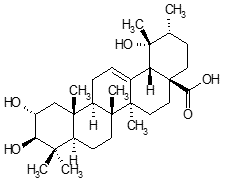 | Triterpenoids | 0.000573% | 6.3mg/1.1kg | leaves of *E. deflexa f. buisanensis* | Chemistry of Natural Compounds (2019), 55, (1), 74-78 |
| **92** | Lawsonin | 403501-43-9 |  | Triterpenoids | 0.000391% | 4.3mg/1.1kg | leaves of *E. deflexa f. buisanensis* | Chemistry of Natural Compounds (2019), 55, (1), 74-78 |
| **93** | methyl ursolate | 32208-45-0 |  | Triterpenoids | 0.000555% | 6.1mg/1,1kg | leaves of E. deflexa f. buisanensis | Chemistry of Natural Compounds (2019), 55, (1), 74-78 |
| **94** | Eichlerianic acid | 56421-13-7 | 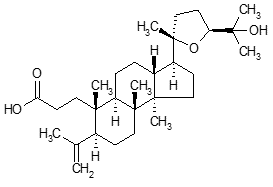 | Triterpenoids | 0.000300% | 15mg/5kg | roots of A. odorata | Natural Product Research (2016), 30, (4), 433-437 |
| **95** | shoreic acid | 21671-00-1 | 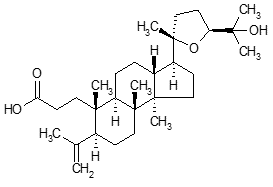 | Triterpenoids | 0.000180% | 9mg/5kg | roots of A. odorata | Natural Product Research (2016), 30, (4), 433-437 |
| **96** | eichlerialactone | 2202-01--9 |  | Triterpenoids | 0.000700% | 35mg/5kg | roots of A. odorata | Natural Product Research (2016), 30, (4), 433-437 |
| **97** | sapelins A | 26790-93-2 |  | Triterpenoids | 0.000140% | 7mg/5kg | roots of A. odorata | Natural Product Research (2016), 30, (4), 433-437 |
| **98** | isofouquierone | 100508-50-7 |  | Triterpenoids | 0.000260% | 13mg/5kg | roots of A. odorata | Natural Product Research (2016), 30, (4), 433-437 |
| **99** | Agladupol E | 952604-82-9 |  | Triterpenoids | 0.000620% | 31mg/5kg | roots of A. odorata | Natural Product Research (2016), 30, (4), 433-437 |
| **100** | 3-epimeliantriol | 57496-07-8 |  | Triterpenoids | 0.000200% | 10mg/5kg | roots of A. odorata | Natural Product Research (2016), 30, (4), 433-437 |
| **101** | 2β,3β-dihydroxy-5α-pregnane-16-one | 194033-59-5 |  | Triterpenoids | 0.000220% | 11mg/5kg | roots of A. odorata | Natural Product Research (2016), 30, (4), 433-437 |
| **102** | Micranoic acid A | 659738-08-6 | 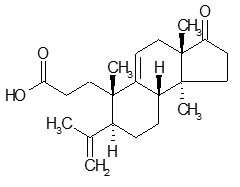 | Triterpenoids | 0.001368% | 93mg/6.8kg | leaves and stems of S. micrantha | Chemical & Pharmaceutical Bulletin (2003), 51, (10), 1174-1176 |
| **103** | Micranoic acid B | 659738-09-7 |  | Triterpenoids | 0.001074% | 73mg/6.8kg | leaves and stems of S. micrantha | Chemical & Pharmaceutical Bulletin (2003), 51, (10), 1174-1176 |
| **104** | Masticadienolic acid | 472-30-0 |  | Triterpenoids | 0.000603% | 41mg/6.8kg | leaves and stems of S. micrantha | Chemical & Pharmaceutical Bulletin (2003), 51, (10), 1174-1176 |
| **105** | Uncargenin C | 152243-70-4 | 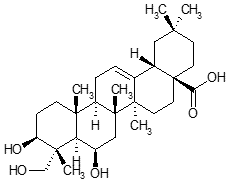 | Triterpenoids | 0.000147% | 3.8mg/2.59kg | roots of C. hystrix | Planta Medica (2013), 79, (14), 1356-1361 |
| **106** | (3β)​-​3,​19,​23,​24-​Tetrahydroxyurs-​12-​en-​28-​oic acid | 1033282-12-0 | 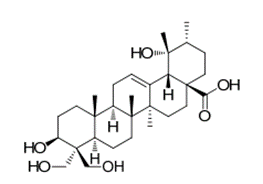 | Triterpenoids | 0.000286% | 7.4mg/2.59kg | roots of C. hystrix | Planta Medica (2013), 79, (14), 1356-1361 |
| **107** | Erubigenin | 173559-50-7 |  | Triterpenoids | 0.000112% | 2.9mg/2.59kg | roots of C. hystrix | Planta Medica (2013), 79, (14), 1356-1361 |
| **108** | (3β,​4α,​6β)​-​3,​6,​19,​23-​Tetrahydroxyurs-​12-​en-​28-​oic acid; 3β,​6β,​19α,​24-​Tetrahydroxyurs-​12-​en-​28-​oic acid | 91095-51-1 |  | Triterpenoids | 0.000463% | 12.0mg/2.59kg | roots of C. hystrix | Planta Medica (2013), 79, (14), 1356-1361 |
| **109** | Uncargenin C | 152243-70-4 |  | Triterpenoids | 0.000147% | 3.8mg/2.59kg | roots of C. hystrix | Planta Medica (2013), 79, (14), 1356-1361 |
| **110** | (3β,​4α,​6β,​19α)​-​3,​6,​19,​23-​Tetrahydroxyolean-​12-​en-​28-​oic acid; 3β,​6β,​19α,​23-​Tetrahydroxyolean-​12-​en-​29-​oic acid | 152243-71-6 |  | Triterpenoids | 0.000965% | 25mg/2.59kg | roots of C. hystrix | Planta Medica (2013), 79, (14), 1356-1361 |
| **111** | Limonexin | 99026-99-0 | 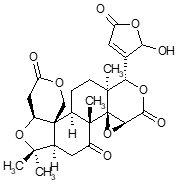 | Triterpenoids | 0.000197% | 5.1mg/2.59kg | roots of C. hystrix | Phytochemistry (Elsevier) (2013), 88, 79-84 |
| **112** | δ-Amyrone | 20248-08-2 | 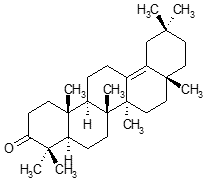 | Triterpenoids | 0.001439% | 1007mg/70kg | fresh whole Sedum sarmentosum Bunge | Phytochemistry (1998), 49, (8), 2607-2610. |
| **113** | Richenoic acid | 134476-74-7 |  | Triterpenoids | 0.000080% | 10mg/12.5kg | dried bark of D. hainanense | A Journal of Chemical Sciences (2010), 65, (9), 1161-1163 |
| **114** | 4,​23,​30-​Trihydroxy-​3,​4-​secoolean-​9,​12-​dien-​3-​oic acid | 2241558-52-9 |  | Triterpenoids | 0.000056% | 7mg/12.5kg | dried bark of D. hainanense | A Journal of Chemical Sciences (2010), 65, (9), 1161-1163 |
| **115** | Richenoic acid | 134476-74-7 | 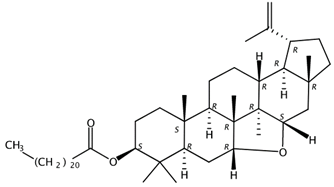 | Triterpenoids | 0.000048% | 6mg/12.5kg | dried bark of D. hainanense | A Journal of Chemical Sciences (2010), 65, (9), 1161-1163 |
| **116** | Friedelin | 559-74-0 | 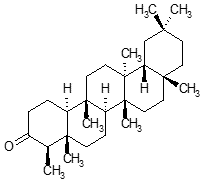 | Triterpenoids | 0.001061% | 35mg/3.3kg | *leaves of Globimetula braunii.* | Nat Prod Res. 2019 Mar 31:1-8 |
| **117** | globrauneine A | 825631-17-2 | 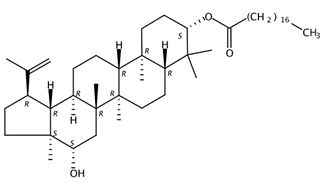 | Triterpenoids | 0.003273% | 108mg/3.3kg | *leaves of Globimetula braunii.* | Nat Prod Res. 2019 Mar 31:1-8 |
| **118** | globrauneine B | 848093-37-8 | 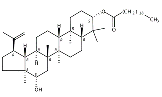 | Triterpenoids | 0.000197% | 6.5mg/3.3kg | *leaves of Globimetula braunii.* | Nat Prod Res. 2019 Mar 31:1-8 |
| **119** | globrauneine D | 2241087-60-3 | 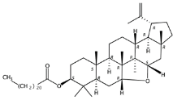 | Triterpenoids | 0.004818% | 159mg/3.3kg | *leaves of Globimetula braunii.* | Nat Prod Res. 2019 Mar 31:1-8 |
| **120** | Globimetulin A | 2241087-60-3 | 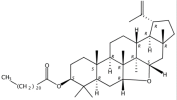 | Triterpenoids | 0.000242% | 8mg/3.3kg | *leaves of Globimetula braunii.* | Nat Prod Res. 2019 Mar 31:1-8 |
| **121** | globrauneine F | 2241087-60-3 | 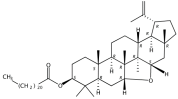 | Triterpenoids | 0.003136% | 103.5mg/3.3kg | *leaves of Globimetula braunii.* | Nat Prod Res. 2019 Mar 31:1-8 |
| **122** | lupeol | 545-47-1 |  | Triterpenoids | 0.004348% | 143.5mg/3.3kg | *leaves of Globimetula braunii.* | Nat Prod Res. 2019 Mar 31:1-8 |
| **123** | lupeol palmitate | 32214-80-5 |  | Triterpenoids | 0.006152% | 203mg/3.3kg | *leaves of Globimetula braunii.* | Nat Prod Res. 2019 Mar 31:1-8 |
| **124** | lup-20,29-en-3β,15α-diol | 913745-73-0 |  | Triterpenoids | 0.004333% | 143mg/3.3kg | *leaves of Globimetula braunii.* | Nat Prod Res. 2019 Mar 31:1-8 |
| **125** | β-sitosterol | 83-46-5 |  | Triterpenoids | 0.002303% | 76mg/3.3kg 0.17% | *leaves of Globimetula braunii.* | Nat Prod Res. 2019 Mar 31:1-8 |
| **126** | Aglinin A | 246868-97-3 | 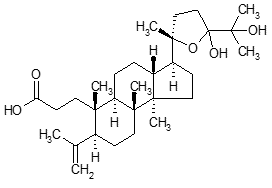 | Triterpenoids | 0.251667% | 151mg/60g | dried pericarp of A. erythrosperma | [Nat Prod Res. 2011 Oct;25(17):1621-11](https://www.ncbi.nlm.nih.gov/pubmed/?term=Biologically+active+constituents+of+Aglaia+erythrosperma) |
| **127** | Serratenediol diacetate | 2239-24-9 | 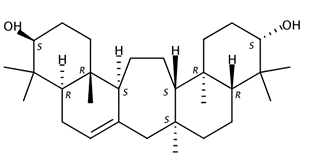 | Triterpenoids | 0.003023% | 130mg/4.3kg | whole plants of H. miyoshianu w | Chinese Journal of Chemistry (2003), 21, (10), 1364-1368 |
| **128** | Miyoshianol C | 667457-68-3 | 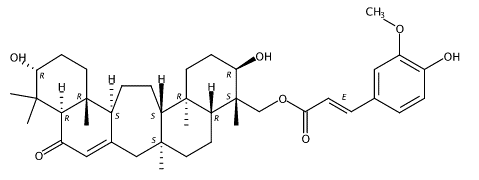 | Triterpenoids | 0.000140% | 6mg/4.3kg | whole plants of H. miyoshianu w | Chinese Journal of Chemistry (2003), 21, (10), 1364-1368 |
| **129** | Cabraleadiol | 67253-01-4 |  | Triterpenoids | 0.019250% | 77mg/400g | dried leave of A. erythrosperma | Nat Prod Res. 2011 Oct;25(17):1621-8 |
| **130** | 13(18)-Oleanen-3-ol | 508-04-3 |  | Triterpenoids | 0.000857% | 600mg/70kg | fresh whole Sedum sarmentosum Bunge | Phytochemistry (1998), 49, (8), 2607-2610. |
| **131** | δ- amyrin | 508-04-3 |  | Triterpenoids | 0.000157% | 110mg/70kg | fresh whole Sedum sarmentosum Bunge | Phytochemistry (1998), 49, (8), 2607-2610. |
| **132** | Sarmentolin | 220857-14-7 |  | Triterpenoids | 0.000071% | 50mg/70kg | fresh whole Sedum sarmentosum Bunge | Phytochemistry (1998), 49, (8), 2607-2610. |
| **133** | Cabraleahydroxylactone | 35833-69-3 | 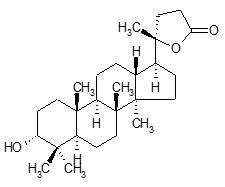 | Triterpenoids | 0.004750% | 19mg /400g | dried leave of A. erythrosperma | Nat Prod Res. 2011 Oct;25(17):1621-8 |
| **134** | Eichlerialactone | 2202-01-9 | 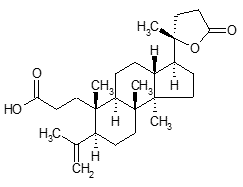 | Triterpenoids | 0.021500% | 86mg/400g | dried leave of A. erythrosperma | Nat Prod Res. 2011 Oct;25(17):1621-8 |
| **135** | Cabraleadiol | 67253-01-4 |  | Triterpenoids | 0.019250% | 77mg/400g | dried leave of A. erythrosperma | Nat Prod Res. 2011 Oct;25(17):1621-8 |
| **136** | 5,6-desmethylenedioxy-5-methoxy-aglalactone | 922169-96-8 |  | Triterpenoids | 0.025500% | 102mg/400g | dried leave of A. erythrosperma | [Nat Prod Res. 2011 Oct;25(17):1621-10](https://www.ncbi.nlm.nih.gov/pubmed/?term=Biologically+active+constituents+of+Aglaia+erythrosperma) |
| **137** | Eichlerianic acid | 56421-13-7 |  | Triterpenoids | 0.008333% | 5mg/60g | dried pericarp of A. erythrosperma | [Nat Prod Res. 2011 Oct;25(17):1621-12](https://www.ncbi.nlm.nih.gov/pubmed/?term=Biologically+active+constituents+of+Aglaia+erythrosperma) |
| **138** | cabralealactone | 19865-87-3 |  | Triterpenoids | 0.038500% | 77mg/200g | dried seeds of A. erythrosperma | [Nat Prod Res. 2011 Oct;25(17):1621-13](https://www.ncbi.nlm.nih.gov/pubmed/?term=Biologically+active+constituents+of+Aglaia+erythrosperma) |
| **139** | Taraxasterol acetate | 6426-43-3 | 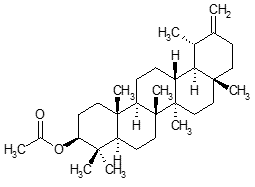 | Triterpenoids | 0.002231% | 11mg/493g | *Dried roots of E. heterophylla* | Nat Prod Res. 2018 Apr 9:1-4 |
| **140** | β-Sitosterol | 83-46-5 |  | Triterpenoids | 0.003164% | 15.6/493 | *Dried roots of E. heterophylla* | Nat Prod Res. 2018 Apr 9:1-4 |
| **141** | Lupeol acetate | 1617-68-1 |  | Triterpenoids | 0.005477% | 27mg/493g | *Dried roots of E. heterophylla* | Nat Prod Res. 2018 Apr 9:1-4 |
| **142** | Taraxasterol acetate | 6426-43-3 |  | Triterpenoids | 0.002231% | 11mg/493g | *Dried roots of E. heterophylla* | Nat Prod Res. 2018 Apr 9:1-4 |
| **143** | Pseudo-taraxasterol acetate | 42482-72-4 |  | Triterpenoids | 0.002231% | 11mg/493g | *Dried roots of E. heterophylla* | *Dried roots of E. heterophylla* |
| **144** | Germanicol acetate | 10483-91-7 |  | Triterpenoids | 0.004462% | 22mg/493 | *Dried roots of E. heterophylla* | *Dried roots of E. heterophylla* |
| **145** | Germanicol hexanoate | 863287-55-2 |  | Triterpenoids | 0.005882% | 29mg/493g | *Dried roots of E. heterophylla* | *Dried roots of E. heterophylla* |
| **146** | Cycloart-25-ene-3β,24-diol | 10388-48-4 | 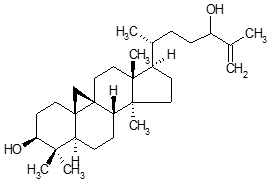 | Triterpenoids | 0.000750% | 60mg/8kg | *Euphorbia altotibetic* | Helvetica Chimica Acta (2003), 86, (7), 2525-2532 |
| **147** | Cycloartane-3,24,25-triol 24,25-acetonide | 57576-31-5 | 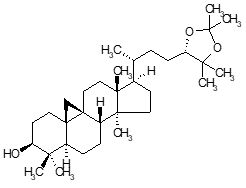 | Triterpenoids | 0.000063% | 5mg/8kg | *Leaves of Lyonia ovalifilia* | Chemical & Pharmaceutical Bulletin (1975), 23, (2), 460-2 |
| **148** | δ-Amyrin acetate | 51361-60-5 |  | Triterpenoids | 0.000250% | 25mg/10kg | *roots of Vemonia cinerea Less* | Journal of Natural Products, 47, 368–372. |
| **149** | β-Amyrin | 559-70-6 |  | Triterpenoids | 0.000250% | 25mg/10kg | *roots of Vemonia cinerea Less* | Journal of Natural Products, 47, 368–372. |
| **150** | β-amyrin acetate | 1616-93-9 |  | Triterpenoids | 0.000400% | 40mg/10kg | *roots of Vemonia cinerea Less* | Journal of Natural Products, 47, 368–372. |
| **151** | 3β-acetoxyurs-13 (18) ene | 90745-23-6 |  | Triterpenoids | 0.001500% | 150mg/10kg | *roots of Vemonia cinerea Less* | Journal of Natural Products, 47, 368–372. |
| **152** | Phyllanthone | 1036382-95-2 |  | Triterpenoids | 0.000960% | 10.6mg/1104g | *stem bark and leaves of P.polyanthu* | Phytochemistry Letters (2008), 1, (1), 11-17 |
| **153** | Phyllanthol | 546-51-0 |  | Triterpenoids | 0.001359% | 15mg/1104g | stem bark and leaves of P. polyanthu | Phytochemistry Letters (2008), 1, (1), 11-17 |
| **154** | (20S)-3β-Acetoxy-24-methylenedammaran-20-ol | 77518-12-8 |  | Triterpenoids | 0.000562% | 6.2mg/1104g | stem bark (754 g) and leaves(350g）of P. polyanthu | Phytochemistry Letters (2008), 1, (1), 11-17 |
| **155** | (20S)-3α-Acetoxy-24-methylenedammaran-20-ol | 1206897-30-4 |  | Triterpenoids | 0.000697% | 7.7mg/1104g | stem bark and leaves of P. polyanthu | Phytochemistry Letters (2008), 1, (1), 11-17 |
| **156** | friedelin | 559-74-0 | 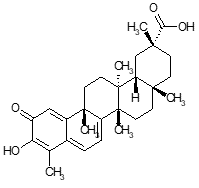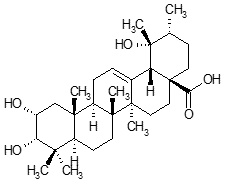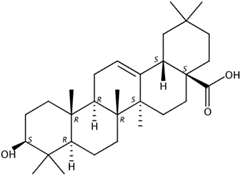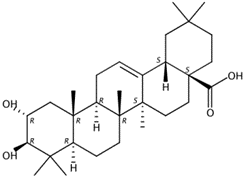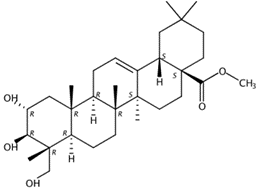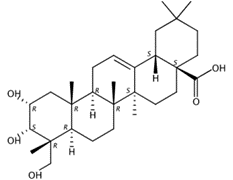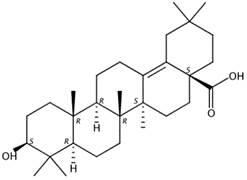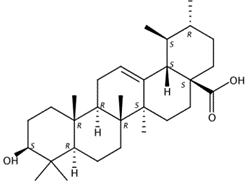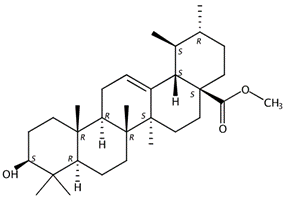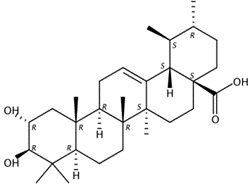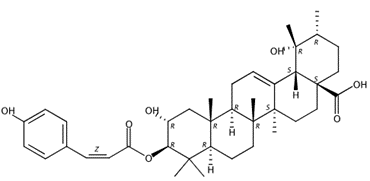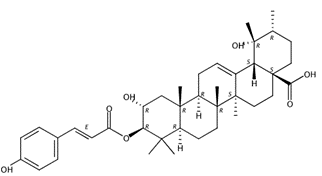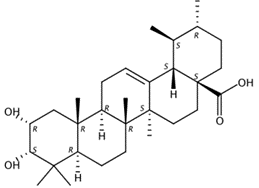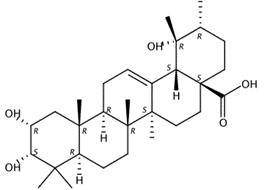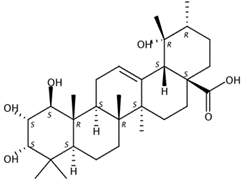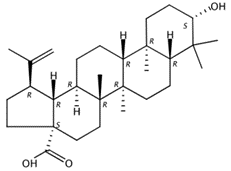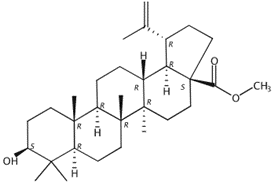 | Triterpenoids | 0.012044% | 108.4mg/900g | air-dried roots of S. impressifolia | Chemistry of Natural Compounds (2018), 54(1), 200-201 |
| **157** | β -sitosterol | 83-46-5 |  | Triterpenoids | 0.000700% | 6.3mg/900g | air-dried roots of S. impressifolia | Chemistry of Natural Compounds (2018), 54(1), 200-201 |
| **158** | quinovic acid | 465-74-7 |  | Triterpenoids | 0.000367% | 3.3mg/900g | air-dried roots of S. impressifolia | Chemistry of Natural Compounds (2018), 54(1), 200-201 |
| **159** | cincholic acid | 107160-24-7 |  | Triterpenoids | 0.000322% | 2.9mg/900g | air-dried roots of S. impressifolia | Chemistry of Natural Compounds (2018), 54(1), 200-201 |
| **160** | cincholic-3-O-6-deoxy--D-glucopyranoside acid | 111665-41-9 |  | Triterpenoids | 0.000211% | 1.9mg/900g | air-dried roots of S. impressifolia | Chemistry of Natural Compounds (2018), 54(1), 200-201 |
| **161** | quinovic-3-O--D-glucopyranoside acid | 167882-67-9 |  | Triterpenoids | 0.000167% | 1.5mg/900g | air-dried roots of S. impressifolia | Chemistry of Natural Compounds (2018), 54(1), 200-201 |
| **162** | celastrol | 34157-83-0 |  | Triterpenoids | 0.000311% | 2.8mg/900g | air-dried roots of S. impressifolia | Chemistry of Natural Compounds (2018), 54(1), 200-201 |
| **163** | Euscaphic acid | 53155-25-2 |  | Triterpenoids | 0.000176 | 440mg/2.5kg | Rosa sterilis S.D. Shi (Rosaceae) | Journal of Natural Products Voi. 52, No. 1, pp. 162-166, Jan-Feb 1989 |
| **164** | oleanolic acid | 508-02-1 |  | Triterpenoids | 0.062910% | 629.1mg/1kg | leaves of Eriobotrya japonica | Biol. Pharm. Bull. 28(10) 1995—1999 (2005) |
| **165** | maslinic acid | 4373-41-5 |  | Triterpenoids | 0.039610% | 396.1mg/1kg | leaves of Eriobotrya japonica | Biol. Pharm. Bull. 28(10) 1995—1999 (2005) |
| **166** | methyl arjunolate | 22452-82-0 |  | Triterpenoids | 0.004610% | 46.6mg/1kg | leaves of Eriobotrya japonica | Biol. Pharm. Bull. 28(10) 1995—1999 (2005) |
| **167** | 2α,3α,23-trihydroxyolean-12-en-28-oic acid | 102519-34-6 |  | Triterpenoids | 0.030200% | 302mg/1kg | leaves of Eriobotrya japonica | Biol. Pharm. Bull. 28(10) 1995—1999 (2005) |
| **168** | δ-oleanolic acid | 6713-26-4 |  | Triterpenoids | 0.023300% | 233mg/1kg | leaves of Eriobotrya japonica | Biol. Pharm. Bull. 28(10) 1995—1999 (2005) |
| **169** | ursolic acid | 77-52-1 |  | Triterpenoids | 0.226010% | 2260.1mg/1kg | leaves of Eriobotrya japonica | Biol. Pharm. Bull. 28(10) 1995—1999 (2005) |
| **170** | methyl ursolate | 32208-45-0 |  | Triterpenoids | 0.004660% | 46.6mg/1kg | leaves of Eriobotrya japonica | Biol. Pharm. Bull. 28(10) 1995—1999 (2005) |
| **171** | corosolic acid | 4547-24-4 |  | Triterpenoids | 0.102520% | 1025.2mg/1kg | leaves of Eriobotrya japonica | Biol. Pharm. Bull. 28(10) 1995—1999 (2005) |
| **172** | 3-O-cis-p-coumaroyltormentic acid | 121072-40-0 |  | Triterpenoids | 0.018640% | 186.4mg/1kg | leaves of Eriobotrya japonica | Biol. Pharm. Bull. 28(10) 1995—1999 (2005) |
| **173** | 3-O-trans-p-coumaroyltormentic acid | 121064-78-6 |  | Triterpenoids | 0.093200% | 932mg/1kg | leaves of Eriobotrya japonica | Biol. Pharm. Bull. 28(10) 1995—1999 (2005) |
| **174** | 3-epicorosolic acid | 52213-27-1 |  | Triterpenoids | 0.004660% | 46.6mg/1kg | leaves of Eriobotrya japonica | Biol. Pharm. Bull. 28(10) 1995—1999 (2005) |
| **175** | euscaphic acid | 53155-25-2 |  | Triterpenoids | 0.114170% | 1141.7mg/1kg | leaves of Eriobotrya japonica | Biol. Pharm. Bull. 28(10) 1995—1999 (2005) |
| **176** | 1β-hydroxyeuscaphic acid | 120211-98-5 |  | Triterpenoids | 0.067570% | 675.7mg/1kg | leaves of Eriobotrya japonica | Biol. Pharm. Bull. 28(10) 1995—1999 (2005) |
| **177** | betulinic acid | 472-15-1 |  | Triterpenoids | 0.002330% | 23.3mg/1kg | leaves of Eriobotrya japonica | Biol. Pharm. Bull. 28(10) 1995—1999 (2005) |
| **178** | methyl betulinate | ,2259-06-5 |  | Triterpenoids | 0.002330% | 23.3mg/1kg | leaves of Eriobotrya japonica | Biol. Pharm. Bull. 28(10) 1995—1999 (2005) |
| **179** | Euscaphic Acid A | 1252662-04-6 |  | Triterpenoids | 0.000086% | 6.5mg/7.6kg | Twigs of Euscaphis japonica | J. Nat. Prod. 2010, 73, 1655–1658 |
| **180** | Euscaphic Acid B | 1252662-06-8 |  | Triterpenoids | 0.000091% | 6.9mg/7.6kg | Twigs of Euscaphis japonica | J. Nat. Prod. 2010, 73, 1655–1658 |
| **181** | Euscaphic Acid C | 1252662-08-0 |  | Triterpenoids | 0.000174% | 13.2mg/7.6kg | Twigs of Euscaphis japonica | J. Nat. Prod. 2010, 73, 1655–1658 |
| **182** | Euscaphic Acid D | 1252662-10-4 |  | Triterpenoids | 0.000463% | 35.2mg/7.6kg | Twigs of Euscaphis japonica | J. Nat. Prod. 2010, 73, 1655–1658 |
| **183** | Euscaphic Acid E | 1252662-11-5 |  | Triterpenoids | 0.000084% | 6.4mg/7.6kg | Twigs of Euscaphis japonica | J. Nat. Prod. 2010, 73, 1655–1658 |
| **184** | Euscaphic Acid F | 1252662-12-6 |  | Triterpenoids | 0.000088% | 6.7mg.7.6kg | Twigs of Euscaphis japonica | J. Nat. Prod. 2010, 73, 1655–1658 |
| **185** | euscaphic acid | 53155-25-2 |  | Triterpenoids | 0.000395% | 30mg/7.6kg | Twigs of Euscaphis japonica | J. Nat. Prod. 2010, 73, 1655–1658 |
| **186** | tormentic acid | 13850-16-3 |  | Triterpenoids | 0.000075% | 5.7mg/7.6kg | Twigs of Euscaphis japonica | J. Nat. Prod. 2010, 73, 1655–1658 |
| **187** | pomolic acid | 13849-91-7 |  | Triterpenoids | 0.000289% | 22mg/7.6kg | Twigs of Euscaphis japonica | J. Nat. Prod. 2010, 73, 1655–1658 |
| **188** | rotundic acid | 20137-37-5 |  | Triterpenoids | 0.000071% | 5.4mg/7.6kg | Twigs of Euscaphis japonica | J. Nat. Prod. 2010, 73, 1655–1658 |
| **189** | rotungenic acid | 121467-43-4 |  | Triterpenoids | 0.000295% | 22.4mg/7.6kg | Twigs of Euscaphis japonica | J. Nat. Prod. 2010, 73, 1655–1658 |
| **190** | 2α-hydroxyursolic acid | 4547-24-4 |  | Triterpenoids | 0.102520% | 1025.2mg/1kg | Twigs of Euscaphis japonica | J. Nat. Prod. 2010, 73, 1655–1658 |
| **191** | Quinovin | 107870-05-3 |  | Triterpenoids | 0.003100% | 15.5mg/500g | aerial parts of Z. aegytpium | Magn Reson Chem. 2016 Sep;54(9) ;771-773 |
| **192** | quinovic acid | 465-74-7 |  | Triterpenoids | 0.000143% | 4.3g/3kg | The dried, pulverized bark of Mitragyna stipulosa | Arch Pharm Res Vol 25, No 3, 270-274, 2002 |
| **193** | β-sitosterol β-D-glucopyranoside | 474-58-8 |  | Triterpenoids | 0.013333% | 400mg/3kg | The dried, pulverized bark of Mitragyna stipulosa | Arch Pharm Res Vol 25, No 3, 270-274, 2002 |
| **194** | Quinovic acid 3-O-β-D-glucopyranoside | 79955-41-2 |  | Triterpenoids | 0.026667% | 800mg/3kg | The dried, pulverized bark of Mitragyna stipulosa | Arch Pharm Res Vol 25, No 3, 270-274, 2002 |
| **195** | zygophyloside B | 133084-07-8 |  | Triterpenoids | 0.023333% | 700mg/3kg | The dried, pulverized bark of Mitragyna stipulosa | Arch Pharm Res Vol 25, No 3, 270-274, 2002 |
| **196** | quinovic acid | 465-74-7 |  | Triterpenoids | 0.000143% | 4.3g/3kg | The dried, pulverized bark of Mitragyna stipulosa | Arch Pharm Res Vol 25, No 3, 270-274, 2002 |
| **197** | 24,25-Epoxydammar-20(21)-en-3-one | 63543-52-2 |  | Triterpenoids | 0.013408% | 7.2mg/53.7g | leaves of A. odorata | Phytochemistry (Elsevier) (2012), 76, 83-91 |
| **198** | 2α,24-Dihydroxyursolic acid | 143839-02-5 |  | Triterpenoids | 0.000164% | 8.2mg/5kg | aerial parts of Isodon excisoides | Journal of Asian Natural Products Research (2013), 15(9), 962-968. |
| **199** | Dammar-20(21)-ene-3β,24,25-triol | 55050-69-6 |  | Triterpenoids | 0.009311% | 5mg/53.7g | leaves of A. odorata | Phytochemistry (Elsevier) (2012), 76, 83-91 |
| **200** | Pseudotaraxasterol | 464-98-2 |  | Triterpenoids | 0.000400% | 12mg/3kg | Ixeridium gracile (DC.) Shih | Chemistry of Natural Compounds, Vol. 44, No. 3, 2008 |
| **201** | β-Amyrinβ香树脂醇 | 559-70-6 |  | Triterpenoids | 0.000333% | 10mg/3kg | Ixeridium gracile (DC.) Shih | Chemistry of Natural Compounds, Vol. 44, No. 3, 2008 |
| **202** | Taraxasterol | 1059-14-9 |  | Triterpenoids | 0.000300% | 9mg/3kg | Ixeridium gracile (DC.) Shih | Chemistry of Natural Compounds, Vol. 44, No. 3, 2008 |
| **203** | Evodol | 22318-10-1 |  | Triterpenoids | 0.000063% | 3.2mg/5.1kg | Tetradium ruticarpum | CHEMISTRY & BIODIVERSITY – Vol. 7 (2010) |
| **204** | Lupeol | 545-47-1 |  | Triterpenoids | 0.000500% | 15mg/3kg | Ixeridium gracile (DC.) Shih | Chemistry of Natural Compounds, Vol. 44, No. 3, 2008 |
| **205** | Glaucin B | 115458-73-6 |  | Triterpenoids | 0.000700% | 7mg/1kg | Evodia olauca Mlq. | Phytochemtstry, Vol 27, No 5, pp 1429 1432, 1988 |
| **206** | Taraxerone | 514-07-8 |  | Triterpenoids | 0.000133% | 6mg/4.5kg | V. trifolia var. simplicifolia | Chemistry of Natural Compounds, Vol. 52, No. 4, July, 2016 |
| **207** | Limonol | 989-61-7 |  | Triterpenoids |  | 2mg/580g | leaves of C. odorata | [Chemistry. 2016 Sep 5;22(37):13236-50](https://www.ncbi.nlm.nih.gov/pubmed/?term=Identification+of+Limonol+Derivatives+as+Heat+Shock+Protein+90+(Hsp90)+Inhibitors+through+a+Multidisciplinary+Approach" \o "Chemistry (Weinheim an der Bergstrasse, Germany).) |
| **208** | limonin | 1180-71-8 |  | Triterpenoids | 0.000500% | 20mg/4kg | Euodia daniellii leaves and fruits | Arch Pharm Res Vol 25, No 6, 824-830, 2002 |
| **209** | Bauerenol acetate | 17020-04-1 |  | Triterpenoids | 0.000833% | 25mg/3kg | Euphorbia chrysocoma | Journal of Asian Natural Products Research, Vol. 7, No. 6, December 2005, 857–860 |
| **210** | α-amyrenone | 638-96-0 |  | Triterpenoids | 0.000500% | 10mg/2kg | Dried roots of Euphorbia chrysocoma | Journal of Asian Natural Products Research, Vol. 7, No. 6, December 2005, 857–860 |
| **211** | β-sitosterol | 83-46-5 |  | Triterpenoids | 0.002650% | 53mg/2kg | Dried roots of Euphorbia chrysocoma | Journal of Asian Natural Products Research, Vol. 7, No. 6, December 2005, 857–860 |
| **212** | stigmasterol | 83-48-7 |  | Triterpenoids | 0.002650% | 53mg/2kg | Dried roots of Euphorbia chrysocoma | Journal of Asian Natural Products Research, Vol. 7, No. 6, December 2005, 857–860 |
| **213** | betulinic acid | 472-15-1 |  | Triterpenoids | 0.001600% | 32mg/2kg | Dried roots of Euphorbia chrysocoma | Journal of Asian Natural Products Research, Vol. 7, No. 6, December 2005, 857–860 |
| **214** | 3β, 23-dihydroxy-urs-12-en-28-oic acid | 120092-82-2 |  | Triterpenoids | 0.000039% | 7mg/18kg | Air-dried leaves of Isodon xerophilus | Chinese Journal of Natural Medicines 2012, 10(4): 0307-0310 |
| **215** | arjunolic acid | 465-00-9 |  | Triterpenoids | 0.000111% | 20mg/18kg | Air-dried leaves of Isodon xerophilus | Chinese Journal of Natural Medicines 2012, 10(4): 0307-0310 |
| **216** | 2α,3α,24-Trihydroxyolean-12-en-28-oic acid | 150821-16-2 |  | Triterpenoids | 0.000250% | 25mg/10kg | Actinidia chinensis PLANCH. | Chem. Biodiversity 2018, 15, e1700454 |
| **217** | 2α,3α,24-Trihydroxyurs-12-en-28-oic acid | 89786-83-4 |  | Triterpenoids | 0.000280% | 28mg/10kg | Actinidia chinensis PLANCH. | Chem. Biodiversity 2018, 15, e1700454 |
| **218** | Lup-20(29)-ene-3α,23-diol | 32451-85-7 |  | Triterpenoids | 0.001440% | 36mg/2.5kg | Glochidion sphaerogynum Kurz. | Planta Med 2005;71: 208- 213 |
| **219** | Esculentic acid | 103974-74-9 |  | Triterpenoids | 0.000100% | 6.7mg/6.7kg | Weigela subsessilis | Biol. Pharm. Bull. 29(4) 830—833 (2006) |
| **220** | Oleanolic acid | 508-02-1 |  | Triterpenoids | 0.084140% | 169.7-841.4mg/kg olives | Radermachera boniana twigs and leaves | J. Nat. Prod. 2011, 74, 1318–1322 doi.org/10.1021/np200022b |
| **221** | 24,25-Dihydroxydammar-20-en-3-one | 63543-53-3 |  | Triterpenoids | 0.000101% | 10.1mg/10kg | Ailanthus altissima | Phytochemistry 86 (2013) 159–167 |
| **222** | Maslinic acid | 4373-41-5 |  | Triterpenoids | 0.131840% | 287.1-1318.4mg/kg | olives | C. Romero et al. / Food Chemistry 118 (2010) 670–674 |
| **223** | β-Amyrin palmitate | 5973-06-8 |  | Triterpenoids | 0.002000% | 80mg/4kg | Wrightia tomentosa | Phytomedicine 19 (2012) 682–685 |
| **224** | 3-Epicorosolic acid | 52213-27-1 |  | Triterpenoids | 0.003333% | 3.7mg/111g | Perilla frutescens | Biosci. Biotechnol. Biochem.,68(1), 85-90,2004 |
| **225** | Glochidiol | 6610-56-6 |  | Triterpenoids | 0.002000% | 50mg/2.5kg | Glochidion sphaerogynum Kurz. | Planta Med 2005;71: 208- 213 |
| **226** | Actein | 18642-44-9 |  | Triterpenoids | 0.001500% | 300mg/20kg | rhizomes Cimicifuga foetida | Chem. Pharm. Bull. 60(5) 571–577 (2012) |
| **227** | Erythrodiol 3-palmitate | 19833-13-7 |  | Triterpenoids | 0.000159% | 10mg/6.3kg | Tagetes erecta | Chemistry of Natural Compounds, Vol. 47, No. 2, May, 2011 |
| **228** | Betulin palmitate | 582315-55-7 |  | Triterpenoids | 0.000167% | 10mg/6kg | stem of S. affinis | J. Nat. Prod. 2012, 75, 1160−1166 |
| **229** | Lupenone | 1617-70-5 |  | Triterpenoids | 0.000324% | 12.3mg/3.8kg | Zanthoxylum tessmannii | Chem. Biodiversity 2019, 16, e1800590 |
| **230** | Coronalolide methyl ester | 268214-50-2 |  | Triterpenoids | 1.879497% | 1.75g/93.11g | Gardenia sootepensis | Journal of Natural Products, 2009, Vol. 72, No. 6 |
| **231** | Cimiracemoside C | 256925-92-5 |  | Triterpenoids | 0.003300% | 33mg/1kg | Cimicifuga racemosa | Journal of Natural Products, 2000, Vol. 63, No. 7 |
| **232** | Bryonolic acid | 24480-45-3 |  | Triterpenoids | 0.033333% | 500mg/1.5kg | Bryonia aspera | Planta Med 2010; 76: 1014–1018 |
| **233** | 26-O-Acetylsootepin A | 1772588-99-4 |  | Triterpenoids | 0.003125% | 500mg/1.6kg | Bryonia aspera | Planta Med 2010; 76: 1014–1018 |
| **234** | Cabraleone | 35761-54-7 |  | Triterpenoids | 0.010000% | 50mg/500g | Dysoxylum mafabaricum | MAGNETIC RESONANCE IN CHEMISTRY, VOL. 34, 146-150 (1996) |
| **235** | Dipterocarpol | 471-69-2 |  | Triterpenoids | 1.60% | 1.60% | Pistacia lentiscus L. (Anacardiaceae) | Phytochemistry, Vol. 30, No. 11, pp. 3709-3712, 1991 |
| **236** | 2-Hydroxydiplopterol | 1193250-54-2 |  | Triterpenoids | 0.000250% | 10mg/4kg | Arisaema jacquemonti | Journal of Medicinal Plants Research,Vol. 7(27), pp. 2040-2042, 17 July, 2013 DOI: 10.5897/JMPR12.1258 |
| **237** | Cimigenoside | 27994-11-2 |  | Triterpenoids | 0.000353% | 5.3mg/1.5kg | rhizomes of S. vaginata (Maxim.) Franch | Journal of Asian Natural Products Research (2017), 19(12), 1177-1182. |
| **238** | 1-Dehydroxy-23-deoxojessic acid | 149252-87-9 |  | Triterpenoids | 0.022000% | 1.10g/5kg | leaves and twigs of *Gardenia thailandica* | Planta Medica (2004), 70(4), 366-370. |
| **239** | uncaric acid | 123135-05-7 |  | Teterpenoid | 0.000089% | 8mg/9kg | *Hooks and stems of Uucaria sessilifructus* | Molecules 2013, 18, 9727-9734 |
| **240** | Scutellaric acid | 102919-76-6 |  | Triterpenoids | 0.000046% | 2.3mg/5kg | dried leaves of D. kaki | Journal of Natural Products (2008), 71(10), 1775-1778 |
| **241** | Coronalolic acid | 268214-52-4 |  | Triterpenoids | 0.027143% | 9.5mg/35g | GARDENIA CORONARIA | Tmzhrdron. Vol. 53. No. 2, pp. 529-538, 1997 |
| **242** | Ganoderiol F | 114567-47-4 |  | Triterpenoids | 0.000720% | 36mg/5kg | fruiting body of Ganoderma lucidum | Phytochemistry Vol. 49, No. 6, pp. 1651-1657, 1998 |
| **243** | Ganoderic acid a | 81907-62-2 |  | Triterpenoids | 0.000800% | 40mg/5kg | fruiting body of Ganoderma lucidum | Phytochemistry Vol. 49, No. 6, pp. 1651-1657, 1998 |
| **244** | Ganoderic acid A | 81907-62-2 |  | Triterpenoids | 0.000860% | 43mg/5kg | fruiting body of Ganoderma lucidum | Phytochemistry Vol. 49, No. 6, pp. 1651-1657, 1998 |
| **245** | Ganoderic acid B | 81907-61-1 |  | Triterpenoids | 0.004700% | 47mg/5kg | fruiting body of Ganoderma lucidum | Phytochemistry Vol. 49, No. 6, pp. 1651-1657, 1998 |
| **246** | Ganoderic acid C | 95311-97-0 |  | Triterpenoids | 0.000160% | 8mg/5kg | fruiting body of Ganoderma lucidum | Phytochemistry Vol. 49, No. 6, pp. 1651-1657, 1998 |
| **247** | Ganoderic acid H | 98665-19-1 |  | Triterpenoids | 0.000180% | 9mg/5kg | fruiting body of Ganoderma lucidum | Phytochemistry Vol. 49, No. 6, pp. 1651-1657, 1998 |
| **248** | Ganoderiol A | 106518-61-0 |  | Triterpenoids | 0.000120% | 6mg/5kg | fruiting body of Ganoderma lucidum | Phytochemistry Vol. 49, No. 6, pp. 1651-1657, 1998 |
| **249** | Ganoderiol B | 106518-62-1 |  | Triterpenoids | 0.000300% | 15mg/5kg | fruiting body of Ganoderma lucidum | Phytochemistry Vol. 49, No. 6, pp. 1651-1657, 1998 |
| **250** | Ganodermanontriol | 106518-63-2 |  | Triterpenoids | 0.000320% | 16mg/5kg | fruiting body of Ganoderma lucidum | Phytochemistry Vol. 49, No. 6, pp. 1651-1657, 1998 |
| **251** | Ergosterol | 57-87-4 |  | Triterpenoids | 0.007300% | 365mg/5kg | fruiting body of Ganoderma lucidum | Phytochemistry Vol. 49, No. 6, pp. 1651-1657, 1998 |
| **252** | Ergosterol peroxide | 2061-64-5 |  | Triterpenoids | 0.001240% | 62mg/5kg | fruiting body of Ganoderma lucidum | Phytochemistry Vol. 49, No. 6, pp. 1651-1657, 1998 |
| **253** | Cerevisterol | 516-37-0 |  | Triterpenoids | 0.000280% | 14mg/5kg | fruiting body of Ganoderma lucidum | Phytochemistry Vol. 49, No. 6, pp. 1651-1657, 1998 |
| **254** | Coronalolide | 268214-51-3 |  | Triterpenoids | 0.015036% | 14mg/93.11g | Gardenia sootepensis | Journal of Natural Products, 2009, Vol. 72, No. 6 |
| **255** | Ganodermanontriol | 106518-63-2 |  | Triterpenoids | 0.002971% | 74.3mg/2.5kg | dry fruiting bodies of G. calidophilum | Phytochemistry (Elsevier) (2017), 143, 104-110 |
| **256** | 11β-Hydroxycedrelone | 283174-18-5 |  | Triterpenoids | 0.007738% | 325mg/4.2kg | Walsura Roxb. (Meliaceae) | J. Nat. Prod. 2000, 63, 947-951 dio.10.1021/np990607x |
| **257** | walsurin | 283174-64-1 |  | Triterpenoids | 0.000667% | 28mg/4.2kg | Walsura Roxb. (Meliaceae) | J. Nat. Prod. 2000, 63, 947-951 dio.10.1021/np990607x |
| **258** | isowalsuranolide | 283174-65-2 |  | Triterpenoids | 0.000286% | 12mg/4.2kg | Walsura Roxb. (Meliaceae) | J. Nat. Prod. 2000, 63, 947-951 dio.10.1021/np990607x |
| **259** | walsuranolide | 283174-66-3 |  | Triterpenoids | 0.000476% | 20mg/4.2kg | Walsura Roxb. (Meliaceae) | J. Nat. Prod. 2000, 63, 947-951 dio.10.1021/np990607x |
| **260** | 11β-acetoxywalsuranolide | 283174-67-4 |  | Triterpenoids | 1300/4200000 | 1300mg/4.2kg | Walsura Roxb. (Meliaceae) | J. Nat. Prod. 2000, 63, 947-951 dio.10.1021/np990607x |
| **261** | 20,22-dihydro-22,23-epoxywalsuranolide | 283174-68-5 |  | Triterpenoids | 0.045238% | 1900mg/4.2kg | Walsura Roxb. (Meliaceae) | J. Nat. Prod. 2000, 63, 947-951 dio.10.1021/np990607x |
| **262** | '11β-hydroxydihydrocedrelone | 283174-16-3 |  | Triterpenoids | 0.001476% | 62mg/4.2kg | Walsura Roxb. (Meliaceae) | J. Nat. Prod. 2000, 63, 947-951 dio.10.1021/np990607x |
| **263** | '11β-acetoxydihydrocedrelone | 283174-17-4 |  | Triterpenoids | 0.000810% | 34mg/4.2kg | Walsura Roxb. (Meliaceae) | J. Nat. Prod. 2000, 63, 947-951 dio.10.1021/np990607x |
| **264** | cedrelone | 1254-85-9 |  | Triterpenoids | 0.044048% | 1850mg/4.2kg | Walsura Roxb. (Meliaceae) | J. Nat. Prod. 2000, 63, 947-951 dio.10.1021/np990607x |
| **265** | 6β,19-Dihydroxy-3-oxours-12-en-28-oic acid | 194027-11-7 |  | Triterpenoids | 0.001500% | 15mg/1kg | Root bark of U. tomentosa | Phytochemistry (1997), 45, (5), 1035-104 |
| **266** | Ganoderic acid DM | 173075-45-1 |  | Triterpenoids | 0.001000% | 150mg/15kg | Ganoderma lucidum | European Journal of Pharmacology 602 (2009) 1–7 |
| **267** | Secaubryenol | 925932-08-7 |  | Triterpenoids | 0.002429% | 34mg/1.4kg | Gardenia aubryi | J. Nat. Prod. 2006, 69, 1711-1714 |
| **268** | Ganoderol A真菌提取物 | 104700-97-2 |  | Triterpenoids | 0.190000% | 19mg/10g | Ganderma lucidum | Chem. Pharm. Bull. 34(7)3025-3028(1986) |
| **269** | 3-O-acetyluncaric acid | 1290628-16-8 |  | Triterpenoids | 0.000140% | 7mg/5kg | Radermachera boniana twigs and leaves | J. Nat. Prod. 2011, 74, 1318–1322 doi.org/10.1021/np200022b |
| **270** | ursolic acid | 77-52-1 |  | Triterpenoids | 0.030000% | 1500mg/5kg | Radermachera boniana twigs and leaves | J. Nat. Prod. 2011, 74, 1318–1322 doi.org/10.1021/np200022b |
| **271** | 3-epioleanolic acid | 25499-90-5 |  | Triterpenoids | 0.000400% | 20mg/5kg | Radermachera boniana twigs and leaves | J. Nat. Prod. 2011, 74, 1318–1322 doi.org/10.1021/np200022b |
| **272** | ergosterol peroxide | 2061-64-5 |  | Triterpenoids | 0.000200% | 10mg/5kg | Radermachera boniana twigs and leaves | J. Nat. Prod. 2011, 74, 1318–1322 doi.org/10.1021/np200022b |
| **273** | β-sitostenone | 1058-61-3 |  | Triterpenoids | 0.000280% | 14mg/5kg | Radermachera boniana twigs and leaves | J. Nat. Prod. 2011, 74, 1318–1322 doi.org/10.1021/np200022b |
| **274** | rubelloside B | 167875-39-0 |  | Teterpenoid | 0.001240% | 62 mg/5 kg | Roots of Adina rubella | Phytochemistry, 39, 5, 1241-1243, 1996 |
| **275** | ganoderic acid A (6), | 81907-62-2 |  | Teterpenoid | 0.001492% | 149.2mg/10kg | Fruit Bodies of Ganoderma lucidum | Arch Pharm Res 32, 11, 1573-1579, 2009 |
| **276** | methyl ganoderate A | 81907-63-3 |  | Teterpenoid | 0.000110% | 11mg/10kg | Fruit Bodies of Ganoderma lucidum | Arch Pharm Res 32, 11, 1573-1579, 2009 |
| **277** | lucidenic acid A (8) | 95311-94-7 |  | Teterpenoid | 0.000270% | 27mg/10kg | Fruit Bodies of Ganoderma lucidum | Arch Pharm Res 32, 11, 1573-1579, 2009 |
| **278** | ganoderic acid SZ | 865543-37-9 |  | Teterpenoid | 0.000140% | 14mg/10kg | Fruit Bodies of Ganoderma lucidum | Arch Pharm Res 32, 11, 1573-1579, 2009 |
| **279** | 3-epimaslinic acid | 26563-68-8 |  | Teterpenoid | 0.003459% | 25 mg/722.7 g | Aerial parts of Centella asiatica | Biol. Pharm. Bull. 28(1) 173—175 (2005) |
| **280** | 11,12-dehydroursolic acid lactone | 35959-05-8 |  | Teterpenoid | 0.258000% | 2.4 mg/722.7 g | Aerial parts of Centella asiatica | Biol. Pharm. Bull. 28(1) 173—175 (2005) |
| **281** | ursolic acid | 77-52-1 |  | Teterpenoid | 0.268000% | 240 mg/722.7g | Aerial parts of Centella asiatica | Biol. Pharm. Bull. 28(1) 173—175 (2005) |
| **282** | pomolic acid | 13849-91-7 |  | Teterpenoid | 0.000553% | 4mg/mg/722.7 g | Aerial parts of Centella asiatica | Biol. Pharm. Bull. 28(1) 173—175 (2005) |
| **283** | pygenic acid A | 52213-27-1 |  | Teterpenoid | 0.288000% | 60 mg/722.7 g | Aerial parts of Centella asiatica | Biol. Pharm. Bull. 28(1) 173—175 (2005) |
| **284** | asiatic acid | 464-92-6 |  | Teterpenoid | 0.005535% | 40 mg/722.7 g | Aerial parts of Centella asiatica | Biol. Pharm. Bull. 28(1) 173—175 (2005) |
| **285** | corosolic acid | 4547-24-4 |  | Teterpenoid | 0.308000% | 8.4 mg/722.7 g | Aerial parts of Centella asiatica | Biol. Pharm. Bull. 28(1) 173—175 (2005) |
| **286** | cimiside B | 152685-91-1 |  | Teterpenoid | 0.000222% | 30mg/13.5kg | Roots of Cimicifuga dahurica | Aeta Pharmaeeutieas sinica 1993;28(10) :777一781 |
| **287** | rubraside A | 945267-63-0 |  | Teterpenoid | 0.000033% | 5.3mg/16kg | Roots of Cimicifuga dahurica | Journal of Shenyang Pharmaceutical University Vol.35 No.4.Apr．2018 |
| **288** | 25-acetylcimigenol-3-O-β-D-xylopyranoside | 1147272-91-0 |  | Teterpenoid | 0.014700% | 23.5mg/16kg | Roots of Cimicifuga dahurica | Journal of Shenyang Pharmaceutical University Vol.35 No.4.Apr．2018 |
| **289** | 25-anhydrocimigenol-3-O-β-D-xylopyranoside | 473554-75-5 |  | Teterpenoid | 0.000681% | 109mg/16kg | Roots of Cimicifuga dahurica | Journal of Shenyang Pharmaceutical University Vol.35 No.4.Apr．2018 |
| **290** | cimiracemoside E | 290821-40-8 |  | Teterpenoid | 0.000262% | 41.9mg/16kg | Roots of Cimicifuga dahurica | Journal of Shenyang Pharmaceutical University Vol.35 No.4.Apr．2018 |
| **291** | cimidahuside C | 851706-28-0 |  | Teterpenoid | 0.000103% | 15mg/14.5kg | Aerial parts of Cimicifuga dahurica | Yaoxue Xuebao (2003), 38(10), 763-766 |
| **292** | cimidahuside D | 851706-30-4 |  | Teterpenoid | 0.000069% | 10mg/14.5kg | Aerial parts of Cimicifuga dahurica | Yaoxue Xuebao (2003), 38(10), 763-766 |
| **293** | cimigenol | 3779-59-7 |  | Teterpenoid | 0.000065% | 12.3g/19kg | Rhizomes Cimicifuga dahurica | journal of Yunnan University of Traditional Chinese Medicine Vol. 36 No. 3 6. 2013 |
| **294** | cimigenol-3-one | 31222-32-9 |  | Teterpenoid | 0.000070% | 13.3mg/19kg | Rhizomes Cimicifuga dahurica | journal of Yunnan University of Traditional Chinese Medicine Vol. 36 No. 3 6. 2013 |
| **295** | 25-O-methyl-cimigenol | 1228555-24-5 |  | Teterpenoid | 0.000066% | 12.5mg/19kg | Rhizomes Cimicifuga dahurica | journal of Yunnan University of Traditional Chinese Medicine Vol. 36 No. 3 6. 2013 |
| **296** | 25-O-acetyl-cimigenol | 150972-73-9 |  | Teterpenoid | 0.000186% | 35.4mg/19kg | Rhizomes Cimicifuga dahurica | journal of Yunnan University of Traditional Chinese Medicine Vol. 36 No. 3 6. 2013 |
| **297** | 25-dehydrocimigenol | 2101869-11-6 |  | Teterpenoid | 0.000057% | 10.8mg/19kg | Rhizomes Cimicifuga dahurica | journal of Yunnan University of Traditional Chinese Medicine Vol. 36 No. 3 6. 2013 |
| **298** | cimigoside | 27994-11-2 |  | Teterpenoid | 0.000080% | 15.2mg/19kg | Rhizomes Cimicifuga dahurica | journal of Yunnan University of Traditional Chinese Medicine Vol. 36 No. 3 6. 2013 |
| **299** | acerinol | 19902-53-5 |  | Teterpenoid | 0.000156% | 70mg/45kg | Rhizomes of Cimicifuga species | Molecules. 2018 May 4;23(5) |
| **300** | 24-epi-acerinol | 151061-95-9 |  | Teterpenoid | 0.000178% | 80mg/45kg | Rhizomes of Cimicifuga species | Molecules. 2018 May 4;23(5) |
| **301** | 25-O-acetyl-cimigenol | 150972-73-9 |  | Teterpenoid | 0.000222% | 100mg/45kg | Rhizomes of Cimicifuga species | Molecules. 2018 May 4;23(5) |
| **302** | cimiside E | 154822-57-8 |  | Teterpenoid | 0.000003% | 1.5g/45kg | Rhizomes of Cimicifuga species | Molecules. 2018 May 4;23(5) |
| **303** | ceanothic acid | 21302-79-4 |  | Teterpenoid | 0.000030% | 10.5g/35kg | roots of Paliurus ramosissimus | Journal of Natural Products Vol. 54, No. 2, pp. 615-618, Mar-Apr 1991 |
| **304** | dimethyl emmolate | 31300-24-0 |  | Teterpenoid | 0.002470% | 410mg/16.6kg | Roots of Paliurus ramosissimus | Journal of Natural Products Vol. 55, No. 5, pp. 602-606, May 1992 |
| **305** | Ceanothic acid | 21302-79-4 |  | Teterpenoid | 0.000054% | 8.9g/16.6kg | Roots of Paliurus ramosissimus | Journal of Natural Products Vol. 55, No. 5, pp. 602-606, May 1992 |
| **306** | zizyberenalic acid | 125456-52-2 |  | Teterpenoid | 0.000054% | 22mg/41kg | Aerial parts Paliurus ramosissimus | Chinese Traditional and Herbal Drugs Vol.48 No.1 2017 |
| **307** | 3-O-protocatechuoylceanothic acid | 182682-97-9 |  | Teterpenoid | 0.000017% | 7mg/41kg | Aerial parts Paliurus ramosissimus | Chinese Traditional and Herbal Drugs Vol.48 No.1 2017 |
| **308** | messagenic acid A | 195312-17-5 |  | Teterpenoid | 0.000111% | 22.2mg/20kg | Lveaves of *Paliurus ramosissimus* | Chin. J. Org. Chem. 2017, 37, 520～525 |
| **309** | messagenic acid B | 195312-18-6 |  | Teterpenoid | 0.000078% | 15.5mg/20kg | Lveaves of *Paliurus ramosissimus* | Chin. J. Org. Chem. 2017, 37, 520～525 |
| **310** | betulinaldehyde | 13159-28-9 |  | Teterpenoid | 0.000231% | 46.1mg/20kg | Lveaves of *Paliurus ramosissimus* | Chin. J. Org. Chem. 2017, 37, 520～525 |
| **311** | ganoderol B | 104700-96-1 |  | Teterpenoid | 0.144000% | 72mg/50g | ruiting body of *Ganoderma lucidum* | Bioorganic & Medicinal Chemistry (2007), 15(14), 4966-4972. |
| **312** | ganodermenonol | 104700-97-2 |  | Teterpenoid | 0.000400% | 32mg/8kg | Fruiting bodies of *Ganoderma lucidum* | Journal of Natural Products Vol. 49, No. 4,pp. 621-625, Jul-Aug 1986 |
| **313** | ganodermadiol | 104700-96-1 |  | Teterpenoid | 0.000413% | 33mg/8kg | Fruiting bodies of Ganoderma lucidum | Journal of Natural Products Vol. 49, No. 4,pp. 621-625, Jul-Aug 1986 |
| **314** | alisol A 23-acetate | 19865-75-9 |  | Teterpenoid | 0.000600% | 18mg/3kg | Rhizomes of *Alismatis rhizoma* | J. Nat. Prod. 2014, 77, 1764−1769 |
| **315** | hibicusin | 853233-65-5 |  | Teterpenoid | 0.000025% | 5mg/20kg | *Stems of Hibiscus taiwanensis* | Chem. Pharm. Bull. 53(1) 56—59 (2005) |
| **316** | myriceric acid C | 162059-94-1 |  | Teterpenoid | 0.000080% | 16mg/20kg | *Stems of Hibiscus taiwanensis* | Chem. Pharm. Bull. 53(1) 56—59 (2005) |
| **317** | uncarinic acid A | 206256-62-4 |  | Teterpenoid | 0.000035% | 7mg/20kg | *Stems of Hibiscus taiwanensis* | Chem. Pharm. Bull. 53(1) 56—59 (2005) |
| **318** | uncarinic acid C | 277751-59-4 |  | Teterpenoid | 0.001000% | 18mg/1.5kg | *Hook of Uncaria rhynchophylla* | J Nat Med (2010) 64:506–509 |
| **319** | uncarinic acid D | 277751-60-7 |  | Teterpenoid | 0.000600% | 9mg/1.5kg | *Hook of Uncaria rhynchophylla* | J Nat Med (2010) 64:506–509 |
| **320** | obtusilinin | 139253-57-9 |  | Teterpenoid | 0.000007% | 0.3mg/4.1kg | *Barks of Betula platyphylla var. japonica* | Chem Biodivers. 2017 Apr;14(4) |
| **321** | winchic acid | 455886-25-6 |  | Teterpenoid | 0.000059% | 2.4mg/4.1kg | *Barks of Betula platyphylla var. japonica* | Chem Biodivers. 2017 Apr;14(4) |
| **322** | uncarinic acid E | 277751-61-8 |  | Teterpenoid | 0.000015% | 0.6mg/4.1kg | *Barks of Betula platyphylla var. japonica* | Chem Biodivers. 2017 Apr;14(4) |
| **323** | myriceric acid B | 55497-79-5 |  | Teterpenoid | 0.000127% | 5.2mg/4.1kg | *Barks of Betula platyphylla var. japonica* | Chem Biodivers. 2017 Apr;14(4) |
| **324** | lupenone | 1617-70-5 |  | Teterpenoid | 0.002867% | 43mg/1.5kg | *Twigs of Gardenia saxatilis* | Journal of Ethnopharmacology 88 (2003) 275–277 |
| **325** | lupeol | 545-47-1 |  | Teterpenoid | 0.004533% | 68mg/1.5kg | *Twigs of Gardenia saxatilis* | Journal of Ethnopharmacology 88 (2003) 275–277 |
| **326** | betulinic acid | 472-15-1 |  | Teterpenoid | 0.001000% | 15mg/1.5kg | *Twigs of Gardenia saxatilis* | Journal of Ethnopharmacology 88 (2003) 275–277 |
| **327** | oleanolic acid | 508-02-1 |  | Teterpenoid | 0.001067% | 16mg/1.5kg | *Twigs of Gardenia saxatilis* | Journal of Ethnopharmacology 88 (2003) 275–277 |
| **328** | ursolic acid | 77-52-1 |  | Teterpenoid | 0.000933% | 14mg/1.5kg | *Twigs of Gardenia saxatilis* | Journal of Ethnopharmacology 88 (2003) 275–277 |
| **329** | messagenic acid A | 195312-17-5 |  | Teterpenoid | 0.000200% | 3mg/1.5kg | *Twigs of Gardenia saxatilis* | Journal of Ethnopharmacology 88 (2003) 275–277 |
| **330** | messagenic acid B | 195312-18-6 |  | Teterpenoid | 0.000750% | 12mg/1.6kg | *Twigs of Gardenia saxatilis* | Journal of Ethnopharmacology 88 (2003) 275–277 |
| **331** | gardeniside B | 1458681-38-3 |  | Teterpenoid | 0.000367% | 55mg/15kg | *Roots of Gardenia jasminoidesEllis* | Fitoterapia (2012), 83(8), 1396-1401 |
| **332** | gardeniside C | 1458681-39-4 |  | Teterpenoid | 0.000733% | 110mg/15kg | *Roots of Gardenia jasminoidesEllis* | Fitoterapia (2012), 83(8), 1396-1401 |
| **333** | quinovic acid | 465-74-7 |  | Teterpenoid | 0.000140% | 7mg/5kg | *Shoots of Uncaria Scandens(Smith) Hutchins* | Molecules (2013), 18, 9727-9734 |
| **334** | ursolic acid | 77-52-1 |  | Teterpenoid | 0.000160% | 8mg/5kg | *Shoots of Uncaria Scandens(Smith) Hutchins* | Molecules (2013), 18, 9727-9734 |
| **335** | cycloeucalenol | 469-39-6 |  | Teterpenoid | 0.000180% | 9mg/5kg | *Shoots of Uncaria Scandens(Smith) Hutchins* | Molecules (2013), 18, 9727-9734 |
| **336** | 3-acetyl oleanolic acid | 14605-17-5 |  | Teterpenoid | 0.000250% | 20mg/8kg | *Roots of Adina rubella* | Journal of China Pharmaceutical University  2015，46(5):556-560 |
| **337** | uncarilic acid | 1613244-08-8 |  | Teterpenoid | 0.000133% | 12mg/9kg | *Hooks and stems of Uucaria sessilifructus* | Molecules 2013, 18, 9727-9734 |
| **338** | secouncarilic acid | 1800563-52-3 |  | Teterpenoid | 0.000078% | 7mg/9kg | *Hooks and stems of Uucaria sessilifructus* | Molecules 2013, 18, 9727-9734 |
| **339** | ursolic acid | 77-52-1 |  | Teterpenoid | 0.000322% | 29mg/9kg | *Hooks and stems of Uucaria sessilifructus* | Molecules 2013, 18, 9727-9734 |
| **340** | uncaric acid | 123135-05-7 |  | Teterpenoid | 0.000089% | 8mg/9kg | *Hooks and stems of Uucaria sessilifructus* | Molecules 2013, 18, 9727-9734 |
| **341** | 3β,6β,19α-trihydroxy-23-oxo-urs-12-en-28-oic acid | 131984-82-2 |  | Teterpenoid | 0.000122% | 11mg/9kg | *Hooks and stems of Uucaria sessilifructus* | Molecules 2013, 18, 9727-9734 |
| **342** | 2α,​3β,​6β,​7β-​Tetrahydroxyurs-​12-​en-​28-​oic acid | 2197080-98-9 |  | Teterpenoid | 0.000256% | 23mg/9kg | *Hooks and stems of Uucaria sessilifructus* | Molecules 2013, 18, 9727-9734 |
| **343** | quinovic acid | 465-74-7 |  | Teterpenoid | 0.000189% | 17mg/9kg | *Hooks and stems of Uucaria sessilifructus* | Molecules 2013, 18, 9727-9734 |
| **344** | cinchonaglycoside C | 79955-41-2 |  | Teterpenoid | 0.000178% | 16mg/9kg | *Hooks and stems of Uucaria sessilifructus* | Molecules 2013, 18, 9727-9734 |
| **345** | zygophyloside B | 133084-07-8 |  | Teterpenoid | 0.000144% | 13mg/9kg | *Hooks and stems of Uucaria sessilifructus* | Molecules 2013, 18, 9727-9734 |
| **346** | walsuronoid B | 942582-15-2 |  | Teterpenoid | 0.000205% | 24.2mg/11.8kg | *Fruits of Walsura robusta* | Phytochemistry 136 (2017) 108-118 |
| **347** | dysoxylumosin G | 1802570-79-1 |  | Teterpenoid | 0.000051% | 6mg/11.8kg | *Fruits of Walsura robusta* | Phytochemistry 136 (2017) 108-118 |
| **348** | dihydrocedrelone | 1254-84-8 |  | Teterpenoid | 0.000055% | 6.5mg/11.8kg | *Fruits of Walsura robusta* | Phytochemistry 136 (2017) 108-118 |
| **349** | 11β-hydroxydihydrocedrelone | 283174-16-3 |  | Teterpenoid | 0.000024% | 2.8mg/11.8kg | *Fruits of Walsura robusta* | Phytochemistry 136 (2017) 108-118 |
| **350** | 11β-acetoxydihydrocedrelone | 283174-17-4 |  | Teterpenoid | 0.000205% | 24.2mg/11.8kg | *Fruits of Walsura robusta* | Phytochemistry 136 (2017) 108-118 |
| **351** | cedrelone | 1254-85-9 |  | Teterpenoid | 0.000029% | 5.2mg/11.8kg | *Fruits of Walsura robusta* | Phytochemistry 136 (2017) 108-118 |
| **352** | 11β-hydroxycedrelone | 283174-18-5 |  | Teterpenoid | 0.000041% | 4.8mg/11.8kg | *Fruits of Walsura robusta* | Phytochemistry 136 (2017) 108-118 |
| **353** | 11β, 12α-diacetoxycedrelone | 220494-50-8 |  | Teterpenoid | 0.000025% | 3mg/11.8kg | *Fruits of Walsura robusta* | Phytochemistry 136 (2017) 108-118 |
| **354** | anthothecol | 10410-83-0 |  | Teterpenoid | 0.001270% | 150mg/11.8kg | *Fruits of Walsura robusta* | Phytochemistry 136 (2017) 108-118 |
| **355** | walsunoid H | 1885961-75-0 |  | Teterpenoid | 0.000205% | 24.2mg/11.8kg | *Fruits of Walsura robusta* | Phytochemistry 136 (2017) 108-118 |
| **356** | isowalsuranolide | 283174-65-2 |  | Teterpenoid | 0.000024% | 2.8mg/11.8kg | *Fruits of Walsura robusta* | Phytochemistry 136 (2017) 108-118 |
| **357** | walsurin | 283174-64-1 |  | Teterpenoid | 0.000055% | 6.5mg/11.8kg | *Fruits of Walsura robusta* | Phytochemistry 136 (2017) 108-118 |
| **358** | grandifoliolenone | 18524-60-2 |  | Teterpenoid | 0.000030% | 3.5mg/11.8kg | *Fruits of Walsura robusta* | Phytochemistry 136 (2017) 108-118 |
| **359** | hispidol B | 78739-39-6 |  | Teterpenoid | 0.000069% | 8.2mg/11.8kg | *Fruits of Walsura robusta* | Phytochemistry 136 (2017) 108-118 |
| **360** | betulinic acid | 472-15-1 |  | Teterpenoid | 0.000032% | 3.8mg/11.8kg | *Fruits of Walsura robusta* | Phytochemistry 136 (2017) 108-118 |
| **361** | walsunoids A | 1885961-62-5 |  | Teterpenoid | 0.000042% | 5mg/12kg | *Fruits of Walsura robusta* | J. Nat. Prod. 2016, 79, 899−906 |
| **362** | walsuranolide B | 620887-95-7 |  | Teterpenoid | 0.000120% | 6mg/5kg | *Leaves and twigs of Walsura yunnanensis* | J. Nat. Prod. 2014, 77, 1764−1769 |
| **363** | 11β-​Hydroxy-​23-​O-​methylwalsuranolide | 1620887-96-8 |  | Teterpenoid | 0.000040% | 2mg/5kg | *Leaves and twigs of Walsura yunnanensis* | J. Nat. Prod. 2014, 77, 1764−1769 |
| **364** | yunnanolide A | 1620887-97-9 |  | Teterpenoid | 0.000120% | 6mg/5kg | *Leaves and twigs of Walsura yunnanensis* | J. Nat. Prod. 2014, 77, 1764−1769 |
| **365** | yunnanol A | 1620887-98-0 |  | Teterpenoid | 0.000060% | 3mg/5kg | *Leaves and twigs of Walsura yunnanensis* | J. Nat. Prod. 2014, 77, 1764−1769 |
| **366** | 11β-hydroxyisowalsuranolide | 1620832-80-5 |  | Teterpenoid | 0.000200% | 10mg/5kg | *Leaves and twigs of Walsura yunnanensis* | J. Nat. Prod. 2014, 77, 1764−1769 |
| **367** | 11β-hydroxy-1,2-dihydroisowalsuranolide | 1620832-81-6 |  | Teterpenoid | 0.000160% | 8mg/5kg | *Leaves and twigs of Walsura yunnanensis* | J. Nat. Prod. 2014, 77, 1764−1769 |
| **368** | 1α,11β-dihydroxy-1,2-dihydroisowalsuranolide | 1620832-82-7 |  | Teterpenoid | 0.000120% | 6mg/5kg | *Leaves and twigs of Walsura yunnanensis* | J. Nat. Prod. 2014, 77, 1764−1769 |
| **369** | 11β-hydroxy-1α-methoxy-1,2-dihydroisowalsuranolide | 1620832-83-8 |  | Teterpenoid | 0.000040% | 2mg/5kg | *Leaves and twigs of Walsura yunnanensis* | J. Nat. Prod. 2014, 77, 1764−1769 |
| **370** | yunnanolide B | 1620887-99-1 |  | Teterpenoid | 0.002000% | 100mg/5kg | *Leaves and twigs of Walsura yunnanensis* | J. Nat. Prod. 2014, 77, 1764−1769 |
| **371** | (24S*,25R*)-cycloartane-3β,24,25,26-tetrol | 1620888-00-7 |  | Teterpenoid | 0.001600% | 80mg/5kg | *Leaves and twigs of Walsura yunnanensis* | J. Nat. Prod. 2014, 77, 1764−1769 |
| **372** | cedrelone | 1254-85-9 |  | Teterpenoid | 0.000250% | 15mg/6kg | *Leaves of Toona ciliata var. yunnanensis* | Phytochemistry 76 (2012) 141–149 |
| **373** | epoxyazadiradione | 18385-59-6 |  | Teterpenoid | 0.000200% | 12mg/6kg | *Leaves of Toona ciliata var. yunnanensis* | Phytochemistry 76 (2012) 141–149 |
| **374** | toonafolin | 80161-81-5 |  | Teterpenoid | 0.000667% | 40mg/6kg | *Leaves of Toona ciliata var. yunnanensis* | Phytochemistry 76 (2012) 141–149 |
| **375** | toonaciliatin E | 948556-28-3 |  | Teterpenoid | 0.000083% | 5mg/6kg | *Leaves of Toona ciliata var. yunnanensis* | Phytochemistry 76 (2012) 141–149 |
| **376** | toonaciliatin H | 81047-67-8 |  | Teterpenoid | 0.000117% | 7mg/6kg | *Leaves of Toona ciliata var. yunnanensis* | Phytochemistry 76 (2012) 141–149 |
| **377** | dysobinin | 62655-03-2 |  | Teterpenoid | 0.000067% | 4mg/6kg | *Leaves of Toona ciliata var. yunnanensis* | Phytochemistry 76 (2012) 141–149 |
| **378** | 17-hydroxyazadiradione | 67106-58-5 |  | Teterpenoid | 0.000050% | 3mg/6kg | *Leaves of Toona ciliata var. yunnanensis* | Phytochemistry 76 (2012) 141–149 |
| **379** | dihydroniloticin | 115334-05-9 |  | Teterpenoid | 0.000300% | 18mg/6kg | *Leaves of Toona ciliata var. yunnanensis* | Phytochemistry 76 (2012) 141–149 |
| **380** | dehydroodoratol | 16962-90-6 |  | Teterpenoid | 0.000250% | 15mg/6kg | *Leaves of Toona ciliata var. yunnanensis* | Phytochemistry 76 (2012) 141–149 |
| **381** | 21-α-methylmelianodiol | 956104-89-5 |  | Teterpenoid | 0.000200% | 12mg/6kg | *Leaves of Toona ciliata var. yunnanensis* | Phytochemistry 76 (2012) 141–149 |
| **382** | walsurin | 283174-64-1 |  | Teterpenoid | 0.000667% | 28mg/4.2kg | *Barks of Walsura yunnanensis* | Journal of Natural Products (2000), 63(7), 947-951 |
| **383** | isowalsuranolide | 283174-65-2 |  | Teterpenoid | 0.000286% | 12mg/4.2kg | *Barks of Walsura yunnanensis* | Journal of Natural Products (2000), 63(7), 947-951 |
| **384** | walsuranolide | 283174-66-3 |  | Teterpenoid | 0.000476% | 20mg/4.2kg | *Barks of Walsura yunnanensis* | Journal of Natural Products (2000), 63(7), 947-951 |
| **385** | 11β-acetoxywalsuranolide | 283174-67-4 |  | Teterpenoid | 0.000667% | 28mg/4.2kg | *Barks of Walsura yunnanensis* | Journal of Natural Products (2000), 63(7), 947-951 |
| **386** | 20,22-dihydro-22,23-epoxywalsuranolide | 283174-68-5 |  | Teterpenoid | 0.000071% | 3mg/4.2kg | *Barks of Walsura yunnanensis* | Journal of Natural Products (2000), 63(7), 947-951 |
| **387** | 11β-hydroxydihydrocedrelone | 283174-16-3 |  | Teterpenoid | 0.001476% | 62mg/4.2kg | *Barks of Walsura yunnanensis* | Journal of Natural Products (2000), 63(7), 947-951 |
| **388** | 11β-acetoxydihydrocedrelone | 283174-17-4 |  | Teterpenoid | 0.000810% | 34mg/4.2kg | *Barks of Walsura yunnanensis* | Journal of Natural Products (2000), 63(7), 947-951 |
| **389** | 11β-hydroxycedrelone | 283174-18-5 |  | Teterpenoid | 0.007738% | 325mg/4.2kg | *Barks of Walsura yunnanensis* | Journal of Natural Products (2000), 63(7), 947-951 |
| **390** | cedrelone | 1254-85-9 |  | Teterpenoid | 0.044048% | 1.85g/4.2kg | *Barks of Walsura yunnanensis* | Journal of Natural Products (2000), 63(7), 947-951 |
| **391** | (20R,24R)-ocotillone | 54911-11-4 |  | Teterpenoid | 1.792453% | 950mg/53g | *Apical buds of Gardenia collinsae* | Phytochemistry Letters 4 (2011) 26–29 |
| **392** | coronalonic acid | 268214-52-4 |  | Teterpenoid | 0.000210% | 0.5mg/238g | *Apical buds of Gardenia sootepensis* | Fitoterapia 114 (2016) 92–97 |
| **393** | sootepin D | 1154518-97-4 |  | Teterpenoid | 0.000420% | 1mg/238g | *Apical buds of Gardenia sootepensis* | Fitoterapia 114 (2016) 92–97 |
| **394** | coronalolide | 268214-51-3 |  | Teterpenoid | 0.001680% | 4mg/238g | *Apical buds of Gardenia sootepensis* | Fitoterapia 114 (2016) 92–97 |
| **395** | coronalolide methyl ester | 268214-50-2 |  | Teterpenoid | 0.006303% | 1.5mg/238g | *Apical buds of Gardenia sootepensis* | Fitoterapia 114 (2016) 92–97 |
| **396** | α-Onocerin | 511-01-3 |  | Teterpenoid | 0.001800% | 153mg/8.5kg | *Whole plant of Lycopodium Japonicum* | Chin J Mod Appl Pharm, 2018 December.35 No.12 |
| **397** | lycoclavanol | 13956-51-9 |  | Teterpenoid | 0.001200% | 102mg/8.5kg | *Whole plant of Lycopodium Japonicum* | Chin J Mod Appl Pharm, 2018 December.35 No.12 |
| **398** | α-Onocerin | 511-01-3 |  | Teterpenoid | 0.000182% | 20mg/11kg | *Whole plant of Lycopodium Japonicum* | Journal of Hainan Normal University （Natural Science）Vol.29 No.3 Sep.2016 |
| **399** | 26-nor-8-oxo-α-onocerin | 125124-68-7 |  | Teterpenoid | 0.000182% | 20mg/11kg | *Whole plant of Lycopodium Japonicum* | Journal of Hainan Normal University （Natural Science）Vol.29 No.3 Sep.2016 |
| **400** | diepiserratenediol | 3604-92-0 |  | Teterpenoid | 0.001273% | 140mg/11kg | *Whole plant of Lycopodium Japonicum* | Journal of Hainan Normal University （Natural Science）Vol.29 No.3 Sep.2016 |
| **401** | serrat-14-en-3β,21α-diol | 2239-24-9 |  | Teterpenoid | 0.000060% | 6mg/10kg | *Whole plant of Lycopodium Japonicum* | Chinese Journal of Experimental Traditional Medical Formulae Vol． 18，No．9 |
| **402** | serrat-14-en-3β,21β-diol | 1449-06-5 |  | Teterpenoid | 0.000230% | 23mg/10kg | *Whole plant of Lycopodium Japonicum* | Chinese Journal of Experimental Traditional Medical Formulae Vol． 18，No．9 |
| **403** | α-Onocerin | 511-01-3 |  | Teterpenoid | 0.000250% | 25mg/10kg | *Whole plant of Lycopodium Japonicum* | Chinese Journal of Experimental Traditional Medical Formulae Vol． 18，No．9 |
| **404** | daucosterol | 474-58-8 |  | Teterpenoid | 0.000120% | 12mg/10kg | *Whole plant of Lycopodium Japonicum* | Chinese Journal of Experimental Traditional Medical Formulae Vol． 18，No．9 |
| **405** | glutinone | 508-09-8 |  | Teterpenoid | 0.000150% | 15mg/10kg | *Whole plant of Lycopodium Japonicum* | 杨跃辉2004 |
| **406** | 24-methylenecycloartan-3β-ol | 1449-09-8 |  | Teterpenoid | 0.004839% | 15mg/310g | *Aerial parts of Euphorbia macrostegia* | Iranian Journal of Pharmaceutical Research (2015), 14 (1): 243-249 |
| **407** | butyrospermol | 472-28-6 |  | Teterpenoid | 0.014516% | 45mg/310g | *Aerial parts  of Euphorbia macrostegia* | Iranian Journal of Pharmaceutical Research (2015), 14 (1): 243-249 |
| **408** | cycloartenol | 469-38-5 |  | Teterpenoid | 0.006452% | 20mg/310g | *Aerial parts  of Euphorbia macrostegia* | Iranian Journal of Pharmaceutical Research (2015), 14 (1): 243-249 |
| **409** | lycojaponicuminol A | 1651839-32-5 |  | Teterpenoid | 0.000010% | 2mg/20.8kg | *Whole moss of Lycopodium japonicum* | Fitoterapia (2014), 96, 95-102. |
| **410** | lycojaponicuminol B | 1651839-33-6 |  | Teterpenoid | 0.000012% | 2.5mg/20.8kg | *Whole moss of Lycopodium japonicum* | Fitoterapia (2014), 96, 95-102. |
| **411** | lycojaponicuminol C | 1651839-34-7 |  | Teterpenoid | 0.000012% | 2.5mg/20.8kg | *Whole moss of Lycopodium japonicum* | Fitoterapia (2014), 96, 95-102. |
| **412** | lycojaponicuminol D | 1651839-35-8 |  | Teterpenoid | 0.000019% | 4mg/20.8kg | *Whole moss of Lycopodium japonicum* | Fitoterapia (2014), 96, 95-102. |
| **413** | Lycojaponicuminol E | 1651839-36-9 |  | Teterpenoid | 0.000019% | 4mg/20.8kg | *Whole moss of Lycopodium japonicum* | Fitoterapia (2014), 96, 95-102. |
| **414** | lycojaponicuminol F | 56423-30-4 |  | Teterpenoid | 0.000010% | 2.1mg/20.8kg | *Whole moss of Lycopodium japonicum* | Fitoterapia (2014), 96, 95-102. |
| **415** | Sablacaurin A | 719302-63-3 |  | Teterpenoid | 0.000214% | 7.7mg/3.6kg | *Leaves of Sabal causiarum* | Phytochemistry 65 (2004) 1153–1157 |
| **416** | sablacaurin B | 719302-65-5 |  | Teterpenoid | 0.000244% | 8.8mg/3.6kg | *Leaves of Sabal causiarum* | Phytochemistry 65 (2004) 1153–1157 |
| **417** | serratenediol | 2239-24-9 |  | Teterpenoid | 0.010900% | 10.9mg/100g | *Aerial parts of Huperzia crassa* | Journal of Ethnopharmacology 193 (2016) 546–554 |
| **418** | 21-episerratenediol | 1449-06-5 |  | Teterpenoid | 0.004200% | 4.2mg/100g | *Aerial parts of Huperzia crassa* | Journal of Ethnopharmacology 193 (2016) 546–554 |
| **419** | rubuminatus A | 1772614-24-0 |  | Teterpenoid | 0.000024% | 5mg/20.5kg | *Underground portions of Rubus innominatus* | Phytochemistry 116 (2015) 329–336 |
| **420** | rubuminatus B | 1772614-25-1 |  | Teterpenoid | 0.000034% | 7mg/20.5kg | *Underground portions of Rubus innominatus* | Phytochemistry 116 (2015) 329–336 |
| **421** | 2-O-benzoyl alphitolic acid | 1772612-74-4 |  | Teterpenoid | 0.000020% | 4mg/20.5kg | *Underground portions of Rubus innominatus* | Phytochemistry 116 (2015) 329–336 |
| **422** | euscaphic acid | 53155-25-2 |  | Teterpenoid | 0.000566% | 116mg/20.5kg | *Underground portions of Rubus innominatus* | Phytochemistry 116 (2015) 329–336 |
| **423** | 2-oxo-pomolic acid | 54963-52-9 |  | Teterpenoid | 0.000107% | 22mg/20.5kg | *Underground portions of Rubus innominatus* | Phytochemistry 116 (2015) 329–336 |
| **424** | ursolic acid | 77-52-1 |  | Teterpenoid | 0.000161% | 33mg/20.5kg | *Underground portions of Rubus innominatus* | Phytochemistry 116 (2015) 329–336 |
| **425** | 1β-hydroxyeuscaphic acid | 120211-98-5 |  | Teterpenoid | 0.000185% | 38mg/20.5kg | *Underground portions of Rubus innominatus* | Phytochemistry 116 (2015) 329–336 |
| **426** | pinfaensic acid | 158848-21-6 |  | Teterpenoid | 0.000039% | 8mg/20.5kg | *Underground portions of Rubus innominatus* | Phytochemistry 116 (2015) 329–336 |
| **427** | tormentic acid | 13850-16-3 |  | Teterpenoid | 0.000102% | 21mg/20.5kg | *Underground portions of Rubus innominatus* | Phytochemistry 116 (2015) 329–336 |
| **428** | uvaol | 545-46-0 |  | Teterpenoid | 0.000039% | 8mg/20.5kg | *Underground portions of Rubus innominatus* | Phytochemistry 116 (2015) 329–336 |
| **429** | fupenzic acid | 119725-20-1 |  | Teterpenoid | 0.000059% | 12mg/20.5kg | *Underground portions of Rubus innominatus* | Phytochemistry 116 (2015) 329–336 |
| **430** | pomolic acid | 13849-91-7 |  | Teterpenoid | 0.000707% | 145mg/20.5kg | *Underground portions of Rubus innominatus* | Phytochemistry 116 (2015) 329–336 |
| **431** | annurcoic acid | 877995-09-0 |  | Teterpenoid | 0.000058% | 12mg/20.5kg | *Underground portions of Rubus innominatus* | Phytochemistry 116 (2015) 329–336 |
| **432** | swinhoeic acid | 343782-24-1 |  | Teterpenoid | 0.000054% | 11mg/20.5kg | *Underground portions of Rubus innominatus* | Phytochemistry 116 (2015) 329–336 |
| **433** | 1β-hydroxy-2-oxopomolic acid | 217466-37-0 |  | Teterpenoid | 0.000029% | 6mg/20.5kg | *Underground portions of Rubus innominatus* | Phytochemistry 116 (2015) 329–336 |
| **434** | Ziyuglycoside II | 35286-59-0 |  | Teterpenoid | 0.218000% | 2.18∼2.91 mg/g | *Roots of Sanguisorba officinalis L.* | Front Pharmacol. 2017 Mar 16;8:130 |
| **435** | quinatic acid | 119863-89-7 |  | Teterpenoid | 0.000405% | 60mg/14.8kg | *Intermedia stems of Stauntonia obovatifoliola Hayata subsp.* | China Journal of chinese material medics vol 39,issue 23,december ,2014 |
| **436** | 24-O-acetyl quinatic acid | 119863-92-2 |  | Teterpenoid | 0.000068% | 10mg/14.8kg | *Intermedia stems of Stauntonia obovatifoliola Hayata subsp.* | China Journal of chinese material medics vol 39,issue 23,december ,2014 |
| **437** | stauntoside A | 261636-64-0 |  | Teterpenoid | 0.000176% | 26mg/14.8kg | *Intermedia stems of Stauntonia obovatifoliola Hayata subsp.* | China Journal of chinese material medics vol 39,issue 23,december ,2014 |
| **438** | kalopanax saponin A | 27013-91-8 |  | Teterpenoid | 0.004392% | 650mg/14.8kg | *Intermedia stems of Stauntonia obovatifoliola Hayata subsp.* | China Journal of chinese material medics vol 39,issue 23,december ,2014 |
| **439** | kalopanax saponin J | 29781-27-9 |  | Teterpenoid | 0.002703% | 400mg/14.8kg | *Intermedia stems of Stauntonia obovatifoliola Hayata subsp.* | China Journal of chinese material medics vol 39,issue 23,december ,2014 |
| **440** | (-)-leucophyllone | 170904-49-1 |  | Teterpenoid | 0.000492% | 46.7mg/9.5kg | *Stems of Aphanamixis grandifolia* | Phytochemistry 80 (2012) 148–155 |
| **441** | alphitolic acid | 19533-92-7 |  | Teterpenoid | 0.000320% | 8mg/2.5kg | *Twigs of Chaenomeles sinensis* | Chem. Pharm. Bull. 51(11) 1318—1321 (2003) |
| **442** | tormentic acid | 13850-16-3 |  | Teterpenoid | 0.000720% | 18mg/2.5kg | *Twigs of Chaenomeles sinensis* | Chem. Pharm. Bull. 51(11) 1318—1321 (2003) |
| **443** | euscaphic acid | 53155-25-2 |  | Teterpenoid | 0.000120% | 3mg/2.5kg | *Twigs of Chaenomeles sinensis* | Chem. Pharm. Bull. 51(11) 1318—1321 (2003) |
| **444** | corosolic acid | 4547-24-4 |  | Teterpenoid | 0.000200% | 5mg/2.5kg | *Twigs of Chaenomeles sinensis* | Chem. Pharm. Bull. 51(11) 1318—1321 (2003) |
| **445** | maslinic acid | 4373-41-5 |  | Teterpenoid | 0.000240% | 6mg/2.5kg | *Twigs of Chaenomeles sinensis* | Chem. Pharm. Bull. 51(11) 1318—1321 (2003) |
| **446** | erythrodiol | 545-48-2 |  | Teterpenoid | 0.000800% | 20mg/2.5kg | *Twigs of Chaenomeles sinensis* | Chem. Pharm. Bull. 51(11) 1318—1321 (2003) |
| **447** | dysolenticin A | 1337972-99-2 |  | Teterpenoid | 0.000027% | 4mg/14.9kg | *Leaves of Dysoxylum lenticellatum* | J. Nat. Prod. 2011, 74, 2235–2242 |
| **448** | dysolenticin B | 1337973-00-8 |  | Teterpenoid | 0.000020% | 3mg/14.9kg | *Leaves of Dysoxylum lenticellatum* | J. Nat. Prod. 2011, 74, 2235–2242 |
| **449** | dysolenticin C | 1337973-01-9 |  | Teterpenoid | 0.000020% | 3mg/14.9kg | *Leaves of Dysoxylum lenticellatum* | J. Nat. Prod. 2011, 74, 2235–2242 |
| **450** | kairatenyl palmitate | 455253-27-7 |  | Teterpenoid | 0.371429% | 2.6mg/0.7g | *twigs of Brachylaena ramiflora* | J. Nat. Prod. 2002, 65, 1222-1224 |
| **451** | hopenyl palmitate | 455253-28-8 |  | Teterpenoid | 0.328571% | 2.3mg/0.7g | *twigs of Brachylaena ramiflora* | J. Nat. Prod. 2002, 65, 1222-1224 |
| **452** | α-amyrin palmitate | 22255-10-3 |  | Teterpenoid | 0.171429% | 1.2mg/0.7g | *twigs of Brachylaena ramiflora* | J. Nat. Prod. 2002, 65, 1222-1224 |
| **453** | β-amyrin palmitate | 5973--6--8 |  | Teterpenoid | 0.300000% | 2.1mg/0.7g | *twigs of Brachylaena ramiflora* | J. Nat. Prod. 2002, 65, 1222-1224 |
| **454** | β-amyrin acetate | 1616-93-9 |  | Teterpenoid | 0.171429% | 1.2mg/0.7g | *twigs of Brachylaena ramiflora* | J. Nat. Prod. 2002, 65, 1222-1224 |
| **455** | lupeyl acetate | 1617-68-1 |  | Teterpenoid | 0.371429% | 2.6mg/0.7g | *twigs of Brachylaena ramiflora* | J. Nat. Prod. 2002, 65, 1222-1224 |
| **456** | lupeol | 545-47-1 |  | Teterpenoid | 0.400000% | 2.8mg/0.7g | *twigs of Brachylaena ramiflora* | J. Nat. Prod. 2002, 65, 1222-1224 |
| **457** | 3-epicabraleadiol | 19942-04-**2** |  | Teterpenoid | 0.000273% | 12mg/4.4kg | *Oil of Camellia japonica* | Chem. Pharm. Bull. 52(1) 153—156 (2004) |
| **458** | ocotillol II | 19942-04-2 |  | Teterpenoid | 0.000086% | 3.8mg/4.4kg | *Oil of Camellia japonica* | Chem. Pharm. Bull. 52(1) 153—156 (2004) |
| **459** | ocotillol I | 28506-81-2 |  | Teterpenoid | 0.000045% | 2mg/4.4kg | *Oil of Camellia japonica* | Chem. Pharm. Bull. 52(1) 153—156 (2004) |
| **460** | dammarenediol II | 14351-29-2 |  | Teterpenoid | 0.001523% | 67mg/4.4kg | *Oil of Camellia japonica* | Chem. Pharm. Bull. 52(1) 153—156 (2004) |
| **461** | davinvolunol B | 1820946-30-2 |  | Teterpenoid | 0.000152% | 15.2mg/10kg | *Branch Barks of Davidia involucrata* | Molecules 2014, 19, 17619-17631 |
| **462** | 21-episerratenediol | 1449-06-5 |  | Teterpenoid | 0.000157% | 15.7mg/10kg | *Whole plant of Davidia involucrata* | Chinese Traditional and Herbal Drugs  Vol 45 .24 2014 12 |
| **463** | serratenediol | 2239-24-9 |  | Teterpenoid | 0.000405% | 40.5mg/10kg | *Whole plant of Davidia involucrata* | Chinese Traditional and Herbal Drugs  Vol 45 .24 2014 12 |
| **464** | α-onocerin | 511-01-3 |  | Teterpenoid | 0.000352% | 35.2mg/10kg | *Whole plant of Davidia involucrata* | Chinese Traditional and Herbal Drugs  Vol 45 .24 2014 12 |
| **465** | lycoclavanol | 13956-51-9 |  | Teterpenoid | 0.000081% | 8.1mg/10kg | *Whole plant of Davidia involucrata* | Chinese Traditional and Herbal Drugs  Vol 45 .24 2014 12 |
| **466** | lyclaninol | 53755-76-3 |  | Teterpenoid | 0.000062% | 6.2mg/10kg | *Whole plant of Davidia involucrata* | Chinese Traditional and Herbal Drugs  Vol 45 .24 2014 12 |
| **467** | lycernuic ketone C | 53800-21-8 |  | Teterpenoid | 0.000030% | 3mg/10kg | *Whole plant of Davidia involucrata* | Chinese Traditional and Herbal Drugs  Vol 45 .24 2014 12 |
| **468** | limonin | 1180-71-8 |  | Teterpenoid | 0.000220% | 110mg/50kg | *Root barks of Dictamnus dasycarpus* | Chinese Traditional Patent Medicine November 2016 Vol．38 No．11 |
| **469** | soyasapogenol B | 595-15-3 |  | Teterpenoid | 0.000011% | 3mg/27kg | *Whole plant of Crotalaria sessiliflora* | Acta Pharmaceutica Sinica 2016, 51 (5): 775−779 |
| **470** | Soyasapogenol B | 595-15-3 |  | Teterpenoid | 0.000100% | 10mg/10kg | *Whole plant of Sophora subprostrata* | Chinese Traditional Patent Medicine November 2016 Vol．38 No．4 |
| **471** | Obacunone | 751-03-1 |  | Teterpenoid | 0.130000% | 0.130000% | *Seeds of Citrus limon* | J. Agric. Food Chem. 2002, 50, 6766-6774 |
| **472** | limonin | 1180-71-8 |  | Teterpenoid | 0.240000% | 0.240000% | *Seeds of Citrus limon* | J. Agric. Food Chem. 2002, 50, 6766-6774 |
| **473** | nomilin | 1063-77-0 |  | Teterpenoid | 0.200000% | 0.200000% | *Seeds of Citrus limon* | J. Agric. Food Chem. 2002, 50, 6766-6774 |
| **474** | Ziyuglycoside I | 35286-58-9 |  | Teterpenoid | 1.635000% | 16.35∼18.17 mg/g | *Seeds of Sanguisorba officinalis L.* | Front Pharmacol. 2017 Mar 16;8:130 |
| **475** | Arjunolic acid | 465-00-9 |  | Teterpenoid | 0.000333% | 18mg/5.4kg | *Leaves of Terminalia catappa* | Chinese Traditional Patent Medicine November 2018 Vol． 40 No． 11 |
| **476** | Zeorin | 22570-53-2 |  | Teterpenoid | 0.002229% | 7.8mg/350g | *Whole moss of Frullania brasiliensis* | Phytochemistry 59 (2002) 205–213 |
| **477** | Dysolenticin A | 1337972-99-2 |  | Teterpenoid | 0.000002% | 4mg/14.9kg | *Leaves and twigs of Dysoxylum lenticellatum* | J. Nat. Prod. 2011, 74, 2235–2242 |
| **478** | dysolenticin B | 1337973-00-8 |  | Teterpenoid | 0.000020% | 3mg/14.9kg | *Leaves and twigs of Dysoxylum lenticellatum* | J. Nat. Prod. 2011, 74, 2235–2242 |
| **479** | dysolenticin C | 1337973-01-9 |  | Teterpenoid | 0.000020% | 3mg/14.9kg | *Leaves and twigs of Dysoxylum lenticellatum* | J. Nat. Prod. 2011, 74, 2235–2242 |
| **480** | dysolenticins D | 1337973-02-0 |  | Teterpenoid | 0.000302% | 45mg/14.9kg | *Leaves and twigs of Dysoxylum lenticellatum* | J. Nat. Prod. 2011, 74, 2235–2242 |
| **481** | dysolenticin E | 1337973-03-1 |  | Teterpenoid | 0.000054% | 8mg/14.9kg | *Leaves and twigs of Dysoxylum lenticellatum* | J. Nat. Prod. 2011, 74, 2235–2242 |
| **482** | dysolenticin F | 1337973-04-2 |  | Teterpenoid | 0.000121% | 18mg/14.9kg | *Leaves and twigs of Dysoxylum lenticellatum* | J. Nat. Prod. 2011, 74, 2235–2242 |
| **483** | dysolenticin G | 1337973-05-3 |  | Teterpenoid | 0.000027% | 4mg/14.9kg | *Leaves and twigs of Dysoxylum lenticellatum* | J. Nat. Prod. 2011, 74, 2235–2242 |
| **484** | dysolenticin H | 1337973-06-4 |  | Teterpenoid | 0.000309% | 46mg/14.9kg | *Leaves and twigs of Dysoxylum lenticellatum* | J. Nat. Prod. 2011, 74, 2235–2242 |
| **485** | dysolenticin I | 1337973-07-5 |  | Teterpenoid | 0.000034% | 5mg/14.9kg | *Leaves and twigs of Dysoxylum lenticellatum* | J. Nat. Prod. 2011, 74, 2235–2242 |
| **486** | laxiracemosin H | 1241871-28-2 |  | Teterpenoid | 0.000215% | 32mg/14.9kg | *Leaves and twigs of Dysoxylum lenticellatum* | J. Nat. Prod. 2011, 74, 2235–2242 |
| **487** | Obacunone | 751-03-1 |  | Triterpenoids | 0.002682% | 321.8mg/12kg | *root bark of D. dasycarpus* | Zhongyaocai (2014), 37(2), 263-265 |
| **488** | kihadanin D | 73793-68-7 |  | Triterpenoids | 0.000033% | 4mg/12kg | *root bark of D. dasycarpus* | Zhongyaocai (2014), 37(2), 263-265 |
| **489** | kihadanin A | 125276-62-2 |  | Triterpenoids | 0.000139% | 16.7mg/12kg | *root bark of D. dasycarpus* | Zhongyaocai (2014), 37(2), 263-265 |
| **490** | kihadanin B | 73793-68-7 |  | Triterpenoids | 0.000043% | 5.1mg/12kg | *root bark of D. dasycarpus* | Zhongyaocai (2014), 37(2), 263-265 |
| **491** | kihadanin C | 1632410-41-3 |  | Triterpenoids | 0.000025% | 3mg/12kg | *root bark of D. dasycarpus* | Zhongyaocai (2014), 37(2), 263-265 |
| **492** | limonin | 1180-71-8 |  | Triterpenoids | 0.000192% | 23mg/12kg | *root bark of D. dasycarpus* | Zhongyaocai (2014), 37(2), 263-265 |
| **493** | evodol | 22318-10-1 |  | Triterpenoids | 0.000101% | 12.1mg/12kg | *root bark of D. dasycarpus* | Zhongyaocai (2014), 37(2), 263-265 |
| **494** | Ziyuglycoside I | 35286-58-9 |  | Triterpenoids | 0.000210% | 0.000210% | *Ilex paraguariensis* | Journal of the Brazilian Chemical Society 2004, (2), 205-211 |
| **495** | Arjunolic acid | 465-00-9 |  | Triterpenoids | 0.000500% | 0.000500% | *Symplocos lancifolia* | J. Nat. Prod.2011742163-168 |
| **496** | Zeorin | 22570-53-2 |  | Triterpenoids | 0.375000% | 0.375000% | *Lecanora muralis var. muralis* | Chemische Berichte 1961, 94, P614-22 |
| **497** | Laxiracemosin H | 1241871-28-2 |  | Triterpenoids | 0.000300% | 0.000300% | *Dysoxylum laxiracemosum* | Journal of Natural Products 2010, 73(8), 1385-1388 |
| **498** | 3-Oxo-21α-methoxy-24,25,26,27- tetranortirucall-7-ene-23(21)-lactone | 1260173-73-6 |  | Triterpenoids | 0.000230% | 0.000230% | *Aphanamixis grandifolia* | Phytochemistry (2010), 71, (17-18), 2199-2204 |
| **499** | Aphagranin A | 1318173-53-3 |  | Triterpenoids | 0.000240% | 0.000240% | *Aphanamixis grandifolia* | Magnetic Resonance in Chemistry (2011), 49, (7), 450-457 |
| **500** | 3-Oxo-24,25,26,27- tetranortirucall-7-en-23,21-olide | 828935-47-3 |  | Triterpenoids | 0.003000% | 0.003000% | *Amoora dasyclada* | Acta Botanica Sinica 2004, 46(10), P256-1260 |
| **501** | Asiatic acid | 464-92-6 |  | Triterpenoids | 0.247000% | 0.247000% | *Centella asiatic* | Nat Prod Res. 2018 Nov 10:1-7. |
| **502** | Lycernuic acid A | 53755-77-4 |  | Triterpenoids | 0.002300% | 0.002300% | *Lycopodium cernuum* | J. Nat. Prod.2002.657.979-985 |
| **503** | 3,4-Dihydroxybenzoyllupeol | 2231323-99-0 |  | Triterpenoids | 0.000130% | 0.000130% | *Orophea yunnanensis* | Nat Prod Res. 2018 Jun 8:1-6 |
| **504** | Cycloeucalenol | 469-39-6 |  | Triterpenoids | 0.000300% | 0.000300% | *Eucalyptus* | Journal of the Chemical Society 1956, 1384-92 |
| **505** | Piscidinol A | 100198-09-2 |  | Triterpenoids | 0.001450% | 0.001450% | *C. sinensis* | Chemical & Pharmaceutical Bulletin (2007), 55, (10), 1442-1447 |
| **506** | Lupeol caffeate | 103917-26-6 |  | Triterpenoids | 0.004000% | 0.004000% | *Couroupita guianensis Aubl* | Asian Journal of Chemistry (2013), 25, (8), 4559-4562 |
| **507** | Uvaol | 545-46-0 |  | Triterpenoids | 0.000200% | 0.000200% | *Asteraceae Flowers* | Biol. Pharm. Bull. 28(1) 158—160 (2005) |
| **508** | Phellochin | 115334-04-8 |  | Triterpenoids | 0.003000% | 0.003000% | *phellodendron chinense* | Chem.Pharm.Bull.38(6) 1616-1619 (1990) |
| **509** | 1β,​3β,​7β-​Trihydroxylup-​20(29)​-​en-​28-​oic acid | 315224-52-3 |  | Triterpenoids | 0.000100% | 0.000100% | *Schefflera taiwaniana* | Jour nal of the Chi nese Chem i cal So ci ety, 2002, 49, 427-431 |
| **510** | 3-Hydroxy-12-oleanene-23,28-dioic acid | 226562-47-6 |  | Triterpenoids | 0.000100% | 0.000100% | *Schefflera taiwaniana* | Jour nal of the Chi nese Chem i cal So ci ety, 2002, 49, 427-431 |
| **511** | Glochidonol | 23963-54-4 |  | Triterpenoids | 0.000200% | 0.000200% | *Glochidion heyneanum* | Phytochemistry (1988), 27, (11), 3575-8 |
| **512** | Betulin caffeate | 89130-86-9 |  | Triterpenoids | 0.050000% | 0.050000% | *quercus suber* | Journal of Natural Products 1988, V51(2), 217-20 |
| **513** | Hederagonic acid | 466-01-3 |  | Triterpenoids | 0.000082% | 8.2mg/10kg | *root of Pulsatilla cernua* | Zhongcaoyao (2013), 44(23), 3264-3269 |
| **514** | 23-hydroxy-3-oxoolean-12-en-28-oic acid | 59157-83-4 |  | Triterpenoids | 0.000050% | 5mg/10kg | *aerial parts of T. hypoglaucum* | Chemistry of Natural Compounds (2018), 54, (3), 471-474 |
| **515** | 3,23-hydroxy-olean-12-en-28-oic acid | 1392415-94-9 |  | Triterpenoids | 0.000050% | 5mg/10kg | *aerial parts of T. hypoglaucum* | Chemistry of Natural Compounds (2018), 54, (3), 471-474 |
| **516** | α-boswellic acid | 471-66-9 |  | Triterpenoids | 0.000080% | 8mg/10kg | *aerial parts of T. hypoglaucum* | Chemistry of Natural Compounds (2018), 54, (3), 471-474 |
| **517** | wilforlide B | 84104-70-1 |  | Triterpenoids | 0.000070% | 7mg/10kg | *aerial parts of T. hypoglaucum* | Chemistry of Natural Compounds (2018), 54, (3), 471-474 |
| **518** | olean-12-en-28-oic acid | 17990-43-1 |  | Triterpenoids | 0.000058% | 5.8mg/10kg | *aerial parts of T. hypoglaucum* | Chemistry of Natural Compounds (2018), 54, (3), 471-474 |
| **519** | 3α,28-dihydroxy-olean-12-en-29-oic acid | 381691-24-3 |  | Triterpenoids | 0.000060% | 6mg/10kg | *aerial parts of T. hypoglaucum* | Chemistry of Natural Compounds (2018), 54, (3), 471-474 |
| **520** | Odoratone | 16962-90-6 |  | Triterpenoids | 0.000200% | 0.000200% | *A.indica* | Natural Product Research (2019), 33(13), 1903-1908. |
| **521** | Bourjotinolone A | 6985-35-9 |  | Triterpenoids | 0.000100% | 0.000100% | *C. sinensis* | Chemical & Pharmaceutical Bulletin (2007), 55, (10), 1442-1447 |
| **522** | 3β-hydroxy -2-oxoolean-12-ene-22, 29-lactone | 1433665-19-0 |  | Triterpenoids | 0.000022% | 5.6mg/25kg | *stems of C. orbiculatus* | Chinese Journal of Natural Medicines 2012, 10(4): 279−283 |
| **523** | Hedragonic acid | 466-02-4 |  | Triterpenoids | 0.000324% | 81mg/25kg(0.0003%) | *stems of C. orbiculatus* | Chinese Journal of Natural Medicines 2012, 10(4): 279−283 |
| **524** | 3-Epiglochidiol | 29028-10-2 |  | Triterpenoids | 0.002200% | 0.002200% | *glochidion sphareogynum kurz* | planta med 2005;71:208-213 |
| **525** | Wilforol C | 168254-95-3 |  | Triterpenoids | 0.000670% | 0.000670% | *S. taiwaniana* | J. Chin. Chem. Soc.,. 49, 3, 2002 |
| **526** | Simiarenone | 2318-78-7 |  | Triterpenoids | 0.000325% | 13mg/4kg | *Putranjiva roxburghii* | Natural Product Communications . 7 (4) 2012, 511-513 |
| **527** | Putralone | 1404308-17-3 |  | Triterpenoids | 0.000300% | 12mg/4kg | *Putranjiva roxburghii* | Natural Product Communications . 7 (4) 2012, 511-513 |
| **528** | Putrol | 20711-13-1 |  | Triterpenoids | 0.000500% | 20mg/4kg | *Putranjiva roxburghii* | Natural Product Communications . 7 (4) 2012, 511-513 |
| **529** | Putrone | 20711-12-0 |  | Triterpenoids | 0.000325% | 13mg/4kg | *Putranjiva roxburghii* | Natural Product Communications . 7 (4) 2012, 511-513 |
| **530** | Simiarenol acetate | 4965-99-5 |  | Triterpenoids | 0.000425% | 17mg/4kg | *Putranjiva roxburghii* | Natural Product Communications . 7 (4) 2012, 511-513 |
| **531** | Dysolenticin H | 1337973-06-4 |  | Triterpenoids | 0.000034% | （9）5mg/14.9kg | *Dysoxylum lenticellatum* | J. Nat. Prod. 2011, 74, 2235–2242 |
| **532** | Dysolenticin I | 1337973-07-5 |  | Triterpenoids | 0.000034% | 5mg/14.9kg | *Dysoxylum lenticellatum* | J. Nat. Prod. 2011, 74, 2235–2242 |
| **533** | Dysolenticin F | 1337973-04-2 |  | Triterpenoids | 0.000121% | 18mg/14.9kg | *Dysoxylum lenticellatum* | J. Nat. Prod. 2011, 74, 2235–2242 |
| **534** | 24,25-Epoxytirucall-7-en-3,23-dione | 890928-81-1 |  | Triterpenoids | 0.000309% | 46mg/14.9kg | *Dysoxylum lenticellatum* | J. Nat. Prod. 2011, 74, 2235–2242 |
| **535** | Epifriedelanol | 16844-71-6 |  | Triterpenoids | 0.001000% | 100mg/10kg | *root barks of U. davidiana* | Archives of Pharmacal Research (2010), 33, (9), 1307-1315 |
| **536** | β-​Sitosterol | 83-46-5 |  | Triterpenoids | 0.010000% | 1g/10kg | *root barks of U. davidiana* | Archives of Pharmacal Research (2010), 33, (9), 1307-1315 |
| **537** | Maslic acid | 4373-41-5 |  | Triterpenoids | 0.000270% | 27mg/10kg | *root barks of U. davidiana* | Archives of Pharmacal Research (2010), 33, (9), 1307-1315 |
| **538** | (3β,​4β)​-​Stigmast-​5-​ene-​3,​4-​diol | 141602-53-1 |  | Triterpenoids | 0.000080% | 8mg/10kg | *root barks of U. davidiana* | Archives of Pharmacal Research (2010), 33, (9), 1307-1315 |
| **539** | 6-Deoxy-9α-hydroxycedrodorin | 247036-52-8 |  | Triterpenoids | 0.092000% | 4.6 m/5g | *Leaf of C. odorata* | JNP, 1999, 62,9 1263 |
| **540** | cedrodorin | 247050-82-4 |  | Triterpenoids | 0.440000% | 22mg/5g | *Leaf of C. odorata* | JNP, 1999, 62,9 1263 |
| **541** | 6-acetoxycedrodorin | 247050-83-5 |  | Triterpenoids | 0.116000% | 5.8mg/5g | *Leaf of C. odorata* | JNP, 1999, 62,9 1263 |
| **542** | 9-hydroxycedrodorin | 247036-52-8 |  | Triterpenoids | 0.080000% | 4.0mg/5g | *Leaf of C. odorata* | JNP, 1999, 62,9 1263 |
| **543** | Glochidone | 6610-55-5 |  | Triterpenoids | 0.000291% | 12.8mg/4.4kg | *stems of B. microphylla* | Phytochemistry 66 (2005) 2388–2392 |
| **544** | Camelledionol | 81426-90-6 |  | Triterpenoids | 0.000068% | 3mg/4.4kg | *stems of B. microphylla* | Phytochemistry 66 (2005) 2388–2392 |
| **545** | 17β-Hydroxy-3,11,16-dioxo-28-norolean-12-ene | 617722-64-2 |  | Triterpenoids | 0.000068% | 3mg/4.4kg | *stems of B. microphylla* | Phytochemistry 66 (2005) 2388–2392 |
| **546** | Dammaradienyl acetate | 52914-31-5 |  | Triterpenoids | 0.002947% | 30mg/1018g | *root of Microglossa pyrifolia* | Planta Med 2003;69,258-264 |
| **547** | Erythrodiol | 545-48-2 |  | Triterpenoids | 0.000600% | 30mg/5kg | *stems of C. kusanoi* | J. Agric. Food Chem.2010,58,6,3808-3812 |
| **548** | Wilforlide A | 84104-71-2 |  | Triterpenoids | 0.000100% | 10mg/10kg | *root xylem of T. wilfordii* | Chemistry of Natural Compounds,54, 3, 2018 |
| **549** | (3,4-Dihydroxy-5-methoxybenzoyl)taraxerol | 2241135-31-7 |  | Triterpenoids | 0.000108% | 8mg/7.4kg | *leaves and twigs of Orophea yunnanensis* | Natural Product Research (2018) |
| **550** | Ursonic acid | 6246-46-4 |  | Triterpenoids | 0.000667% | 10mg/1.5kg | *whole plants of B. himalaica* | Chemistry of Natural Compounds (2019), 55, (1), 105-106 |
| **551** | 3β-Acetylursolic Acid | 56973-19-4 |  | Triterpenoids | 0.004333% | 65mg/1.5kg | *whole plants of B. himalaica* | Chemistry of Natural Compounds (2019), 55, (1), 105-106 |
| **552** | Triptotriterpenic acid A | 84108-17-8 |  | Triterpenoids | 0.000315% | 63mg/20kg | *air-dried root xylem of T. wilfordii* | Asian Journal of Chemistry (2014), 26, (14), 4344-4346 |
| **553** | Salasone A | 612836-42-7 |  | Triterpenoids | 0.004400% | 123mg 0.0044% | *stems of S. chinensis L.* | J. Nat. Prod.20036691191-1196 |
| **554** | Salasone B | 612836-43-8 |  | Triterpenoids | 0.000500% | 14mg 0.0005% | *stems of S. chinensis L.* | J. Nat. Prod.20036691191-1196 |
| **555** | Salasone C | 612836-44-9 |  | Triterpenoids | 0.001300% | 55mg 0.0013% | *stems of S. chinensis L.* | J. Nat. Prod.20036691191-1196 |
| **556** | Chinensone F | 612836-45-0 |  | Triterpenoids | 0.000600% | 24mg 0.0006% | *stems of S. chinensis L.* | J. Nat. Prod.20036691191-1196 |
| **557** | Tingenine B | 50656-68-3 |  | Triterpenoids | 0.000500% | 19mg 0.0005% | *stems of S. chinensis L.* | J. Nat. Prod.20036691191-1196 |
| **558** | Momordicoside G | 81371-54-2 |  | Triterpenoids | 0.000275% | 13.2mg/4.8kg | *leaf material of M. charantia* | Chemistry & Biodiversity (2012), 9, (2), 428-440 |
| **559** | Phytolaccoside B | 60820-94-2 |  | Triterpenoids | 0.000269% | 13.2mg/4.9kg | *leaf material of M. charantia* | Chemistry & Biodiversity (2012), 9, (2), 428-441 |
| **560** | 5,19-Epoxy-19,25- dimethoxycucurbita-6,23-dien-3-ol | 85372-72-1 |  | Triterpenoids | 0.000233% | 8.4mg/3.6kg | *M.charantia* | J. Nat. Prod.20077081233-1239 |
| **561** | Karavilagenin A | 912329-03-4 |  | Triterpenoids | 0.000056% | 10mg/18kg | *Stems of Momordica charantia* | J. Nat. Prod. 2008, 71, 1327–1330 |
| **562** | Cucurbita-5(10),6,23(E)-triene-3β,25-diol | 1039502-30-1 |  | Triterpenoids | 0.000083% | 15mg/18kg | *Stems of Momordica charantia* | J. Nat. Prod. 2008, 71, 1327–1330 |
| **563** | Cucurbita-5,24-diene-3,7,23-trione | 1039502-32-3 |  | Triterpenoids | 0.000044% | 8mg/18kg | *Stems of Momordica charantia* | J. Nat. Prod. 2008, 71, 1327–1330 |
| **564** | BMT-​17 | 85372-65-2 |  | Triterpenoids | 0.000289% | 52mg/18kg | *Stems of Momordica charantia* | J. Nat. Prod. 2008, 71, 1327–1330 |
| **565** | 3,​7-​Dihydroxy-​25-​methoxycucurbita-​5,​23-​dien-​19-​al | 132587-67-8 |  | Triterpenoids | 0.000039% | 7mg/18kg | *Stems of Momordica charantia* | J. Nat. Prod. 2008, 71, 1327–1330 |
| **566** | 3-Acetoxy-24-hydroxydammara-20,25-diene | 143519-04-4 |  | Triterpenoids | 0.001392% | 100mg/7185g | *Ditrrichia uiscosa* | Phytochemistry (1992), 31, (5), 1826-8. |
| **567** | Melliferone | 377724-68-0 |  | Triterpenoids | 0.000050% | 5mg/10kg | *propolis of Africanized Apis mellifera* | J. Nat. 2001,64,10,1278-1281 |
| **568** | Betulonic acid | 4481-62-3 |  | Triterpenoids | 0.000220% | 22mg/10kg | *propolis of Africanized Apis mellifera* | J. Nat. 2001,64,10,1278-1281 |
| **569** | Sendanolactone | 64929-59-5 |  | Triterpenoids | 0.000019% | 5.8mg/30kg | *bark of M.azedarach* | J. Nat. Prod.2010,73,4,693-697 |
| **570** | 3α,16β,20,22-Tetrahydroxyergosta-5,24(28)-diene | 1222475-76-4 |  | Triterpenoids | 0.000050% | 15.0mg/30kg | *bark of M.azedarach* | J. Nat. Prod.2010,73,4,693-697 |
| **571** | 3-Epiakebonoic acid | 104777-61-9 |  | Triterpenoids | 0.000400% | 4mg/1kg | *Akebia quinata Decne* | CN 103622972 A |
| **572** | Actinidic acid | 341971-45-7 |  | Triterpenoids | 0.000023% | 11mg/48.5kg | *leaves of C. paliurus* | Phytochemistry 151 (2018) 119e127 |
| **573** | 2α, 3β, 23-trihydroxyurs-12, 20(30)-dien-28-oic acid | 143839-01-4 |  | Triterpenoids | 0.000027% | 13mg/48.5kg | *leaves of C. paliurus* | Phytochemistry 151 (2018) 119e127 |
| **574** | Rubuminatus B | 1772614-25-1 |  | Triterpenoids | 0.000024% | 5mg/20.5kg | *R. innominatus* | Phytochemistry 116 (2015) 329–336 |
| **575** | 1β,2α,3α,19α-Tetrahydroxyurs-12-en-23-formyl-28-oic acid | 1772612-70-0 |  | Triterpenoids | 0.000039% | 8mg/20.5kg | *R. innominatus* | Phytochemistry 116 (2015) 329–336 |
| **576** | 1β,2α,3α,19α,23-Pentahydroxyurs-11-en-28-oic acid | 122537-37-5 |  | Triterpenoids | 0.000073% | 15mg/20.5kg | *R. innominatus* | Phytochemistry 116 (2015) 329–336 |
| **577** | 3β,19α-Dihydroxy-1-oxo-olean-12-en-28-oic acid | 877995-09-0 |  | Triterpenoids | 0.000034% | 7mg/20.5kg | *R. innominatus* | Phytochemistry 116 (2015) 329–336 |
| **578** | 2α,3α-Dihydroxyolean-11,13(18)-dien-19b,28-olide | 1542948-65-1 |  | Triterpenoids | 0.000020% | 4mg/20.5kg | *R. innominatus* | Phytochemistry 116 (2015) 329–336 |
| **579** | 3-Dehydro-15-deoxoeucosterol | 81678-46-8 |  | Triterpenoids | 0.001905% | 181mg/9.5kg | whole plan ts of *Scilla scilloides* | Natural Products and Bioprospecting (2015), 5, (5), 263-270. |
| **580** | scillascillol | 2023822-39-9 |  | Triterpenoids | 0.000421% | 40mg/9.5kg | whole plan ts of *Scilla scilloides* | Natural Products and Bioprospecting (2015), 5, (5), 263-270. |
| **581** | scillascillone | 2023822-40-2 |  | Triterpenoids | 0.000516% | 49mg/9.5kg | whole plan ts of *Scilla scilloides* | Natural Products and Bioprospecting (2015), 5, (5), 263-270. |
| **582** | 15-Deoxoeucosterol | 81241-53-4 |  | Triterpenoids | 0.002624% | 6.9mg/263g | bulbs of *Massonia bifolia* | Planta Medica (2018), 84, (9/10), 638-644. |
| **583** | eucosterol | 57092-34-9 |  | Triterpenoids | 0.007439% | 50mg/672.1g | bulbs of *Massonia pustulata (Hyacinthaceae sensu APGII)* | Planta Medica (2018), 84, (9/10), 638-644. |
| **584** | 1-Deacetylnimbolinin B | 76689-98-0 |  | Triterpenoids | 0.001887% | 71.7mg/3.8kg | fruits of *Melia toosendan* | Phytochemistry Letters (2013), 6, (3), 418-424. |
| **585** | nimbolinin A | 220698-26-0 |  | Triterpenoids | 0.010526% | 400mg/3.8kg | fruits of *Melia toosendan* | Phytochemistry Letters (2013), 6, (3), 418-424. |
| **586** | nimbolinin B | 76689-93-5 |  | Triterpenoids | 0.000411% | 15.6mg/3.8kg | fruits of *Melia toosendan* | Phytochemistry Letters (2013), 6, (3), 418-424. |
| **587** | 12-1-O-tigloyl-1-O-deacetyl-nimbolinin B | 1383604-85-0 |  | Triterpenoids | 0.000703% | 26.7mg/3.8kg | fruits of *Melia toosendan* | Phytochemistry Letters (2013), 6, (3), 418-424. |
| **588** | 3-O-Acetyloleanderolide | 62498-83-3 |  | Triterpenoids | 0.000033% | 3mg/9kg | air-dried pericarps of *Akebia trifoliata* | Food Chemistry (2015), 168, 623-629. |
| **589** | 2a,3b,29-trihydroxyolean-12-en-28-oic acid | 1622393-95-6 |  | Triterpenoids | 0.000022% | 2mg/9kg | air-dried pericarps of *Akebia trifoliata* | Food Chemistry (2015), 168, 623-629. |
| **590** | 2a,3b-dihydroxy-23- oxo-olean-12-en-28-oic acid | 1622393-96-7 |  | Triterpenoids | 0.000067% | 6mg/9kg | air-dried pericarps of *Akebia trifoliata* | Food Chemistry (2015), 168, 623-629. |
| **591** | 2a,3b,21b,22a-tetrahydroxyolean-12-en-28,29-dioic acid | 1622393-97-8 |  | Triterpenoids | 0.000356% | 32mg/9kg | air-dried pericarps of *Akebia trifoliata* | Food Chemistry (2015), 168, 623-629. |
| **592** | maslinic acid | 4373-41-5 |  | Triterpenoids | 0.000300% | 27mg/9kg | air-dried pericarps of *Akebia trifoliata* | Food Chemistry (2015), 168, 623-629. |
| **593** | arjunolic acid | 465-00-9 |  | Triterpenoids | 0.002044% | 184mg/9kg | air-dried pericarps of *Akebia trifoliata* | Food Chemistry (2015), 168, 623-629. |
| **594** | oleanolic acid | 508-02-1 |  | Triterpenoids | 0.000278% | 25mg/9kg | air-dried pericarps of *Akebia trifoliata* | Food Chemistry (2015), 168, 623-629. |
| **595** | 3-epi-oleanolic acid | 25499-90-5 |  | Triterpenoids | 0.000033% | 3mg/9kg | air-dried pericarps of *Akebia trifoliata* | Food Chemistry (2015), 168, 623-629. |
| **596** | stachlic acid A | 922143-91-7 |  | Triterpenoids | 0.000867% | 26mg/3kg | air-dried pericarps of *Akebia trifoliata* | Food Chemistry (2015), 168, 623-629. |
| **597** | serratagenic acid | 6488-64-8 |  | Triterpenoids | 0.000038% | 3.4mg/9kg | air-dried pericarps of *Akebia trifoliata* | Food Chemistry (2015), 168, 623-629. |
| **598** | gypsogenic acid | 5143--05-5 |  | Triterpenoids | 0.000078% | 7mg/9kg | air-dried pericarps of *Akebia trifoliata* | Food Chemistry (2015), 168, 623-629. |
| **599** | mesembryanthemoidigenic acid | 4871-87-8 |  | Triterpenoids | 0.000089% | 8mg/9kg | air-dried pericarps of *Akebia trifoliata* | Food Chemistry (2015), 168, 623-629. |
| **600** | Fupenzic acid | 119725-20-1 |  | Triterpenoids | 0.000165% | 15mg/9.1kg | root bark of *Actinidia chinensis* | Pest Management Science (2018), 74, (7), 1630-1636. |
| **601** | 12-Diene-30-oic acid | 39704-66-0 |  | Triterpenoids | 0.000066% | 6mg/9.1kg | root bark of *Actinidia chinensis* | Pest Management Science (2018), 74, (7), 1630-1636. |
| **602** | Phthalic acidPhthalic acid, ester with 3β-​hydroxyolean-​12-​en-​28-​oic acid | 4847-31-8 |  | Triterpenoids | 0.000088% | 8mg/9.1kg | root bark of *Actinidia chinensis* | Pest Management Science (2018), 74, (7), 1630-1636. |
| **603** | 1β-Hydroxy-2-oxopomolic acid | 217466-37-0 |  | Triterpenoids | 0.001043% | 24mg/2.3kgkg | dried leaves of *Rosa woodsii* | J. Nat. Prod. 1998, 61, 1090-1095 |
| **604** | Pomolic Acid | 13849-91-7 |  | Triterpenoids | 0.001435% | 33mg/2.3kg | dried leaves of *Rosa woodsii* | J. Nat. Prod. 1998, 61, 1090-1095 |
| **605** | 2-Oxopomolic Acid | 54963-52-9 |  | Triterpenoids | 0.002348% | 54mg/2.3kg | dried leaves of *Rosa woodsii* | J. Nat. Prod. 1998, 61, 1090-1095 |
| **606** | Jacarandic acid | 53155-25-2 |  | Triterpenoids | 0.005391% | 124mg/2.3kg | dried leaves of *Rosa woodsii* | J. Nat. Prod. 1998, 61, 1090-1095 |
| **607** | Isoarborinol | 5532-41-2 |  | Triterpenoids | 0.000600% | 30mg/5kg | *whole plants of Anoectochilus roxburghii* | Phytochemical Analysis (2008), 19, (5), 438-443. |
| **608** | Sorghumol acetate | 5595-78-8 |  | Triterpenoids | 0.000920% | 23mg/2.5kg | air-dried roots and rhizomes of *Crassocephalum biafrae (Asteraceae)* | Planta Medica (2009), 75, (11), 1258-1261. |
| **609** | fernenol | 4966-00-1 |  | Triterpenoids | 0.001720% | 43mg/2.5kg | air-dried roots and rhizomes of *Crassocephalum biafrae (Asteraceae)* | Planta Medica (2009), 75, (11), 1258-1261. |
| **610** | Meliasenin B | 1221262-77-6 |  | Triterpenoids | 0.002413% | 111mg/4.6kg | air-dried stem bark of *Melia toosendan* | J. Nat. Prod. 2010, 73, 664–668 |
| **611** | meliatoosenin A | 1221262-72-1 |  | Triterpenoids | 0.000109% | 5mg/4.6kg | air-dried stem bark of *Melia toosendan* | J. Nat. Prod. 2010, 73, 664–668 |
| **612** | meliatoosenin B | 1221262-73-2 |  | Triterpenoids | 0.000130% | 6mg/4.6kg | air-dried stem bark of *Melia toosendan* | J. Nat. Prod. 2010, 73, 664–668 |
| **613** | meliatoosenins C | 1221262-74-3 |  | Triterpenoids | 0.000217% | 10mg/4.6kg | air-dried stem bark of *Melia toosendan* | J. Nat. Prod. 2010, 73, 664–668 |
| **614** | meliatoosenins D | 71590-47-1 |  | Triterpenoids | 0.002587% | 119mg/4.6kg | air-dried stem bark of *Melia toosendan* | J. Nat. Prod. 2010, 73, 664–668 |
| **615** | Meliasenin A | 1221262-76-5 |  | Triterpenoids | 0.000152% | 7mg/4.6kg | air-dried stem bark of *Melia toosendan* | J. Nat. Prod. 2010, 73, 664–668 |
| **616** | Meliasenin C | 1221262-78-7 |  | Triterpenoids | 0.000174% | 8mg/4.6kg | air-dried stem bark of *Melia toosendan* | J. Nat. Prod. 2010, 73, 664–668 |
| **617** | Meliasenin D | 1221262-79-8 |  | Triterpenoids | 0.000652% | 30mg/4.6kg | air-dried stem bark of *Melia toosendan* | J. Nat. Prod. 2010, 73, 664–668 |
| **618** | Meliasenin E | 1221262-80-1 |  | Triterpenoids | 0.000391% | 18mg/4.6kg | air-dried stem bark of *Melia toosendan* | J. Nat. Prod. 2010, 73, 664–668 |
| **619** | Meliasenin F | 1221262-81-2 |  | Triterpenoids | 0.000457% | 21mg/4.6kg | air-dried stem bark of *Melia toosendan* | J. Nat. Prod. 2010, 73, 664–668 |
| **620** | Meliasenin G | 1221262-82-3 |  | Triterpenoids | 0.000261% | 12mg/4.6kg | air-dried stem bark of *Melia toosendan* | J. Nat. Prod. 2010, 73, 664–668 |
| **621** | Meliasenin H | 1221262-83-4 |  | Triterpenoids | 0.000391% | 18mg/4.6kg | air-dried stem bark of *Melia toosendan* | J. Nat. Prod. 2010, 73, 664–668 |
| **622** | Pyrocincholic acid methyl ester | 107160-24-7 |  | Triterpenoids | 0.016600% | 160mg/1kg | bark of C*inchona* | Justus Liebigs Annalen der Chemie (1963), 667, 151-63. |
| **623** | 3-O-Acetyloleanolic acid | 4339-72-4 |  | Triterpenoids | 0.000060% | 3mg/5kg | stems of *Lagerstroemia indica* | Archives of Pharmacal Research (2016), 39, (5), 631-635. |
| **624** | Helexin | 465-99-6 |  | Triterpenoids | 0.000080% | 4mg/5kg | stems of *Lagerstroemia indica* | Archives of Pharmacal Research (2016), 39, (5), 631-635. |
| **625** | Urjinolic acid | 465-00-9 |  | Triterpenoids | 0.000040% | 2mg/5kg | stems of *Lagerstroemia indica* | Archives of Pharmacal Research (2016), 39, (5), 631-635. |
| **626** | Maslic acid | 4373-41-5 |  | Triterpenoids | 0.000060% | 3mg/5kg | stems of *Lagerstroemia indica* | Archives of Pharmacal Research (2016), 39, (5), 631-635. |
| **627** | (3β,​4α)​-​3,​23-​Dihydroxy-​1-​oxoolean-​12-​en-​28-​oic acid | 618390-67-3 |  | Triterpenoids | 0.000040% | 2mg/5kg | stems of *Lagerstroemia indica* | Archives of Pharmacal Research (2016), 39, (5), 631-635. |
| **628** | Epifriedelanol acetate | 2259-07-6 |  | Triterpenoids | 0.000279% | 14mg/4.3kg | fresh leaves of *Euphorbia neriifolia* | Natural Product Communications (2012), 7, (11), 1415-1417. |
| **629** | 3β-friedelanol | 19553-18-5 |  | Triterpenoids | 0.001000% | 43mg/4.3kg | fresh leaves of *Euphorbia neriifolia* | Natural Product Communications (2012), 7, (11), 1415-1417. |
| **630** | epitaraxeryl acetate | 20460-35-9 |  | Triterpenoids | 0.000047% | 2mg/4.3kg | fresh leaves of *Euphorbia neriifolia* | Natural Product Communications (2012), 7, (11), 1415-1417. |
| **631** | dammarenediol II acetate | 22558-20-9 |  | Triterpenoids | 0.000100% | 4.3mg/4.3kg | fresh leaves of *Euphorbia neriifolia* | Natural Product Communications (2012), 7, (11), 1415-1417. |
| **632** | 3β-simiarenol | 1615-94-7 |  | Triterpenoids | 0.000305% | 13.1mg/4.3kg | fresh leaves of *Euphorbia neriifolia* | Natural Product Communications (2012), 7, (11), 1415-1417. |
| **633** | 24-oxocycloart-25-en-3β-ol | 2172704-00-4 |  | Triterpenoids | 0.000051% | 2.2mg/4.3kg | fresh leaves of *Euphorbia neriifolia* | Natural Product Communications (2012), 7, (11), 1415-1417. |
| **634** | Sterculin A | 149252-09-5 |  | Triterpenoids | 0.000100% | 4.3mg/4.3kg | fresh leaves of *Euphorbia neriifolia* | Natural Product Communications (2012), 7, (11), 1415-1417. |
| **635** | 29-norcycloartanol | 17320-15-9 |  | Triterpenoids | 0.000035% | 1.5mg/4.3kg | fresh leaves of *Euphorbia neriifolia* | Natural Product Communications (2012), 7, (11), 1415-1417. |
| **636** | 2-Epitormentic acid | 119725-19-8 |  | Triterpenoids | 0.001875% | 60mg/3.2kg | stem bark of *Vitellaria paradoxa (Sapotaceae)* | Medicinal Chemistry Research (2018), 27, (1), 268-277. |
| **637** | 1α,2β,3β,19α-tretrahydroxyurs-12-en-28-oic acid | 2138924-30-6 |  | Triterpenoids | 0.006250% | 200mg/3.2kg | stem bark of *Vitellaria paradoxa (Sapotaceae)* | Medicinal Chemistry Research (2018), 27, (1), 268-277. |
| **638** | β-sitosterol | 83-46-5 |  | Triterpenoids | 100/3200000 | 100mg/3.2kg | stem bark of *Vitellaria paradoxa (Sapotaceae)* | Medicinal Chemistry Research (2018), 27, (1), 268-277. |
| **639** | stigmasterol | 83-48-7 |  | Triterpenoids | 80/3200000 | 80mg/3.2kg | stem bark of *Vitellaria paradoxa (Sapotaceae)* | Medicinal Chemistry Research (2018), 27, (1), 268-277. |
| **640** | Taraxerol acetate | 2189-80-2 |  | Triterpenoids | 55/1500000 | 55mg/1.5kg | roots of *Artemisia incisa* | Records of Natural Products (2015), 9, (3), 294-304. |
| **641** | β-Amyrone | 638-97-1 |  | Triterpenoids | 26/3000000 | 26mg/3kg | stems of *Diospyros morrisiana* | Phytochemistry (1989), 28, (5), 1541-1543. |
| **642** | Methyl 6-acetoxyangolensate | 16566-88-4 |  | Triterpenoids | 0.000162% | 2.1mg/1.3kg | root bark of *Entandrophragma angolense* | Phytochemistry (Elsevier) (2011), 72, (14-15), 1854-1858. |
| **643** | 5β-​Hydroxy-​7-​deacetoxy-​7-​oxogedunin | 1332344-90-7 |  | Triterpenoids | 0.000077% | 1mg/1.3kg | root bark of *Entandrophragma angolense* | Phytochemistry (Elsevier) (2011), 72, (14-15), 1854-1858. |
| **644** | 5,6-dehydro-7-deacetoxy-7-oxogedunin | 1332344-91-8 |  | Triterpenoids | 0.000392% | 5.1mg/1.3kg | root bark of *Entandrophragma angolense* | Phytochemistry (Elsevier) (2011), 72, (14-15), 1854-1858. |
| **645** | Moluccensin O | 1210345-98-4 |  | Triterpenoids | 0.000362% | 4.7mg/1.3kg | root bark of *Entandrophragma angolense* | Phytochemistry (Elsevier) (2011), 72, (14-15), 1854-1858. |
| **646** | entangosin | 1332517-50-6 |  | Triterpenoids | 0.000969% | 12.6mg/1.3kg | root bark of *Entandrophragma angolense* | Phytochemistry (Elsevier) (2011), 72, (14-15), 1854-1858. |
| **647** | methyl angolensate | 2629-14-3 |  | Triterpenoids | 0.005738% | 74.6mg/1.3kg | root bark of *Entandrophragma angolense* | Phytochemistry (Elsevier) (2011), 72, (14-15), 1854-1858. |
| **648** | secomahoganin | 123941-65-1 |  | Triterpenoids | 0.000246% | 3.2mg/1.3kg | root bark of *Entandrophragma angolense* | Phytochemistry (Elsevier) (2011), 72, (14-15), 1854-1858. |
| **649** | Cabralealactone | 19865-87-3 |  | Triterpenoids | 0.000092% | 23.1mg/25kg | stem bark of *Toona sinensis* | Fitoterapia (2016), 112, 97-103. |
| **650** | (5α,​13α,​14β,​17α,​20S)​-​4,​4,​14-​Trimethyl-​3-​oxochol-​7-​en-​24-​oic acid | 2097419-27-5 |  | Triterpenoids | 0.000015% | 3.7mg/25kg | stem bark of *Toona sinensis* | Fitoterapia (2016), 112, 97-103. |
| **651** | (20S)-5α,8α-epidioxy-3-oxo-24-nor-6.9(11)-dien-23-oic acid | 2185049-60-7 |  | Triterpenoids | 0.000045% | 11.3mg/25kg | stem bark of *Toona sinensis* | Fitoterapia (2016), 112, 97-103. |
| **652** | (20S,24R)-epoxydammarane-12.25- diol-3-one | 25279-15-6 |  | Triterpenoids | 0.000360% | 90mg/25kg | stem bark of *Toona sinensis* | Fitoterapia (2016), 112, 97-103. |
| **653** | Ocotillol | 5986-39-0 |  | Triterpenoids | 0.000024% | 6mg/25kg | stem bark of *Toona sinensis* | Fitoterapia (2016), 112, 97-103. |
| **654** | methyl shoreate | 21671-01-2 |  | Triterpenoids | 0.000048% | 12mg/25kg | stem bark of *Toona sinensis* | Fitoterapia (2016), 112, 97-103. |
| **655** | richenone | 134528-34-0 |  | Triterpenoids | 0.000052% | 13mg/25kg | stem bark of *Toona sinensis* | Fitoterapia (2016), 112, 97-103. |
| **656** | cylindrictone D | 1075182-95-4 |  | Triterpenoids | 0.000057% | 14.3mg/25kg | stem bark of *Toona sinensis* | Fitoterapia (2016), 112, 97-103. |
| **657** | Hydroxydammarenone I | 22549-23-1 |  | Triterpenoids | 0.000949% | 237.3mg/25kg | stem bark of *Toona sinensis* | Fitoterapia (2016), 112, 97-103. |
| **658** | hispidol B | 78739-39-6 |  | Triterpenoids | 0.000880% | 220mg/25kg | stem bark of *Toona sinensis* | Fitoterapia (2016), 112, 97-103. |
| **659** | hollongdione | 32206-97-6 |  | Triterpenoids | 0.000054% | 13.5mg/25kg | stem bark of *Toona sinensis* | Fitoterapia (2016), 112, 97-103. |
| **660** | 4,4,14-trimethyl-3-oxo-24-nor- 5α,13α,14β,17α,20S-chol-7-en-23-oic acid | 1346641-36-8 |  | Triterpenoids | 0.000921% | 230.3mg/25kg | stem bark of *Toona sinensis* | Fitoterapia (2016), 112, 97-103. |
| **661** | bourjotinolone B | 6985-31-5 |  | Triterpenoids | 0.000093% | 23.3mg/25kg | stem bark of *Toona sinensis* | Fitoterapia (2016), 112, 97-103. |
| **662** | Methyl 6-hydroxyangolensate | 22255-07-8 |  | Triterpenoids | 0.000789% | 15mg/1.9kg | leaves of *Swietenia mahogani (Meliaceae)* | Phytochemistry 96 (2013) 312–317 |
| **663** | swietephragmin H | 1187316-85-3 |  | Triterpenoids | 0.001921% | 36.5mg/1.9kg | leaves of *Swietenia mahogani (Meliaceae)* | Phytochemistry 96 (2013) 312–317 |
| **664** | swietephragmin I | 1187316-86-4 |  | Triterpenoids | 0.001211% | 23mg/1.9kg | leaves of *Swietenia mahogani (Meliaceae)* | Phytochemistry 96 (2013) 312–317 |
| **665** | 2-hydroxy-6-deacetoxyswietenine | 205518-97-4 |  | Triterpenoids | 0.003184% | 60.5mg/1.9kg | leaves of *Swietenia mahogani (Meliaceae)* | Phytochemistry 96 (2013) 312–317 |
| **666** | swietemahonin G | 128355-19-1 |  | Triterpenoids | 0.000632% | 12mg/1.9kg | leaves of *Swietenia mahogani (Meliaceae)* | Phytochemistry 96 (2013) 312–317 |
| **667** | 7-deacetoxy-7-oxogedunin | 13072-74-7 |  | Triterpenoids | 0.000026% | 0.5mg/1.9kg | leaves of *Swietenia mahogani (Meliaceae)* | Phytochemistry 96 (2013) 312–317 |
| **668** | Khayalenoid E | 1220508-29-1 |  | Triterpenoids | 0.000213% | 17mg/8kg | leaves and twigs of *Khaya senegalensis* | J. Nat. Prod.2013, 76, 3, 327-333 |
| **669** | khayseneganins A | 1415659-20-9 |  | Triterpenoids | 0.000046% | 3.7mg/8kg | leaves and twigs of *Khaya senegalensis* | J. Nat. Prod.2013, 76, 3, 327-333 |
| **670** | khayseneganins B | 1415659-22-1 |  | Triterpenoids | 0.000250% | 20mg/8kg | leaves and twigs of *Khaya senegalensis* | J. Nat. Prod.2013, 76, 3, 327-333 |
| **671** | khayseneganins C | 1415659-23-2 |  | Triterpenoids | 0.000260% | 20.8mg/8kg | leaves and twigs of *Khaya senegalensis* | J. Nat. Prod.2013, 76, 3, 327-333 |
| **672** | khayseneganins D | 1415659-25-4 |  | Triterpenoids | 0.000113% | 9mg/8kg | leaves and twigs of *Khaya senegalensis* | J. Nat. Prod.2013, 76, 3, 327-333 |
| **673** | khayseneganins E | 1415659-27-6 |  | Triterpenoids | 0.000196% | 15.7mg/8kg | leaves and twigs of *Khaya senegalensis* | J. Nat. Prod.2013, 76, 3, 327-333 |
| **674** | khayseneganins F | 1415659-29-8 |  | Triterpenoids | 0.000088% | 7mg/8kg | leaves and twigs of *Khaya senegalensis* | J. Nat. Prod.2013, 76, 3, 327-333 |
| **675** | khayseneganins G | 1415659-31-2 |  | Triterpenoids | 0.000145% | 11.6mg/8kg | leaves and twigs of *Khaya senegalensis* | J. Nat. Prod.2013, 76, 3, 327-333 |
| **676** | khayseneganins H | 1415659-33-4 |  | Triterpenoids | 0.000250% | 20mg/8kg | leaves and twigs of *Khaya senegalensis* | J. Nat. Prod.2013, 76, 3, 327-333 |
| **677** | 3-deacetylkhivorin | 13947-03-0 |  | Triterpenoids | 0.000213% | 17mg/8kg | leaves and twigs of *Khaya senegalensis* | J. Nat. Prod.2013, 76, 3, 327-333 |
| **678** | 1-deacetylkhivorin | 30437-54-8 |  | Triterpenoids | 0.000625% | 50mg/8kg | leaves and twigs of *Khaya senegalensis* | J. Nat. Prod.2013, 76, 3, 327-333 |
| **679** | 7-oxokhivorin | 15004-51-0 |  | Triterpenoids | 0.000188% | 15mg/8kg | leaves and twigs of *Khaya senegalensis* | J. Nat. Prod.2013, 76, 3, 327-333 |
| **680** | swietmanin B | 1197347-40-2 |  | Triterpenoids | 0.000125% | 10mg/8kg | leaves and twigs of *Khaya senegalensis* | J. Nat. Prod.2013, 76, 3, 327-333 |
| **681** | 11α-acetoxy-2α-hydroxy-6- deoxydestigloylswietenine acetate | 1189801-55-5 |  | Triterpenoids | 0.000125% | 10mg/8kg | leaves and twigs of *Khaya senegalensis* | J. Nat. Prod.2013, 76, 3, 327-333 |
| **682** | 3-O-acetylswietenolide | 121825-43-2 |  | Triterpenoids | 0.000100% | 8mg/8kg | leaves and twigs of *Khaya senegalensis* | J. Nat. Prod.2013, 76, 3, 327-333 |
| **683** | khayanone | 345289-50-1 |  | Triterpenoids | 0.000075% | 6mg/8kg | leaves and twigs of *Khaya senegalensis* | J. Nat. Prod.2013, 76, 3, 327-333 |
| **684** | 3-acetoxy-8,14-dien-8,30- seco-khayalactone | 849642-30-4 |  | Triterpenoids | 0.000056% | 4.5mg/8kg | leaves and twigs of *Khaya senegalensis* | J. Nat. Prod.2013, 76, 3, 327-333 |
| **685** | methyl ivorensate | 24022-48-8 |  | Triterpenoids | 0.000250% | 20mg/8kg | leaves and twigs of *Khaya senegalensis* | J. Nat. Prod.2013, 76, 3, 327-333 |
| **686** | khayanolide B | 300833-57-2 |  | Triterpenoids | 0.000038% | 3mg/8kg | leaves and twigs of *Khaya senegalensis* | J. Nat. Prod.2013, 76, 3, 327-333 |
| **687** | 1-O-acetylkhayanolide B | 309946-47-2 |  | Triterpenoids | 0.000321% | 25.7mg/8kg | leaves and twigs of *Khaya senegalensis* | J. Nat. Prod.2013, 76, 3, 327-333 |
| **688** | khayanolide E | 460054-35-7 |  | Triterpenoids | 0.000095% | 7.6mg/8kg | leaves and twigs of *Khaya senegalensis* | J. Nat. Prod.2013, 76, 3, 327-333 |
| **689** | Cabraleahydroxylactone acetate | 35833-70-6 |  | Triterpenoids | 0.003636% | 200mg/5.5kg | leaves and twigs of *Amoora tsangii* | Phytochemistry Letters (2016), 15, 251-255. |
| **690** | Scillascillone | 2023822-40-2 |  | Triterpenoids | 0.000516% | 49mg/9.5kg | whole plan ts of *Scilla scilloides* | Natural Products and Bioprospecting (2015), 5, (5), 263-270. |
| **691** | Scillascillol | 2023822-39-9 |  | Triterpenoids | 0.000421% | 40mg/9.5kg | whole plan ts of *Scilla scilloides* | Natural Products and Bioprospecting (2015), 5, (5), 263-270. |
| **692** | 1-O-Deacetyl-2α-hydroxykhayanolide E | 1189801-51-1 |  | Triterpenoids | 0.000146% | 7mg/4.8kg | stems of *Khaya ivorensis* | Phytochemistry (Elsevier) (2009), 70, (10), 1305-1308. |
| **693** | 22α-Hydroxy-3-oxo-12-ursen-30-oic acid | 173991-81-6 |  | Triterpenoids | 0.000026% | 2.1mg/8kg | stems of *Tripterygium regelii* | Fitoterapia (2016), 113, 69-73. |
| **694** | Moronic acid | 6713-27-5 |  | Triterpenoids | 0.055000% | 220mg/400g | aerial parts of *Schinus lentiscifolius* | Journal of Ethnopharmacology (2013), 148, (2), 486-491. |
| **695** | Seneganolide | 301530-12-1 |  | Triterpenoids | 0.003055% | 27.8mg/910kg | air-dried stem bark of *Khaya senegalensis* | Journal of Applied Entomology (2003), 127, (4), 236-239. |
| **696** | Swietemahalactone | 1514669-21-6 |  | Triterpenoids | 250/5000000 | 250mg/5kg | air-dried powder of leaves and branches of *Swietenia mahagoni* | RSC Advances (2013), 3, (15), 4890-4893. |
| **697** | Tetrahydroxysqualene | 1043629-23-7 |  | Triterpenoids | 0.001657% | 1.8mg/108.6g | air-dried leaves and twigs of *Rhus taitensis* | J. Nat. Prod. 2008, 71, 1623–1624 |
| **698** | Phytolaccagenin | 1802-12-6 |  | Triterpenoids | 1.833333% | 275mg/15g | Dried and ground roots of *Phytolacca dodecandra* | Phytochemistry (1996), 43, (1), 179-182. |
| **699** | Ohchinin acetate | 67023-81-8 |  | Triterpenoids | 0.000049% | 9.6mg/19.7kg | fruits of *Melia azedarach* | Chemistry & Biodiversity (2014), 11, (7), 987-1000. |
| **700** | 21,23:24,25-Diepoxy-21,23- dimethoxytirucall-7-en-3-one | 1351617-74-7 |  | Triterpenoids | 0.000080% | 8mg/10kg | stem bark of *Aphanamixis grandifolia* | Chemistry & Biodiversity (2011), 8, (11), 2025-2034. |
| **701** | Scillascilloside B-1 | 2023822-41-3 |  | Triterpenoids | 0.003063% | 291mg/9.5kg | *Scilla scilloides* | Natural Products and Bioprospecting (2015), 5, (5), 263-270. |
| **702** | Echinocystic acid | 510-30-5 |  | Triterpenoids | 0.097653% | 416mg/426g | bark of *Entada phaseoloides* | Phytochemistry (1987), 26, (10), 2789-96. |
| **703** | Jangomolide | 93767-25-0 |  | Triterpenoids | 0.000300% | 30mg/10kg | fruit of *Evodia rutaecarpa* | Chemical & Pharmaceutical Bulletin (1988), 36, (11), 4453-61. |
| **704** | 3-Epicabraleahydroxylactone | 35833-72-8 |  | Triterpenoids | 0.061176% | 520mg/850g | bark of *Aglaia crassinervia* | Bioorganic & Medicinal Chemistry (2006), 14, (4), 960-972. |
| **705** | 24,25-Dihydroxycycloartan-3-one | 155060-48-3 |  | Triterpenoids | 0.002857% | 20mg/700g | leaves of *Aglaia harmsiana* | Journal of Natural Products (1995),58,(7),1143-1146 |
| **706** | 20,24-Epoxy-24-methoxy- 23(24-25)abeo-dammaran-3-one | 1020074-97-8 |  | Triterpenoids | 0.000037% | 17mg/46kg | twigs of *Aglaia perviridis* | Helvetica Chimica Acta (2008), 91, (2), 333-337. |
| **707** | Semialactone | 366450-46-6 |  | Triterpenoids | 0.009700% | 194mg/2kg | stem bark of *Rhus javanica* | Chemical & Pharmaceutical Bulletin (2001), 49, (8), 1024-1026. |
| **708** | Cycloartane-3,24,25-triol | 57586-98-8 |  | Triterpenoids | 0.000059% | 12.4mg/21kg | branch and leaf of *Abies fargesii* | Phytochemistry (Elsevier) (2016), 130, 301-312. |
| **709** | Urs-12-ene-3β,16β,22α-triol | 1242085-06-8 |  | Triterpenoids | 0.002700% | 2.7mg/100g | *Nardophyllum bryoides* | Phytochemistry 71 (2010) 1395–1399 |
| **710** | Taraxerol | 127-22-0 |  | Triterpenoids | 0.000343% | 5.9mg/1.72kg | *Marchantia paleacea* | Chemistry of Natural Compounds (2018), 54, (3), 541-544. |
| **711** | 20,24-Dihydroxydammar-25-en-3-one | 75069-59-9 |  | Triterpenoids | 0.000093% | 23.2mg/25kg | *Toona sinensis* | Fitoterapia (2016), 112, 97-103. |
| **712** | 5,19-Epoxy-19,25- dimethoxycucurbita-6,23-dien-3-ol | 85372-70-9 |  | Triterpenoids | 0.001477% | 6.5mg/440g | *Momordica foetida* | Phytochemistry (1997), 45, (2), 391-395. |
| **713** | Triptohypol F | 268541-26-0 |  | Triterpenoids | 0.000489% | 13.2mg/2.7kg | *Boswellia carterii* | Journal of Natural Medicines (2011), 65, (1), 129-134. |
| **714** | 1β,2α,3β,19α-Tetrahydroxy-12-ursen-28-oic acid | 113558-03-5 |  | Triterpenoids | 0.005000% | 150mg/3kg | *Mimusops hexandra* | International Journal of Pharmacognosy (1994), 32, (2), 197-200. |
| **715** | Oleana-9(11),12-dien-3β-ol | 94530-87-7 |  | Triterpenoids | 0.000233% | <7mg/3kg | *Maytenus obtusifolia* | Biochemical Systematics and Ecology (2008), 36, (5-6), 500-503. |
| **716** | 25-Hydroxycycloart-23-en-3-one | 148044-47-7 |  | Triterpenoids | 0.002286% | 1.6 mg/70 g (wet weight) | *fungus Glomerella fusarioides* | J. Nat. Prod.2006, 69, 4, 604-607 |
| **717** | Myricadiol | 17884-88-7 |  | Triterpenoids | 0.000500% | 20mg/4kg | *Aralia cordata* | Archives of Pharmacal Research (2006), 29, (7), 548-555. |
| **718** | 5,19-Epoxy-25-methoxycucurbita-6,23-dien-3-ol | 81910-39-6 |  | Triterpenoids | 0.001091% | 4.8mg/440g | *Momordica foetida* | Phytochemistry (1997), 45, (2), 391-395. |
| **719** | Karavilagenin D | 934739-29-4 |  | Triterpenoids | 0.000507% | 71mg/14kg | *Momordica charantia* | Helvetica Chimica Acta (2014), 97, (11), 1546-1554. |
| **720** | sandorinic acid A | 369647-34-7 |  | Triterpenoids | 0.001630% | 5.2mg/319g | *stem bark of Sandoricum indicum* | J. Nat. Prod. 2001, 64, 1243-1245 |
| **721** | sandorinic acid B | 369647-35-8 |  | Triterpenoids | 0.008088% | 25.8mg/319g | *stem bark of Sandoricum indicum* | J. Nat. Prod. 2001, 64, 1243-1245 |
| **722** | sandorinic acid C | 369647-36-9 |  | Triterpenoids | 0.001442% | 4.6mg/319g | *stem bark of Sandoricum indicum* | J. Nat. Prod. 2001, 64, 1243-1245 |
| **723** | katonic acid | 6894-46-8 |  | Triterpenoids | 0.141066% | 450mg/319g | *stem bark of Sandoricum indicum* | J. Nat. Prod. 2001, 64, 1243-1245 |
| **724** | 20- epikoetjapic acid | 270907-28-3 |  | Triterpenoids | 0.017837% | 56.9mg/319g | *stem bark of Sandoricum indicum* | J. Nat. Prod. 2001, 64, 1243-1245 |
| **725** | katonic acid | 6894-46-8 |  | Triterpenoids | 0.015361% | 49mg/319g | *stem bark of Sandoricum indicum* | J. Nat. Prod. 2001, 64, 1243-1245 |
| **726** | 3-oxoolean-12-en-29-oic acid | 76094-29-6 |  | Triterpenoids | 0.003762% | 12mg/319g | *stem bark of Sandoricum indicum* | J. Nat. Prod. 2001, 64, 1243-1245 |
| **727** | 3-Epikatonic acid | 76035-62-6 |  | Triterpenoids | 0.003511% | 11.2mg/319g | *stem bark of Sandoricum indicum* | J. Nat. Prod. 2001, 64, 1243-1245 |
| **728** | Myriceric acid B | 55497-79-5 |  | Triterpenoids | 0.000250% | 50mg/20kg | *stems of Hibiscus taiwanensis* | Chem. Pharm. Bull. 53(1) 56—59 (2005) |
| **729** | myricerol | 55497-67-1 |  | Triterpenoids | 0.000120% | 24mg/20kg | *stems of Hibiscus taiwanensis* | Chem. Pharm. Bull. 53(1) 56—59 (2005) |
| **730** | myriceric acid A | 142877-49-4 |  | Triterpenoids | 0.000020% | 4mg/20kg | *stems of Hibiscus taiwanensis* | Chem. Pharm. Bull. 53(1) 56—59 (2005) |
| **731** | myriceric acid C | 162059-94-1 |  | Triterpenoids | 0.000080% | 16mg/20kg | *stems of Hibiscus taiwanensis* | Chem. Pharm. Bull. 53(1) 56—59 (2005) |
| **732** | 3-oxo-olean-12-en-28-oic acid | 17990-42-0 |  | Triterpenoids | 0.000025% | 5mg/20kg | *stems of Hibiscus taiwanensis* | Chem. Pharm. Bull. 53(1) 56—59 (2005) |
| **733** | stigmasterol | 83-48-7 |  | Triterpenoids | 0.000630% | 126mg/20kg | *stems of Hibiscus taiwanensis* | Chem. Pharm. Bull. 53(1) 56—59 (2005) |
| **734** | hibicusin | 853233-65-5 |  | Triterpenoids | 0.000025% | 5mg/20kg | *stems of Hibiscus taiwanensis* | Chem. Pharm. Bull. 53(1) 56—59 (2005) |
| **735** | uncarinic acid A | 206256-62-4 |  | Triterpenoids | 0.000035% | 7mg/20kg | *stems of Hibiscus taiwanensis* | Chem. Pharm. Bull. 53(1) 56—59 (2005) |
| **736** | uncarinic acid B | 238748-74-8 |  | Triterpenoids | 0.000010% | 2mg/20kg | *stems of Hibiscus taiwanensis* | Chem. Pharm. Bull. 53(1) 56—59 (2005) |
| **737** | Arjunic acid | 31298-06-3 |  | Triterpenoids | 0.000055% | 5.5mg/10kg | *twigs of Euscaphis japonica* | Planta Med 2012; 78: 1584–1590 |
| **738** | vergatic acid | 14356-51-5 |  | Triterpenoids | 0.000021% | 3.5mg/17kg | *twigs of Euscaphis japonica* | Planta Med 2012; 78: 1584–1590 |
| **739** | maslinic acid | 4373-41-5 |  | Triterpenoids | 0.000316% | 60mg/19kg | *twigs of Euscaphis japonica* | Planta Med 2012; 78: 1584–1590 |
| **740** | 2α,3α,23-trihy-droxyolean-12-en-28-oic acid | 102519-34-6 |  | Triterpenoids | 0.000031% | 6.2mg/20kg | *twigs of Euscaphis japonica* | Planta Med 2012; 78: 1584–1590 |
| **741** | 1α,3β-dihydroxy-12-oleanen-28-oic acid | 55497-67-1 |  | Triterpenoids | 0.000012% | 2.7mg/22kg | *twigs of Euscaphis japonica* | Planta Med 2012; 78: 1584–1590 |
| **742** | ilexosapogenin A | 148031-70-3 |  | Triterpenoids | 0.000016% | 3.6mg/23kg | *twigs of Euscaphis japonica* | Planta Med 2012; 78: 1584–1590 |
| **743** | Hop-17(21)-en-3β-ol | 564-14-7 |  | Triterpenoids | 0.000080% | 1.2mg/1.5kg | *rhizomes and roots of Gentiana scabra Bunge* | Phytochemistry 59 (2002) 791–794 |
| **744** | (20S)-dammara-13(17),24- dien-3-one | 138590-98-4 |  | Triterpenoids | 0.000093% | 1.4mg/1.5kg | *rhizomes and roots of Gentiana scabra Bunge* | Phytochemistry 59 (2002) 791–794 |
| **745** | 17b,21b-epoxyhopan-3-one | 131985-08-5 |  | Triterpenoids | 0.000033% | 0.5mg/1.5kg | *rhizomes and roots of Gentiana scabra Bunge* | Phytochemistry 59 (2002) 791–794 |
| **746** | chiratenol | 132865-97-5 |  | Triterpenoids | 0.000080% | 1.2mg/1.5kg | *rhizomes and roots of Gentiana scabra Bunge* | Phytochemistry 59 (2002) 791–794 |
| **747** | hop-17(21)-en-3- one | 471-61-4 |  | Triterpenoids | 0.000067% | 1mg/1.5kg | *rhizomes and roots of Gentiana scabra Bunge* | Phytochemistry 59 (2002) 791–794 |
| **748** | lupeol | 545-47-1 |  | Triterpenoids | 0.000033% | 0.5mg/1.5kg | *rhizomes and roots of Gentiana scabra Bunge* | Phytochemistry 59 (2002) 791–794 |
| **749** | α-amyrin | 638-95-9 |  | Triterpenoids | 0.000031% | 0.5mg/1.6kg | *rhizomes and roots of Gentiana scabra Bunge* | Phytochemistry 59 (2002) 791–794 |
| **750** | Myriceric acid C | 162059-94-1 |  | Triterpenoids | 0.000020% | 4mg/20kg | *stems of Hibiscus taiwanensis* | Chem. Pharm. Bull. 53(1) 56—59 (2005) |
| **751** | 1-O-Deacetylkhayanolide E | 1007387-95-2 |  | Triterpenoids | 0.000240% | 12mg/5kg | *Dried leaves of Swietenia macrophylla* | Natural Product Research Vol. 26, No. 20, October 2012, 1887–1891 |
| **752** | Swietenine J | 1402715-67-6 |  | Triterpenoids | 0.000440% | 22mg/5kg | *Dried leaves of Swietenia macrophylla* | Natural Product Research Vol. 26, No. 20, October 2012, 1887–1891 |
| **753** | 1-O-Acetylkhayanolide A | 345232-57-7 |  | Triterpenoids | 0.000240% | 12mg/5kg | *Dried leaves of Swietenia macrophylla* | Natural Product Research Vol. 26, No. 20, October 2012, 1887–1891 |
| **754** | Khayanolide E | 460054-35-7 |  | Triterpenoids | 0.000820% | 41mg/5kg | *Dried leaves of Swietenia macrophylla* | Natural Product Research Vol. 26, No. 20, October 2012, 1887–1891 |
| **755** | Khayalactone | 203861-84-1 |  | Triterpenoids | 0.000200% | 10mg/5kg | *Dried leaves of Swietenia macrophylla* | Natural Product Research Vol. 26, No. 20, October 2012, 1887–1891 |
| **756** | Khayanone | 345289-50-1 |  | Triterpenoids | 0.000620% | 31mg/5kg | *Dried leaves of Swietenia macrophylla* | Natural Product Research Vol. 26, No. 20, October 2012, 1887–1891 |
| **757** | 1-O-Acetylkhayanolide B | 309946-47-2 |  | Triterpenoids | 0.000240% | 12mg/5kg | *Dried leaves of Swietenia macrophylla* | Natural Product Research Vol. 26, No. 20, October 2012, 1887–1891 |
| **758** | Khayanolide A | 300833-56-1 |  | Triterpenoids | 0.000200% | 10mg/5kg | *Dried leaves of Swietenia macrophylla* | Natural Product Research Vol. 26, No. 20, October 2012, 1887–1891 |
| **759** | Khayanolide B | 300833-57-2 |  | Triterpenoids | 0.000700% | 35mg/5kg | *Dried leaves of Swietenia macrophylla* | Natural Product Research Vol. 26, No. 20, October 2012, 1887–1891 |
| **760** | Olean-12-ene-3β,24-diol | 119318-15-9 |  | Triterpenoids | 0.000050% | 2.4mg/4.8kg | *stem root of C. gabunensis* | Chemical & Pharmaceutical Bulletin (2010), 58, (8), 1100-1102 |
| **761** | α-Boswellic acid | 471-66-9 |  | Triterpenoids | 0.000080% | 8mg/10kg | *aerial parts of T. hypoglaucum* | Chemistry of Natural Compounds (2018), 54, (3), 471-474 |
| **762** | β-Amyrin acetate | 1616-93-9 |  | Triterpenoids | 0.000400% | 40mg/10kg | *roots of Vemonia cinerea Less* | Journal of Natural Products, 47, 368–372. |
| **763** | 29-Hydroxyfriedelan-3-one | 39903-21-4 |  | Triterpenoids | 0.000046% | 7.1mg/15.3kg | *The root bark of Tripterigyum hypoglaucum (Levl.)* | R. Fujita et al. / Phytochemistry 53 (2000) 715±722 |
| **764** | triptohypol D | 268541-24-8 |  | Triterpenoids | 0.000038% | 5.8mg/15.3kg | *The root bark of Tripterigyum hypoglaucum (Levl.)* | R. Fujita et al. / Phytochemistry 53 (2000) 715±722 |
| **765** | triptohypol E | 268541-25-9 |  | Triterpenoids | 0.000099% | 15.1mg/15.3kg | *The root bark of Tripterigyum hypoglaucum (Levl.)* | R. Fujita et al. / Phytochemistry 53 (2000) 715±722 |
| **766** | triptohypol F | 268541-26-0 |  | Triterpenoids | 0.000041% | 6.3mg/15.3kg | *The root bark of Tripterigyum hypoglaucum (Levl.)* | R. Fujita et al. / Phytochemistry 53 (2000) 715±722 |
| **767** | oleanoic acid 3-O-acetate | 25493-91-8 |  | Triterpenoids | 0.000027% | 4.2mg/15.3kg | *The root bark of Tripterigyum hypoglaucum (Levl.)* | R. Fujita et al. / Phytochemistry 53 (2000) 715±722 |
| **768** | triptocallic acid D | 201534-09-0 |  | Triterpenoids | 0.000031% | 4.7mg/15.3kg | *The root bark of Tripterigyum hypoglaucum (Levl.)* | R. Fujita et al. / Phytochemistry 53 (2000) 715±722 |
| **769** | 3-epikatonic acid | 76035-62-6 |  | Triterpenoids | 0.000100% | 15.3mg/15.3kg | *The root bark of Tripterigyum hypoglaucum (Levl.)* | R. Fujita et al. / Phytochemistry 53 (2000) 715±722 |
| **770** | triptocallic acid C | 201534-08-9 |  | Triterpenoids | 0.000055% | 8.4mg/15.3kg | *The root bark of Tripterigyum hypoglaucum (Levl.)* | R. Fujita et al. / Phytochemistry 53 (2000) 715±722 |
| **771** | hypodiol | 198129-86-1 |  | Triterpenoids | 11.5/15300000% | 11.5mg/15.3kg | *The root bark of Tripterigyum hypoglaucum (Levl.)* | R. Fujita et al. / Phytochemistry 53 (2000) 715±722 |
| **772** | polpunonic acid | 33600-93-0 |  | Triterpenoids | 0.000259% | 39.6mg/15.3kg | *The root bark of Tripterigyum hypoglaucum (Levl.)* | R. Fujita et al. / Phytochemistry 53 (2000) 715±722 |
| **773** | celastrol | 34157-83-0 |  | Triterpenoids | 0.001064% | 162.8mg/15.3kg | *The root bark of Tripterigyum hypoglaucum (Levl.)* | R. Fujita et al. / Phytochemistry 53 (2000) 715±722 |
| **774** | Masticadienolic acid | 472-30-0 |  | Triterpenoids | 0.000603% | 41mg/6.8kg | leaves and stems of S. micrantha | Chemical & Pharmaceutical Bulletin (2003), 51, (10), 1174-1176 |
| **775** | Olean-12-ene-3β,11α-diol | 5282-14-4 |  | Triterpenoids | 0.010000% | 10mg/100g | *barks of Platanus acerifolia Willd* | Modern Chemistry & Applications (2016), 4, (1), 178/1-178/3 |
| **776** | betulinic acid | 472-15-1 |  | Triterpenoids | 0.100000% | 1000mg/100g | *barks of Platanus acerifolia Willd* | Modern Chemistry & Applications (2016), 4, (1), 178/1-178/3 |
| **777** | Acetyl betulinaldehyde | 27570-21-4 |  | Triterpenoids | 0.020000% | 20mg/100g | *barks of Platanus acerifolia Willd* | Modern Chemistry & Applications (2016), 4, (1), 178/1-178/3 |
| **778** | olean-12-en-3β,11α,16β-triol-3-O-palmitate | 889686-52-6 |  | Triterpenoids | 0.000143% | 5mg/3.5kg | *Pyrethrum tatsienense* | Pharmazie 61: 70–73 (2006) |
| **779** | α-amyrin | 638-95-9 |  | Triterpenoids | 0.000171% | 6mg/3.5kg | *Pyrethrum tatsienense* | Pharmazie 61: 70–73 (2006) |
| **780** | taraxasterol | 1059-14-9 |  | Triterpenoids | 0.000214% | 75mg/3.5kg | *Pyrethrum tatsienense* | Pharmazie 61: 70–73 (2006) |
| **781** | pseudotaraxasterol | 464-98-2 |  | Triterpenoids | 0.002429% | 85mg/3.5kg | *Pyrethrum tatsienense* | Pharmazie 61: 70–73 (2006) |
| **782** | 3α,22β-Dihydroxyolean-12-en-29-oic acid | 808769-54-2 |  | Triterpenoids | 0.000292% | 28mg/9.6kg | *roots of Celastrus stylosus* | Chem Biodivers. 2015 Aug;12(8):1222-8. |
| **783** | 24-nor-friedelan-6α,10- dihydroxy-1,2-dioxo-4,7-dien-29-oic acid | 1807459-33-1 |  | Triterpenoids | 0.000323% | 31mg/9.6kg | *roots of Celastrus stylosus* | Chem Biodivers. 2015 Aug;12(8):1222-8. |
| **784** | celastrol | 34157-83-0 |  | Triterpenoids | 0.000313% | 30mg/9.6kg | *roots of Celastrus stylosus* | Chem Biodivers. 2015 Aug;12(8):1222-8. |
| **785** | pristimerin | 1258-84-0 |  | Triterpenoids | 0.000333% | 32mg/9.6kg | *roots of Celastrus stylosus* | Chem Biodivers. 2015 Aug;12(8):1222-8. |
| **786** | 23-nor-6- oxodemethyl pristimerol | 118172-80-8 |  | Triterpenoids | 0.000385% | 37mg/9.6kg | *roots of Celastrus stylosus* | Chem Biodivers. 2015 Aug;12(8):1222-8. |
| **787** | wilforol A | 167882-66-8 |  | Triterpenoids | 0.000365% | 35mg/9.6kg | *roots of Celastrus stylosus* | Chem Biodivers. 2015 Aug;12(8):1222-8. |
| **788** | orthosphenic acid | 86632-20-4 |  | Triterpenoids | 0.000177% | 17mg/9.6kg | *roots of Celastrus stylosus* | Chem Biodivers. 2015 Aug;12(8):1222-8. |
| **789** | polpunonic acid | 33600-93-0 |  | Triterpenoids | 0.000271% | 26mg/9.6kg | *roots of Celastrus stylosus* | Chem Biodivers. 2015 Aug;12(8):1222-8. |
| **790** | Triptocallic acid D | 201534-09-0 |  | Triterpenoids | 0.057407% | 31mg/54g | *powdered extract TII of Tripterygium wilfordii* | Phytochemistry (2000), 53, (7), 805-810 |
| **791** | α-Hederin | 27013-91-8 |  | Triterpenoids | 0.373333% | 280mg/75g | *Stem barks of Hedera rhombea* | Phytochemistry, Vol. 29, No. 2, pp. 451452, 1990 |
| **792** | Hederagenin | 465-99-6 |  | Triterpenoids | 0.000034% | 7.1mg/21kg | *twigs of Euscaphis japonica* | Planta Med 2012; 78: 1584–1590 |
| **793** | Triptocallic acid A | 190906-61-7 |  | Triterpenoids | 0.000025% | 2mg/8kg | *stems of Tripterygium regelii* | Fitoterapia 113 (2016) 69–73 |
| **794** | Camaric acid | 146450-83-1 |  | Triterpenoids | 0.011870% | 1187mg/10kg | *Air-dried aerial parts of Lantana camara* | Phytochemistry, Vol. 38, No. 3, pp. 681 685. 1995 |
| **795** | camarinic acid | 163565-67-1 |  | Triterpenoids | 0.001160% | 116mg/10kg | *Air-dried aerial parts of Lantana camara* | Phytochemistry, Vol. 38, No. 3, pp. 681 685. 1996 |
| **796** | oleanolic acid | 508-02-1 |  | Triterpenoids | 0.005410% | 541mg/10kg | *Air-dried aerial parts of Lantana camara* | Phytochemistry, Vol. 38, No. 3, pp. 681 685. 1997 |
| **797** | pomolic acid | 13849-91-7 |  | Triterpenoids | 0.000950% | 95mg/10kg | *Air-dried aerial parts of Lantana camara* | Phytochemistry, Vol. 38, No. 3, pp. 681 685. 1998 |
| **798** | lantanolic acid | 32303-26-7 |  | Triterpenoids | 0.000096% | 9.6mg/10kg | *Air-dried aerial parts of Lantana camara* | Phytochemistry, Vol. 38, No. 3, pp. 681 685. 1999 |
| **799** | lantanilic acid | 60657-41-2 |  | Triterpenoids | 0.026600% | 2660mg/10kg | *Air-dried aerial parts of Lantana camara* | Phytochemistry, Vol. 38, No. 3, pp. 681 685. 2000 |
| **800** | lantic acid | 22626-26-2 |  | Triterpenoids | 0.000675% | 67.5mg/10kg | *Air-dried aerial parts of Lantana camara* | Phytochemistry, Vol. 38, No. 3, pp. 681 685. 2001 |
| **801** | Rubiarbonol B | 130288-60-7 |  | Triterpenoids | 0.000071% | 3.0mg/4.2kg | *roots of Rubia yunnanensis* | J. Nat. Prod. 2002, 65, 1283-1287 |
| **802** | rubiarbonone D | 468732-39-0 |  | Triterpenoids | 0.000076% | 3.2mg/4.2kg | *roots of Rubia yunnanensis* | J. Nat. Prod. 2002, 65, 1283-1287 |
| **803** | rubiarbonone F | 468732-40-3 |  | Triterpenoids | 0.000060% | 2.5mg/4.2kg | *roots of Rubia yunnanensis* | J. Nat. Prod. 2002, 65, 1283-1287 |
| **804** | rubiarboside F | 468732-41-4 |  | Triterpenoids | 0.000131% | 5.5mg/4.2kg | *roots of Rubia yunnanensis* | J. Nat. Prod. 2002, 65, 1283-1287 |
| **805** | rubiarboside G | 468732-42-5 |  | Triterpenoids | 0.000121% | 5.1mg/4.2kg | *roots of Rubia yunnanensis* | J. Nat. Prod. 2002, 65, 1283-1287 |
| **806** | rubiarbonone E | 468732-43-6 |  | Triterpenoids | 0.000071% | 3mg/4.2kg | *roots of Rubia yunnanensis* | J. Nat. Prod. 2002, 65, 1283-1287 |
| **807** | rubiarbonone A | 142778-15-2 |  | Triterpenoids | 0.000119% | 5.0mg/4.2kg | *roots of Rubia yunnanensis* | J. Nat. Prod. 2002, 65, 1283-1287 |
| **808** | rubiarbonone B | 157414-07-8 |  | Triterpenoids | 0.000238% | 10mg/4.2kg | *roots of Rubia yunnanensis* | J. Nat. Prod. 2002, 65, 1283-1287 |
| **809** | rubiarbonol A | 130288-59-4 |  | Triterpenoids | 0.000714% | 30mg/4.2kg | *roots of Rubia yunnanensis* | J. Nat. Prod. 2002, 65, 1283-1287 |
| **810** | rubiarbonol F | 130295-73-7 |  | Triterpenoids | 0.000071% | 3.0mg/4.2kg | *roots of Rubia yunnanensis* | J. Nat. Prod. 2002, 65, 1283-1287 |
| **811** | rubiarbonol G | 142778-14-1 |  | Triterpenoids | 0.000071% | 3.0mg/4.2kg | *roots of Rubia yunnanensis* | J. Nat. Prod. 2002, 65, 1283-1287 |
| **812** | rubiarboside A | 142778-16-3 |  | Triterpenoids | 0.000357% | 15mg/4.2kg | *roots of Rubia yunnanensis* | J. Nat. Prod. 2002, 65, 1283-1287 |
| **813** | 30-Hydroxylup-20(29)-en-3-one | 72944-06-0 |  | Triterpenoids | 0.032795% | 289.7mg/883.36g | *Maytenus imbricata* | HELVETICA CHIMICA ACTA-Vol.88(2005) |
| **814** | 3-oxolup-20(30)-en-29-al | 79875-79-9 |  | Triterpenoids | 0.002038% | 18mg/883.36g | *Maytenus imbricata* | HELVETICA CHIMICA ACTA-Vol.88(2005) |
| **815** | (11α)-11-hydroxylup-20(29)-en-3-one | 71298-27-6 |  | Triterpenoids | 0.002038% | 18mg/883.36g | *Maytenus imbricata* | HELVETICA CHIMICA ACTA-Vol.88(2005) |
| **816** | β-Amyrenonol | 38242-02-3 |  | Triterpenoids | 0.000356% | 3.2mg/900g | *root bark of Maytenus cuzcoina Loesener* | Natural Product Communications Vol. 12 (5) 2017 677 |
| **817** | 3β-hydroxy-olean-9(11):12-diene | 94530-87-7 |  | Triterpenoids | 0.000267% | 2.4mg/900g | *root bark of Maytenus cuzcoina Loesener* | Natural Product Communications Vol. 12 (5) 2017 677 |
| **818** | polpunonic acid | 33600-93-0 |  | Triterpenoids | 0.000689% | 6.2mg/900g | *root bark of Maytenus cuzcoina Loesener* | Natural Product Communications Vol. 12 (5) 2017 677 |
| **819** | 11-oxo-α-amyrin | 2118-90-3 |  | Triterpenoids | 0.000222% | 2mg/900g | *root bark of Maytenus cuzcoina Loesener* | Natural Product Communications Vol. 12 (5) 2017 677 |
| **820** | glutinol | 545-24-4 |  | Triterpenoids | 0.000622% | 5.6mg/900g | *root bark of Maytenus cuzcoina Loesener* | Natural Product Communications Vol. 12 (5) 2017 677 |
| **821** | falcatin A | 1174530-95-0 |  | Triterpenoids | 0.000257% | 9mg/3.5kg | *aerial parts roots of Oxytropis falcat* | J. Nat. Prod.20097281410-1413 |
| **822** | Falcatin B | 1174530-97-2 |  | Triterpenoids | 0.000120% | 4.2mg/3.5kg | *aerial parts roots of Oxytropis falcat* | J. Nat. Prod.20097281410-1413 |
| **823** | 3-Oxo-azukisapogenol | 1174209-16-5 |  | Triterpenoids | 0.000123% | 4.3mg/3.5kg | *aerial parts roots of Oxytropis falcat* | J. Nat. Prod.20097281410-1413 |
| **824** | melilotigenin B | 91269-84-0 |  | Triterpenoids | 0.000057% | 2mg/3.5kg | *aerial parts roots of Oxytropis falcat* | J. Nat. Prod.20097281410-1413 |
| **825** | melilotigenin C | 188970-21-0 |  | Triterpenoids | 0.000074% | 2.6mg/3.5kg | *aerial parts roots of Oxytropis falcat* | J. Nat. Prod.20097281410-1413 |
| **826** | 3β,​24-​Dihydroxyolean-​12-​ene | 119318-15-9 |  | Triterpenoids | 0.000089% | 3.1mg/3.5kg | *aerial parts roots of Oxytropis falcat* | J. Nat. Prod.20097281410-1413 |
| **827** | azukisapogenol | 86425-21-0 |  | Triterpenoids | 0.000243% | 8.5mg/3.5kg | *aerial parts roots of Oxytropis falcat* | J. Nat. Prod.20097281410-1413 |
| **828** | soyasapogenol E | 6750-59-0 |  | Triterpenoids | 0.000391% | 13.7mg/3.5kg | *aerial parts roots of Oxytropis falcat* | J. Nat. Prod.20097281410-1413 |
| **829** | wistariasapogenol B | 121994-07-8 |  | Triterpenoids | 0.000186% | 6.5mg/3.5kg | *aerial parts roots of Oxytropis falcat* | J. Nat. Prod.20097281410-1413 |
| **830** | soyasapogenol B | 595-15-3 |  | Triterpenoids | 0.000429% | 15mg/3.5kg | *aerial parts roots of Oxytropis falcat* | J. Nat. Prod.20097281410-1413 |
| **831** | Olean-12-ene-3,11-dione | 2935-32-2 |  | Triterpenoids | 0.000029% | 3mg/10.3kg | *root of of Indigofera pseudotinctoria* | Zhongguo Zhongyao Zazhi (2010), 35(20), 2708-2711 |
| **832** | β-Amyrenonol acetate | 5356-56-9 |  | Triterpenoids | 0.000029% | 3mg/10.3kg | *root of of Indigofera pseudotinctoria* | Zhongguo Zhongyao Zazhi (2010), 35(20), 2708-2711 |
| **833** | Rehmannic acid | 467-81-2 |  | Triterpenoids | 0.001400% | 14mg/1kg | *aerial parts of Lantana camara L* | Phytochemistry (Elsevier) (2017), 144, 106-112. |
| **834** | 29-Norcycloart-23-ene-3,25-diol | 115040-04-5 |  | Triterpenoids | 0.000333% | 17mg/5.1kg | *Aganosma marginata* | Chinese Traditional and Herbal Drugs, Vol. 46, No.12, pp. 1742-1748, 2015. |
| **835** | 2',3'-Dehydrosalannol | 97411-50-2 |  | Triterpenoids | 0.003188% | 255mg/8kg | *leaves of Azadirachta indica* | Phytochemistry, Vol. 24, No. 4, pp. 866-867, 1985. |
| **836** | Ohchinin | 67023-80-7 |  | Triterpenoids | 0.024250% | 485mg/2kg | *fruit of Melia azadarach L. var. japonica Makino* | Bull.Chem.Soc.Jpn.,56,1139-1142(1983) |
| **837** | Ohchinolal | 86160-86-3 |  | Triterpenoids | 0.019000% | 380mg/2kg | *fruit of Melia azadarach L. var. japonica Makino* | Bull.Chem.Soc.Jpn.,56,1139-1142(1983) |
| **838** | Epitaraxerol | 20460-33-7 |  | Triterpenoids | 0.008222% | 370mg/4.5kg | *leaves of Mallotus apelta* | Arch Pharm Res Vol 27, No 11, 1109-1113, 2004 |
| **839** | 3α-O-acetyl-20(29)-lupen-2α-ol | 1233355-70-8 |  | Triterpenoids | 0.000103% | 20mg/19.5kg | *Salviia trijuga* | J. Nat. Prod. 2010, 73, 1146–1150 |
| **840** | Lup-20(29)-ene-2α,3β-diol | 61448-03-1 |  | Triterpenoids | 0.002000% | 6mg/300g | *roots of Salvia viridis L. cvar. Blue Jeans* | Phytochemistry 108 (2014) 177–188 |
| **841** | 28-Deoxonimbolide | 126005-94-5 |  | Triterpenoids | 0.000128% | 3.2mg/2.5kg | *rhizomes of Arisaema decipiens* | Arch Pharm Res Vol 33, No 11, 1735-1739, 2010 |
| **842** | Mupinensisone | 152253-67-3 |  | Triterpenoids | 0.000400% | 20mg/5kg | *Euonymus mupinensis Loes et Rehd* | Acta Pharmaceutica Sinica 1993;28(9) :684~689 |
| **843** | wilforlide A | 84104-71-2 |  | Triterpenoids | 0.000300% | 15mg/5kg | *Euonymus mupinensis Loes et Rehd* | Acta Pharmaceutica Sinica 1993;28(9) :684~689 |
| **844** | wilforlide B | 84104-70-1 |  | Triterpenoids | 0.000200% | 10mg/5kg | *Euonymus mupinensis Loes et Rehd* | Acta Pharmaceutica Sinica 1993;28(9) :684~689 |
| **845** | Hennadiol | 20065-99-0 |  | Triterpenoids | 0.000133% | 6mg/4.5kg | *leaves of Mallotus apelta* | Arch Pharm Res Vol 27, No 11, 1109-1113, 2004 |
| **846** | 3α~-hydroxyhop-22(29)-ene | 58801-24-4 |  | Triterpenoids | 0.001422% | 64mg/4.5kg | *leaves of Mallotus apelta* | Arch Pharm Res Vol 27, No 11, 1109-1113, 2004 |
| **847** | friedelin | 559-74-0 |  | Triterpenoids | 0.000711% | 32mg/4.5kg | *leaves of Mallotus apelta* | Arch Pharm Res Vol 27, No 11, 1109-1113, 2004 |
| **848** | friedelanol | 5085-72-3 |  | Triterpenoids | 0.001711% | 77mg/4.5kg | *leaves of Mallotus apelta* | Arch Pharm Res Vol 27, No 11, 1109-1113, 2004 |
| **849** | epifriedelanol | 16844-71-6 |  | Triterpenoids | 0.000378% | 17mg/4.5kg | *leaves of Mallotus apelta* | Arch Pharm Res Vol 27, No 11, 1109-1113, 2004 |
| **850** | taraxerone | 514-07-8 |  | Triterpenoids | 0.003022% | 136mg/4.5kg | *leaves of Mallotus apelta* | Arch Pharm Res Vol 27, No 11, 1109-1113, 2004 |
| **851** | Dulcioic acid | 78516-69-5 |  | Triterpenoids | 0.003793% | 110mg/2.9kg | *the fruits of Hippophae rhamnoides* | Natural Product Research Vol. 23, No. 15, 15 October 2009, 1451–1456 |
| **852** | , oleanolic acid | 508-02-1 |  | Triterpenoids | 0.003655% | 106mg/2.9kg | *the fruits of Hippophae rhamnoides* | Natural Product Research Vol. 23, No. 15, 15 October 2009, 1451–1456 |
| **853** | 19--hydroxyursolic acid | 574747-41-4 |  | Triterpenoids | 0.000207% | 6mg/2.9kg | *the fruits of Hippophae rhamnoides* | Natural Product Research Vol. 23, No. 15, 15 October 2009, 1451–1456 |
| **854** | 28-Hydroxy-3-oxoolean-12-en-29-oic acid | 381691-22-1 |  | Triterpenoids | 0.105556% | 57mg/54g | extracts of *T.wilfordii* | Tetrahedron (2001), 57, (40), 8413-8424. |
| **855** | 3-O-p-Coumaroyltormentic acid | 121064-78-6 |  | Triterpenoids | 0.093200% | 932mg/1kg | *leaves of Eriobotrya japonica* | Biol. Pharm. Bull. 28(10) 1995—1999 (2005) |
| **856** | 3-O-trans-p-Coumaroyltormentic acid | 121064-78-6 |  | Triterpenoids | 0.002383% | 71.5mg/3kg | *Goreishi* | Chem. Pharm.Bull.37(3)648-651(1989) |
| **857** | 3-O-cis-p-Coumaroyltormentic acid | 121072-40-0 |  | Triterpenoids | 0.000593% | 23.7mg/4kg | *Goreishi* | Chem. Pharm.Bull.37(3)648-651(1990) |
| **858** | Tormentic Acid | 13850-16-3 |  | Triterpenoids | 0.000204% | 10.2mg/5kg | *Goreishi* | Chem. Pharm.Bull.37(3)648-651(1991) |
| **859** | euscaphic acid | 53155-25-2 |  | Triterpenoids | 0.000117% | 7mg/6kg | *Goreishi* | Chem. Pharm.Bull.37(3)648-651(1992) |
| **860** | jacoumaric acid | 63303-42-4 |  | Triterpenoids | 0.000197% | 13.8mg/7kg | *Goreishi* | Chem. Pharm.Bull.37(3)648-651(1993) |
| **861** | 2α-hydroxyursolic acid | 4547-24-4 |  | Triterpenoids | 0.000191% | 15.3mg/8kg | *Goreishi* | Chem. Pharm.Bull.37(3)648-651(1994) |
| **862** | pomolic acid | 13849-91-7 |  | Triterpenoids | 0.000380% | 34.2mg/9kg | *Goreishi* | Chem. Pharm.Bull.37(3)648-651(1995) |
| **863** | ursolic acid | 77-52-1 |  | Triterpenoids | 0.011233% | 337mg/3kg | *Goreishi* | Chem. Pharm.Bull.37(3)648-651(1989) |
| **864** | maslinic acid | 4373-41-5 |  | Triterpenoids | 0.000357% | 10.7mg/3kg | *Goreishi* | Chem. Pharm.Bull.37(3)648-651(1989) |
| **865** | Madecassic acid | 18449-41-7 |  | Triterpenoids | 0.000106% | 21.2/20kg | *aerial parts of D. superbus* | Chemistry of Natural Compounds (2017), 53, (4), 740-741 |
| **866** | Mangiferolic acid | 4184-34-3 |  | Triterpenoids | 0.002600% | 130mg/5kg | *aerial parts of A. nukiangensis* | Phytochemistry (2014), 106, 116-123 |
| **867** | isomangiferolic acid | 13878-92-7 |  | Triterpenoids | 0.000580% | 29mg/5kg | *aerial parts of A. nukiangensis* | Phytochemistry (2014), 106, 116-124 |
| **868** | neoabiestrine F | 1395049-58-7 |  | Triterpenoids | 0.000460% | 23mg/5kg | *aerial parts of A. nukiangensis* | Phytochemistry (2014), 106, 116-125 |
| **869** | 3-Oxotirucalla-7,24-dien-21-oic acid | 82464-35-5 |  | Triterpenoids | 0.000259% | 7.5mg/2.9kg | *air-dried roots of A. cobbe* | Nat Prod Res. 2019 Jan 19:1-7. |
| **870** | 27-Hydroxymangiferonic acid | 5132-66-1 |  | Triterpenoids | 0.121000% | 121mg/100g | *Propolis* | J. Nat. Prod.2009,72,7,1283-1287 |
| **871** | 27-Hydroxymangiferolic acid | 17983-82-3 |  | Triterpenoids | 0.010800% | 10.8mg/100g | *Propolis* | J. Nat. Prod.2009,72,7,1283-1287 |
| **872** | 23-Hydroxymangiferonic acid | 232266-08-9 |  | Triterpenoids | 0.017500% | 17.5mg/100g | *Propolis* | J. Nat. Prod.2009,72,7,1283-1287 |
| **873** | 21αH-24-Norhopa-4(23),22(29)-diene-3β,6β-diol | 194613-74-6 |  | Triterpenoids | 0.001186% | 33.2mg/2.8kg | *Leaves and trunk wood of Diatenopteryx sorbifolia Radlk.* | J. Nat. Prod.1997,609,909-911 |
| **874** | Isomangiferolic acid | 13878-92-7 |  | Triterpenoids | 0.000293% | 8.2mg/2.8kg | *Leaves and trunk wood of Diatenopteryx sorbifolia Radlk.* | J. Nat. Prod.1997,609,909-911 |
| **875** | Oleanonic acid | 17990-42-0 |  | Triterpenoids | 0.004500% | 45mg/1kg | *aerial parts of Lantana camara L* | Phytochemistry (Elsevier) (2017), 144, 106-112. |
| **876** | β-betulinic acid | 472-15-1 |  | Triterpenoids | 0.000060% | 30mg/50kg | *fruits of C. acuminata Decne* | Nat Prod Res. 2018 Jun 20:1-6 |
| **877** | Ursolic aldehyde | 19132-81-1 |  | Triterpenoids | 0.000014% | 7mg/50kg | *fruits of C. acuminata Decne* | Nat Prod Res. 2018 Jun 20:1-6 |
| **878** | Ursolisome | 77-52-1 |  | Triterpenoids | 0.000013% | 6.7mg/50kg | *fruits of C. acuminata Decne* | Nat Prod Res. 2018 Jun 20:1-6 |
| **879** | α-​Ursolic acid | 989-30-0 |  | Triterpenoids | 0.000088% | 44mg/50kg | *fruits of C. acuminata Decne* | Nat Prod Res. 2018 Jun 20:1-6 |
| **880** | Virgaureagenin B | 508-02-1 |  | Triterpenoids | 0.000022% | 11mg/50kg | *fruits of C. acuminata Decne* | Nat Prod Res. 2018 Jun 20:1-6 |
| **881** | (23R,24S)-23,24,25- trihydroxytirucall-7-ene-3,6-dione | 1270001-74-5 |  | Triterpenoids | 0.000012% | 6mg/50kg | *fruits of C. acuminata Decne* | Nat Prod Res. 2018 Jun 20:1-6 |
| **882** | isofouquierol | 53822-99-4 |  | Triterpenoids | 0.000013% | 6.3mg/50kg | *fruits of C. acuminata Decne* | Nat Prod Res. 2018 Jun 20:1-6 |
| **883** | Cycloartanol | 4657-58-3 |  | Triterpenoids | 0.000125% | 5mg/4kg | *d rhizomes of C. speciosus* | Nat Prod Res. 2018 Jan;32(1):18-22 |
| **884** | 24-methylenecycloartanol | 1449-09-8 |  | Triterpenoids | 0.000305% | 12.2mg/4kg | *d rhizomes of C. speciosus* | Nat Prod Res. 2018 Jan;32(1):18-22 |
| **885** | stigmasterol | 83-48-7 |  | Triterpenoids | 0.000383% | 15.3mg/4kg | *d rhizomes of C. speciosus* | Nat Prod Res. 2018 Jan;32(1):18-22 |
| **886** | Songoroside A | 61617-29-6 |  | Triterpenoids |  | 8.1mg/12kg | *roots of A. tomentosa* | Nat Prod Res. 2019 Feb 27:1-8 |
| **887** | Oleonolic acid | 508-02-1 |  | Triterpenoids | 0.000500% | 12.5mg/2.5kg | *Patrinia scabiosaefolia Fisch.* | Zhongcaoyao (2011), 42(8), 1477-1480 |
| **888** | 3,​11-​Dioxoolean-​12-​en-​28-​oic acid | 94492-08-7 |  | Triterpenoids | 0.000900% | 22.5mg/2.5kg | *Patrinia scabiosaefolia Fisch.* | Zhongcaoyao (2011), 42(8), 1477-1480 |
| **889** | 3-​O-​[α-​L-​Rhamnopyranosyl(1→4)​-​β-​D-​glucopyranosyl]​oleanolic acid 28-​O-​β-​D-​glucopyranosyl ester | 2106839-33-0 |  | Triterpenoids | 0.000500% | 12.5mg.2.5kg | *Patrinia scabiosaefolia Fisch.* | Zhongcaoyao (2011), 42(8), 1477-1480 |
| **890** | 3-oxo-29- hydroxy-olean-12-en-28-oic acid | 1033282-04-0 |  | Triterpenoids | 0.000736% | 18.4mg.2.5kg | *Patrinia scabiosaefolia Fisch.* | Zhongcaoyao (2011), 42(8), 1477-1480 |
| **891** | 11α,12α-Epoxy-3β,23-dihydroxy- 30-norolean-20(29)-en-28,13β-olide | 186140-36-3 |  | Triterpenoids | 0.000059% | 1.6mg/2.73kg | *d roots of P. rockii ssp. rockii* | J. Nat. Prod.201174102116-2121 |
| **892** | 24(31)-Dehydrocarboxyacetylquercinic acid | 127970-62-1 |  | Triterpenoids | 0.000644% | 2.9mg/450g | *fruiting body of Ganoderma applanatum* | J. Nat. Prod.20066981245-1248 |
| **893** | 3α,16α-Dihydroxylanosta-7,9(11),24-trien-21-oic acid | 76390-66-2 |  | Triterpenoids | 0.000756% | 3.4mg/450g | *fruiting body of Ganoderma applanatum* | J. Nat. Prod.20066981245-1248 |
| **894** | 16α-Hydroxy-3-oxolanosta-7,9(11),24-trien-21-oic acid | 862109-64-6 |  | Triterpenoids | 0.001200% | 5.4mg/450g | *fruiting body of Ganoderma applanatum* | J. Nat. Prod.20066981245-1248 |
| **895** | Gynosaponin I | 1207861-69-5 |  | Triterpenoids | 0.001600% | 80mg/5.0kg | *arial parts of G. pentaphyllum* | Helvetica Chimica Acta (2009), 92, (12), 2737-2745 |
| **896** | Lup-20(29)-ene-3β,23-diol | 163060-07-9 |  | Triterpenoids | 0.002750% | 22mg/800g | *Leaves of C. ternata Kunth* | Planta Medica (2012), 78(18), 1983 |
| **897** | 23-Hydroxybetulin | 84414-40-4 |  | Triterpenoids | 0.000028% | 1.4mg/5kg | *leaves, twigs, and fruits of B. cochinchinensis* | Planta Medica (2015), 81, (12/13), 1133-1140 |
| **898** | Acantrifoic acid A | 654663-85-1 |  | Triterpenoids | 0.000811% | 30mg/3.7kg | *leaves of A. trifoliatus* | Chemical & Pharmaceutical Bulletin (2003), 51, (12), 1432-1435 |
| **899** | Dammarenediol II 3-O-caffeate | 171438-55-4 |  | Triterpenoids | 0.005050% | 101mg/2kg | *leaves of B.ermanii* | Chemical & Pharmaceutical Bulletin (1995), 43, (11), 1937-42 |
| **900** | Lupeolic acid | 87355-32-6 |  | Triterpenoids | 0.083333% | 25mg/30g | *Erytrean-type resin* | Phytochemistry (Elsevier) (2003), 62, (4), 537-541 |
| **901** | 16-Deoxysaikogenin F | 57475-62-4 |  | Triterpenoids | 0.000070% | 8.7mg/12.5kg | *aerial parts of Buddleja asiatica* | Zeitschrift fuer Naturforschung, B: Chemical Sciences (2008), 63, (7), 915-919 |
| **902** | pomolic acid | 13849-91-7 |  | Triterpenoids | 0.000426% | 6mg/1.408kg | *fruits and leaves of R. allegheniensis* | Natural Product Research (2014), 28, (24), 2347-2350. |
| **903** | tormentic acid | 13850-16-3 |  | Triterpenoids | 0.001550% | 16mg/1.032kg | *fruits and leaves of R. allegheniensis* | Natural Product Research (2014), 28, (24), 2347-2350. |
| **904** | euscaphic acid | 53155-25-2 |  | Triterpenoids | 0.001938% | 20mg/1.032kg | *fruits and leaves of R. allegheniensis* | Natural Product Research (2014), 28, (24), 2347-2350. |
| **905** | 1β-hydroxyeuscaphic acid | 120211-98-5 |  | Triterpenoids | 0.001938% | 20mg/1.032kg | *fruits and leaves of R. allegheniensis* | Natural Product Research (2014), 28, (24), 2347-2350. |
| **906** | myrianthic acid | 89786-84-5 |  | Triterpenoids | 0.002519% | 26mg/1.032kg | *fruits and leaves of R. allegheniensis* | Natural Product Research (2014), 28, (24), 2347-2350. |
| **907** | ziyu glycoside II | 35286-61-4 |  | Triterpenoids | 0.001163% | 12mg/1.032kg | *fruits and leaves of R. allegheniensis* | Natural Product Research (2014), 28, (24), 2347-2350. |
| **908** | sericic acid | 55306-03-1 |  | Triterpenoids | 0.001938% | 20mg/1.032kg | *fruits and leaves of R. allegheniensis* | Natural Product Research (2014), 28, (24), 2347-2350. |
| **909** | 19-hydroxy-2,3-secours-12-ene-2,3,28-trioic acid 3-methyl ester | 135203-39-3 |  | Triterpenoids | 0.009399% | 97mg/1.032kg | *fruits and leaves of R. allegheniensis* | Natural Product Research (2014), 28, (24), 2347-2350. |
| **910** | 6β-hydroxy-3-oxoolean-12- en-27-oic acid | 1528752-47-7 |  | Triterpenoids | 0.013680% | 684mg/5kg | *whole plant of C. carnosum* | Fitoterapia. 2013 Mar;85:119-24. |
| **911** | 3β,​21α-​Dihydroxyolean-​12-​en-​27-​oic acid | 1528752-48-8 |  | Triterpenoids | 0.000440% | 22mg/5kg | *whole plant of C. carnosum* | Fitoterapia. 2013 Mar;85:119-24. |
| **912** | Astilbic acid | 41411-00-1 |  | Triterpenoids | 0.003600% | 180mg/5kg | *whole plant of C. carnosum* | Fitoterapia. 2013 Mar;85:119-24. |
| **913** | 3β,24β-dihydroxyolean-12-en-27-oic acid | 461044-01-9 |  | Triterpenoids | 0.010880% | 544mg/5kg | *whole plant of C. carnosum* | Fitoterapia. 2013 Mar;85:119-24. |
| **914** | 3-oxoolean- 12-en-27-oic acid | 86747-46-8 |  | Triterpenoids | 0.010800% | 540mg/5kg | *whole plant of C. carnosum* | Fitoterapia. 2013 Mar;85:119-24. |
| **915** | 3β-hydroxy-olean-12-en-27-oic acid | 24778-48-1 |  | Triterpenoids | 0.000246% | 12.3g/5kg | *whole plant of C. carnosum* | Fitoterapia. 2013 Mar;85:119-24. |
| **916** | Longipedlactone J | 1011762-93-8 |  | Triterpenoids | 0.000040% | 4mg/10kg | *d stems of K. ananosma* | J. Nat. Prod.201073112-16 |
| **917** | Rotundic acid | 20137-37-5 |  | Triterpenoids | 0.000745% | 149mg/20kg | *dried leaves of I. chinensi* | hytochemistry (2018), 148, 113-121 |
| **918** | Glutinol acetate | 6426-44-4 |  | Triterpenoids | 0.000442% | 19mg/4.3kg | *fresh leaves of E. neriifolia,* | Nat Prod Commun. 2012 Nov;7(11):1415-7. |
| **919** | 3β-acetoxy friedelane | 2259--07-6 |  | Triterpenoids | 0.000326% | 14mg/4.3kg | *fresh leaves of E. neriifolia,* | Nat Prod Commun. 2012 Nov;7(11):1415-7. |
| **920** | friedelin | 559-74-0 |  | Triterpenoids | 0.000223% | 9.6mg/4.3kg | *fresh leaves of E. neriifolia,* | Nat Prod Commun. 2012 Nov;7(11):1415-7. |
| **921** | glutinone | 508-09-8 |  | Triterpenoids | 0.000095% | 4.1mg/4.3kg | *fresh leaves of E. neriifolia,* | Nat Prod Commun. 2012 Nov;7(11):1415-7. |
| **922** | glutin-5-en-3β-ol | 545-24-4 |  | Triterpenoids | 0.000472% | 20.3mg/4.3kg | *fresh leaves of E. neriifolia,* | Nat Prod Commun. 2012 Nov;7(11):1415-7. |
| **923** | glutinol acetate | 6426-44-4 |  | Triterpenoids | 0.000442% | 19mg/4.3kg | *fresh leaves of E. neriifolia,* | Nat Prod Commun. 2012 Nov;7(11):1415-7. |
| **924** | lupenone | 1617-70-5 |  | Triterpenoids | 0.000095% | 4.1mg/4.3kg | *fresh leaves of E. neriifolia,* | Nat Prod Commun. 2012 Nov;7(11):1415-7. |
| **925** | epitaraxerol | 20460-33-7 |  | Triterpenoids | 0.000535% | 23mg/4.3kg | *fresh leaves of E. neriifolia,* | Nat Prod Commun. 2012 Nov;7(11):1415-7. |
| **926** | epitaraxeryl acetate | '20460-33-7 |  | Triterpenoids | 0.000465% | 20mg/4.3kg | *fresh leaves of E. neriifolia,* | Nat Prod Commun. 2012 Nov;7(11):1415-7. |
| **927** | taraxeryl acetate | 2189-80-2 |  | Triterpenoids | 0.000163% | 7mg/4.3kg | *fresh leaves of E. neriifolia,* | Nat Prod Commun. 2012 Nov;7(11):1415-7. |
| **928** | β-amyrin | 559-70-6 |  | Triterpenoids | 0.000100% | 4.3mg/4.3kg | *fresh leaves of E. neriifolia,* | Nat Prod Commun. 2012 Nov;7(11):1415-7. |
| **929** | β-amyrin acetate | 1616-93-9 |  | Triterpenoids | 0.000100% | 4.3mg/4.3kg | *fresh leaves of E. neriifolia,* | Nat Prod Commun. 2012 Nov;7(11):1415-7. |
| **930** | dammarenediol II acetate | 22558-20-9 |  | Triterpenoids | 0.000100% | 4.3mg/4.3kg | *fresh leaves of E. neriifolia,* | Nat Prod Commun. 2012 Nov;7(11):1415-7. |
| **931** | cabraleadiol monoacetate | 67253-01-4 |  | Triterpenoids | 0.000107% | 4.6mg/4.3kg | *fresh leaves of E. neriifolia,* | Nat Prod Commun. 2012 Nov;7(11):1415-7. |
| **932** | simiarenone | 2318-78-7 |  | Triterpenoids | 0.000174% | 7.5mg/4.3kg | *fresh leaves of E. neriifolia,* | Nat Prod Commun. 2012 Nov;7(11):1415-7. |
| **933** | cycloartanol | 4657-58-3 |  | Triterpenoids | 0.000100% | 4.3mg/4.3kg | *fresh leaves of E. neriifolia,* | Nat Prod Commun. 2012 Nov;7(11):1415-7. |
| **934** | (23Z)-cycloart-23-ene-3β,25-diol | 149252-09-5 |  | Triterpenoids | 0.000100% | 4.3mg/4.3kg | *fresh leaves of E. neriifolia,* | Nat Prod Commun. 2012 Nov;7(11):1415-7. |
| **935** | cycloeucalenol | 469-39-6 |  | Triterpenoids | 0.000095% | 4.1mg/4.3kg | *fresh leaves of E. neriifolia,* | Nat Prod Commun. 2012 Nov;7(11):1415-7. |
| **936** | 29-norcycloartanol | 17320-15-9 |  | Triterpenoids | 0.000035% | 1.5mg/4.3kg | *fresh leaves of E. neriifolia,* | Nat Prod Commun. 2012 Nov;7(11):1415-7. |
| **937** | Marsformoxide B | 2111-46-8 |  | Triterpenoids | 0.000078% | 15.6mg/20kg | *aerial parts of C. setosum* | Zhongguo Zhongyao Zazhi (2012), 37(7), 951-955. |
| **938** | Barbinervic acid | 64199-78-6 |  | Triterpenoids | 0.000392% | 24.5mg/6.28kg | *leaves of C. barbinervis* | hemical & Pharmaceutical Bulletin (2013), 61, (5), 581-586 |
| **939** | ryobunin A | 1622394-63-1 |  | Triterpenoids | 0.000135% | 8.5mg/6.28kg | *leaves of C. barbinervis* | hemical & Pharmaceutical Bulletin (2013), 61, (5), 581-586 |
| **940** | ryobunin B | 1622394-64-2 |  | Triterpenoids | 0.000135% | 8.5mg/6.28kg | *leaves of C. barbinervis* | hemical & Pharmaceutical Bulletin (2013), 61, (5), 581-586 |
| **941** | ryobunin C | 1622394-65-3 |  | Triterpenoids | 0.000438% | 27.5mgh/6.28kg | *leaves of C. barbinervis* | hemical & Pharmaceutical Bulletin (2013), 61, (5), 581-586 |
| **942** | clethric acid | 68800-57-7 |  | Triterpenoids | 0.000347% | 21.8mg/6.28kg | *leaves of C. barbinervis* | hemical & Pharmaceutical Bulletin (2013), 61, (5), 581-586 |
| **943** | barbinervic acid | 64199-78-6 |  | Triterpenoids | 0.000390% | 24.5mg/6.28kg | *leaves of C. barbinervis* | hemical & Pharmaceutical Bulletin (2013), 61, (5), 581-586 |
| **944** | kakisaponin A | 1003864-78-5 |  | Triterpenoids | 0.000532% | 33.4mg/6.28kg | *leaves of C. barbinervis* | hemical & Pharmaceutical Bulletin (2013), 61, (5), 581-586 |
| **945** | 11α,12α-Oxidotaraxerol palmitate | 495389-95-2 |  | Triterpenoids | 0.000292% | 7.6mg/2.6kg | *leaves of E. passerinum* | Journal of the Brazilian Chemical Society (2002), 13, (5), 669-673 |
| **946** | β-Amyrin palmitate | 5973-06--8 |  | Triterpenoids | 0.001685% | 43.8mg/2.6kg | *leaves of E. passerinum* | Journal of the Brazilian Chemical Society (2002), 13, (5), 669-673 |
| **947** | taraxasterol | 1059-14-9 |  | Triterpenoids | 0.001250% | 125mg/10kg | *Saussurea nivea* | Zhongcaoyao (2011), 42(10), 1913-1916 |
| **948** | ussuriensin A | 1030609-73-4 |  | Triterpenoids | 0.000180% | 18mg/10kg | *Saussurea nivea* | Zhongcaoyao (2011), 42(10), 1913-1916 |
| **949** | ussuriensin B | 946082-54-8 |  | Triterpenoids | 0.000150% | 15mg/10kg | *Saussurea nivea* | Zhongcaoyao (2011), 42(10), 1913-1916 |
| **950** | 3β-hydroxy-11α, 12α-epoxy-friedo-olean-14-enyl palmitate | 422308-58-5 |  | Triterpenoids | 0.000420% | 42mg/10kg | *Saussurea nivea* | Zhongcaoyao (2011), 42(10), 1913-1916 |
| **951** | Myrianthic acid | 89786-84-5 |  | Triterpenoids | 0.000300% | 30mg/10kg | leaves of *E. japonica* | Chemistry of Natural Compounds (2019), 55(1), 169-171 |
| **952** | Jacoumaric Acid | 63303-42-4 |  | Triterpenoids | 0.000160% | 16mg/10kg | The leaves of *Eriobotrya japonica* | Chemistry of Natural Compounds (2019), 55, (1), 169-17 |
| **953** | 3-O-trans-Feruloyl Euscaphic Acid | 184424-55-3 |  | Triterpenoids | 0.000400% | 40mg/10kg | *The leaves of Eriobotrya japonica* | Chemistry of Natural Compounds (2019), 55, (1), 169-17 |
| **954** | Euscaphic Acid | 53155-25-2 |  | Triterpenoids | 0.002000% | 200mg/10kg | *The leaves of Eriobotrya japonica* | Chemistry of Natural Compounds (2019), 55, (1), 169-17 |
| **955** | Ursolic Acid | 77-52-1 |  | Triterpenoids | 0.001500% | 150mg/10kg | *The leaves of Eriobotrya japonica* | Chemistry of Natural Compounds (2019), 55, (1), 169-17 |
| **956** | Corosolic Acid | 4547-24-4 |  | Triterpenoids | 0.000320% | 32mg/10kg | *The leaves of Eriobotrya japonica* | Chemistry of Natural Compounds (2019), 55, (1), 169-17 |
| **957** | Arjunic Acid | 31298-06-3 |  | Triterpenoids | 0.000200% | 20mg/10kg | *The leaves of Eriobotrya japonica* | Chemistry of Natural Compounds (2019), 55, (1), 169-17 |
| **958** | 2α,3α-Dihydroxy-12-ursen-28-oic Acid | 73584-74-4 |  | Triterpenoids | 0.000230% | 23mg/10kg | *The leaves of Eriobotrya japonica* | Chemistry of Natural Compounds (2019), 55, (1), 169-17 |
| **959** | Cauloside A | 17184-21-3 |  | Triterpenoids | 0.000920% | 23mg/2.5kg | *Whole plants of C. tangutica* | Phytochemistry (Elsevier) (2016), 130, 228-237 |
| **960** | 3-O-Coumaroylarjunolic acid | 171864-20-3 |  | Triterpenoids | 0.000150% | 15mg/10kg | *Roots of Rhodomyrtus tomentosa* | Youji Huaxue (2013), 33(6), 1304-1308 |
| **961** | 3-O-Coumaroylasiatic acid | 143773-52-8 |  | Triterpenoids | 0.000148% | 14.8mg/10kg | *Roots of Rhodomyrtus tomentosa* | Youji Huaxue (2013), 33(6), 1304-1308 |
| **962** | Lupeol palmitate | 32214-80-5 |  | Triterpenoids | 0.006152% | 203mg/3.3kg | *leaves of Globimetula braunii.* | Nat Prod Res. 2019 Mar 31:1-8 |
| **963** | 30-Oxopseudotaraxasterol | 160481-71-0 |  | Triterpenoids | =6/3125000 | 6mg/3.125kg | *P. burchardi* | Chemistry & Biodiversity (2011), 8, (11), 2080-2089 |
| **964** | Toosendanin | 58812-37-6 |  | Triterpenoids | 0.014667% | 22mg/150g | *Fructus Toosendan* | Zhongnan Yaoxue (2016), 14(4), 395-397 |
| **965** | taraxeryl acetate | 2189-80-2 |  | Triterpenoids | 0.000514% | 19mg/3.7kg | *aerial parts of C. clematidea* | Journal of Natural Medicines (2008), 62, (2), 236-238 |
| **966** | 3β-acetoxyoleanan-12-one | 5916-19-8 |  | Triterpenoids | 0.000541% | 20mg/3.7kg | *aerial parts of C. clematidea* | Journal of Natural Medicines (2008), 62, (2), 236-238 |
| **967** | Rubiprasin B | 125263-66-3 |  | Triterpenoids | 0.000108% | 4mg/3.7kg | *aerial parts of C. clematidea* | Journal of Natural Medicines (2008), 62, (2), 236-238 |
| **968** | Ginsenoside Rb1 | 41753-43-9 |  | Triterpenoids | 0.880000% | 0.880000% | *Panax ginseng* |  |
| **969** | Quillaic acid | 631-01-6 |  | Triterpenoids | 0.000538% | 4mg/744g | *roots of G. trichotoma Wend* | Phytochemistry Letters (2012), 5, (4), 752-755 |
| **970** | gypsogenin | 639-14-5 |  | Triterpenoids | 0.000806% | 6mg/744g | *roots of G. trichotoma Wend* | Phytochemistry Letters (2012), 5, (4), 752-755 |
| **971** | gypsogenic acid | ‘5143-05-5 |  | Triterpenoids | 0.006720% | 50mg/744g | *roots of G. trichotoma Wend* | Phytochemistry Letters (2012), 5, (4), 752-755 |
| **972** | gypsogenin | 639-14-5 |  | Triterpenoids | 0.000128% | 12.8mg/10kg | *Psammosilene tunicoides* | Zhongguo Shiyan Fangjixue Zazhi (2012), 18(14), 92-94 |
| **973** | α-spinasterol | 481-18-5 |  | Triterpenoids | 0.000200% | 20mg/10kg | *Psammosilene tunicoides* | Zhongguo Shiyan Fangjixue Zazhi (2012), 18(14), 92-94 |
| **974** | daucosterol | 474-58-8 |  | Triterpenoids | 0.000139% | 13.9mg.10kg | *Psammosilene tunicoides* | Zhongguo Shiyan Fangjixue Zazhi (2012), 18(14), 92-94 |
| **975** | Salaspermic acid | 71247-78-4 |  | Triterpenoids | 0.006040% | 302mg/5kg | *Kokoona ochracea stem bark* | Journal of Natural Products Vol. 57, No. l,pp. 1-8, January 1994 |
| **976** | Tingenone | 50802-21-6 |  | Triterpenoids | 0.000028% | 1.42mg/5kg | *Kokoona ochracea stem bark* | Journal of Natural Products Vol. 57, No. l,pp. 1-8, January 1994 |
| **977** | 20-hydroxy-20-epi-tingenone | 154346-86-8 |  | Triterpenoids | 0.000860% | 43mg/5kg | *Kokoona ochracea stem bark* | Journal of Natural Products Vol. 57, No. l,pp. 1-8, January 1994 |
| **978** | celastrol | 34157-83-0 |  | Triterpenoids | 0.001400% | 70mg/5kg | *Kokoona ochracea stem bark* | Journal of Natural Products Vol. 57, No. l,pp. 1-8, January 1994 |
| **979** | Gardenolic acid B | 108864-53-5 |  | Triterpenoids | 0.000188% | 15mg/8kg | *KleinhoWia hospita Leaves and twigs* | J. Nat. Prod. 2009, 72, 1102–1105 |
| **980** | Isolimonexic acid | 73904-93-5 |  | Triterpenoids | 0.000750% | 30mg/4kg | *Euodia daniellii leaves and fruits* | Arch Pharm Res Vol 25, No 6, 824-830, 2002 |
| **981** | Ganoderal A | 104700-98-3 |  | Triterpenoids | 0.070000% | 7mg/10g | *10g of 70% mathanol extract* | Chem. Pharm. Bull.34(7) 3025-3028(1986) |
| **982** | Virgatic acid | 14356-51-5 |  | Triterpenoids | 0.015000% | 600mg/4kg | *Salvia virgata dried aria1 parts* | Phyrochemwry, 1976, Vol. 15, pp. 309-311 |
| **983** | Triptocalline A | 201534-10-3 |  | Triterpenoids | 0.000019% | 1.5mg/8kg | *stems of Tripterygium regelii* | Fitoterapia 113 (2016) 69–73 |
| **984** | Triregelolide A | 2205911-82-4 |  | Triterpenoids | 0.000013% | 1mg/8kg | *stems of Tripterygium regelii* | Fitoterapia 113 (2016) 69–73 |
| **985** | Triregelolide B | 2205911-83-5 |  | Triterpenoids | 0.000008% | 0.6mg/8kg | *stems of Tripterygium regelii* | Fitoterapia 113 (2016) 69–73 |
| **986** | Triregeloic acid | 2205911-84-6 |  | Triterpenoids | 0.000038% | 3mg/8kg | *stems of Tripterygium regelii* | Fitoterapia 113 (2016) 69–73 |
| **987** | NST6A | 2205911-85-7 |  | Triterpenoids | 0.000025% | 2mg/8kg | *stems of Tripterygium regelii* | Fitoterapia 113 (2016) 69–73 |
| **988** | celastrol | 34157-83-0 |  | Triterpenoids | 0.000750% | 60mg/8kg | *stems of Tripterygium regelii* | Fitoterapia 113 (2016) 69–73 |
| **989** | 22β-hydroxytingenone | 50656-68-3 |  | Triterpenoids | 0.000054% | 4.3mg/8kg | *stems of Tripterygium regelii* | Fitoterapia 113 (2016) 69–73 |
| **990** | polpunonic acid | 33600-93-0 |  | Triterpenoids | 0.000063% | 5mg/8kg | *stems of Tripterygium regelii* | Fitoterapia 113 (2016) 69–73 |
| **991** | orthophenic acid | 86632-20-4 |  | Triterpenoids | 0.000063% | 5mg/8kg | *stems of Tripterygium regelii* | Fitoterapia 113 (2016) 69–73 |
| **992** | demethylzeylasteral | 107316-88-1 |  | Triterpenoids | 0.000375% | 30mg/8kg | *stems of Tripterygium regelii* | Fitoterapia 113 (2016) 69–73 |
| **993** | wilforol A | 167882-66-8 |  | Triterpenoids | 0.000019% | 1.5mg/8kg | *stems of Tripterygium regelii* | Fitoterapia 113 (2016) 69–73 |
| **994** | regelin D | 121880-07-7 |  | Triterpenoids | 0.000025% | 2mg/8kg | *stems of Tripterygium regelii* | Fitoterapia 113 (2016) 69–73 |
| **995** | triptotriterpenic acid B | 128301-32-6 |  | Triterpenoids | 0.000250% | 20mg/8kg | *stems of Tripterygium regelii* | Fitoterapia 113 (2016) 69–73 |
| **996** | abrusgenic acid | 84108-17-8 |  | Triterpenoids | 0.000076% | 6.1mg/8kg | *stems of Tripterygium regelii* | Fitoterapia 113 (2016) 69–73 |
| **997** | wilforlide A | 84104-71-2 |  | Triterpenoids | 0.000033% | 2.6mg/8kg | *stems of Tripterygium regelii* | Fitoterapia 113 (2016) 69–73 |
| **998** | wilforlide B | 84104-70-1 |  | Triterpenoids | 0.000116% | 9.3mg/8kg | *stems of Tripterygium regelii* | Fitoterapia 113 (2016) 69–73 |
| **999** | regelinol | 109974-22-3 |  | Triterpenoids | 0.000026% | 2.1mg/8kg | *stems of Tripterygium regelii* | Fitoterapia 113 (2016) 69–73 |
| **1000** | regelin C | 121880-06-6 |  | Triterpenoids | 0.000019% | 1.5mg/8kg | *stems of Tripterygium regelii* | Fitoterapia 113 (2016) 69–73 |
| **1001** | regelin | 109974-21-2 |  | Triterpenoids | 0.000026% | 2.1mg/8kg | *stems of Tripterygium regelii* | Fitoterapia 113 (2016) 69–73 |
| **1002** | dulcioic acid | 78516-69-5 |  | Triterpenoids | 0.000354% | 28.3mg/8kg | *stems of Tripterygium regelii* | Fitoterapia 113 (2016) 69–73 |
| **1003** | tripterygic acid A | 123914-32-9 |  | Triterpenoids | 0.000025% | 2mg/8kg | *stems of Tripterygium regelii* | Fitoterapia 113 (2016) 69–73 |
| **1004** | demethylregelin | 173991-81-6 |  | Triterpenoids | 0.000026% | 2.1mg/8kg | *stems of Tripterygium regelii* | Fitoterapia 113 (2016) 69–73 |
| **1005** | Lucidadiol | 252351-95-4 |  | Triterpenoids | 0.006667% | 20mg/300g | *dried fungi of Ganoderma lucidum* | J. Nat. Prod. 1999, 62, 1700-1701 |
| **1006** | lucidal | 252351-96-5 |  | Triterpenoids | 0.002667% | 8mg/300g | *dried fungi of Ganoderma lucidum* | J. Nat. Prod. 1999, 62, 1700-1701 |
| **1007** | 22,23-dihydroergosterol | 516-79-0 |  | Triterpenoids | 0.050000% | 150mg/300g | *dried fungi of Ganoderma lucidum* | J. Nat. Prod. 1999, 62, 1700-1701 |
| **1008** | fungisterol | 516-78-9 |  | Triterpenoids | 0.040000% | 120mg/300g | *dried fungi of Ganoderma lucidum* | J. Nat. Prod. 1999, 62, 1700-1701 |
| **1009** | ergosterol | 57-87-4 |  | Triterpenoids | 0.070000% | 210mg/300g | *dried fungi of Ganoderma lucidum* | J. Nat. Prod. 1999, 62, 1700-1701 |
| **1010** | ergosterol peroxide | 2061-64-5 |  | Triterpenoids | 0.116667% | 350mg/300g | *dried fungi of Ganoderma lucidum* | J. Nat. Prod. 1999, 62, 1700-1701 |
| **1011** | ergosta-4,6,8(14),22-tetraen-3-one | 19254-69-4 |  | Triterpenoids | 0.026667% | 80mg/300g | *dried fungi of Ganoderma lucidum* | J. Nat. Prod. 1999, 62, 1700-1701 |
| **1012** | ganodermenonol | 104700-97-2 |  | Triterpenoids | 0.003333% | 10mg/300g | *dried fungi of Ganoderma lucidum* | J. Nat. Prod. 1999, 62, 1700-1701 |
| **1013** | ganoderic acid DM | 173075-45-1 |  | Triterpenoids | 0.013333% | 40mg/300g | *dried fungi of Ganoderma lucidum* | J. Nat. Prod. 1999, 62, 1700-1701 |
| **1014** | lucidadiol | 252351-95-4 |  | Triterpenoids | 0.006667% | 20mg/300g | *dried fungi of Ganoderma lucidum* | J. Nat. Prod. 1999, 62, 1700-1701 |
| **1015** | lucidal | 252351-96-5 |  | Triterpenoids | 0.000933% | 2.8mg/300g | *dried fungi of Ganoderma lucidum* | J. Nat. Prod. 1999, 62, 1700-1701 |
| **1016** | Protopanaxatriol | 34080-08-5 |  | Triterpenoids | 2.380000% | 357mg/15g | *ginsenoside Re* | Chemistry of Natural Compounds (2018), 54, (3), 490-495 |
| **1017** | Protopanaxadiol | 30636-90-9 |  | Triterpenoids | 0.000064% | 6.4mg/10kg | *J. mandshurica* | Molecules 2015, 20, 19252-19262 |
| **1018** | Panaxadiol | 19666-76-3 |  | Triterpenoids | 0.000064% | 6.4mg/10kg | *J. mandshurica* | Molecules 2015, 20, 19252-19262 |
| **1019** | Panaxatriol | 32791-84-7 |  | Triterpenoids |  |  |  |  |
| **1020** | Pulchinenoside B5 | 14216-03-6 |  | Triterpenoids | 0.001790% | 17.90mg/1kg | *roots of Pulsatilla chinensis* | Ind. Eng. Chem. Res. 2012, 51, 14859−14866 |
| **1021** | Jujuboside A | 55466-04-1 |  | Triterpenoids | 0.000450% | 90mg/20kg | *seeds of Ziziphus jujuba Mill var. spinosa (Bunge) Hu ex. H.F.* | 药学学报Acta Pharmaceutica Sinica 2003, 38(12):934-937 |
| **1022** | Jujuboside B | 55466-05-2 |  | Triterpenoids | 0.000500% | 100mg/20kg | *seeds of Ziziphus jujuba Mill var. spinosa (Bunge) Hu ex. H.F.* | 药学学报Acta Pharmaceutica Sinica 2003, 38(12):934-937 |
| **1023** | Jujuboside E | 855184-67-7 |  | Triterpenoids | 0.000060% | 12mg/20kg | *seeds of Ziziphus jujuba Mill var. spinosa (Bunge) Hu ex. H.F.* | 药学学报Acta Pharmaceutica Sinica 2003, 38(12):934-937 |
| **1024** | Jujuboside D | 194851-84-8 |  | Triterpenoids | 0.000090% | 18mg/20kg | *seeds of Ziziphus jujuba Mill var. spinosa (Bunge) Hu ex. H.F.* | 药学学报 Acta Pharmaceutica Sinica 2004, 39(8):601-604 |
| **1025** | Glycyrrhizin | 1405-86-3 |  | Triterpenoids | 10.330000% | root of Glycyrrhiza uralensis Fisch. | *root of Glycyrrhiza uralensis Fisch.* | Zhongnan Yaoxue (2014), 12(10), 1022-1024 |
| **1026** | Glycyrrhetic acid | 471-53-4 |  | Triterpenoids | 5.920000% | root of Glycyrrhiza uralensis Fisch. | *root of Glycyrrhiza uralensis Fisch.* | Central South Pharmacy. October 2014, Vol. 12 No.10 |
| **1027** | Saikosaponin a | 20736-09-8 |  | Triterpenoids | 0.310000% | 12.4g/4kg | *root of Bufileurunz falcatum L.* | J. Chem. Soc., Perkin Trans. 1, 1975, 2043-2048 |
| **1028** | Saikosaponin b | 52441-38-0 |  | Triterpenoids | 0.160000% | 6.4g/4kg | *root of Bufileurunz falcatum L.* | J. Chem. Soc., Perkin Trans. 1, 1975, 2043-2048 |
| **1029** | Saikosaponin c | 20736-08-7 |  | Triterpenoids | 0.250000% | 10g/4kg | *root of Bufileurunz falcatum L.* | J. Chem. Soc., Perkin Trans. 1, 1975, 2043-2048 |
| **1030** | Saikosaponin d | 20874-52-6 |  | Triterpenoids | 0.165000% | 6.6g/4kg | *root of Bufileurunz falcatum L.* | J. Chem. Soc., Perkin Trans. 1, 1975, 2043-2048 |
| **1031** | Madecassoside | 34540-22-2 |  | Triterpenoids | 0.021053% | 2g/9.5kg | whole plant of *C.asiatica* | Helvetica Chimica Acta (2005), 88, (2), 297-303 |
| **1032** | asiaticoside C | 425664-06-8 |  | Triterpenoids | 0.000368% | 35mg/9.5kg | whole plant of *C.asiatica* | Helvetica Chimica Acta (2005), 88, (2), 297-303 |
| **1033** | asiaticoside D | 851222-50-9 |  | Triterpenoids | 0.000105% | 10mg/9.5kg | whole plant of *C.asiatica* | Helvetica Chimica Acta (2005), 88, (2), 297-303 |
| **1034** | asiaticoside E | 851222-51-0 |  | Triterpenoids | 0.000211% | 20mg/9.5kg | whole plant of *C.asiatica* | Helvetica Chimica Acta (2005), 88, (2), 297-303 |
| **1035** | asiaticoside F | 593254-83-2 |  | Triterpenoids | 0.000526% | 50mg/9.5kg | whole plant of *C.asiatica* | Helvetica Chimica Acta (2005), 88, (2), 297-303 |
| **1036** | lonimacranthoide IV | 1367103-79-4 |  | Triterpenoids | 0.000438% | 35mg/8kg | *flower buds of Lonicera macranthoides Hand.-Mazz. (Caprifoliaceae)* | Chinese Chemical Letters 23 (2012) 325–328 |
| **1037** | lonimacranthoide V | 1367103-80-7 |  | Triterpenoids | 0.000350% | 28mg/8kg | *flower buds of Lonicera macranthoides Hand.-Mazz. (Caprifoliaceae)* | Chinese Chemical Letters 23 (2012) 325–328 |
| **1038** | Notoginsenoside Ft1 | 155683-00-4 |  | Triterpenoids | 0.000550% | 55mg/10kg | *whole plants of Centella asiatica* | Journal of Asian Natural Products Research Vol. 13, No. 8, August 2011, 749–755 |
| **1039** | ginsenoside Rk1 | 494753-69-4 |  | Triterpenoids | 0.000560% | 56mg/10kg | *whole plants of Centella asiatica* | Journal of Asian Natural Products Research Vol. 13, No. 8, August 2011, 749–755 |
| **1040** | ginsenoside Rg5 | 186763-78-0 |  | Triterpenoids | 0.000500% | 50mg/10kg | *whole plants of Centella asiatica* | Journal of Asian Natural Products Research Vol. 13, No. 8, August 2011, 749–755 |
| **1041** | (20R)-ginsenoside Rg3 | 38243-03-7 |  | Triterpenoids | 0.000330% | 33mg/10kg | *whole plants of Centella asiatica* | Journal of Asian Natural Products Research Vol. 13, No. 8, August 2011, 749–755 |
| **1042** | ginsenoside F2 | 62025-49-4 |  | Triterpenoids | 0.000770% | 77mg/10kg | *whole plants of Centella asiatica* | Journal of Asian Natural Products Research Vol. 13, No. 8, August 2011, 749–755 |
| **1043** | (20S)-ginsenoside Rg3 | 14197-60-5 |  | Triterpenoids | 0.001070% | 107mg/10kg | *whole plants of Centella asiatica* | Journal of Asian Natural Products Research Vol. 13, No. 8, August 2011, 749–755 |
| **1044** | notoginsenoside ST-4 | 155683-02-6 |  | Triterpenoids | 0.000800% | 80mg/10kg | *whole plants of Centella asiatica* | Journal of Asian Natural Products Research Vol. 13, No. 8, August 2011, 749–755 |
| **1045** | ginsenoside Mc | 175484-06-7 |  | Triterpenoids | 0.000550% | 55mg/10kg | *whole plants of Centella asiatica* | Journal of Asian Natural Products Research Vol. 13, No. 8, August 2011, 749–755 |
| **1046** | ginsenoside Y | 83480-65-3 |  | Triterpenoids | 0.000470% | 47mg/10kg | *whole plants of Centella asiatica* | Journal of Asian Natural Products Research Vol. 13, No. 8, August 2011, 749–755 |
| **1047** | notoginsenoside Fe | 88105-29-7 |  | Triterpenoids | 0.001040% | 104mg/10kg | *whole plants of Centella asiatica* | Journal of Asian Natural Products Research Vol. 13, No. 8, August 2011, 749–755 |
| **1048** | ginsenoside Rd2 | 83480-64-2 |  | Triterpenoids | 0.000240% | 24mg/10kg | *whole plants of Centella asiatica* | Journal of Asian Natural Products Research Vol. 13, No. 8, August 2011, 749–755 |
| **1049** | Tenuifolin | 20183-47-5 |  | Triterpenoids | 0.031111% | 0.7g/2.25kg | *roots of Polygala tenuifolia* | Acta Physiol 2009, 196, 419–425 |
| **1050** | Nomilin | 1063-77-0 |  | Triterpenoids | 0.000128% | 23mg/18kg | *root bark of D. angustifolius* | RSC Advances (2015), 5, (31), 24750-24757. |
| **1051** | Dictangustone A | 1961237-40-0 |  | Triterpenoids | 0.000089% | 16mg/18kg | *root bark of D. angustifolius* | RSC Advances (2015), 5, (31), 24750-24757. |
| **1052** | Dictangustone B | 1961237-41-1 |  | Triterpenoids | 0.000061% | 11mg/18kg | *root bark of D. angustifolius* | RSC Advances (2015), 5, (31), 24750-24757. |
| **1053** | Dictangustone C | 1961237-42-2 |  | Triterpenoids | 0.000083% | 15mg/18kg | *root bark of D. angustifolius* | RSC Advances (2015), 5, (31), 24750-24757. |
| **1054** | Dictangustone D | 1961237-44-4 |  | Triterpenoids | 0.000156% | 28mg/18kg | *root bark of D. angustifolius* | RSC Advances (2015), 5, (31), 24750-24757. |
| **1055** | Dictangustone E | 1961237-46-6 |  | Triterpenoids | 0.000100% | 18mg/18kg | *root bark of D. angustifolius* | RSC Advances (2015), 5, (31), 24750-24757. |
| **1056** | Dictangustone F | 1961237-48-8 |  | Triterpenoids | 0.000067% | 12mg/18kg | *root bark of D. angustifolius* | RSC Advances (2015), 5, (31), 24750-24757. |
| **1057** | Liminol | 989-61-7 |  | Triterpenoids | 0.000067% | 12mg/18kg | *root bark of D. angustifolius* | RSC Advances (2015), 5, (31), 24750-24757. |
| **1058** | Demethylzeylasteral | 107316-88-1 |  | Triterpenoids | 0.721400% | 7.214 mg/g | *root of Tripterygium wilfordii Radix* | BMC Complementary and Alternative Medicine (2016) 16:476 |
| **1059** | Cycloastragenol | 78574-94-4 |  | Triterpenoids | 0.001625% | 130mg/8kg | *Roots of korean Astragalus membranaceus Bunge* | Chem. Pharm. Bull. 31(2)689-697(1983) |
| **1060** | Ganoderic acid A | 81907-62-2 |  | Triterpenoids | 0.263000% | 263mg/100g | *epidermis of Ganoderma lucidum (FR.) KARST* | HELVETICA CHIMICA ACTA - Vol. 65. Fasc. 2 (1982) - Nr. 62 |
| **1061** | Ganoderic acid B | 81907-61-1 |  | Triterpenoids | 0.135000% | 135mg100g | *epidermis of Ganoderma lucidum (FR.) KARST* | HELVETICA CHIMICA ACTA - Vol. 65. Fasc. 2 (1982) - Nr. 62 |
| **1062** | Celastrol | 34157-83-0 |  | Triterpenoids | 0.000311% | 2.8mg/900g | *air-dried roots of S. impressifolia* | Chemistry of Natural Compounds (2018), 54(1), 200-201 |
| **1063** | Cimigenol-3-one | 31222-32-9 |  | Triterpenoids | 0.000400% | 80mg/20kg | *rhizomes of Cimicifuga foetida* | Chem. Pharm. Bull. 60(5) 571–577 (2012) |
| **1064** | 24-epi-cimigenol-3-one | 1384384-62-6 |  | Triterpenoids | 0.000050% | 10mg/20kg | *rhizomes of Cimicifuga foetida* | Chem. Pharm. Bull. 60(5) 571–577 (2012) |
| **1065** | foetinoside | 1400771-35-8 |  | Triterpenoids | 0.000150% | 30mg/20kg | *rhizomes of Cimicifuga foetida* | Chem. Pharm. Bull. 60(5) 571–577 (2012) |
| **1066** | 25-O-acetylcimigenol | 24399-54-0 |  | Triterpenoids | 0.000100% | 20mg/20kg | *rhizomes of Cimicifuga foetida* | Chem. Pharm. Bull. 60(5) 571–577 (2012) |
| **1067** | cimigenol | 3779-59-7 |  | Triterpenoids | 0.001500% | 300mg/20kg | *rhizomes of Cimicifuga foetida* | Chem. Pharm. Bull. 60(5) 571–577 (2012) |
| **1068** | cimiacerin B | 210643-85-9 |  | Triterpenoids | 0.000050% | 10mg/20kg | *rhizomes of Cimicifuga foetida* | Chem. Pharm. Bull. 60(5) 571–577 (2012) |
| **1069** | cimifugoside H-1 | 163046-73-9 |  | Triterpenoids | 0.000150% | 30mg/20kg | *rhizomes of Cimicifuga foetida* | Chem. Pharm. Bull. 60(5) 571–577 (2012) |
| **1070** | cimigenol-3-O-β-D-xyloside | 27994-11-2 |  | Triterpenoids | 0.000500% | 100mg/20kg | *rhizomes of Cimicifuga foetida* | Chem. Pharm. Bull. 60(5) 571–577 (2012) |
| **1071** | cimiside B | 152685-91-1 |  | Triterpenoids | 0.000300% | 60mg/20kg | *rhizomes of Cimicifuga foetida* | Chem. Pharm. Bull. 60(5) 571–577 (2012) |
| **1072** | Cucurbitacin B | 6199-67-3 |  | Triterpenoids | 0.000014% | 2.1mg/15kg | *seeds of Herpetospermum pedunculosum* | Nat Prod Res. 2018 Nov 17:1-7. doi.org/10.1080/14786419.2018.1528592 |
| **1073** | cucurbitacin E | 18444-66-1 |  | Triterpenoids | 0.000025% | 3.8mg/15kg | *seeds of Herpetospermum pedunculosum* | Nat Prod Res. 2018 Nov 17:1-7. doi.org/10.1080/14786419.2018.1528592 |
| **1074** | cucurbitacin D | 3877-86-9 |  | Triterpenoids | 0.000030% | 4.5mg/15kg | *seeds of Herpetospermum pedunculosum* | Nat Prod Res. 2018 Nov 17:1-7. doi.org/10.1080/14786419.2018.1528592 |
| **1075** | cucurbitacin I | 2222-07-3 |  | Triterpenoids | 0.000024% | 3.6mg/15kg | *seeds of Herpetospermum pedunculosum* | Nat Prod Res. 2018 Nov 17:1-7. doi.org/10.1080/14786419.2018.1528592 |
| **1076** | 23,24-dihydroisocucurbitacin B | 68354-21-2 |  | Triterpenoids | 0.000017% | 2.6mg/15kg | *seeds of Herpetospermum pedunculosum* | Nat Prod Res. 2018 Nov 17:1-7. doi.org/10.1080/14786419.2018.1528592 |
| **1077** | abruslactone A | 84104-71-2 |  | Triterpenoids | 0.096296% | 52mg/54g | *powdered extract TII of Tripterygium wilfordii* | Phytochemistry 53 (2000) 805-810 |
| **1078** | dulcioic acid | 78516-69-5 |  | Triterpenoids | 0.032692% | 17mg/54g | *powdered extract TII of Tripterygium wilfordii* | Phytochemistry 53 (2000) 805-810 |
| **1079** | regelindiol A | 121880-08-8 |  | Triterpenoids | 0.068519% | 37mg/54g | *powdered extract TII of Tripterygium wilfordii* | Phytochemistry 53 (2000) 805-810 |
| **1080** | hypodiol | 198129-86-1 |  | Triterpenoids | 0.002778% | 1.5mg/54g | *powdered extract TII of Tripterygium wilfordii* | Phytochemistry 53 (2000) 805-810 |
| **1081** | 15α-hydroxy-21-keto-pristimerine | 229181-06-0 |  | Triterpenoids | 0.000067% | 2mg/3kg | *e root-bark of Maytenus catingarum* | J. Nat. Prod. 1999, 62, 750-751 |
| **1082** | pristimerin | 1258-84-0 |  | Triterpenoids | 0.016667% | 500mg/3kg | *e root-bark of Maytenus catingarum* | J. Nat. Prod. 1999, 62, 750-751 |
| **1083** | tingenone | 50802-21-6 |  | Triterpenoids | 0.006667% | 200mg/3kg | *e root-bark of Maytenus catingarum* | J. Nat. Prod. 1999, 62, 750-751 |
| **1084** | 20α-hydroxy-tingenone | 52475-25-9 |  | Triterpenoids | 0.000667% | 20mg/3kg | *e root-bark of Maytenus catingarum* | J. Nat. Prod. 1999, 62, 750-751 |
| **1085** | Glycyrrhizin | 1405-86-3 |  | Triterpenoids | 10.330000% | root of Glycyrrhiza uralensis Fisch. | *root of Glycyrrhiza uralensis Fisch.* | Zhongnan Yaoxue (2014), 12(10), 1022-1024 |
| **1086** | Glycyrrhetic acid | 471-53-4 |  | Triterpenoids | 5.920000% | root of Glycyrrhiza uralensis Fisch. | *root of Glycyrrhiza uralensis Fisch.* | Central South Pharmacy. October 2014, Vol. 12 No.10 |
| **1087** | Salaspermic acid | 71247-78-4 |  | Triterpenoids | 0.000380% | 3.0mg/790g | *root bark of M. jelski* | Phytochemistry 84 (2012) 116–124 |
| **1088** | Saikosaponin d | 20874-52-6 |  | Triterpenoids | 0.000800% | 40mg/5kg | *aerial parts of B. chinense* | Natural Product Communications (2008), 3(2), 155-161 |
| **1089** | tenuifolin | 20183-47-5 |  | Triterpenoids | 0.000200% | 40mg/20kg | *aerial parts of P. sibirica* | Fitoterapia (2013), 84, 295-301. |
| **1090** | Senegenin | 2469-34-3 |  | Triterpenoids | 0.000750% | 150mg/20kg | *aerial parts of P. sibirica* | Fitoterapia (2013), 84, 295-301. |
| **1091** | arilloside A | 273396-03-5 |  | Triterpenoids | 0.000075% | 15mg/20kg | *aerial parts of P. sibirica* | Fitoterapia (2013), 84, 295-301. |
| **1092** | Mogroside V | 88901-36-4 |  | Triterpenoids | 0.022000% | 2.2g/10kg | *Siraitia grosvenorii* | Zhongguo Zhongyao Zazhi (2011), 36(6), 721-724. |
| **1093** | Shionone | 10376-48-4 |  | Triterpenoids | 0.000422% | 63.3mg/15kg | aerial parts of *I .nervosa* | Nat Prod Res Dev 2011, 23:258-261 |
| **1094** | β-sitosterol | 83-46-5 |  | Triterpenoids | 0.000337% | 50.5mg/15kg | aerial parts of *I .nervosa* | Nat Prod Res Dev 2011, 23:258-261 |
| **1095** | α-spinasterol | 481-18-5 |  | Triterpenoids | 0.000800% | 120mg/15kg | aerial parts of *I .nervosa* | Nat Prod Res Dev 2011, 23:258-261 |
| **1096** | daucosterol | 474-58-8 |  | Triterpenoids | 0.000943% | 141.5mg/15kg | aerial parts of *I .nervosa* | Nat Prod Res Dev 2011, 23:258-261 |
| **1097** | bigelovin | 3668-14-2 |  | Triterpenoids | 0.000055% | 8.2mg/15kg | aerial parts of *I .nervosa* | Nat Prod Res Dev 2011, 23:258-261 |
| **1098** | Hederacolchiside A1 | 106577-39-3 |  | Triterpenoids | 0.004000% | 100mg/2.5kg | *leaves of S. hexaphylla* | Bioorganic & Medicinal Chemistry Letters (2019), 29(8), 965-969 |
| **1099** | Astragaloside II | 84676-89-1 |  | Triterpenoids | 0.000125% | 12.5mg/10kg | *Dried leaves of A. membranaceus* | Chemistry & Biodiversity (2015), 12, (10), 1575-1584 |
| **1100** | Hederasaponin C | 14216-03-6 |  | Triterpenoids | 0.000240% | 6mg/2.5kg | *leaves of S. hexaphylla* | Bioorganic & Medicinal Chemistry Letters (2019), 29(8), 965-970 |
| **1101** | Lupeol acetate | 1617-68-1 |  | Triterpenoids | 0.005477% | 27mg/493g | *Dried roots of E. heterophylla* | Nat Prod Res. 2018 Apr 9:1-4 |
| **1102** | 3,4-seco-Olean-12-en-4-ol-3,28-dioic acid | 182249-69-0 |  | Triterpenoids | 0.000190% | 7.8mg/4.1kg | *stems and leaves of A. viridis* | Chemistry of Natural Compounds, Vol. 53, No. 6, November, 2017 |
| **1103** | cycloeucalenol | 469-39-6 |  | Triterpenoids | 0.000195% | 8mg/4.1kg | *stems and leaves of A. viridis* | Chemistry of Natural Compounds, Vol. 53, No. 6, November, 2017 |
| **1104** | Pedunculoside | 42719-32-4 |  | Triterpenoids | 0.000035% | 7mg/20kg | *dried leaves of I. chinensi* | hytochemistry (2018), 148, 113-121 |
| **1105** | Ilexchinenin A | 2254126-12-8 |  | Triterpenoids | 0.000020% | 4mg/20kg | *dried leaves of I. chinensi* | hytochemistry (2018), 148, 113-121 |
| **1106** | rotundic acid | 20137-37-5 |  | Triterpenoids | 0.000745% | 149mg/20kg | *dried leaves of I. chinensi* | hytochemistry (2018), 148, 113-121 |
| **1107** | ilexosapogenin A | 148031-70-3 |  | Triterpenoids | 0.000020% | 4mg/20kg | *dried leaves of I. chinensi* | hytochemistry (2018), 148, 113-121 |
| **1108** | ilexoside B | 108544-40-7 |  | Triterpenoids | 0.000035% | 7mg/20kg | *dried leaves of I. chinensi* | hytochemistry (2018), 148, 113-121 |
| **1109** | 24-Methylenecycloartanone | 1449-**08**-7 |  | Triterpenoids | 0.000750% | 0.3mg/40g | *Dried leaves  of A. precatorius* | J. Agric. Food Chem. 2014, 62, 84118414 |
| **1110** | friedelan-3-one | 559-74-0 |  | Triterpenoids | 0.084419% | 726mg/860g 0.084% | *leaves and branches of Maytenus robusta* | Nat Prod Res. 2018 Nov 2:1-6. |
| **1111** | friedelane-3,16-dione | 72154-52-0 |  | Triterpenoids | 0.076045% | 676.8mg/890g 0.076% | *branches of Maytenus robusta* | Nat Prod Res. 2018 Nov 2:1-6. |
| **1112** | Adiantulupanone | 51511-05-8 |  | Triterpenoids | 0.006000% | 180mg/3kg | *dried aerial parts of Adiantum venustum* | Phytochemistry 54 (2000) 215±220 |
| **1113** | Adiantuoleanone | 287475-12-1 |  | Triterpenoids | 0.001500% | 45mg/3kg | *dried aerial parts of Adiantum venustum* | Phytochemistry 54 (2000) 215±220 |
| **1114** | Adiantulanosterol | 287475-13-2 |  | Triterpenoids | 0.004167% | 125mg/3kg | *dried aerial parts of Adiantum venustum* | Phytochemistry 54 (2000) 215±220 |
| **1115** | Rosamultin | 88515-58-6 |  | Triterpenoids | 0.005000% | 200mg/4kg | *Leaves of Adinandra nitida* | J. Agric. Food Chem. 2019, 67, 67856791 |
| **1116** | Kajiichigoside F1 | 95298-47-8 |  | Triterpenoids | 0.025000% | 1g/4kg | *Leaves of Adinandra nitida* | J. Agric. Food Chem. 2019, 67, 67856791 |
| **1117** | Daucosterol | 474-58-8 |  | Triterpenoids | 0.000375% | 30mg/8kg | *fruit of Rubus chingii Hu* | Chinese Journal of Medicinal ChemistryVol. 21 No. 3 p． 220 Jun. 2011 Sum 101 |
| **1118** | 2α,19α-Dihydroxy-3-oxo-urs-12-en-28-oic acid | 176983-21-4 |  | Triterpenoids | 0.000138% | 11mg/8kg | *fruit of Rubus chingii Hu* | Chinese Journal of Medicinal ChemistryVol. 21 No. 3 p． 220 Jun. 2011 Sum 101 |
| **1119** | β-Hederin | 35790-95-5 |  | Triterpenoids | 0.000891% | 31.2mg/3.5kg | *Dried fruits of S. mukorossi* | Natural Product Research (2016), 30, (13), 1459-1463 |
| **1120** | Ciwujianoside E | 114912-36-6 |  | Triterpenoids | 0.000098% | 8.0mg/8.2kg | *leaves of S. brachyanthera* | Bioorganic & Medicinal Chemistry Letters (2016), 26, (12), 2874-2879 |
| **1121** | 23-O-neochebuloylarjungenin 28-O-β-D-glycopyranosyl ester | 2077101-37-0 |  | Triterpenoids | 0.000278% | 5mg/1.8kg | *dried fruits of T. chebula* | Bioorganic & Medicinal Chemistry Letters (2017), 27, (1), 34-39 |
| **1122** | 23-O-40 -epi-neochebuloylarjungenin | 2077101-41-6 |  | Triterpenoids | 0.000333% | 6mg/1.8kg | *dried fruits of T. chebula* | Bioorganic & Medicinal Chemistry Letters (2017), 27, (1), 34-39 |
| **1123** | arjungenin | 58880-25-4 |  | Triterpenoids | 0.011111% | 200mg/1.8kg | *dried fruits of T. chebula* | Bioorganic & Medicinal Chemistry Letters (2017), 27, (1), 34-39 |
| **1124** | 23-O-galloylarjunic acid | 1604804-45-6 |  | Triterpenoids | 0.000889% | 16mg/1.8kg | *dried fruits of T. chebula* | Bioorganic & Medicinal Chemistry Letters (2017), 27, (1), 34-39 |
| **1125** | arjunglucoside I | 62319-70-4 |  | Triterpenoids | 0.022222% | 400mg/1.8kg | *dried fruits of T. chebula* | Bioorganic & Medicinal Chemistry Letters (2017), 27, (1), 34-39 |
| **1126** | quercotriterpenoside I | 1638340-06-3 |  | Triterpenoids | 0.001500% | 27mg/1.8kg | *dried fruits of T. chebula* | Bioorganic & Medicinal Chemistry Letters (2017), 27, (1), 34-39 |
| **1127** | terminolic acid | 564-13-6 |  | Triterpenoids | 0.004222% | 76mg/1.8kg | *dried fruits of T. chebula* | Bioorganic & Medicinal Chemistry Letters (2017), 27, (1), 34-39 |
| **1128** | 23-O-galloylterminolic acid 28-O-β-D-glucopyranosyl ester | 2236129-59-0 |  | Triterpenoids | 0.000222% | 4mg/1.8kg | *dried fruits of T. chebula* | Bioorganic & Medicinal Chemistry Letters (2017), 27, (1), 34-39 |
| **1129** | arjunolic acid | 465-00-9 |  | Triterpenoids | 0.001444% | 26mg/1.8kg | *dried fruits of T. chebula* | Bioorganic & Medicinal Chemistry Letters (2017), 27, (1), 34-39 |
| **1130** | arjunglucoside II | 62369-72-6 |  | Triterpenoids | 0.001111% | 20mg/1.8kg | *dried fruits of T. chebula* | Bioorganic & Medicinal Chemistry Letters (2017), 27, (1), 34-39 |
| **1131** | 23-O-galloylarjunolic acid | 2077101-47-2 |  | Triterpenoids | 0.000778% | 14mg/1.8kg | *dried fruits of T. chebula* | Bioorganic & Medicinal Chemistry Letters (2017), 27, (1), 34-39 |
| **1132** | 23-O-galloylarjunolic acid 28-O-β-D-glucopyranosyl ester | 2173402-75-8 |  | Triterpenoids | 0.000444% | 8mg/1.8kg | *dried fruits of T. chebula* | Bioorganic & Medicinal Chemistry Letters (2017), 27, (1), 34-39 |
| **1133** | arjunic acid | 31298-06-3 |  | Triterpenoids | 0.001278% | 23mg/1.8kg | *dried fruits of T. chebula* | Bioorganic & Medicinal Chemistry Letters (2017), 27, (1), 34-39 |
| **1134** | arjunetin | 31297-79-7 |  | Triterpenoids | 0.000500% | 9mg/1.8kg | *dried fruits of T. chebula* | Bioorganic & Medicinal Chemistry Letters (2017), 27, (1), 34-39 |
| **1135** | crataegioside | 442681-19-8 |  | Triterpenoids | 0.000278% | 5mg/1.8kg | *dried fruits of T. chebula* | Bioorganic & Medicinal Chemistry Letters (2017), 27, (1), 34-39 |
| **1136** | pinfaenoic acid 28-O-β-D-glucopyranosyl ester | 2236129-59-0 |  | Triterpenoids | 0.000167% | 3mg/1.8kg | *dried fruits of T. chebula* | Bioorganic & Medicinal Chemistry Letters (2017), 27, (1), 34-39 |
| **1137** | Arjunglucoside II | 62369-72-6 |  | Triterpenoids | 0.001111% | 20mg/1.8kg | *dried fruits of T. chebula* | Bioorganic & Medicinal Chemistry Letters (2017), 27, (1), 34-39 |
| **1138** | friedelin | 559-74-0 |  | Triterpenoids | 0.000067% | 8.0mg/12kg | *cane of Pileostegia viburnoides* | Zhongcaoyao (2014), 45(8), 1052-1055 |
| **1139** | stigmast-4-en-3-one | 1058-61-3 |  | Triterpenoids | 0.000057% | 6.8mg/12kg | *cane of Pileostegia viburnoides* | Zhongcaoyao (2014), 45(8), 1052-1055 |
| **1140** | stigmasterol | 83-48-7 |  | Triterpenoids | 0.000171% | 20.5mg/12kg | *cane of Pileostegia viburnoides* | Zhongcaoyao (2014), 45(8), 1052-1055 |
| **1141** | pomolic acid | 13849-91-7 |  | Triterpenoids | 0.000028% | 3.3mg/12kg | *cane of Pileostegia viburnoides* | Zhongcaoyao (2014), 45(8), 1052-1055 |
| **1142** | oleanolic acid | 508-02-1 |  | Triterpenoids | 0.000018% | 2.2mg/12kg | *cane of Pileostegia viburnoides* | Zhongcaoyao (2014), 45(8), 1052-1055 |
| **1143** | daucosterol | 474-58-8 |  | Triterpenoids | 0.000061% | 7.3mg/12kg | *cane of Pileostegia viburnoides* | Zhongcaoyao (2014), 45(8), 1052-1055 |
| **1144** | canophyllal | 14440-40-5 |  | Triterpenoids | 0.000040% | 40mg/100kg | *roots of T. wilfordii* | Natural product research.2019. 24:1-7 |
| **1145** | friedelin | 559-74-0 |  | Triterpenoids | 0.000067% | 8mg/12kg | *cane of Pileostegia viburnoides* | Zhongcaoyao (2014), 45(8), 1052-1055. |
| **1146** | stigmast-4-en-3-one | 1058-61-3 |  | Triterpenoids | 0.000057% | 6.8mg/12kg | *cane of Pileostegia viburnoides* | Zhongcaoyao (2014), 45(8), 1052-1055. |
| **1147** | stigmasterol | 83-48-7 |  | Triterpenoids | 0.000171% | 20.5mg/12kg | *cane of Pileostegia viburnoides* | Zhongcaoyao (2014), 45(8), 1052-1055. |
| **1148** | ursolic acid | 77-52-1 |  | Triterpenoids | 0.000039% | 4.7mg/12kg | *cane of Pileostegia viburnoides* | Zhongcaoyao (2014), 45(8), 1052-1055. |
| **1149** | pomolic acid | 13849-91-7 |  | Triterpenoids | 0.000028% | 3.3mg/12kg | *cane of Pileostegia viburnoides* | Zhongcaoyao (2014), 45(8), 1052-1055. |
| **1150** | oleanolic acid | 508-02-1 |  | Triterpenoids | 0.000018% | 2.2mg/12kg | *cane of Pileostegia viburnoides* | Zhongcaoyao (2014), 45(8), 1052-1055. |
| **1151** | ovalifolone A | 1578239-06-1 |  | Triterpenoids | 0.000267% | 12mg/4.5kg | *stem bark of Garcinia ovalifolia* | Phytochemistry Letters (2013), 6, (2), 157-161 |
| **1152** | ovalifolone B | 1578239-07-2 |  | Triterpenoids | 0.000222% | 10mg/4.5kg | *stem bark of Garcinia ovalifolia* | Phytochemistry Letters (2013), 6, (2), 157-161 |
| **1153** | β-​Friedelinol | 16844-71-6 |  | Triterpenoids | 0.000344% | 15.5mg/4.5kg | *stem bark of Garcinia ovalifolia* | Phytochemistry Letters (2013), 6, (2), 157-161 |
| **1154** | canophyllol | 14440-41-6 |  | Triterpenoids | 0.000222% | 10mg/4.5kg | *stem bark of Garcinia ovalifolia* | Phytochemistry Letters (2013), 6, (2), 157-161 |
| **1155** | canophyllal | 14440-40-5 |  | Triterpenoids | 0.000189% | 8.5mg/4.5kg | *stem bark of Garcinia ovalifolia* | Phytochemistry Letters (2013), 6, (2), 157-161 |
| **1156** | garcinane | 1054553-55-7 |  | Triterpenoids | 0.000133% | 6mg/4.5kg | *stem bark of Garcinia ovalifolia* | Phytochemistry Letters (2013), 6, (2), 157-161 |
| **1157** | fern-8-ene | 1750-35-2 |  | Triterpenoids | 0.020000% | 272mg/1.36kg | *leaves of adianyum pedatum* | Chemical & Pharmaceutical Bulletin (1993), 41, (2), 268-71 |
| **1158** | fern-9(11)-ene | 1615-99-2 |  | Triterpenoids | 0.327206% | 4450mg/1.36kg | *leaves of adianyum pedatum* | Chemical & Pharmaceutical Bulletin (1993), 41, (2), 268-71 |
| **1159** | fern-7,9(11)-diene | 2407-21-8 |  | Triterpenoids | 0.001544% | 21mg/1.36kg | *leaves of adianyum pedatum* | Chemical & Pharmaceutical Bulletin (1993), 41, (2), 268-71 |
| **1160** | fern-7-ene | 1615-98-1 |  | Triterpenoids | 0.019853% | 270mg/1.36kg | *leaves of adianyum pedatum* | Chemical & Pharmaceutical Bulletin (1993), 41, (2), 268-71 |
| **1161** | neohop-13(18)-ene | 72633-85-3 |  | Triterpenoids | 0.001029% | 14mg/1.36kg | *leaves of adianyum pedatum* | Chemical & Pharmaceutical Bulletin (1993), 41, (2), 268-71 |
| **1162** | neohop-12-ene | 2734-37-4 |  | Triterpenoids | 0.005147% | 70mg/1.36kg | *leaves of adianyum pedatum* | Chemical & Pharmaceutical Bulletin (1993), 41, (2), 268-71 |
| **1163** | filic-3-ene | 906320-82-9 |  | Triterpenoids | 0.017206% | 234mg/1.36kg | *leaves of adianyum pedatum* | Chemical & Pharmaceutical Bulletin (1993), 41, (2), 268-71 |
| **1164** | neohopa-11,13(18)-diene | 3608--5-7 |  | Triterpenoids | 0.016912% | 230mg/1.36kg | *leaves of adianyum pedatum* | Chemical & Pharmaceutical Bulletin (1993), 41, (2), 268-71 |
| **1165** | filicenal | 13843-88-4 |  | Triterpenoids | 0.040074% | 545mg/1.36kg | *leaves of adianyum pedatum* | Chemical & Pharmaceutical Bulletin (1993), 41, (2), 268-71 |
| **1166** | adiantone | 1253-69-6 |  | Triterpenoids | 0.001471% | 20mg/1.36kg | *leaves of adianyum pedatum* | Chemical & Pharmaceutical Bulletin (1993), 41, (2), 268-71 |
| **1167** | isoadiantone | 54352-47-5 |  | Triterpenoids | 0.001471% | 20mg/1.36kg | *leaves of adianyum pedatum* | Chemical & Pharmaceutical Bulletin (1993), 41, (2), 268-71 |
| **1168** | isoglaucanone | 1172-78-7 |  | Triterpenoids | 0.001838% | 25mg/1.36kg | *leaves of adianyum pedatum* | Chemical & Pharmaceutical Bulletin (1993), 41, (2), 268-71 |
| **1169** | 23-hydroxyfernene | 70588-14-6 |  | Triterpenoids | 0.038235% | 520mg/1.36kg | *leaves of adianyum pedatum* | Chemical & Pharmaceutical Bulletin (1993), 41, (2), 268-71 |
| **1170** | Deoxyflindissone | 107176-31-8 |  | Triterpenoids | 0.000600% | 15mg/2.5kg | *. Stems and stem bark of C. walteri* | J. Nat. Prod.201174154-59 |
| **1171** | Morolic acid | 559-68-2 |  | Triterpenoids | 0.000055% | 138mg/250kg | *bark of Anthocephalus chinensis* | Nat Prod Res Dev 2011，2 3: 393-398 |
| **1172** | clethric acid | 8800-57-7 |  | Triterpenoids | 0.000243% | 608.6mg/250kg | *bark of Anthocephalus chinensis* | Nat Prod Res Dev 2011，2 3: 393-398 |
| **1173** | Uncarinic acid E | 277751-61-8 |  | Triterpenoids | 0.000015% | 0.6mg/4.1kg | *Barks of Betula platyphylla var. japonica* | Chem Biodivers. 2017 Apr;14(4) |
| **1174** | Germanicol acetate | 10483-91-7 |  | Triterpenoids | 0.004462% | 22mg/493g | *Dried roots of E. heterophylla* | Dried roots of E. heterophylla |
| **1175** | Betulinaldehyde | 13159-28-9 |  | Triterpenoids | 0.000231% | 46.1mg/20kg | *Lveaves of Paliurus ramosissimus160* | Chin. J. Org. Chem. 2017, 37, 520～525 |
| **1176** | Fern-7-en-19-one | 222294-61-3 |  | Triterpenoids | 0.327206% | 4450mg/1.36kg | *leaves of adianyum pedatum* | Chemical & Pharmaceutical Bulletin (1993), 41, (2), 268-71 |
| **1177** | Myrianthic acid 3,23-acetonide | 578710-52-8 |  | Triterpenoids | 0.000893% | 26.8mg/3kg | *roots of R. aleaefolius* | Molbank 2003, 2003(3), M327 |
| **1178** | 7-ketositosterol | 2034-74-4 |  | Triterpenoids | 0.000110% | 11mg/10kg | *fruit of Evodia delavayi* | Zhongguo Yaoxue Zazhi (Beijing, China) (2016), 51(9), 694-697. |
| **1179** | Kaji-ichigoside F1 | 95298-47-8 |  | Triterpenoids | 0.025000% | 1g/4kg | *Leaves of Adinandra nitida* | J. Agric. Food Chem. 2019, 67, 67856791 |
| **1180** | Palustrisoic acid A | 2231778-09-7 |  | Triterpenoids | 0.006923% | 108mg/1.56kg | *fruiting bodies of F. palustris* | Phytochemistry (Elsevier) (2018), 152, 10-21. |
| **1181** | Palustrisoic acid B | 2231778-10-0 |  | Triterpenoids | 0.006282% | 98mg/1.56kg | *fruiting bodies of F. palustris* | Phytochemistry (Elsevier) (2018), 152, 10-21. |
| **1182** | Palustrisoic acid C | 2231778-11-1 |  | Triterpenoids | 0.001218% | 19mg/1.56kg | *fruiting bodies of F. palustris* | Phytochemistry (Elsevier) (2018), 152, 10-21. |
| **1183** | Palustrisoic acid D | 2231778-12-2 |  | Triterpenoids | 0.001090% | 17mg/1.56kg | *fruiting bodies of F. palustris* | Phytochemistry (Elsevier) (2018), 152, 10-21. |
| **1184** | Palustrisoic acid E | 2231778-13-3 |  | Triterpenoids | 0.000962% | 15mg/1.56kg | *fruiting bodies of F. palustris* | Phytochemistry (Elsevier) (2018), 152, 10-21. |
| **1185** | Palustrisoic acid F | 2231778-14-4 |  | Triterpenoids | 0.002244% | 35mg/1.56kg | *fruiting bodies of F. palustris* | Phytochemistry (Elsevier) (2018), 152, 10-21. |
| **1186** | Palustrisoic acid G | 2231805-29-9 |  | Triterpenoids | 0.000577% | 9mg/1.56kg | *fruiting bodies of F. palustris* | Phytochemistry (Elsevier) (2018), 152, 10-21. |
| **1187** | Palustrisoic acid H | 2231778-15-5 |  | Triterpenoids | 0.000833% | 13mg/1.56kg | *fruiting bodies of F. palustris* | Phytochemistry (Elsevier) (2018), 152, 10-21. |
| **1188** | Palustrisolide A | 2231778-16-6 |  | Triterpenoids | 0.008205% | 128mg/1.56kg | *fruiting bodies of F. palustris* | Phytochemistry (Elsevier) (2018), 152, 10-21. |
| **1189** | Palustrisolide B | 2231778-17-7 |  | Triterpenoids | 0.005769% | 90mg/1.56kg | *fruiting bodies of F. palustris* | Phytochemistry (Elsevier) (2018), 152, 10-21. |
| **1190** | Palustrisolide C | 2231778-18-8 |  | Triterpenoids | 0.000167% | 2.6mg/1.56kg | *fruiting bodies of F. palustris* | Phytochemistry (Elsevier) (2018), 152, 10-21. |
| **1191** | Palustrisolide D | 2231778-19-9 |  | Triterpenoids | 0.000641% | 10mg/1.56kg | *fruiting bodies of F. palustris* | Phytochemistry (Elsevier) (2018), 152, 10-21. |
| **1192** | Palustrisolide E | 2231778-20-2 |  | Triterpenoids | 0.000833% | 13mg/1.56kg | *fruiting bodies of F. palustris* | Phytochemistry (Elsevier) (2018), 152, 10-21. |
| **1193** | Palustrisolide F | 2231778-21-3 |  | Triterpenoids | 0.001154% | 18mg/1.56kg | *fruiting bodies of F. palustris* | Phytochemistry (Elsevier) (2018), 152, 10-21. |
| **1194** | Palustrisolide G | 2231778-22-4 |  | Triterpenoids | 0.000192% | 3mg/1.56kg | *fruiting bodies of F. palustris* | Phytochemistry (Elsevier) (2018), 152, 10-21. |
| **1195** | Alisol C 23-acetate | 26575-93-9 |  | Triterpenoids | 0.000165% | 24.82mg/15kg | *dried rhizomes of A. orientale* | Arch Pharm Res Vol 35, No 11, 1919-1926, 2012 |
| **1196** | Ursolic aldehyde | 19132-81-1 |  | Triterpenoids | 0.000014% | 7mg/50kg | *fruits of C. acuminata Decne* | Nat Prod Res. 2018 Jun 20:1-6 |
| **1197** | Wilforic acid F | 273198-92-8 |  | Triterpenoids | 0.011111% | 6mg/54g | *powdered extract TII of Tripterygium wilfordii* | Phytochemistry (2000), 53(7), 805-810 |
| **1198** | Wilforlide B | 84104-70-1 |  | Triterpenoids | 0.010780% | 2156mg/20kg | *root xylem of T. wilfordii* | Asian Journal of Chemistry (2014), 26, (14), 4344-4346 |
| **1199** | wilforlide A | 84104-71-2 |  | Triterpenoids | 0.005260% | 1052mg/20kg | *root xylem of T. wilfordii* | Asian Journal of Chemistry (2014), 26, (14), 4344-4346 |
| **1200** | cangoronine | 138884-84-1 |  | Triterpenoids | 0.000155% | 31mg/20kg | *root xylem of T. wilfordii* | Asian Journal of Chemistry (2014), 26, (14), 4344-4346 |
| **1201** | salaspermic acid | 71247-78-4 |  | Triterpenoids | 0.000145% | 29mg/20kg | *root xylem of T. wilfordii* | Asian Journal of Chemistry (2014), 26, (14), 4344-4346 |
| **1202** | orthosphenic acid | 86632-20-4 |  | Triterpenoids | 0.000075% | 15mg/20kg | *root xylem of T. wilfordii* | Asian Journal of Chemistry (2014), 26, (14), 4344-4346 |
| **1203** | epifriedelinol | 16844-71-6 |  | Triterpenoids | 0.008500% | 68mg/800g | *root bark of C. vulcanicola w* | Phytochemistry (Elsevier) (2012), 84, 116-124 |
| **1204** | maytenfoliol | 84316-84-7 |  | Triterpenoids | 0.003038% | 24.3mg/800g | *root bark of C. vulcanicola w* | Phytochemistry (Elsevier) (2012), 84, 116-124 |
| **1205** | polpunonic acid | 33600-93-0 |  | Triterpenoids | 0.044000% | 352mg/800g | *root bark of C. vulcanicola w* | Phytochemistry (Elsevier) (2012), 84, 116-124 |
| **1206** | Populninic acid | 120090-56-4 |  | Triterpenoids | 0.000813% | 6.5mg/800g | *root bark of C. vulcanicola w* | Phytochemistry (Elsevier) (2012), 84, 116-124 |
| **1207** | epifriedelinol | 16844-71-6 |  | Triterpenoids | 0.002886% | 22.8mg/790g | *root bark of M. jelskii* | Phytochemistry (Elsevier) (2012), 84, 116-124 |
| **1208** | friedelin | 559-74-0 |  | Triterpenoids | 0.007013% | 55.4mg/790g | *root bark of M. jelskii* | Phytochemistry (Elsevier) (2012), 84, 116-124 |
| **1209** | cangoronine | 138884-84-1 |  | Triterpenoids | 0.000835% | 6.6mg/790g | *root bark of M. jelskii* | Phytochemistry (Elsevier) (2012), 84, 116-124 |
| **1210** | cerin | 468-67-7 |  | Triterpenoids | 0.000557% | 4.4mg/790g | *root bark of M. jelskii* | Phytochemistry (Elsevier) (2012), 84, 116-124 |
| **1211** | orthosphenic acid | 86632-20-4 |  | Triterpenoids | 0.000241% | 1.9mg/790g | *root bark of M. jelskii* | Phytochemistry (Elsevier) (2012), 84, 116-124 |
| **1212** | canophyllol | 14440-41-6 |  | Triterpenoids | 0.002785% | 22mg/790g | *root bark of M. jelskii* | Phytochemistry (Elsevier) (2012), 84, 116-124 |
| **1213** | maytenfoliol | 84316-84-7 |  | Triterpenoids | 0.000532% | 4.2mg/790g | *root bark of M. jelskii* | Phytochemistry (Elsevier) (2012), 84, 116-124 |
| **1214** | Polpunonic acid | 33600-93-0 |  | Triterpenoids | 0.010380% | 82mg/790g | *root bark of M. jelskii* | Phytochemistry (Elsevier) (2012), 84, 116-124 |
| **1215** | Demethyl zeylasteral | 107316-88-1 |  | Triterpenoids | 0.000050% | 15g/30kg | *dried root bark of T. wilfordii* | Phytochemistry 39 (1995) 1159. |
| **1216** | Wilforol A | 167882-66-8 |  | Triterpenoids | 0.001567% | 470mg/30kg | *dried root bark of T. wilfordii* | Phytochemistry 39 (1995) 1159. |
| **1217** | Wilforol B | 167875-33-4 |  | Triterpenoids | 0.073333% | 22g/30kg | *dried root bark of T. wilfordii* | Phytochemistry 39 (1995) 1159. |
| **1218** | celastrol | 34157-83-0 |  | Triterpenoids | 0.051667% | 15.5g/30kg | *dried root bark of T. wilfordii* | Phytochemistry 39 (1995) 1159. |
| **1219** | Alisol R | 2221029-52-1 |  | Triterpenoids | 0.000130% | 13mg/10kg | *Dried rhizomes of A. orientale* | Chemistry & Biodiversity (2017), 14, (12) |
| **1220** | 25-Methoxyalisol F | 2221029-53-2 |  | Triterpenoids | 0.001140% | 114mg/10kg | *Dried rhizomes of A. orientale* | Chemistry & Biodiversity (2017), 14, (12) |
| **1221** | 16β-Hydroperoxyalisol B 23-Acetate | 2221029-54-3 |  | Triterpenoids | 0.002000% | 200mg/10kg | *Dried rhizomes of A. orientale* | Chemistry & Biodiversity (2017), 14, (12) |
| **1222** | 16β-Hydroperoxyalisol B | 2221029-56-5 |  | Triterpenoids | 0.000070% | 7mg/10kg | *Dried rhizomes of A. orientale* | Chemistry & Biodiversity (2017), 14, (12) |
| **1223** | 16β-Methoxyalisol B | 2221029-57-6 |  | Triterpenoids | 0.000150% | 15mg/10kg | *Dried rhizomes of A. orientale* | Chemistry & Biodiversity (2017), 14, (12) |
| **1224** | 16β-Methoxyalisol E | 2221029-58-7 |  | Triterpenoids | 0.000050% | 5mg/10kg | *Dried rhizomes of A. orientale* | Chemistry & Biodiversity (2017), 14, (12) |
| **1225** | 16β,25-Dimethoxyalisol E | 2221029-59-8 |  | Triterpenoids | 0.000120% | 12mg/10kg | *Dried rhizomes of A. orientale* | Chemistry & Biodiversity (2017), 14, (12) |
| **1226** | Alisol T | 2221029-61-2 |  | Triterpenoids | 0.000080% | 8mg/10kg% | *Dried rhizomes of A. orientale* | Chemistry & Biodiversity (2017), 14, (12) |
| **1227** | Alisol U | 176045-53-7 |  | Triterpenoids | 0.000500% | 50mg/10kg | *Dried rhizomes of A. orientale* | Chemistry & Biodiversity (2017), 14, (12) |
| **1228** | 16α-Hydroxybauerenol | 214351-30-1 |  | Triterpenoids | 0.007000% | 168mg/2.4kg | *dried rhizome of P. tricholobus* | Phytochemistry (2005), 66, (19), 2340-2345 |
| **1229** | Petatrichol A | 865244-52-6 |  | Triterpenoids | 0.000167% | 4mg/2.4kg | *dried rhizome of P. tricholobus* | Phytochemistry (2005), 66, (19), 2340-2346 |
| **1230** | Petatrichol B | 869502-27-2 |  | Triterpenoids | 0.000042% | 1mg/2.4kg | *dried rhizome of P. tricholobus* | Phytochemistry (2005), 66, (19), 2340-2347 |
| **1231** | Taraxasterone | 6786-16-9 |  | Triterpenoids | 0.000100% | 5mg/5kg | *roots of Taraxacum officinale* | Tetrahedron (2013), 69(5), 1583-1589 |
| **1232** | taraxast-1,20(30)- dien-3-one | 953077-15-1 |  | Triterpenoids | 0.000140% | 7mg/5kg | *roots of Taraxacum officinale* | Tetrahedron (2013), 69(5), 1583-1589 |
| **1233** | Kihadanin A | 125276-62-2 |  | Triterpenoids | 0.004444% | 10mg/225g 0.004% | *T. elegans ssp. elegans seeds* | Phytochemistry (1997), 45(1), 141-148 |
| **1234** | Kihadanin B | 73793-68-7 |  | Triterpenoids | 0.002667% | 6mg/225g 0.003% | *T. elegans ssp. elegans seeds* | Phytochemistry (1997), 45(1), 141-148 |
| **1235** | Elegantin A | 190441-84-0 |  | Triterpenoids | 0.004000% | 9mg/225g 0.004% | *T. elegans ssp. elegans seeds* | Phytochemistry (1997), 45(1), 141-148 |
| **1236** | Euphol | 514-47-6 |  | Triterpenoids | 0.003922% | 13.1mg/334g | *stem bark of S. grantii w* | Planta Medica (2014), 80(6), 458-464 |
| **1237** | Friedelin | 559-74-0 |  | Triterpenoids | 0.001198% | 4mg/334g | *stem bark of S. grantii w* | Planta Medica (2014), 80(6), 458-464 |
| **1238** | 3β-Friedelinol | 16844-71-6 |  | Triterpenoids | 0.000359% | 1.2mg/334g | *stem bark of S. grantii w* | Planta Medica (2014), 80(6), 458-464 |
| **1239** | Saikosaponin b3 | 58316-42-0 |  | Triterpenoids | 0.000580% | 29mg/5kg | *aerial parts of B. chinense* | Natural Product Communications (2008), 3(2), 155-157 |
| **1240** | Saikosaponin b1 | 58558-08-0 |  | Triterpenoids | 0.000400% | 20mg/5kg | *aerial parts of B. chinense* | Natural Product Communications (2008), 3(2), 155-157 |
| **1241** | Saikosaponin a | 20736-09-8 |  | Triterpenoids | 0.000400% | 20mg/5kg | *aerial parts of B. chinense* | Natural Product Communications (2008), 3(2), 155-158 |
| **1242** | Saikosaponin b2 | 58316-41-9 |  | Triterpenoids | 0.000440% | 22mg/5kg | *aerial parts of B. chinense* | Natural Product Communications (2008), 3(2), 155-159 |
| **1243** | Saikosaponin b3 | 58316-42-0 |  | Triterpenoids | 0.000580% | 29mg/5kg | *aerial parts of B. chinense* | Natural Product Communications (2008), 3(2), 155-160 |
| **1244** | Saikosaponin d | 20874-52-6 |  | Triterpenoids | 0.000800% | 40mg/5kg | *aerial parts of B. chinense* | Natural Product Communications (2008), 3(2), 155-161 |
| **1245** | Saikosaponin g | 99365-19-2 |  | Triterpenoids | 0.003600% | 180mg/5kg | *aerial parts of B. chinense* | Natural Product Communications (2008), 3(2), 155-162 |
| **1246** | Saikosaponin k | 224433-69-6 |  | Triterpenoids | 0.000100% | 5mg/5kg | *aerial parts of B. chinense* | Natural Product Communications (2008), 3(2), 155-163 |
| **1247** | Saikosaponin i | 103629-71-6 |  | Triterpenoids | 0.004000% | 200mg/5kg | *aerial parts of B. chinense* | Natural Product Communications (2008), 3(2), 155-164 |
| **1248** | 23-Hydroxytormentic acid | 70868-78-9 |  | Triterpenoids | 0.000072% | 35mg/48.5kg | *dried leaves of C. paliurus* | Phytochemistry (Elsevier) (2018), 151, 119-127 |
| **1249** | Lycojaponicuminol C | 1651839-34-7 |  | Triterpenoids | 0.000061% | 2.5mg/4.1kg | *moss of Lycopodium japonicum* | Fitoterapia. 2014 Jul;96:95-102. |
| **1250** | 16-Oxolycoclavanol | 53800-21-8 |  | Triterpenoids | 0.000100% | 15mg/15kg | *Whole herbs of P. cernua* | Fitoterapia. 2017 Jun;119:45-50 |
| **1251** | Lycernuic ketone D | 2301994-35-2 |  | Triterpenoids | 0.000100% | 15mg/15kg | *Whole herbs of P. cernua* | Fitoterapia. 2017 Jun;119:45-50 |
| **1252** | Lycernuic ketone E | 1217268-18-2 |  | Triterpenoids | 0.000067% | 10mg/15kg | *Whole herbs of P. cernua* | Fitoterapia. 2017 Jun;119:45-50 |
| **1253** | Lycernuic A | 2301994-36-3 |  | Triterpenoids | 0.000033% | 5mg/15kg | *Whole herbs of P. cernua* | Fitoterapia. 2017 Jun;119:45-50 |
| **1254** | 16-Oxolyclanitin-29-yl p-coumarate | 140701-70-8 |  | Triterpenoids | 0.000188% | 15mg/8kg | *Diphasiastrum complanatum* | Phytochemistry. 2008 Jan;69(2):506-10 |
| **1255** | serratane-3α,14α,15α,20β,21β,24,29-heptol | 1004984-92-2 |  | Triterpenoids | 0.000250% | 20mg/8kg | *Diphasiastrum complanatum* | Phytochemistry. 2008 Jan;69(2):506-10 |
| **1256** | 3α,20β,21β-trihydroxyserrat-14-en-24-oic acid | 1004984-93-3 |  | Triterpenoids | 0.001250% | 100mg/8kg | *Diphasiastrum complanatum* | Phytochemistry. 2008 Jan;69(2):506-10 |
| **1257** | 3β,20β,21β-trihydroxyserrat-14-en-24-oic acid | 1004984-94-4 |  | Triterpenoids | 0.000125% | 10mg/8kg | *Diphasiastrum complanatum* | Phytochemistry. 2008 Jan;69(2):506-10 |
| **1258** | 3α,20β,21β-trihydroxy-16- oxoserrat-14-en-24-oic acid | 1004984-95-5 |  | Triterpenoids | 0.000625% | 50mg/8kg | *Diphasiastrum complanatum* | Phytochemistry. 2008 Jan;69(2):506-10 |
| **1259** | serratenediol | 2239-24-9 |  | Triterpenoids | 0.000625% | 50mg/8kg | *Diphasiastrum complanatum* | Phytochemistry. 2008 Jan;69(2):506-10 |
| **1260** | 21-epi-serratenediol | 1449-06-5 |  | Triterpenoids | 0.000250% | 20mg/8kg | *Diphasiastrum complanatum* | Phytochemistry. 2008 Jan;69(2):506-10 |
| **1261** | lycoclaninol | 13956-51-9 |  | Triterpenoids | 0.000375% | 30mg/8kg | *Diphasiastrum complanatum* | Phytochemistry. 2008 Jan;69(2):506-10 |
| **1262** | wightianol B | 77311-65-0 |  | Triterpenoids | 0.001250% | 100mg/8kg | *Diphasiastrum complanatum* | Phytochemistry. 2008 Jan;69(2):506-10 |
| **1263** | lycernuic acid A | 53755-77-4 |  | Triterpenoids | 0.000188% | 15mg/8kg | *Diphasiastrum complanatum* | Phytochemistry. 2008 Jan;69(2):506-10 |
| **1264** | 16-oxolyclanitin-29-yl p-coumarate | 140701-70-8 |  | Triterpenoids | 0.000375% | 30mg/8kg | *Diphasiastrum complanatum* | Phytochemistry. 2008 Jan;69(2):506-10 |
| **1265** | lycoclavanin | 27832-90-2 |  | Triterpenoids | 0.002500% | 200mg/8kg | *Diphasiastrum complanatum* | Phytochemistry. 2008 Jan;69(2):506-10 |
| **1266** | α-onocerin | 511-01-3 |  | Triterpenoids | 0.025000% | 2g/8kg | *Diphasiastrum complanatum* | Phytochemistry. 2008 Jan;69(2):506-10 |
| **1267** | Mogrol | 88930-15-8 |  | Triterpenoids | 0.001917% | 23mg/1.2kg | *The fruits of T. tricuspidata* | Phytochemistry (2002), 59, (2), 215-228 |
| **1268** | mogroside IV | 89590-95-4 |  | Triterpenoids | 0.179710% | 372mg/207g | *Dried and powdered fruits of Siraitia siamensis* | Agricultural and Biological Chemistry (1989), 53, (12) |
| **1269** | mogroside V | 88901-36-4 |  | Triterpenoids | 0.067633% | 140mg/207g | *Dried and powdered fruits of Siraitia siamensis* | Agricultural and Biological Chemistry (1989), 53, (12) |
| **1270** | glycoside E | 115784-07-1 |  | Triterpenoids | 0.007246% | 15mg/207g | *Dried and powdered fruits of Siraitia siamensis* | Agricultural and Biological Chemistry (1989), 53, (12) |
| **1271** | glycoside F | 94992-08-2 |  | Triterpenoids | 0.028986% | 60mg/207g | *Dried and powdered fruits of Siraitia siamensis* | Agricultural and Biological Chemistry (1989), 53, (12) |
| **1272** | glycoside A | 19855-40-4 |  | Triterpenoids | 0.020290% | 42mg/207g | *Dried and powdered fruits of Siraitia siamensis* | Agricultural and Biological Chemistry (1989), 53, (12) |
| **1273** | siamenoside I | 126105-12-2 |  | Triterpenoids | 0.026087% | 54mg/207g | *Dried and powdered fruits of Siraitia siamensis* | Agricultural and Biological Chemistry (1989), 53, (12) |
| **1274** | Khekadaengoside A | 415710-99-5 |  | Triterpenoids | 0.004167% | 50mg/1.2kg | *fruits of T. tricuspidata* | Phytochemistry. 2002 Jan;59(2):215-28 |
| **1275** | Khekadaengoside B | 415711-00-1 |  | Triterpenoids | 0.004250% | 51mg/1.2kg | *fruits of T. tricuspidata* | Phytochemistry. 2002 Jan;59(2):215-28 |
| **1276** | Khekadaengoside C | 415711-01-2 |  | Triterpenoids | 0.008333% | 100mg/1.2kg | *fruits of T. tricuspidata* | Phytochemistry. 2002 Jan;59(2):215-28 |
| **1277** | Khekadaengoside D | 415711-02-3 |  | Triterpenoids | 0.002000% | 24mg/1.2kg | *fruits of T. tricuspidata* | Phytochemistry. 2002 Jan;59(2):215-28 |
| **1278** | Khekadaengoside E | 415711-03-4 |  | Triterpenoids | 0.009250% | 111mg/1.2kg | *fruits of T. tricuspidata* | Phytochemistry. 2002 Jan;59(2):215-28 |
| **1279** | Cucurbitacin J 2-O-β-glucopyranoside | 415711-04-5 |  | Triterpenoids | 0.005167% | 62mg/1.2kg | *fruits of T. tricuspidata* | Phytochemistry. 2002 Jan;59(2):215-28 |
| **1280** | Cucurbitacin K | 622784-88-7 |  | Triterpenoids | 0.011417% | 137mg/1.2kg | *fruits of T. tricuspidata* | Phytochemistry. 2002 Jan;59(2):215-28 |
| **1281** | Khekadaengoside F | 415711-06-7 |  | Triterpenoids | 0.001083% | 13mg/1.2kg | *fruits of T. tricuspidata* | Phytochemistry. 2002 Jan;59(2):215-28 |
| **1282** | Khekadaengoside G | 415711-07-8 |  | Triterpenoids | 0.008417% | 101mg/1.2kg | *fruits of T. tricuspidata* | Phytochemistry. 2002 Jan;59(2):215-28 |
| **1283** | Khekadaengoside I | 415711-10-3 |  | Triterpenoids | 0.000667% | 8mg/1.2kg | *fruits of T. tricuspidata* | Phytochemistry. 2002 Jan;59(2):215-28 |
| **1284** | Khekadaengoside J | 415711-11-4 |  | Triterpenoids | 0.001250% | 15mg/1.2kg | *fruits of T. tricuspidata* | Phytochemistry. 2002 Jan;59(2):215-28 |
| **1285** | Khekadaengoside K | 122738-86-7 |  | Triterpenoids | 0.003000% | 36mg/1.2kg | *fruits of T. tricuspidata* | Phytochemistry. 2002 Jan;59(2):215-28 |
| **1286** | Khekadaengoside L | 415711-12-5 |  | Triterpenoids | 0.000667% | 8mg/1.2kg | *fruits of T. tricuspidata* | Phytochemistry. 2002 Jan;59(2):215-28 |
| **1287** | Khekadaengoside M | 415711-13-6 |  | Triterpenoids | 0.004333% | 52mg/1.2kg | *fruits of T. tricuspidata* | Phytochemistry. 2002 Jan;59(2):215-28 |
| **1288** | Arvenin I | 65247-27-0 |  | Triterpenoids | 0.001200% | 30mg/2.5kg | *roots of T. kirilowii* | Archives of Pharmacal Research (2015), 38, (8), 1443-1448 |
| **1289** | 10α-Cucurbita-5,24-dien-3β-ol | 35012-08-9 |  | Triterpenoids | 0.000320% | 8mg/2.5kg | *roots of T. kirilowii* | Archives of Pharmacal Research (2015), 38, (8), 1443-1448 |
| **1290** | Colocynthenin A | 2244685-32-1 |  | Triterpenoids | 0.000205% | 4.1mg/2kg | *powder of C. colocynthis fruit* | J. Nat. Prod. 2018, 81, 2115−2119 |
| **1291** | Colocynthenin B | 2244685-33-2 |  | Triterpenoids | 0.000275% | 5.5mg/2kg | *powder of C. colocynthis fruit* | J. Nat. Prod. 2018, 81, 2115−2119 |
| **1292** | Colocynthenin C | 2244685-34-3 |  | Triterpenoids | 0.000590% | 11.8mg/2kg | *powder of C. colocynthis fruit* | J. Nat. Prod. 2018, 81, 2115−2119 |
| **1293** | Colocynthenin D | 2244685-35-4 |  | Triterpenoids | 0.000435% | 8.7mg/2kg | *powder of C. colocynthis fruit* | J. Nat. Prod. 2018, 81, 2115−2119 |
| **1294** | Isocucurbitacin B | 17278-28-3 |  | Triterpenoids |  | 20mg/Maxim | *underground parts of Bolbostemma paniculatum* | Journal of Asian Natural Products Research, 9, 2, 2007, 187–190 |
| **1295** | Cucurbitacin E | 18444-66-1 |  | Triterpenoids | 0.000025% | 3.8mg/15kg | *seeds of H. pedunculosum* | Nat Prod Res. 2018 Nov 17:1-7 |
| **1296** | cucurbitacin D | 3877-86-9 |  | Triterpenoids | 0.000030% | 4.5mg/15kg | *seeds of H. pedunculosum* | Nat Prod Res. 2018 Nov 17:1-7 |
| **1297** | cucurbitacin B | 6199-67-3 |  | Triterpenoids | 0.000014% | 2.1mg/15kg | *seeds of H. pedunculosum* | Nat Prod Res. 2018 Nov 17:1-7 |
| **1298** | cucurbitacin I | 2222--07-3 |  | Triterpenoids | 0.000024% | 3.6mg/15kg | *seeds of H. pedunculosum* | Nat Prod Res. 2018 Nov 17:1-7 |
| **1299** | 23,24-dihydroisocucurbitacin B | 68354-21-2 |  | Triterpenoids | 0.000017% | 2.6mg/15kg | *seeds of H. pedunculosum* | Nat Prod Res. 2018 Nov 17:1-7 |
| **1300** | Cucurbitacin A | 6040-19-3 |  | Triterpenoids | 0.100000% | 500mg/0.5kg | *fruits of Cucumis prophetarum* | Pharmaceutical Biology  2002, 40,02, 154–159 |
| **1301** | Lucidal | 252351-96-5 |  | Triterpenoids | 0.000009% | 1.6mg/18kg | *dried fruiting bodies of G. luteomarginatum* | Phytochemistry (Elsevier) (2018), 156, 89-95 |
| **1302** | (+)-(5α,23E)-27-nor-lanosta-8,23-dien-3,7,25-trione | 86377-52-8 |  | Triterpenoids | 0.000023% | 4.1mg/18kg | *dried fruiting bodies of G. luteomarginatum* | Phytochemistry (Elsevier) (2018), 156, 89-95 |
| **1303** | Tirucallone | 54325-09-6 |  | Triterpenoids | 0.000025% | 4.5mg/18kg | *dried fruiting bodies of G. luteomarginatum* | Phytochemistry (Elsevier) (2018), 156, 89-95 |
| **1304** | (+)-(5α,24E)-3β,11α-dihydroxylanosta-8,24-dien-7-oxo-26-al |  |  | Triterpenoids | 0.000021% | 3.7mg/18kg | *dried fruiting bodies of G. luteomarginatum* | Phytochemistry (Elsevier) (2018), 156, 89-95 |
| **1305** | Ganoderic Acid Y | 86377-52-8 |  | Triterpenoids | 0.000018% | 3.2mg/18kg | *dried fruiting bodies of G. luteomarginatum* | Phytochemistry (Elsevier) (2018), 156, 89-95 |
| **1306** | delta7-Avenasterol | 23290-26-8 |  | Triterpenoids | 2.5/18000000 | 2.5mg/18kg | *dried fruiting bodies of G. luteomarginatum* | Phytochemistry (Elsevier) (2018), 156, 89-95 |
| **1307** | icterogenin | 561-47-7 |  | Triterpenoids | 0.001300% | 13mg/1kg | *aerial parts of Lantana camara L* | Phytochemistry (Elsevier) (2017), 144, 106-112. |
| **1308** | oleanolic acid | 508-02-1 |  | Triterpenoids | 0.001000% | 10mg/1kg | *aerial parts of Lantana camara L* | Phytochemistry (Elsevier) (2017), 144, 106-112. |
| **1309** | 22β-oleanolic acid | 13224-63-0 |  | Triterpenoids | 0.000150% | 1.5mg/1kg | *aerial parts of Lantana camara L* | Phytochemistry (Elsevier) (2017), 144, 106-112. |
| **1310** | 22-hydroxy-oleanonic acid | 897952-33-9 |  | Triterpenoids | 0.001100% | 11mg/1kg | *aerial parts of Lantana camara L* | Phytochemistry (Elsevier) (2017), 144, 106-112. |
| **1311** | lantadene B | 467-82-3 |  | Triterpenoids | 0.002000% | 20mg/1kg | *aerial parts of Lantana camara L* | Phytochemistry (Elsevier) (2017), 144, 106-112. |
| **1312** | lantadene A | 467-81-2 |  | Triterpenoids | 0.001400% | 14mg/1kg | *aerial parts of Lantana camara L* | Phytochemistry (Elsevier) (2017), 144, 106-112. |
| **1313** | oleanonic acid | 17990-42-0 |  | Triterpenoids | 0.004500% | 45mg/1kg | *aerial parts of Lantana camara L* | Phytochemistry (Elsevier) (2017), 144, 106-112. |
| **1314** | lantadene D | 132194-33-3 |  | Triterpenoids | 0.001000% | 10mg/1kg | *aerial parts of Lantana camara L* | Phytochemistry (Elsevier) (2017), 144, 106-112. |
| **1315** | pomonic acid | 13849-90-6 |  | Triterpenoids | 0.000100% | 1mg/1kg | *aerial parts of Lantana camara L* | Phytochemistry (Elsevier) (2017), 144, 106-112. |
| **1316** | pomolic acid | 13849-91-7 |  | Triterpenoids | 0.000200% | 2mg/1kg | *aerial parts of Lantana camara L* | Phytochemistry (Elsevier) (2017), 144, 106-112. |
| **1317** | lantanilic acid | 60657-41-2 |  | Triterpenoids | 0.000160% | 1.6mg/1kg | *aerial parts of Lantana camara L* | Phytochemistry (Elsevier) (2017), 144, 106-112. |
| **1318** | camaric acid | 146450-83-1 |  | Triterpenoids | 0.000770% | 7.7mg/1kg | *aerial parts of Lantana camara L* | Phytochemistry (Elsevier) (2017), 144, 106-112. |
| **1319** | Lantanilic acid | 60657-41-2 |  | Triterpenoids | 0.000002% | 1.6mg/1.0kg | *aerial parts of Lantana camara L* | Phytochemistry 144 (2017) 106-112 |
| **1320** | 3β-Hydroxy-lantadene | 107964-01-2 |  | Triterpenoids | 0.000100% | 1.0mg/1.0kg | *aerial parts of Lantana camara L* | Phytochemistry 144 (2017) 106-113 |
| **1321** | Methyl lucidenate E2 | 98665-12-4 |  | Triterpenoids | 0.000080% | 8mg/10kg | *dried fruiting bodies of G. lucidum* | Journal of Natural Products (2010), 73(2), 172-176 |
| **1322** | Butyl ganoderate A | 1207106-19-1 |  | Triterpenoids | 0.000300% | 30mg/10kg | *dried fruiting bodies of G. lucidum* | Journal of Natural Products (2010), 73(2), 172-176 |
| **1323** | Butyl ganoderate B | 1207106-20-4 |  | Triterpenoids | 0.000100% | 10mg/10kg | *dried fruiting bodies of G. lucidum* | Journal of Natural Products (2010), 73(2), 172-176 |
| **1324** | Butyl lucidenate N | 1207106-21-5 |  | Triterpenoids | 0.000150% | 15mg/10kg | *dried fruiting bodies of G. lucidum* | Journal of Natural Products (2010), 73(2), 172-176 |
| **1325** | Butyl lucidenate A | 1207106-22-6 |  | Triterpenoids | 0.000040% | 4mg/10kg | *dried fruiting bodies of G. lucidum* | Journal of Natural Products (2010), 73(2), 172-176 |
| **1326** | ganoderic acid A | 81907-62-2 |  | Triterpenoids | 0.000040% | 4mg/10kg | *dried fruiting bodies of G. lucidum* | Journal of Natural Products (2010), 73(2), 172-176 |
| **1327** | methyl ganoderate A | 81907-63-3 |  | Triterpenoids | 0.000090% | 9mg/10kg | *dried fruiting bodies of G. lucidum* | Journal of Natural Products (2010), 73(2), 172-176 |
| **1328** | ganoderic acid B | 2231061-48-4 |  | Triterpenoids | 0.000400% | 40mg/10kg | *dried fruiting bodies of G. lucidum* | Journal of Natural Products (2010), 73(2), 172-176 |
| **1329** | methyl ganoderate B | 81907-65-5 |  | Triterpenoids | 0.000090% | 9mg/10kg | *dried fruiting bodies of G. lucidum* | Journal of Natural Products (2010), 73(2), 172-176 |
| **1330** | methyl ganoderate D | 97210-12-3 |  | Triterpenoids | 0.000050% | 5mg/10kg | *dried fruiting bodies of G. lucidum* | Journal of Natural Products (2010), 73(2), 172-176 |
| **1331** | ganoderic acid E | 98665-14-6 |  | Triterpenoids | 0.000170% | 17mg/10kg | *dried fruiting bodies of G. lucidum* | Journal of Natural Products (2010), 73(2), 172-176 |
| **1332** | methyl ganoderate E | 98718-43-5 |  | Triterpenoids | 0.000020% | 2mg/10kg | *dried fruiting bodies of G. lucidum* | Journal of Natural Products (2010), 73(2), 172-176 |
| **1333** | ganolucidic acid A | 98665-21-5 |  | Triterpenoids | 0.000220% | 22mg/10kg | *dried fruiting bodies of G. lucidum* | Journal of Natural Products (2010), 73(2), 172-176 |
| **1334** | methyl ganoderate H | 98665-11-3 |  | Triterpenoids | 0.000080% | 8mg/10kg | *dried fruiting bodies of G. lucidum* | Journal of Natural Products (2010), 73(2), 172-176 |
| **1335** | lucidenic acid N | 364622-33-3 |  | Triterpenoids | 0.000070% | 7mg/10kg | *dried fruiting bodies of G. lucidum* | Journal of Natural Products (2010), 73(2), 172-176 |
| **1336** | methyl lucidenate A | 105742-79-8 |  | Triterpenoids | 0.000100% | 10mg/10kg | *dried fruiting bodies of G. lucidum* | Journal of Natural Products (2010), 73(2), 172-176 |
| **1337** | methyl lucidenate P | 647856-35-7 |  | Triterpenoids | 0.000020% | 2mg/10kg | *dried fruiting bodies of G. lucidum* | Journal of Natural Products (2010), 73(2), 172-176 |
| **1338** | methyl lucidenate E | 98665-12-4 |  | Triterpenoids | 0.000060% | 6mg/10kg | *dried fruiting bodies of G. lucidum* | Journal of Natural Products (2010), 73(2), 172-176 |
| **1339** | methyl lucidenate F | 98665-10-2 |  | Triterpenoids | 0.000050% | 5mg/10kg | *dried fruiting bodies of G. lucidum* | Journal of Natural Products (2010), 73(2), 172-176 |
| **1340** | Hancokinol | 132294-77-0 |  | Triterpenoids | 0.001600% | 16mg/1kg | *Vincetoxicum officinale* | Fitoterapia (2000), 71(5), 584-586 |
| **1341** | 7β-Hydroxycucurbitacin B | 1135141-79-5 |  | Triterpenoids | 0.000500% | 25mg/5kg | *aerial parts of Cucumis melo* | Journal of Natural Products (2009), 72(5), 824-829 |
| **1342** | 3α,30-Diacetyloxy-12α-hydroxy-23-oxoeupha-7,24-dien-21,16β-olid-28-oic acid 28-O-β-D-glucopyranosyl ester | 215160-96-6 |  | Triterpenoids | 0.000043% | 12mg/28kg | *Dried leaves of Lantana camara* | Journal of Natural Products (1998), 61(11), 1328-1331 |
| **1343** | Methyl lucidenate L | 110267-46-4 |  | Triterpenoids | 0.000214% | 0.8mg/373g | *fruiting bodies of G. lucidum* | J. Nat. Prod. 2003, 66, 1582-1585 |
| **1344** | Methyl lucidenate P | 647856-35-7 |  | Triterpenoids | 0.002949% | 11.0mg/373g | *fruiting bodies of G. lucidum* | J. Nat. Prod. 2003, 66, 1582-1585 |
| **1345** | Methyl lucidenate Q | 648430-32-4 |  | Triterpenoids | 0.001314% | 4.9mg/373g | *fruiting bodies of G. lucidum* | J. Nat. Prod. 2003, 66, 1582-1585 |
| **1346** | Lucidenic acid D | 98665-16-8 |  | Triterpenoids | 0.065442% | 244.1mg/373g | *fruiting bodies of G. lucidum* | J. Nat. Prod. 2003, 66, 1582-1585 |
| **1347** | Lucidenic acid E | 98665-12-4 |  | Triterpenoids | 0.007426% | 27.7mg/373g | *fruiting bodies of G. lucidum* | J. Nat. Prod. 2003, 66, 1582-1585 |
| **1348** | Lucidenic acid F | 98665-18-0 |  | Triterpenoids | 0.000724% | 2.7mg/373g | *fruiting bodies of G. lucidum* | J. Nat. Prod. 2003, 66, 1582-1585 |
| **1349** | Ganoderic acid F | 98665-15-7 |  | Triterpenoids | 0.002145% | 8.0mg/373g | *fruiting bodies of G. lucidum* | J. Nat. Prod. 2003, 66, 1582-1585 |
| **1350** | Hancolupenone | 132746-04-4 |  | Triterpenoids | 0.001000% | 150mg/15kg | *root of cynanchum hancokianum* | Yao xue xue bao = Acta pharmaceutica Sinica (1991), 26(8), 584-92 |
| **1351** | Niga-ichigoside F | 95262-48-9 |  | Triterpenoids | 0.000667% | 20.0mg/3kg | *Dried root Geum urbanum* | Nat Prod Res. 2018 Nov;32(21):2529-2534 |
| **1352** | pomonic acid | 13849-90-6 |  | Triterpenoids | 0.000267% | 8.0mg/3kg | *Dried root Geum urbanum* | Nat Prod Res. 2018 Nov;32(21):2529-2534 |
| **1353** | rubuside A | 1189131-72-3 |  | Triterpenoids | 0.000167% | 5.0mg/3kg | *Dried root Geum urbanum* | Nat Prod Res. 2018 Nov;32(21):2529-2534 |
| **1354** | rubuside D | 1189131-75-6 |  | Triterpenoids | 0.000143% | 4.3mg/3kg | *Dried root Geum urbanum* | Nat Prod Res. 2018 Nov;32(21):2529-2534 |
| **1355** | Daucosterol | 474-58-8 |  | Triterpenoids | 0.000133% | 4.0mg/3kg | *Dried root Geum urbanum* | Nat Prod Res. 2018 Nov;32(21):2529-2534 |
| **1356** | arjunglucoside I | 62319-70-4 |  | Triterpenoids | 0.000367% | 11.0mg/3kg | *Dried root Geum urbanum* | Nat Prod Res. 2018 Nov;32(21):2529-2534 |
| **1357** | Methyl lucidenate N | 1276655-49-2 |  | Triterpenoids | 0.000009% | 5mg/57.8kg | *stems of S. chinensis w* | Planta Medica (2010), 76(4), 358-361 |
| **1358** | Methyl ganoderenate D | 748136-03-0 |  | Triterpenoids | 0.007143% | 150mg/2.1kg | *fruiting bodies of G. applanatum* | J. Nat. Prod. 2004, 67, 1110-111 |
| **1359** | 2α,3α,24-Trihydroxyursa -12,20(30)-dien-28-oic acid | 341503-22-8 |  | Triterpenoids | 0.000011% | 0.9mg/8kg | *aerial parts of I. coesta* | Archives of Pharmacal Research (2011), 34(12), 2007-2014 |
| **1360** | Methyl lucidenate D | 98665-09-9 |  | Triterpenoids | 0.000429% | 1.6mg/373g | *fruiting bodies of G. lucidum* | Journal of Natural Products (2003), 66(12), 1582-1585 |
| **1361** | Cucurbitacin IIa | 58546-34-2 |  | Triterpenoids | 0.925926% | 25g/2.7kg | *dried rhizomes of Hemsleya* | Fitoterapia (2014), 94, 88-93 |
| **1362** | 7-hydroxy-cucurbitacin F-25-O-acetate | 625834-00-6 |  | Triterpenoids | 0.003444% | 93mg/2.7kg | *dried rhizomes of Hemsleya* | Fitoterapia (2014), 94, 88-93 |
| **1363** | 2β,3α,16α,20(R),24(S),25-hexahydroxy-9-methyl 19- norlanost-5-en-11,22-dione | 1621019-72-4 |  | Triterpenoids | 0.003444% | 93mg/2.7kg | *dried rhizomes of Hemsleya* | Fitoterapia (2014), 94, 88-93 |
| **1364** | scandenogenin D | 1621019-73-5 |  | Triterpenoids | 0.003444% | 93mg/2.7kg | *dried rhizomes of Hemsleya* | Fitoterapia (2014), 94, 88-93 |
| **1365** | 23,24- dihydrocucurbitacin F | 50298-90-3 |  | Triterpenoids | 0.718519% | 19.4g/2.7kg | *dried rhizomes of Hemsleya* | Fitoterapia (2014), 94, 88-93 |
| **1366** | 23,24-dihydrocucurbitacin B | 13201-14-4 |  | Triterpenoids | 0.015704% | 424mg/2.7kg | *dried rhizomes of Hemsleya* | Fitoterapia (2014), 94, 88-93 |
| **1367** | 23,24-dihydro-3-epi-isocucurbitacin B | 121283-86-1 |  | Triterpenoids | 0.000519% | 14mg/2.7kg | *dried rhizomes of Hemsleya* | Fitoterapia (2014), 94, 88-93 |
| **1368** | 23,24-dihydrocucurbitacin E | 28973-67-3 |  | Triterpenoids | 0.005630% | 152mg/2.7kg | *dried rhizomes of Hemsleya* | Fitoterapia (2014), 94, 88-93 |
| **1369** | Cucurbitacin Iia | 58546-34-2 |  | Triterpenoids | 0.011889% | 321mg/2.7kg | *dried rhizomes of Hemsleya* | Fitoterapia (2014), 94, 88-93 |
| **1370** | 2β,3β,16α, 20(R),25-pentahydroxy-9-methyl-19-norlanost-5-en-7,11,22- trione | 2133460-75-8 |  | Triterpenoids | 0.000556% | 15mg/2.7kg | *dried rhizomes of Hemsleya* | Fitoterapia (2014), 94, 88-93 |
| **1371** | 23,24-dihydrocucurbitacin E-2-O-D-glucoside | 61014-18-4 |  | Triterpenoids | 0.019778% | 534mg/2.7kg | *dried rhizomes of Hemsleya* | Fitoterapia (2014), 94, 88-93 |
| **1372** | 2-O-β-D-glucopyranosyl cucurbitacin | 1910091-23-4 |  | Triterpenoids | 0.030000% | 810mg/2.7kg | *dried rhizomes of Hemsleya* | Fitoterapia (2014), 94, 88-93 |
| **1373** | Methyl lucidenate A | 105742-79-8 |  | Triterpenoids | 0.000100% | 10mg/10kg | *dried fruiting bodies of G. lucidum* | Journal of Natural Products (2010), 73(2), 172-176 |
| **1374** | 16,25-Di-O-acetylcucurbitacin F | 2062685-10-1 |  | Triterpenoids | 0.001000% | 30mg/3kg | *dried rhizomes of H. jinfushanensi* | Phytochemistry Letters (2015), 14, 239-244 |
| **1375** | 23,24-Dihydrocucurbitacin F | 50298-90-3 |  | Triterpenoids | 0.001970% | 35mg/1777g | *cortex from I. lindheimeri roots* | Biochemical Systematics and Ecology (2014), 54, 237-239 |
| **1376** | Sericic acid | 55306-03-1 |  | Triterpenoids | 0.000006% | 5.0mg/82kg | *Fresh ripe fruits of F. ananassa cv. Falandi* | Food Chemistry (2016), 203, 67-72 |
| **1377** | tormentic acid | 13850-16-3 |  | Triterpenoids | 0.000006% | 5.0mg/82kg | *Fresh ripe fruits of F. ananassa cv. Falandi* | Food Chemistry (2016), 203, 67-72 |
| **1378** | 16-Desoxycucurbitacin V | 2081098-85-1 |  | Triterpenoids |  |  | *stem root of Hemsleya pengxianensis* | CN 106380503 A |
| **1379** | Ganoderic acid C1 | 95311-97-0 |  | Triterpenoids | 0.472000% | 0.472000% | *Ganoderma lucidum* | Chemical & Pharmaceutical Bulletin (2002), 50(6), 837-840 |
| **1380** | Araloside A | 7518-22-1 |  | Triterpenoids | 0.000163% | 7.5mg/4.6kg | *roots of P. japonicus* | Journal of Natural Products (2011), 74(4), 796-802 |

Table S2. 3D structural similarity score relative to celastrol

| CAS Number | Pubchem  ID | ClosestNames  (celastrol) | Closest  Similarities | Bio-content |
| --- | --- | --- | --- | --- |
| *100198-0*9-2 | 12004524 | 122724 | 0.184783 | 0.001450% |
| 102519-34-6 | 11504083 | 122724 | 0.241379 | 0.030200% |
| 1033288-92-4 | 24879071 | 122724 | 0.126126 | 0.000746% |
| 10388-48-4 | 11419367 | 122724 | 0.172414 | 0.000750% |
| 10483-91-7 | 345509 | 122724 | 0.186047 | 0.004462% |
| 115334-05-9 | 73351981 | 122724 | 0.164835 | 0.000300% |
| 115404-57-4 | 14021529 | 122724 | 0.186813 | 0.000300% |
| 1159579-44-8 | 91895416 | 122724 | 0.241379 | 0.001929% |
| 1159579-45-9 | 91895417 | 122724 | 0.304878 | 0.000153% |
| 1169805-98-4 | 42643119 | 122724 | 0.163043 | 0.008000% |
| 1169806-00-1 | 42643169 | 122724 | 0.208791 | 0.001200% |
| 1169806-02-3 | 42643118 | 122724 | 0.227273 | 0.002000% |
| 117020-59-4 | 6451093 | 122724 | 0.200000 | 0.000059% |
| 1260-05-5 | 91895435 | 122724 | 0.168539 | 0.002020% |
| 128397-09-1 | 14605533 | 122724 | 0.229885 | 0.009920% |
| 129724-83-0 | 71307335 | 122724 | 0.182796 | 0.000155% |
| 134476-74-7 | 91895427 | 122724 | 0.170455 | 0.000080% |
| 13850-16-3 | 73193 | 122724 | 0.235294 | 0.001550% |
| 152243-70-4 | 57397367 | 122724 | 0.222222 | 0.000147% |
| 1615-94-7 | 12442794 | 122724 | 0.156627 | 0.000305% |
| 1616-93-9 | 92156 | 122724 | 0.235294 | 0.171429% |
| 19865-87-3 | 44421647 | 122724 | 0.146341 | 0.038500% |
| 20248-08-2 | 14079468 | 122724 | 0.177215 | 0.001439% |
| 21671-00-1 | 12315515 | 122724 | 0.164835 | 0.000493% |
| ‘2202-01-9 | 76313961 | 122724 | 0.195402 | 0.021500% |
| 2239-24-9 | 164947 | 122724 | 0.162500 | 0.010900% |
| 22549-21-9 | 25567101 | 122724 | 0.149425 | 0.001486% |
| 246868-97-3 | 15887718 | 122724 | 0.163043 | 0.251667% |
| 25279-15-6 | 14038588 | 122724 | 0.142857 | 0.000360% |
| 263844-79-7 | 71307351 | 122724 | 0.168421 | 0.005208% |
| 263844-80-0 | 71307350 | 122724 | 0.163265 | 0.005208% |
| 26790-93-2 | 320908 | 122724 | 0.138298 | 0.000140% |
| 2239-24-9 | 102004501 | 122724 | 0.166667 | 0.003023% |
| 31298-06-3 | 15385516 | 122724 | 0.238095 | 0.001278% |
| 32206-97-6 | 15559638 | 122724 | 0.166667 | 0.000054% |
| 32208-45-0 | 636516 | 122724 | 0.202247 | 0.004660% |
| 32214-80-5 | 161739 | 122724 | 0.182796 | 0.006152% |
| 329975-47-5 | 129316510 | 122724 | 0.180851 | 0.001608% |
| 35761-54-7 | 21625900 | 122724 | 0.149425 | 0.010000% |
| 35833-62-6 | 12019028 | 122724 | 0.153846 | 0.001680% |
| 35833-69-3 | 44421648 | 122724 | 0.156627 | 0.004750% |
| 35959-05-8 | 21606663 | 122724 | 0.138298 | 0.258000% |
| 35959-08-1 | 21606662 | 122724 | 0.153061 | 0.000500% |
| 4547-24-4 | 6918774 | 122724 | 0.232558 | 0.102520% |
| 472-15-1 | 64971 | 122724 | 0.197674 | ​0.1166% |
| 472-30-0 | 102004474 | 122724 | 0.189474 | 0.000603% |
| 473-98-3 | 72326 | 122724 | 0.146067 | ​0.0090% |
| 477529-70-7 | 11142533 | 122724 | 0.168539 | 0.000560% |
| 508-04-3 | 71307354 | 122724 | 0.160494 | 0.000857% |
| 5085-72-3 | 101341 | 122724 | 0.200000 | 0.002000% |
| 51361-60-5 | 13915602 | 122724 | 0.188235 | 0.000250% |
| 53155-25-2 | 471426 | 122724 | 0.235294 | 0.114170% |
| 545-24-4 | 9932254 | 122724 | 0.217949 | 0.003125% |
| 545-47-1 | 259846 | 122724 | 0.170732 | 0.400000% |
| 55511-14-3 | 101277401 | 122724 | 0.202247 | 0.000041% |
| 559-70-6 | 73145 | 122724 | 0.209877 | 0.000333% |
| 559-74-0 | 91472 | 122724 | 0.226667 | 0.012044% |
| 56421-13-7 | 12315516 | 122724 | 0.164835 | 0.008333% |
| 57576-31-5 | 91895423 | 122724 | 0.129032 | 0.000063% |
| 58316-41-9 | 21637642 | 122724 | 0.141593 | 0.000440% |
| 62393-88-8 | 86573576 | 122724 | 0.173469 | 0.000368% |
| 63303-42-4 | 11700083 | 122724 | 0.230000 | 0.000197% |
| 6426-43-3 | 13889352 | 122724 | 0.179775 | 0.002231% |
| 659738-08-6 | 11035347 | 122724 | 0.226190 | 0.001368% |
| 67253-01-4 | 21625899 | 122724 | 0.114943 | 0.019250% |
| 7372-30-7 | 6475119 | 122724 | 0.222222 | 0.000500% |
| 77-52-1 | 64945 | 122724 | 0.220930 | 3.000000% |
| 83-46-5 | 222284 | 122724 | 0.118280 | 0.010000% |
| 85287-58-7 | 10247031 | 122724 | 0.172414 | 0.011480% |
| 91095-51-1 | 73554076 | 122724 | 0.230769 | 0.000463% |
| 99026-99-0 | 5321283 | 122724 | 0.166667 | 0.000197% |
| 879327-55-6 | 11848194 | 122724 | 0.166667 | 0.000068% |
| 659738-09-7 | 11759710 | 122724 | 0.231707 | 0.001074% |
| 1007387-95-2 | 91895449 | 122724 | 0.169811 | 0.000240% |
| 102519-34-6 | 11504083 | 122724 | 0.241379 | 0.030200% |
| 103974-74-9 | 9898760 | 122724 | 0.235955 | 0.000100% |
| 104700-97-2 | 13934284 | 122724 | 0.191489 | 0.190000% |
| 104700-98-3 | 13934282 | 122724 | 0.180851 | 0.070000% |
| 1058-61-3 | 5484202 | 122724 | 0.130435 | 0.000280% |
| 1059-14-9 | 115250 | 122724 | 0.152941 | 0.001250% |
| 106518-61-0 | 14395297 | 122724 | 0.200000 | 0.000120% |
| 106518-62-1 | 471007 | 122724 | 0.170213 | 0.000300% |
| 106518-63-2 | 73177 | 122724 | 0.222222 | 0.000320% |
| 107316-88-1 | 10322911 | 122724 | 0.521127 | 0.000375% |
| 108864-53-5 | 45273405 | 122724 | 0.186813 | 0.000188% |
| 109974-21-2 | 163808 | 122724 | 0.204301 | 0.000026% |
| 109974-22-3 | 163809 | 122724 | 0.210526 | 0.000026% |
| 114567-47-4 | 471008 | 122724 | 0.195652 | 0.000720% |
| 115040-04-5 | 102004564 | 122724 | 0.141304 | 0.000333% |
| 115458-73-6 | 14021705 | 122724 | 0.165049 | 0.000700% |
| 1180-71-8 | 179651 | 122724 | 0.135417 | 0.000500% |
| 118172-80-8 | 14109777 | 122724 | 0.569231 | 0.000385% |
| 119318-15-9 | 71307357 | 122724 | 0.214286 | 0.000050% |
| 1193250-54-2 | 125114671 | 122724 | 0.123457 | 0.000250% |
| 120211-98-5 | 14312996 | 122724 | 0.226190 | 0.067570% |
| 121064-78-6 | 14335955 | 122724 | 0.232323 | 0.093200% |
| 121072-40-0 | 14335956 | 122724 | 0.232323 | 0.018640% |
| 109974-21-2 | 163808 | 122724 | 0.204301 | 0.000026% |
| 121880-07-7 | 129520 | 122724 | 0.235955 | 0.000025% |
| 123914-32-9 | 130072 | 122724 | 0.206897 | 0.000025% |
| 1254-85-9 | 21596358 | 122724 | 0.184783 | 0.044048% |
| 1258-84-0 | 159516 | 122724 | 0.830508 | 0.016667% |
| 61448-03-1 | 15127233 | 122724 | 0.182927 | 0.002000% |
| 128301-32-6 | 195563 | 122724 | 0.268293 | 0.000250% |
| 130288-60-7 | 12019474 | 122724 | 0.131868 | 0.000071% |
| 130295-73-7 | 21582935 | 122724 | 0.164835 | 0.000071% |
| 131985-08-5 | 636694 | 122724 | 0.142857 | 0.000033% |
| 13849-91-7 | 382831 | 122724 | 0.223529 | 0.001435% |
| 13850-16-3 | 73193 | 122724 | 0.235294 | 0.001550% |
| 14197-60-5 | 9918693 | 122724 | 0.119266 | 0.001070% |
| 14216-03-6 | 11491905 | 122724 | 0.178862 | 0.001790% |
| 142778-14-1 | 56658413 | 122724 | 0.183673 | 0.000071% |
| 142778-15-2 | 10391774 | 122724 | 0.200000 | 0.000119% |
| 468732-42-5 | 56600068 | 122724 | 0.141667 | 0.000121% |
| 142877-49-4 | 6450144 | 122724 | 0.207547 | 0.000020% |
| 14356-51-5 | 14489125 | 122724 | 0.244186 | 0.015000% |
| 146450-83-1 | 91895465 | 122724 | 0.217822 | 0.011870% |
| 150821-16-2 | 69570248 | 122724 | 0.241379 | 0.000250% |
| 152253-67-3 | 158802 | 122724 | 0.240964 | 0.000400% |
| 152685-91-1 | 91895424 | 122724 | 0.128440 | 0.000300% |
| 155683-00-4 | 91973814 | 122724 | 0.116071 | 0.000550% |
| 157414-07-8 | 44566782 | 122724 | 0.177778 | 0.000238% |
| 1617-70-5 | 92158 | 122724 | 0.197531 | 0.000324% |
| 162059-94-1 | 15767725 | 122724 | 0.194444 | 0.000080% |
| 163046-73-9 | 15241163 | 122724 | 0.181818 | 0.000150% |
| 167882-66-8 | 10096097 | 122724 | 0.558824 | 0.001567% |
| 16844-71-6 | 119242 | 122724 | 0.200000 | 0.008500% |
| 17020-04-1 | 177801 | 122724 | 0.202247 | 0.000833% |
| 173075-45-1 | 11784642 | 122724 | 0.189474 | 0.013333% |
| 173991-81-6 | 73554051 | 122724 | 0.213483 | 0.000026% |
| 17990-42-0 | 12313704 | 122724 | 0.250000 | 0.004500% |
| 18444-66-1 | 5281319 | 122724 | 0.170000 | 0.000025% |
| 18449-41-7 | 73412 | 122724 | 0.230769 | 0.000106% |
| 18642-44-9 | 10032468 | 122724 | 0.125000 | 0.001500% |
| 186763-78-0 | 11550001 | 122724 | 0.133333 | 0.000500% |
| 188970-21-0 | 10551785 | 122724 | 0.206897 | 0.000074% |
| 190906-61-7 | 44575704 | 122724 | 0.206897 | 0.000025% |
| 19254-69-4 | 6441416 | 122724 | 0.151163 | 0.026667% |
| 194851-84-8 | 92043344 | 122724 | 0.102941 | 0.000090% |
| 198129-86-1 | 101936045 | 122724 | 0.142857 | 0.000075% |
| 19833-13-7 | 15922574 | 122724 | 0.206186 | 0.000159% |
| 20065-99-0 | 122130012 | 122724 | 0.164706 | 0.000133% |
| 20137-37-5 | 12315075 | 122724 | 0.227273 | 0.000745% |
| 201534-08-9 | 101937091 | 122724 | 0.328947 | 0.000055% |
| 201534-09-0 | 44575705 | 122724 | 0.268293 | 0.057407% |
| 201534-10-3 | 44559634 | 122724 | 0.207317 | 0.000019% |
| 20183-47-5 | 21588226 | 122724 | 0.192308 | 0.031111% |
| 127-22-0 | 344467 | 122724 | 0.179487 | 0.000343% |
| 277751-59-4 | 44583694 | 122724 | 0.190909 | 0.001000% |
| 20736-08-7 | 381832788 | 122724 | 0.119658 | 0.250000% |
| ’2222-07-3 | 5281321 | 122724 | 0.168421 | 0.000024% |
| 22318-10-1 | 185481 | 122724 | 0.189474 | 0.000063% |
| ‘2259-06-5 | 73493 | 122724 | 0.177778 | 0.002330% |
| 22626-26-2 | 185503 | 122724 | 0.208791 | 0.000675% |
| 238748-74-8 | 129716127 | 122724 | 0.190909 | 0.000010% |
| 24480-45-3 | 472768 | 122724 | 0.383562 | 0.033333% |
| 252351-96-5 | 10366713 | 122724 | 0.164948 | 0.002667% |
| 25499-90-5 | 11869658 | 122724 | 0.238095 | 0.000400% |
| 256925-92-5 | 15541911 | 122724 | 0.113208 | 0.003300% |
| 268214-50-2 | 5468948 | 122724 | 0.160377 | 1.879497% |
| 268214-51-3 | 6479499 | 122724 | 0.174757 | 0.015036% |
| 268214-52-4 | 10648063 | 122724 | 0.160000 | 0.027143% |
| 268541-24-8 | 101062653 | 122724 | 0.200000 | 0.000038% |
| 268541-25-9 | 44559123 | 122724 | 0.193182 | 0.000099% |
| 268541-26-0 | 91895434 | 122724 | 0.197674 | 0.000489% |
| 283174-18-5 | 16724459 | 122724 | 0.159574 | 0.007738% |
| 2935-32-2 | 14287354 | 122724 | 0.234568 | 0.000029% |
| 31222-32-9 | 90471440 | 122724 | 0.147368 | 0.000400% |
| 31298-06-3 | 15385516 | 122724 | 0.238095 | 0.001278% |
| 32208-45-0 | 636516 | 122724 | 0.202247 | 0.004660% |
| 32303-26-7 | 182076 | 122724 | 0.224719 | 0.000096% |
| 32451-85-7 | 21672675 | 122724 | 0.176471 | 0.001440% |
| 33600-93-0 | 169521 | 122724 | 0.380282 | 0.044000% |
| 34157-83-0 | 122724 | 122724 | 1.000000 | 0.051667% |
| 345289-50-1 | 275400609 | 122724 | 0.148515 | 0.000620% |
| 35761-54-7 | 21625900 | 122724 | 0.149425 | 0.010000% |
| 369647-34-7 | 10323147 | 122724 | 0.250000 | 0.001630% |
| 369647-35-8 | 10323148 | 122724 | 0.238636 | 0.008088% |
| 369647-36-9 | 11048975 | 122724 | 0.337662 | 0.001442% |
| 3779-59-7 | 16020000 | 122724 | 0.117021 | 0.000065% |
| 381691-22-1 | 73554062 | 122724 | 0.320988 | 0.105556% |
| 38242-02-3 | 20055661 | 122724 | 0.246914 | 0.000356% |
| 3877-86-9 | 5281318 | 122724 | 0.166667 | 0.000030% |
| 39903-21-4 | 14108943 | 122724 | 0.263158 | 0.000046% |
| 4373-41-5 | 73659 | 122724 | 0.238095 | 0.131840% |
| 4547-24-4 | 6918774 | 122724 | 0.232558 | 0.102520% |
| 464-98-2 | 3034659 | 122724 | 0.176471 | 0.002429% |
| 465-00-9 | 73641 | 122724 | 0.241379 | 0.002044% |
| 465-74-7 | 120678 | 122724 | 0.188889 | 0.000143% |
| 465-99-6 | 73299 | 122724 | 0.241379 | 0.000080% |
| 468732-43-6 | 637433 | 122724 | 0.182796 | 0.000071% |
| 471-69-2 | 441676 | 122724 | 0.168539 | 1.600000% |
| 472-15-1 | 64971 | 122724 | 0.197674 | ​0.1166% |
| 474-58-8 | 5742590 | 122724 | 0.100000 | 0.000120% |
| 481-18-5 | 5281331 | 122724 | 0.130435 | 0.000200% |
| 494753-69-4 | 11499198 | 122724 | 0.118182 | 0.000560% |
| 50656-68-3 | 73147 | 122724 | 0.606061 | 0.000054% |
| 508-02-1 | 10494 | 122724 | 0.238095 | 0.062910% |
| 50802-21-6 | 101520 | 122724 | 0.640625 | 0.006667% |
| 5085-72-3 | 101341 | 122724 | 0.200000 | 0.002000% |
| 514-07-8 | 92785 | 122724 | 0.192308 | 0.003022% |
| ’5143-05-5 | 15560324 | 122724 | 0.235294 | 0.000078% |
| 516-37-0 | 10181133 | 122724 | 0.142857 | 0.000280% |
| 516-78-9 | 5283646 | 122724 | 0.123596 | 0.040000% |
| 516-79-0 | 5326970 | 122724 | 0.134831 | 0.050000% |
| 52213-27-1 | 15917998 | 122724 | 0.232558 | 0.288000% |
| 53155-25-2 | 471426 | 122724 | 0.235294 | 0.114170% |
| 545-24-4 | 9932254 | 122724 | 0.217949 | 0.000622% |
| 545-47-1 | 259846 | 122724 | 0.170732 | 0.004348% |
| 55497-79-5 | 15767724 | 122724 | 0.198113 | 0.000127% |
| 559-70-6 | 73145 | 122724 | 0.209877 | 0.000250% |
| 559-74-0 | 91472 | 122724 | 0.226667 | 0.012044% |
| 564-14-7 | 91632878 | 122724 | 0.117647 | 0.000080% |
| 57-87-4 | 444679 | 122724 | 0.122222 | 0.070000% |
| 595-15-3 | 115012 | 122724 | 0.186047 | 0.000100% |
| 61448-03-1 | 15127233 | 122724 | 0.182927 | 0.002000% |
| 6199-67-3 | 5281316 | 122724 | 0.168317 | 0.000014% |
| 62025-49-4 | 9918692 | 122724 | 0.121495 | 0.000770% |
| 631-01-6 | 101810 | 122724 | 0.213483 | 0.000538% |
| 63303-42-4 | 11700083 | 122724 | 0.230000 | 0.000160% |
| 63543-53-3 | 101087017 | 122724 | 0.191011 | 0.000101% |
| 638-95-9 | 73170 | 122724 | 0.204819 | 0.000171% |
| 638-96-0 | 12306155 | 122724 | 0.216867 | 0.000500% |
| 639-14-5 | 92825 | 122724 | 0.229885 | 0.000806% |
| 6610-56-6 | 13969554 | 122724 | 0.168675 | 0.002000% |
| 67023-80-7 | 102004580 | 122724 | 0.159292 | 0.024250% |
| 6750-59-0 | 13632872 | 122724 | 0.218391 | 0.000391% |
| 68354-21-2 | 14354066 | 122724 | 0.170000 | 0.000017% |
| 6894-46-8 | 9804114 | 122724 | 0.333333 | 0.141066% |
| 71247-78-4 | 44593364 | 122724 | 0.328947 | 0.006040% |
| 72944-06-0 | 15038289 | 122724 | 0.200000 | 0.032795% |
| 73904-93-5 | 5321282 | 122724 | 0.154639 | 0.000750% |
| 76035-62-6 | 10434225 | 122724 | 0.333333 | 0.003511% |
| 76094-29-6 | 9981416 | 122724 | 0.346154 | 0.346154% |
| 77-52-1 | 64945 | 122724 | 0.220930 | 0.268000% |
| 78516-69-5 | 101051955 | 122724 | 0.235294 | 0.032600% |
| 78574-94-4 | 13943286 | 122724 | 0.146067 | 0.001625% |
| 79955-41-2 | 14194006 | 122724 | 0.180952 | 0.026667% |
| 808769-54-2 | 71307339 | 122724 | 0.268293 | 0.000292% |
| 81907-61-1 | 471003 | 122724 | 0.195652 | 0.004700% |
| 81907-62-2 | 471002 | 122724 | 0.197802 | 0.000800% |
| 83-46-5 | 222284 | 122724 | 0.118280 | 0.002303% |
| 83-48-7 | 5280794 | 122724 | 0.139785 | 0.002650% |
| 83480-64-2 | 137706407 | 122724 | 0.120690 | 0.000240% |
| 83480-65-3 | 21672570 | 122724 | 0.127273 | 0.000470% |
| 84104-70-1 | 174362 | 122724 | 0.181818 | 0.000070% |
| 84104-71-2 | 158477 | 122724 | 0.193182 | 0.000300% |
| 84108-17-8 | 21594203 | 122724 | 0.268293 | 0.000076% |
| 853233-65-5 | 5274618 | 122724 | 0.189189 | 0.000025% |
| 55466-04-1 | 53399223 | 122724 | 0.102941 | 0.000450% |
| 86425-21-0 | 158946 | 122724 | 0.333333 | 0.002430% |
| 86632-20-4 | 20056194 | 122724 | 0.316456 | 0.000241% |
| 88105-29-7 | 90657714 | 122724 | 0.115044 | 0.001040% |
| 89786-83-4 | 12308659 | 122724 | 0.235955 | 0.00028%% |
| 91269-84-0 | 91895471 | 122724 | 0.220930 | 0.000057% |
| 925932-08-7 | 16099423 | 122724 | 0.177083 | 0.002429% |
| 94530-87-7 | 73554080 | 122724 | 0.227848 | 0.000267% |
| 81907-61-1 | 471003 | 122724 | 0.195652 | 0.135000% |
| 97411-50-2 | 91886694 | 122724 | 0.135135 | 0.003188% |
| 98665-19-1 | 471005 | 122724 | 0.183673 | 0.000180% |
| 989-61-7 | 91809625 | 122724 | 0.142857 | 0.000067% |
| 26563-68-8 | 3694932 | 122724 | 0.238095 | 0.003459% |
| 35286-59-0 | 71773126 | 122724 | 0.204082 | 0.218000% |
| 511-01-3 | 11453544 | 122724 | 0.148649 | 0.025000% |
| 10410-83-0 | 99980 | 122724 | 0.193878 | 0.001270% |
| 104700-96-1 | 13934286 | 122724 | 0.170213 | 0.144000% |
| 104700-97-2 | 13934284 | 122724 | 0.191489 | 0.190000% |
| 1063-77-0 | 46783795 | 122724 | 0.161616 | 0.200000% |
| 115334-05-9 | 73351981 | 122724 | 0.164835 | 0.000300% |
| 1154518-97-4 | 44179869 | 122724 | 0.153846 | 0.000420% |
| 1180-71-8 | 179651 | 122724 | 0.135417 | 0.240000% |
| 119725-20-1 | 12045007 | 122724 | 0.265060 | 0.000165% |
| 120211-98-5 | 14312996 | 122724 | 0.226190 | 0.067570% |
| 123135-05-7 | 10838721 | 122724 | 0.227273 | 0.000089% |
| 1241871-28-2 | 46918651 | 122724 | 0.159091 | 0.000215% |
| 125124-68-7 | 14447050 | 122724 | 0.166667 | 0.000182% |
| 1254-85-9 | 21596358 | 122724 | 0.184783 | 0.044048% |
| 125456-52-2 | 15958448 | 122724 | 0.215909 | 0.000054% |
| 13159-28-9 | 99615 | 122724 | 0.157303 | 0.000231% |
| 131984-82-2 | 21672641 | 122724 | 0.217391 | 0.000122% |
| 1337972-99-2 | 56673749 | 122724 | 0.193548 | 0.000027% |
| 1337973-00-8 | 56601655 | 122724 | 0.159574 | 0.000020% |
| 1337973-01-9 | 56601656 | 122724 | 0.164835 | 0.000020% |
| 1337973-03-1 | 56659899 | 122724 | 0.180851 | 0.000054% |
| 1337973-06-4 | 56673750 | 122724 | 0.189474 | 0.000309% |
| 13849-91-7 | 382831 | 122724 | 0.223529 | 0.001435% |
| 13850-16-3 | 73193 | 122724 | 0.235294 | 0.001550% |
| 13956-51-9 | 91895419 | 122724 | 0.162791 | 0.001200% |
| 14351-29-2 | 10895555 | 122724 | 0.134831 | 0.001523% |
| 1449-06-5 | 12309682 | 122724 | 0.162500 | 0.004200% |
| 1449-09-8 | 94204 | 122724 | 0.147727 | 0.004839% |
| 152685-91-1 | 91895424 | 122724 | 0.128440 | 0.000300% |
| 1617-68-1 | 628706 | 122724 | 0.188235 | 0.005477% |
| 1616-93-9 | 92156 | 122724 | 0.235294 | 0.171429% |
| 1617-68-1 | 628706 | 122724 | 0.188235 | 0.371429% |
| 1617-70-5 | 92158 | 122724 | 0.197531 | 0.002867% |
| 162059-94-1 | 15767725 | 122724 | 0.194444 | 0.000080% |
| 167875-39-0 | 91895455 | 122724 | 0.172727 | 0.001240% |
| 16962-90-6 | 76311433 | 122724 | 0.157895 | 0.000250% |
| 170904-49-1 | 15241162 | 122724 | 0.168421 | 0.000492% |
| 182682-97-9 | 85245649 | 122724 | 0.232323 | 0.000017% |
| 18385-59-6 | 122801 | 122724 | 0.177083 | 0.000200% |
| 19533-92-7 | 12305767 | 122724 | 0.209302 | 0.000320% |
| 19865-75-9 | 70690607 | 122724 | 0.180000 | 0.000600% |
| 19902-53-5 | 71448948 | 122724 | 0.111111 | 0.000156% |
| 19942-04-2 | 21575463 | 122724 | 0.114943 | 0.000273% |
| 277751-59-4 | 44583694 | 122724 | 0.190909 | 0.001000% |
| 21302-79-4 | 161352 | 122724 | 0.195402 | 0.000054% |
| 217466-37-0 | 10625284 | 122724 | 0.235294 | 0.001043% |
| 22255-10-3 | 10394654 | 122724 | 0.212766 | 0.171429% |
| 2239-24-9 | 164947 | 122724 | 0.162500 | 0.010900% |
| 22570-53-2 | 159931 | 122724 | 0.132530 | 0.375000% |
| 268214-50-2 | 5468948 | 122724 | 0.160377 | 0.006303% |
| 268214-51-3 | 6479499 | 122724 | 0.174757 | 0.015036% |
| 268214-52-4 | 252435869 | 122724 | 0.138614 | 0.027143% |
| 27013-91-8 | 73296 | 122724 | 0.203704 | 0.373333% |
| 277751-59-4 | 44583694 | 122724 | 0.190909 | 0.001000% |
| 277751-61-8 | 10746421 | 122724 | 0.205882 | 0.000015% |
| 283174-18-5 | 16724459 | 122724 | 0.159574 | 0.007738% |
| 290821-40-8 | 91827210 | 122724 | 0.172727 | 0.000262% |
| 31222-32-9 | 90471440 | 122724 | 0.147368 | 0.000400% |
| 31300-24-0 | 21672698 | 122724 | 0.173913 | 0.002470% |
| 35286-58-9 | 71609288 | 122724 | 0.181818 | 0.000210% |
| 35959-05-8 | 21606663 | 122724 | 0.138298 | 0.258000% |
| 3604-92-0 | 21672659 | 122724 | 0.162500 | 0.001273% |
| 3779-59-7 | 16020000 | 122724 | 0.117021 | 0.001500% |
| 4373-41-5 | 73659 | 122724 | 0.238095 | 0.131840% |
| 4547-24-4 | 6918774 | 122724 | 0.232558 | 0.102520% |
| 455253-27-7 | 636841 | 122724 | 0.202128 | 0.371429% |
| 464-92-6 | 119034 | 122724 | 0.235955 | 0.247000% |
| 465-00-9 | 73641 | 122724 | 0.241379 | 0.002044% |
| 465-74-7 | 120678 | 122724 | 0.188889 | 0.000367% |
| 469-38-5 | 92110 | 122724 | 0.160920 | 0.006452% |
| 469-39-6 | 101690 | 122724 | 0.155556 | 0.000180% |
| 472-15-1 | 64971 | 122724 | 0.197674 | ​0.1166% |
| 472-28-6 | 12302182 | 122724 | 0.175824 | 0.014516% |
| 473554-75-5 | 10930352 | 122724 | 0.125000 | 0.000681% |
| 474-58-8 | 5742590 | 122724 | 0.100000 | 0.000943% |
| 508-02-1 | 10494 | 122724 | 0.238095 | 0.084140% |
| 508-09-8 | 10071029 | 122724 | 0.246753 | 0.000150% |
| 511-01-3 | 11453544 | 122724 | 0.148649 | 0.025000% |
| 52213-27-1 | 15917998 | 122724 | 0.232558 | 0.288000% |
| 53155-25-2 | 471426 | 122724 | 0.235294 | 0.062910% |
| 53755-76-3 | 91895437 | 122724 | 0.170455 | 0.000062% |
| 545-46-0 | 92802 | 122724 | 0.168539 | 0.001000% |
| 545-47-1 | 259846 | 122724 | 0.170732 | 0.400000% |
| 545-48-2 | 101761 | 122724 | 0.188235 | 0.000800% |
| 54963-52-9 | 44593379 | 122724 | 0.232558 | 0.002348% |
| 55497-79-5 | 15767724 | 122724 | 0.198113 | 0.000250% |
| 595-15-3 | 115012 | 122724 | 0.186047 | 0.000100% |
| 62655-03-2 | 181314 | 122724 | 0.187500 | 0.000067% |
| 67106-58-5 | 52951892 | 122724 | 0.215054 | 0.000050% |
| 751-03-1 | 119041 | 122724 | 0.173913 | 0.130000% |
| 77-52-1 | 64945 | 122724 | 0.220930 | 3.000000% |
| 79955-41-2 | 14194006 | 122724 | 0.180952 | 0.026667% |
| 81907-62-2 | 471002 | 122724 | 0.197802 | 0.263000% |
| 81907-63-3 | 21632954 | 122724 | 0.191489 | 0.000110% |
| 853233-65-5 | 5274618 | 122724 | 0.189189 | 0.000025% |
| 865543-37-9 | 46888171 | 122724 | 0.215054 | 0.000140% |
| 942582-15-2 | 16724460 | 122724 | 0.142857 | 0.000205% |
| 95311-94-7 | 14109375 | 122724 | 0.200000 | 0.000270% |
| 1337972-99-2 | 56673749 | 122724 | 0.193548 | 0.000027% |
| 1337973-00-8 | 56601655 | 122724 | 0.159574 | 0.000020% |
| 1337973-01-9 | 56601656 | 122724 | 0.164835 | 0.000020% |
| 1337973-04-2 | 56677063 | 122724 | 0.175258 | 0.000121% |
| 1337973-05-3 | 51042542 | 122724 | 0.180723 | 0.000027% |
| 1337973-07-5 | 56683720 | 122724 | 0.177083 | 0.000034% |
| 53800-21-8 | 10863674 | 122724 | 0.200000 | 0.000030% |
| 277751-59-4 | 44583694 | 122724 | 0.190909 | 0.001000% |
| 100198-09-2 | 12004524 | 122724 | 0.184783 | 0.001450% |
| 103917-26-6 | 73554036 | 122724 | 0.200000 | 0.004000% |
| 104777-61-9 | 91895432 | 122724 | 0.215909 | 0.000400% |
| 115334-04-8 | 189290 | 122724 | 0.191489 | 0.003000% |
| 1180-71-8 | 179651 | 122724 | 0.135417 | 0.240000% |
| 1241871-28-2 | 46918651 | 122724 | 0.159091 | 0.000300% |
| 125276-62-2 | 180311 | 122724 | 0.195652 | 0.004444% |
| 1260173-73-6 | 102263760 | 122724 | 0.148936 | 0.000230% |
| 1318173-53-3 | 102527814 | 122724 | 0.206186 | 0.000240% |
| 16844-71-6 | 119242 | 122724 | 0.200000 | 0.008500% |
| 16962-90-6 | 76311433 | 122724 | 0.157895 | 0.000250% |
| 17990-43-1 | 619168 | 122724 | 0.240964 | 0.000058% |
| 22570-53-2 | 159931 | 122724 | 0.132530 | 0.375000% |
| 226562-47-6 | 71307355 | 122724 | 0.235294 | 0.000100% |
| 2318-78-7 | 10410123 | 122724 | 0.170732 | 0.000325% |
| 23963-54-4 | 14167335 | 122724 | 0.207317 | 0.000200% |
| 247036-52-8 | 21668774 | 122724 | 0.183673 | 0.092000% |
| 29028-10-2 | 488250 | 122724 | 0.168675 | 0.002200% |
| 341971-45-7 | 70680338 | 122724 | 0.225806 | 0.000023% |
| 35286-58-9 | 71609288 | 122724 | 0.181818 | 1.635000% |
| 464-92-6 | 119034 | 122724 | 0.235955 | 0.247000% |
| 465-00-9 | 73641 | 122724 | 0.241379 | 0.002044% |
| 466-01-3 | 12310388 | 122724 | 0.255814 | 0.000082% |
| 466-02-4 | 125114857 | 122724 | 0.244186 | 0.000324% |
| 469-39-6 | 101690 | 122724 | 0.155556 | 0.000300% |
| 471-53-4 | 10114 | 122724 | 0.346154 | 5.920000% |
| 471-66-9 | 637234 | 122724 | 0.268293 | 0.000080% |
| 4965-99-5 | 71307340 | 122724 | 0.183908 | 0.000425% |
| 52914-31-5 | 14137680 | 122724 | 0.152174 | 0.002947% |
| 53755-77-4 | 636674 | 122724 | 0.192771 | 0.002300% |
| 545-46-0 | 92802 | 122724 | 0.168539 | 0.001000% |
| 545-48-2 | 101761 | 122724 | 0.188235 | 0.000800% |
| 7372-30-7 | 6475119 | 122724 | 0.222222 | 0.000500% |
| 6246-46-4 | 9890209 | 122724 | 0.232558 | 0.000667% |
| 64929-59-5 | 15906464 | 122724 | 0.228261 | 0.000019% |
| 6610-55-5 | 13942832 | 122724 | 0.237500 | 0.000291% |
| 6985-35-9 | 21603611 | 122724 | 0.156250 | 0.000100% |
| 73793-68-7 | 156766 | 122724 | 0.193548 | 0.002667% |
| 81241-53-4 | 21632988 | 122724 | 0.173913 | 0.002624% |
| 81371-54-2 | 91895422 | 122724 | 0.123894 | 0.000275% |
| 828935-47-3 | 70698071 | 122724 | 0.168539 | 0.003000% |
| 84104-70-1 | 174362 | 122724 | 0.181818 | 0.010780% |
| 84104-71-2 | 158477 | 122724 | 0.193182 | 0.096296% |
| 85372-70-9 | 44445558 | 122724 | 0.138614 | 0.001477% |
| 890928-81-1 | 91895452 | 122724 | 0.182796 | 0.000309% |
| 912329-03-4 | 16079963 | 122724 | 0.144330 | 0.000056% |
| 1337973-04-2 | 56677063 | 122724 | 0.175258 | 0.000121% |
| 1337973-06-4 | 56673750 | 122724 | 0.189474 | 0.000309% |
| 1337973-07-5 | 56683720 | 122724 | 0.177083 | 0.000034% |
| 1405-86-3 | 14982 | 122724 | 0.288660 | 10.330000% |
| 143519-04-4 | 91668425 | 122724 | 0.186813 | 0.001392% |
| 143839-01-4 | 347673005 | 122724 | 0.225806 | 0.000027% |
| 168254-95-3 | 12302572 | 122724 | 0.241379 | 0.000670% |
| 22318-10-1 | 185481 | 122724 | 0.189474 | 0.000101% |
| 247036-52-8 | 21668774 | 122724 | 0.183673 | 0.092000% |
| 4184-34-3 | 45270099 | 122724 | 0.177778 | 0.002600% |
| 4373-41-5 | 73659 | 122724 | 0.238095 | 0.131840% |
| 4481-62-3 | 122844 | 122724 | 0.223529 | 0.000220% |
| 50656-68-3 | 73147 | 122724 | 0.606061 | 0.000500% |
| 612836-45-0 | 11091624 | 122724 | 0.507042 | 0.000600% |
| 751-03-1 | 119041 | 122724 | 0.173913 | 0.130000% |
| 83-46-5 | 222284 | 122724 | 0.118280 | 0.002303% |
| 84108-17-8 | 21594203 | 122724 | 0.268293 | 0.000315% |
| 85372-65-2 | 44577792 | 122724 | 0.145833 | 0.000289% |
| 1337973-06-4 | 56673750 | 122724 | 0.189474 | 0.000309% |
| 377724-68-0 | 489940 | 122724 | 0.142857 | 0.000050% |
| 1020074-97-8 | 73554035 | 122724 | 0.147727 | 0.000037% |
| 1043629-23-7 | 73554037 | 122724 | 0.066667 | 0.001657% |
| 107160-24-7 | 91895433 | 122724 | 0.152174 | 0.016600% |
| 113558-03-5 | 69624177 | 122724 | 0.226190 | 0.005000% |
| 1189801-51-1 | 91895445 | 122724 | 0.180952 | 0.000146% |
| 119725-19-8 | 490364 | 122724 | 0.235294 | 0.001875% |
| 119725-20-1 | 12045007 | 122724 | 0.265060 | 0.000165% |
| 121825-43-2 | 274041706 | 122724 | 0.149533 | 0.000100% |
| 1220508-29-1 | 71716357 | 122724 | 0.122642 | 0.000213% |
| 1221262-77-6 | 46210176 | 122724 | 0.236559 | 0.002413% |
| 1242085-06-8 | 129316641 | 122724 | 0.159091 | 0.002700% |
| 127-22-0 | 92097 | 122724 | 0.179487 | 0.000343% |
| 13072-74-7 | 1886 | 122724 | 0.164835 | 0.000026% |
| 1351617-74-7 | 56838493 | 122724 | 0.164948 | 0.000080% |
| 13849-91-7 | 382831 | 122724 | 0.223529 | 0.001435% |
| 13947-03-0 | 6708634 | 122724 | 0.175258 | 0.000213% |
| 148044-47-7 | 252551523 | 122724 | 0.172414 | 0.002286% |
| 1514669-21-6 | 137795474 | 122724 | 0.194175 | 0.005000% |
| 155060-48-3 | 101929808 | 122724 | 0.211765 | 0.002857% |
| 1615-94-7 | 12442794 | 122724 | 0.156627 | 0.000305% |
| 16566-88-4 | 91895444 | 122724 | 0.138889 | 0.000162% |
| 167875-39-0 | 91895455 | 122724 | 0.172727 | 0.001240% |
| 173991-81-6 | 73554051 | 122724 | 0.213483 | 0.000026% |
| 17884-88-7 | 3084282 | 122724 | 0.154762 | 0.000500% |
| 1802-12-6 | 21594228 | 122724 | 0.266667 | 1.833333% |
| 19865-87-3 | 44421647 | 122724 | 0.146341 | 0.038500% |
| 2023822-39-9 | 134715088 | 122724 | 0.170213 | 0.000421% |
| 2023822-40-2 | 134715091 | 122724 | 0.173913 | 0.000516% |
| 2023822-41-3 | 134715090 | 122724 | 0.153153 | 0.003063% |
| 21671-01-2 | 44421646 | 122724 | 0.157895 | 0.000048% |
| 217466-37-0 | 76532587 | 122724 | 0.235294 | 0.001043% |
| 2189-80-2 | 94225 | 122724 | 0.207317 | 0.000514% |
| 22255-07-8 | 40469567 | 122724 | 0.163462 | 0.000789% |
| 471-69-2 | 441676 | 122724 | 0.168539 | 1.600000% |
| 22558-20-9 | 185500 | 122724 | 0.164835 | 0.000100% |
| 2259-07-6 | 13688748 | 122724 | 0.243590 | 0.000279% |
| 24022-48-8 | 71720629 | 122724 | 0.153846 | 0.000250% |
| 25279-15-6 | 14038588 | 122724 | 0.142857 | 0.000360% |
| 25499-90-5 | 11869658 | 122724 | 0.238095 | 0.000400% |
| 2629-14-3 | 419676 | 122724 | 0.155340 | 0.005738% |
| 268541-26-0 | 124222305 | 122724 | 0.197674 | 0.000489% |
| 301530-12-1 | 102004498 | 122724 | 0.130000 | 0.003055% |
| 32206-97-6 | 15559638 | 122724 | 0.166667 | 0.000054% |
| 345289-50-1 | 164148808 | 122724 | 0.180000 | 0.000620% |
| 35833-72-8 | 252420405 | 122724 | 0.147727 | 0.061176% |
| 366450-46-6 | 91895428 | 122724 | 0.166667 | 0.009700% |
| 4339-72-4 | 151202 | 122724 | 0.238636 | 0.000060% |
| 4373-41-5 | 73659 | 122724 | 0.238095 | 0.131840% |
| 465-00-9 | 73641 | 122724 | 0.241379 | 0.000333% |
| 465-99-6 | 73299 | 122724 | 0.241379 | 0.000080% |
| 4871-87-8 | 21594136 | 122724 | 0.241379 | 0.000089% |
| 4966-00-1 | 12305178 | 122724 | 0.151163 | 0.001720% |
| 508-02-1 | 10494 | 122724 | 0.238095 | 0.084140% |
| 510-30-5 | 73309 | 122724 | 0.220930 | 0.097653% |
| ‘5143-05-5 | 348484812 | 122724 | 0.235294 | 0.000078% |
| 13850-16-3 | 73193 | 122724 | 0.235294 | 0.000573% |
| 54963-52-9 | 44593379 | 122724 | 0.232558 | 0.002348% |
| 5532-41-2 | 12305182 | 122724 | 0.151163 | 0.000600% |
| 5595-78-8 | 91885233 | 122724 | 0.177778 | 0.000920% |
| 57586-98-8 | 5270670 | 122724 | 0.176471 | 0.000059% |
| 62498-83-3 | 10929355 | 122724 | 0.184783 | 0.000033% |
| 638-97-1 | 12306160 | 122724 | 0.222222 | 0.000867% |
| 6488-64-8 | 21594175 | 122724 | 0.296296 | 0.000038% |
| 67023-81-8 | 274953239 | 122724 | 0.146552 | 0.000049% |
| 6713-27-5 | 489941 | 122724 | 0.231707 | 0.055000% |
| 71590-47-1 | 326835 | 122724 | 0.147059 | 0.002587% |
| 75069-59-9 | 252076750 | 122724 | 0.175824 | 0.000093% |
| 76689-93-5 | 273551190 | 122724 | 0.125000 | 0.000411% |
| 76689-98-0 | 91895443 | 122724 | 0.123894 | 0.001887% |
| 81241-53-4 | 21632988 | 122724 | 0.173913 | 0.002624% |
| 81678-46-8 | 91895430 | 122724 | 0.188889 | 0.001905% |
| 81907-62-2 | 471002 | 122724 | 0.197802 | 0.000860% |
| 81910-39-6 | 91884853 | 122724 | 0.142857 | 0.001091% |
| 83-46-5 | 222284 | 122724 | 0.118280 | 0.003164% |
| 83-48-7 | 5280794 | 122724 | 0.139785 | 0.002650% |
| 85372-70-9 | 44445558 | 122724 | 0.138614 | 0.001477% |
| 934739-29-4 | 57330179 | 122724 | 0.177083 | 0.000507% |
| 93767-25-0 | 131676077 | 122724 | 0.159574 | 0.000300% |
| 94530-87-7 | 73554080 | 122724 | 0.227848 | 0.000233% |
| 465-00-9 | 73641 | 122724 | 0.241379 | 0.000333% |
| 1197347-40-2 | 44613295 | 122724 | 0.149533 | 0.000125% |
| 6426-44-4 | 1781002 | 122724 | 0.243902 | 0.000442% |
| 1011762-93-8 | 24854383 | 122724 | 0.198113 | 0.000040% |
| 1207861-69-5 | 102165747 | 122724 | 0.115044 | 0.001600% |
| 13849-91-7 | 382831 | 122724 | 0.223529 | 0.000553% |
| 13850-16-3 | 73193 | 122724 | 0.235294 | 0.001550% |
| 13878-92-7 | 14034468 | 122724 | 0.177778 | 0.000580% |
| 1449-09-8 | 94204 | 122724 | 0.147727 | 0.000305% |
| 1617-70-5 | 92158 | 122724 | 0.197531 | 0.000324% |
| 163060-07-9 | 68956386 | 122724 | 0.176471 | 0.002750% |
| 171438-55-4 | 10746256 | 122724 | 0.161905 | 0.005050% |
| 17983-82-3 | 45269256 | 122724 | 0.173913 | 0.010800% |
| 17990-42-0 | 12313704 | 122724 | 0.250000 | 0.004500% |
| 19132-81-1 | 14423521 | 122724 | 0.179775 | 0.000014% |
| 20137-37-5 | 12315075 | 122724 | 0.227273 | 0.000745% |
| 127-22-0 | 344467 | 122724 | 0.179487 | 0.000343% |
| 2111-46-8 | 15598266 | 122724 | 0.184783 | 0.000078% |
| 2189-80-2 | 94225 | 122724 | 0.207317 | 0.003667% |
| ’2259-07-6 | 13688748 | 122724 | 0.243590 | 0.000279% |
| 232266-08-9 | 45271778 | 122724 | 0.229885 | 0.017500% |
| 24778-48-1 | 12016584 | 122724 | 0.223529 | 0.000246% |
| 32214-80-5 | 161739 | 122724 | 0.182796 | 0.006152% |
| 4547-24-4 | 6918774 | 122724 | 0.232558 | 0.010000% |
| 4657-58-3 | 313075 | 122724 | 0.141176 | 0.000125% |
| 469-39-6 | 101690 | 122724 | 0.155556 | 0.000300% |
| 472-15-1 | 64971 | 122724 | 0.197674 | ​0.1166% |
| 508-02-1 | 10494 | 122724 | 0.238095 | 0.062910% |
| 508-09-8 | 10071029 | 122724 | 0.246753 | 0.000150% |
| 5132-66-1 | 45270932 | 122724 | 0.197802 | 0.121000% |
| 53155-25-2 | 471426 | 122724 | 0.235294 | 0.114170% |
| 53822-99-4 | 274885938 | 122724 | 0.112360 | 0.000013% |
| 545-24-4 | 9932254 | 122724 | 0.217949 | 0.000622% |
| 55306-03-1 | 124214 | 122724 | 0.241379 | 0.001938% |
| 57475-62-4 | 21594257 | 122724 | 0.142857 | 0.000070% |
| 63303-42-4 | 11700083 | 122724 | 0.230000 | 0.000197% |
| 64199-78-6 | 194183 | 122724 | 0.227273 | 0.000392% |
| 6426-44-4 | 1781002 | 122724 | 0.243902 | 0.000442% |
| 654663-85-1 | 91886677 | 122724 | 0.191489 | 0.000811% |
| 68800-57-7 | 20055826 | 122724 | 0.227273 | 0.000347% |
| 77-52-1 | 64945 | 122724 | 0.220930 | 3.000000% |
| 82464-35-5 | 158100 | 122724 | 0.204301 | 0.000259% |
| 83-48-7 | 5280794 | 122724 | 0.139785 | 0.002650% |
| 87355-32-6 | 12111950 | 122724 | 0.219512 | 0.083333% |
| 89786-84-5 | 182497 | 122724 | 0.238636 | 0.002519% |
| 989-30-0 | 7163177 | 122724 | 0.220930 | 0.000088% |
| 1059-14-9 | 115250 | 122724 | 0.152941 | 0.000300% |
| 120211-98-5 | 14312996 | 122724 | 0.226190 | 0.067570% |
| 125263-66-3 | 21594133 | 122724 | 0.220930 | 0.000108% |
| 13878-92-7 | 14034468 | 122724 | 0.177778 | 0.000580% |
| 143773-52-8 | 9898761 | 122724 | 0.221154 | 0.000148% |
| 160481-71-0 | 131849190 | 122724 | 0.170455 | 0.000192% |
| 1616-93-9 | 92156 | 122724 | 0.235294 | 0.000400% |
| 1622394-64-2 | 275221798 | 122724 | 0.150442 | 0.000135% |
| 1622394-65-3 | 275221799 | 122724 | 0.203883 | 0.000438% |
| 17184-21-3 | 441928 | 122724 | 0.224490 | 0.000920% |
| 171864-20-3 | 11135961 | 122724 | 0.225490 | 0.000150% |
| 17320-15-9 | 44423588 | 122724 | 0.147727 | 0.000035% |
| 2189-80-2 | 94225 | 122724 | 0.207317 | 0.000163% |
| 2318-78-7 | 10410123 | 122724 | 0.170732 | 0.000325% |
| 31298-06-3 | 15385516 | 122724 | 0.238095 | 0.001278% |
| 41753-43-9 | 9898279 | 122724 | 0.112069 | 0.880000% |
| 4184-34-3 | 45270099 | 122724 | 0.177778 | 0.002600% |
| 495389-95-2 | 125115276 | 122724 | 0.180000 | 0.000292% |
| 559-70-6 | 73145 | 122724 | 0.209877 | 0.000333% |
| 559-74-0 | 91472 | 122724 | 0.226667 | 0.012044% |
| 58812-37-6 | 9851101 | 122724 | 0.148515 | 0.014667% |
| 22255-10-3 | 131752026 | 122724 | 0.212766 | 0.171429% |
| 67253-01-4 | 21625899 | 122724 | 0.114943 | 0.019250% |
| 77-52-1 | 64945 | 122724 | 0.220930 | 0.000933% |
| 105742-79-8 | 21636089 | 122724 | 0.193548 | 0.000100% |
| 110267-46-4 | 44566900 | 122724 | 0.180851 | 0.000214% |
| 1276655-49-2 | 71718315 | 122724 | 0.193548 | 0.000009% |
| 132746-04-4 | 195724 | 122724 | 0.190476 | 0.001000% |
| 13843-88-4 | 42608303 | 122724 | 0.135802 | 0.040074% |
| 13849-91-7 | 382831 | 122724 | 0.223529 | 0.000707% |
| 13850-16-3 | 73193 | 122724 | 0.235294 | 0.001550% |
| 146450-83-1 | 91895465 | 122724 | 0.217822 | 0.011870% |
| 1615-99-2 | 441679 | 122724 | 0.142857 | 0.327206% |
| 252351-96-5 | 10366713 | 122724 | 0.164948 | 0.002667% |
| 26575-93-9 | 14036813 | 122724 | 0.166667 | 0.000165% |
| 277751-61-8 | 10746421 | 122724 | 0.205882 | 0.000015% |
| 3668-14-2 | 3080597 | 122724 | 0.166667 | 0.000055% |
| 467-81-2 | 6436598 | 122724 | 0.239583 | 0.001400% |
| 467-82-3 | 15560077 | 122724 | 0.244681 | 0.002000% |
| 50298-90-3 | 10481797 | 122724 | 0.168421 | 0.718519% |
| 508-02-1 | 10494 | 122724 | 0.238095 | 0.084140% |
| 514-47-6 | 441678 | 122724 | 0.168539 | 0.003922% |
| 55306-03-1 | 124214 | 122724 | 0.241379 | 0.001938% |
| 559-68-2 | 489944 | 122724 | 0.219512 | 0.000055% |
| 564-13-6 | 12314613 | 122724 | 0.227273 | 0.004222% |
| 58546-34-2 | 181183 | 122724 | 0.171717 | 0.925926% |
| 6040-19-3 | 5281315 | 122724 | 0.186275 | 0.100000% |
| 60657-41-2 | 44584761 | 122724 | 0.222222 | 0.026600% |
| 62369-72-6 | 52951052 | 122724 | 0.207921 | 0.001111% |
| 65247-27-0 | 6441104 | 122724 | 0.145299 | 0.001200% |
| 7518-22-1 | 10079497 | 122724 | 0.201835 | 0.000163% |
| 88930-15-8 | 137705614 | 122724 | 0.188889 | 0.001917% |
| 89590-95-4 | 102004786 | 122724 | 0.134454 | 0.179710% |
| 89786-84-5 | 490367 | 122724 | 0.238636 | 0.002519% |
| 95298-47-8 | 14019178 | 122724 | 0.207921 | 0.025000% |
| 95311-97-0 | 471004 | 122724 | 0.195652 | 0.472000% |
| 62369-72-6 | 52951052 | 122724 | 0.207921 | 0.001111% |
| 105742-79-8 | 21636089 | 122724 | 0.193548 | 0.000100% |
| 10376-48-4 | 12315507 | 122724 | 0.172414 | 0.000422% |
| 10483-91-7 | 345509 | 122724 | 0.186047 | 0.004462% |
| 105742-79-8 | 21636089 | 122724 | 0.193548 | 0.000100% |
| 1058-61-3 | 5484202 | 122724 | 0.130435 | 0.000280% |
| 107176-31-8 | 70698213 | 122724 | 0.156250 | 0.000600% |
| 108544-40-7 | 348541700 | 122724 | 0.204082 | 0.000035% |
| 114912-36-6 | 21626484 | 122724 | 0.180180 | 0.000098% |
| 1172-78-7 | 14605585 | 122724 | 0.144737 | 0.001838% |
| 1189131-72-3 | 44557350 | 122724 | 0.227723 | 0.000167% |
| 1207106-20-4 | 104244446 | 122724 | 0.176471 | 0.000100% |
| 1207106-21-5 | 46184563 | 122724 | 0.181818 | 0.000150% |
| 1207106-22-6 | 46184564 | 122724 | 0.181818 | 0.000040% |
| 122738-86-7 | 57399249 | 122724 | 0.168317 | 0.003000% |
| 125276-62-2 | 180311 | 122724 | 0.195652 | 0.004444% |
| 126105-12-2 | 71307460 | 122724 | 0.134454 | 0.026087% |
| 13159-28-9 | 99615 | 122724 | 0.157303 | 0.000231% |
| 13201-14-4 | 267250 | 122724 | 0.181818 | 0.015704% |
| 13224-63-0 | 6438155 | 122724 | 0.229167 | 0.000150% |
| 132294-77-0 | 195707 | 122724 | 0.176471 | 0.001600% |
| 13849-90-6 | 12314450 | 122724 | 0.235294 | 0.000267% |
| 13849-91-7 | 382831 | 122724 | 0.223529 | 0.000553% |
| 138884-84-1 | 101616678 | 122724 | 0.405405 | 0.000835% |
| 13956-51-9 | 91895419 | 122724 | 0.162791 | 0.001200% |
| 14216-03-6 | 11491905 | 122724 | 0.178862 | 0.001790% |
| 14440-40-5 | 12302400 | 122724 | 0.170732 | 0.000189% |
| 14440-41-6 | 7330581 | 122724 | 0.185185 | 0.002785% |
| 1449-06-5 | 12309682 | 122724 | 0.162500 | 0.004200% |
| 1449-08-7 | 14635659 | 122724 | 0.170455 | 0.000750% |
| 1617-68-1 | 92157 | 122724 | 0.188235 | 0.005477% |
| 167875-33-4 | 10366522 | 122724 | 0.379747 | 0.073333% |
| 167882-66-8 | 10096097 | 122724 | 0.558824 | 0.001567% |
| 16844-71-6 | 119242 | 122724 | 0.200000 | 0.008500% |
| 176983-21-4 | 637229 | 122724 | 0.258824 | 0.000138% |
| 17990-42-0 | 12313704 | 122724 | 0.250000 | 0.004500% |
| 182249-69-0 | 53322486 | 122724 | 0.238636 | 0.000190% |
| 18444-66-1 | 5281319 | 122724 | 0.170000 | 0.000025% |
| 19132-81-1 | 14423521 | 122724 | 0.179775 | 0.000014% |
| 20137-37-5 | 12315075 | 122724 | 0.227273 | 0.000745% |
| 20183-47-5 | 21588226 | 122724 | 0.192308 | 0.031111% |
| 2034-74-4 | 160608 | 122724 | 0.150538 | 0.000110% |
| 20736-09-8 | 11167 | 122724 | 0.123894 | 0.310000% |
| 20874-52-6 | 3722004 | 122724 | 0.123894 | 0.165000% |
| 214351-30-1 | 102119762 | 122724 | 0.149425 | 0.007000% |
| ‘2222-07-3 | 5281321 | 122724 | 0.168421 | 0.000024% |
| 222294-61-3 | 134714938 | 122724 | 0.172414 | 0.327206% |
| 81907-61-1 | 471003 | 122724 | 0.195652 | 0.135000% |
| 2239-24-9 | 164947 | 122724 | 0.162500 | 0.001140% |
| 53755-77-4 | 50593378 | 122724 | 0.000000 | 0.002300% |
| 23290-26-8 | 12795736 | 122724 | 0.115789 | 0.000014% |
| 2469-34-3 | 12442762 | 122724 | 0.170213 | 0.000750% |
| 287475-13-2 | 273047764 | 122724 | 0.117021 | 0.004167% |
| 31297-79-7 | 3052779 | 122724 | 0.210000 | 0.000500% |
| 31298-06-3 | 15385516 | 122724 | 0.238095 | 0.001278% |
| 33600-93-0 | 169521 | 122724 | 0.380282 | 0.044000% |
| 35012-08-9 | 14543446 | 122724 | 0.177778 | 0.000320% |
| 35790-95-5 | 441929 | 122724 | 0.198113 | 0.000891% |
| 364622-33-3 | 21592283 | 122724 | 0.200000 | 0.000070% |
| 3877-86-9 | 5281318 | 122724 | 0.166667 | 0.000030% |
| 42719-32-4 | 14286954 | 122724 | 0.194175 | 0.000035% |
| 465-00-9 | 73641 | 122724 | 0.241379 | 0.002044% |
| 468-67-7 | 271910 | 122724 | 0.250000 | 0.000557% |
| 469-39-6 | 101690 | 122724 | 0.155556 | 0.000300% |
| 481-18-5 | 5281331 | 122724 | 0.130435 | 0.000800% |
| 50298-90-3 | 72421 | 122724 | 0.168421 | 0.718519% |
| 511-01-3 | 11453544 | 122724 | 0.148649 | 0.000352% |
| 51511-05-8 | 10787860 | 122724 | 0.177215 | 0.006000% |
| 53755-77-4 | 636674 | 122724 | 0.192771 | 0.002300% |
| 53800-21-8 | 42608307 | 122724 | 0.200000 | 0.000100% |
| 54352-47-5 | 3041569 | 122724 | 0.144737 | 0.001471% |
| 559-74-0 | 91472 | 122724 | 0.226667 | 0.012044% |
| 561-47-7 | 45268134 | 122724 | 0.244898 | 0.001300% |
| 578710-52-8 | 134714937 | 122724 | 0.212766 | 0.000893% |
| 58316-41-9 | 21637642 | 122724 | 0.141593 | 0.000440% |
| 58316-42-0 | 21637635 | 122724 | 0.130435 | 0.000580% |
| 58546-34-2 | 181183 | 122724 | 0.171717 | 0.925926% |
| 58558-08-0 | 9875547 | 122724 | 0.141593 | 0.000400% |
| 58880-25-4 | 12444386 | 122724 | 0.241379 | 0.011111% |
| 6199-67-3 | 5281316 | 122724 | 0.168317 | 0.000014% |
| 62319-70-4 | 14658050 | 122724 | 0.207921 | 0.022222% |
| 62369-72-6 | 52951052 | 122724 | 0.207921 | 0.001111% |
| 647856-35-7 | 11203394 | 122724 | 0.183673 | 0.002949% |
| 648430-32-4 | 11271456 | 122724 | 0.193548 | 0.001314% |
| 6786-16-9 | 14485466 | 122724 | 0.168675 | 0.000100% |
| 68354-21-2 | 14354067 | 122724 | 0.170000 | 0.000017% |
| 89786-84-5 | 490367 | 122724 | 0.238636 | 0.002519% |
| 71247-78-4 | 44593364 | 122724 | 0.328947 | 0.006040% |
| 72633-85-3 | 16212876 | 122724 | 0.160494 | 0.001029% |
| 73793-68-7 | 274393121 | 122724 | 0.193548 | 0.002667% |
| 77-52-1 | 64945 | 122724 | 0.220930 | 3.000000% |
| 81907-62-2 | 471002 | 122724 | 0.197802 | 0.263000% |
| 81907-63-3 | 21632954 | 122724 | 0.191489 | 0.000110% |
| 81907-65-5 | 21632955 | 122724 | 0.189474 | 0.000090% |
| 83-46-5 | 222284 | 122724 | 0.118280 | 0.002650% |
| 83-48-7 | 5280794 | 122724 | 0.139785 | 0.002650% |
| 84104-70-1 | 174362 | 122724 | 0.181818 | 0.010780% |
| 84104-71-2 | 158477 | 122724 | 0.193182 | 0.096296% |
| 86377-52-8 | 57397445 | 122724 | 0.204301 | 0.000023% |
| 86632-20-4 | 20056194 | 122724 | 0.316456 | 0.000241% |
| 68800-57-7 | 20055826 | 122724 | 0.227273 | 0.000347% |
| 88515-58-6 | 21122581 | 122724 | 0.207921 | 0.005000% |
| 88901-36-4 | 24721270 | 122724 | 0.134454 | 0.067633% |
| 906320-82-9 | 12309889 | 122724 | 0.164557 | 0.017206% |
| 95298-47-8 | 14019178 | 122724 | 0.207921 | 0.025000% |
| 98665-10-2 | 21633085 | 122724 | 0.175824 | 0.000050% |
| 98665-11-3 | 21633082 | 122724 | 0.168317 | 0.000080% |
| 98665-12-4 | 23247892 | 122724 | 0.187500 | 0.007426% |
| 98665-15-7 | 23247895 | 122724 | 0.187500 | 0.002145% |
| 98665-16-8 | 23247891 | 122724 | 0.191489 | 0.065442% |
| 98665-18-0 | 74028536 | 122724 | 0.193182 | 0.000724% |
| 98665-21-5 | 475412 | 122724 | 0.186813 | 0.000220% |
| 98718-43-5 | 21632959 | 122724 | 0.172043 | 0.000020% |
| 99365-19-2 | 101826550 | 122724 | 0.160377 | 0.003600% |
| 1207106-19-1 | 46881265 | 122724 | 0.180000 | 0.000300% |
| 222294-61-3 | 134714938 | 122724 | 0.172414 | 0.327206% |
